# Regiocontrolled One-Step Indole Synthesis from Carbonyl Compounds via Organobismuth Chemistry

**DOI:** 10.1021/jacs.6c15371

**Published:** 2026-10-19

**Authors:** Filippo Carpaneto, Li Li, Viresh H. Rawal

**Affiliations:** Department of Chemistry, 2462 University of Chicago, Chicago, Illinois 60637, United States

## Abstract

Indoles are privileged scaffolds in natural products, pharmaceuticals, and synthetic intermediates, and their concise, regioselective synthesis remains an enduring challenge. Here, we report a one-step indole synthesis enabled by 2-aminophenyl-*t*BuBisTrip (aryl–BisTrip) (**2**), a bismuth­(V) reagent that effects rapid α-arylation of lithium enolates followed by spontaneous annulation. The method is effective for a wide range of cyclic and acyclic ketones, including challenging cyclopentanones and heterocyclic substrates, as well as 1,2- and 1,3-dicarbonyl compounds and enolates derived from silyl enol ethers. Notably, the platform enables direct indole annulation of enones, a substrate class that has remained problematic via Fischer or transition-metal-catalyzed methods. The utility of the transformation is further illustrated by late-stage indole installation on complex bioactive molecules and by a desymmetrization sequence that translates asymmetric deprotonation into chiral indole synthesis. Together, these results establish a general main-group-enabled approach for regiocontrolled indole construction from simple carbonyl precursors.

## Graphical abstract

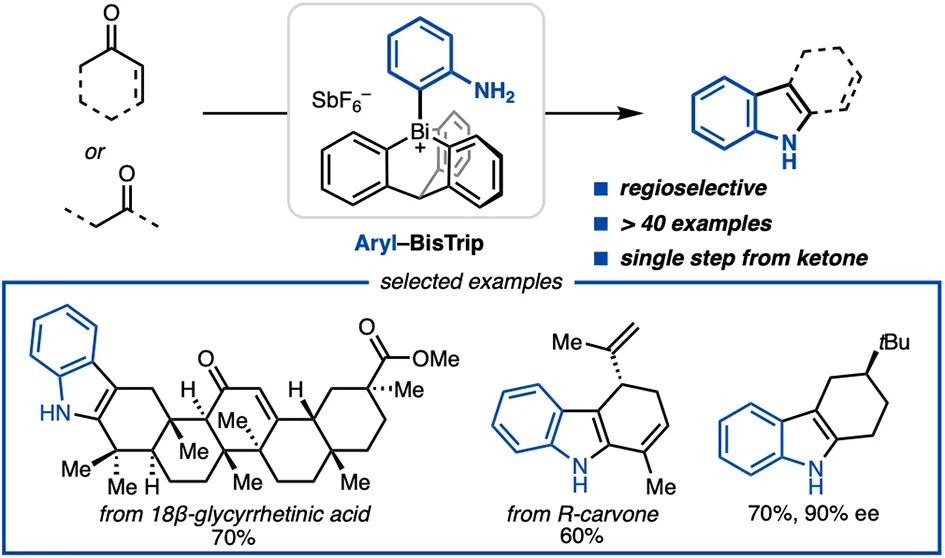

## Introduction

Indoles occupy a position of prominence in the hierarchy of heteroaromatic scaffolds, occurring with striking frequency in bioactive natural products, synthetic intermediates, and modern pharmaceuticals (Figure 1A).^1–4^ Their prevalence and rich reactivity profile have motivated a long-standing interest in the development of concise and regioselective strategies for their construction. Among the diverse paradigms for indole synthesis, approaches based on carbonyl compounds have garnered significant attention, reflecting the versatility of this functional group and the conceptual simplicity of the corresponding disconnection.^5^ Yet, translating this disconnection logic into concise, broadly applicable, and regioselective indole syntheses remains an enduring challenge, particularly for unsymmetrical ketones, enones, and structurally complex carbonyl compounds.

**Figure 1:**
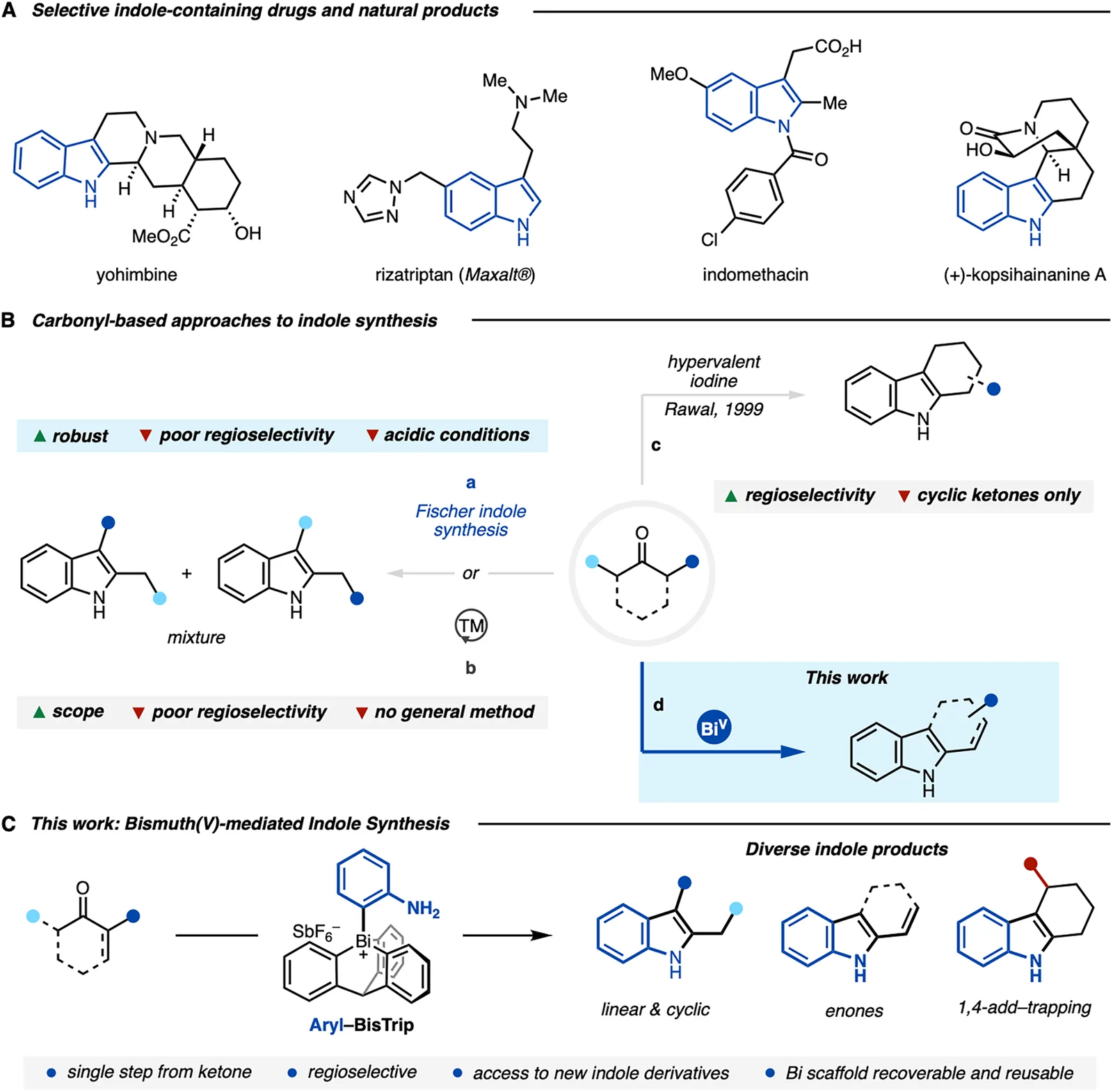
Overview of synthetic strategies for indole construction. (A) Selected indole-containing natural products and pharmaceuticals of interest. (B) Currently available synthetic methods. (C) This work: aryl-BisTrip enabled direct synthesis of indoles.

In this context, the venerable Fischer indole synthesis (Figure 1Ba) remains widely utilized owing to its robustness.^6,7^ Additionally, the development of transition-metal-catalyzed transformations (Figure 1Bb) over the past several decades has greatly expanded the synthetic logic available for indole construction. The latter generally proceed either via an aniline–ketone condensation followed by intramolecular Heck-type cyclization or via C–N bond formation through the cross-coupling of ketone-derived imines with ortho-functionalized arenes.^8–11^ Although both approaches are highly efficacious and offer versatile toolkits for indole construction, each is subject to its own distinct limitations. The Fischer indole synthesis can present regioselectivity challenges with unsymmetrical ketones and compatibility issues with acid-sensitive functional groups under the strongly acidic conditions typically used.^12,13^ While milder acid and Lewis-acid-catalyzed as well as room-temperature protocols have expanded functional group tolerance and improved regiocontrol, orthogonal strategies that operate under nonacidic regimes remain highly desirable.^14^ Similarly, transition-metal-catalyzed methods can still present challenges in controlling regioselectivity with unsymmetrical ketones,^9b,11c^ while certain classes of carbonyl substrates may require distinct modes of substrate activation or prefunctionalization, thereby limiting their generality.^9d,11a^

On the other hand, main-group-mediated strategies for indole construction can provide a complementary approach to some transition-metal-catalyzed transformations.^15–18^ These protocols offer operational simplicity while obviating the issues associated with transition-metal residues in the final products. These strategies, however, come at the expense of substrate scope: they are restricted to narrow classes of carbonyl compounds or rely on pretethered reacting sites and specialized auxiliary groups, diminishing their versatility. To address some of the limitations, we designed new main-group reagents for indole assembly, culminating in *o*-nitrophenylphenyliodonium fluoride (NPIF), which enables regioselective indole synthesis via α-arylation of silyl enol ethers followed by reductive annulation (Figure 1Bc).^18^ While this two-step sequence (three steps from the ketone) delivered excellent regiocontrol, its utility was circumscribed to simple cyclic frameworks, and some classes of substrates, such as acyclic and enone-derived compounds, proved ineffective. The many advances in indole synthesis notwithstanding, the direct and regiocontrolled conversion of structurally diverse carbonyl compounds to indoles remains a worthy challenge.

In our ongoing efforts to expand main-group-mediated transformations, we recently reported 9-bismatriptycene (BisTrip), a novel bismuth-based scaffold that functions as a universal aryl group transporter for the regiospecific α-arylation of carbonyl compounds.^19^ Given its distinctive reactivity and broad functional group tolerance, we envisioned that the BisTrip framework could provide a more general solution to the direct conversion of carbonyl compounds to indoles while overcoming some limitations of current methods (Figure 1Bd). Herein, we report the development of 2-aminophenyl-*t*BuBisTrip, a reagent that effects selective aryl transfer and annulation in a single step, thereby providing direct, regiocontrolled access to indoles from a wide range of carbonyl substrates (Figure 1C).

## Results and Discussion

### Reagent Development

We initiated our studies by considering the ideal bismuth reagent for this transformation: a BisTrip scaffold bearing an unprotected 2-aminophenyl group. This target reagent, however, presents several inherent challenges. First, the hypervalent bismuth­(V) intermediate is highly oxidizing, raising concerns that transmetalation to install the electron-rich aniline unit could lead to its oxidative destruction. Second, even if the aniline was successfully installed, the electron-rich amino group might coordinate, either intra- or intermolecularly, with the highly electrophilic and coordinatively unsaturated bismuth­(V) center, thereby attenuating its reactivity toward enolate nucleophiles. Lastly, upon addition of the enolate partner, proton transfer from the free NH_2_ group would quench the enolate, thereby precluding the desired coupling. Although we considered using a suitably protected aniline (for example, as a phthalimide), we elected to pursue the ideal reagent. Among the BisTrip derivatives we examined, we found 3-*tert*-butyl-9-bismatriptycene (*t*BuBisTrip) **1** to be well-suited for the desired aryl transport. This next-generation platform was prepared on a multigram scale in three steps from commercially available starting materials in 57% overall yield (Figure 2A). Crucially, this engineered scaffold fully retains the exceptional aryl-transfer properties of the parent system while lowering the synthetic burden required for its preparation. Consistent with the anticipated liabilities, our initial attempts to prepare 2-aminophenyl-*t*BuBisTrip salt **2** from **1** using our previously reported standard protocol proved to be challenging.^19,20^ However, careful optimization of the reaction conditions ultimately enabled its synthesis by a protection-free, one-pot protocol (Figure 2, Table S1). This method smoothly afforded aryl-*t*BuBisTrip **2** in 61% yield on a multigram scale after straightforward chromatographic purification (Figure 2B).

**Figure 2:**
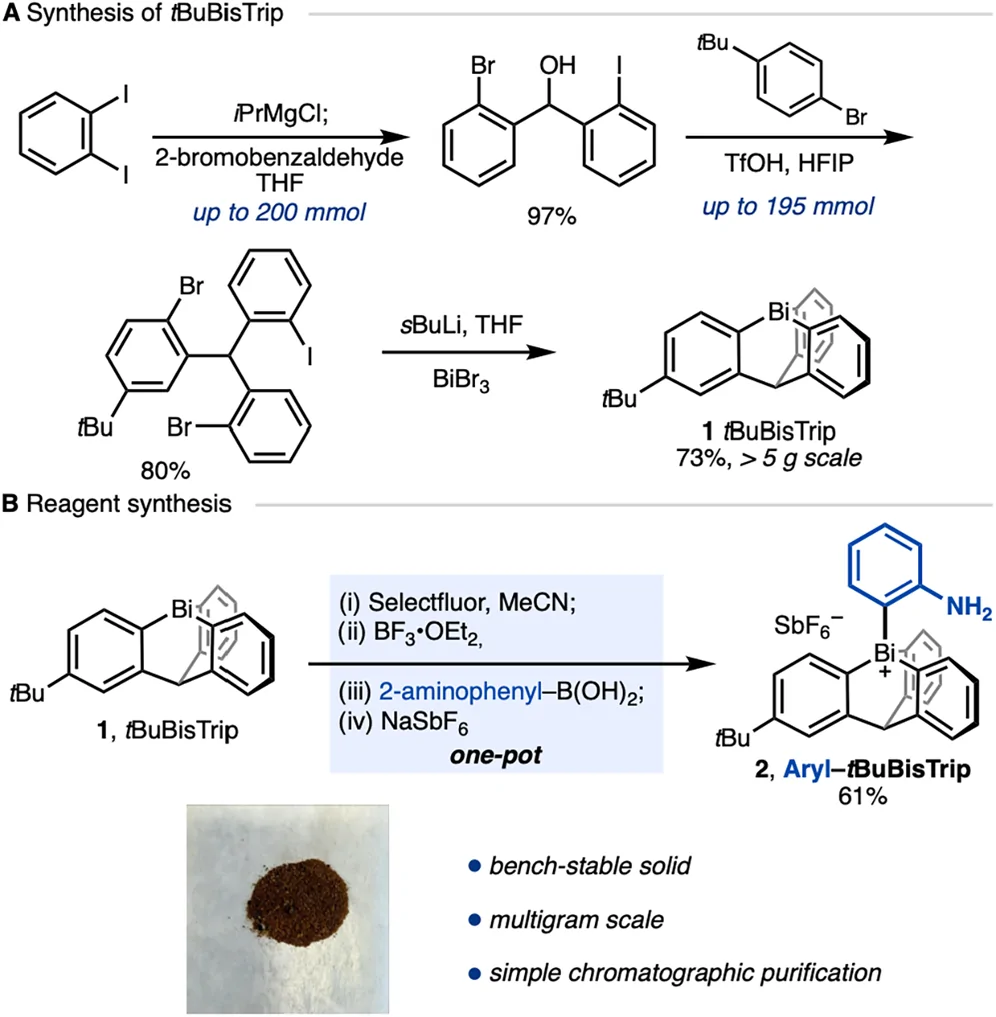
(A) Synthesis of *t*BuBisTrip **1**. (B) Synthesis and picture of aryl-*t*BuBisTrip reagent **2**.

### Reaction Optimization

With reagent **2** in hand, we examined its reactivity toward enolates using 4-*tert*-butylcyclohexanone **3c** as a model substrate. To our delight, treatment of the lithium enolate of **3c** with reagent **2** resulted in rapid α-arylation and spontaneous annulation to afford indole **4c** in 78% yield, along with >95% recovery of *t*BuBisTrip **1** (Table 1, entry 1) and 12% of starting ketone **3c**. The feared proton abstraction from the aniline nitrogen did not derail the desired aryl-transfer reaction. We further investigated the effect of different metal counterions on the transformation. Interestingly, the yield decreased along the sequence Li > Na > K (entries 2–4), in contrast to our previous observations.^19^ The higher reactivity of the sodium and potassium species likely promotes competitive acid–base reaction with the reagent’s aniline protons, thereby diverting material from the productive aryl-transfer pathway.^21^ In addition, the use of more tightly coordinating cations, such as Zn or Mg, or increasing the equivalents of base did not improve the yield (entries 5–7). For direct comparison with established organobismuth chemistry, we also prepared and evaluated the Barton-type analog of reagent **2**, Ph_3_Bi–(*o*-NH_2_)­Ph^+^BF_4_^–^ **2a**.^22^ Subjecting the lithium enolate of **3c** to this reagent afforded the annulated product **4c** in only 26% yield (entry 9), and use of the SbF_6_^–^ salt of this reagent did not improve the outcome. Curiously, only 21% of triphenylbismuth was recovered from the reaction mixture, the remainder consisting of other Ar_3_Bi species, presumably arising through dismutation.^23^ These results demonstrate that the absence of a rigid scaffold allows ligand scrambling and impedes productive aryl transfer, underscoring the unique capability of the rigid BisTrip framework.

**Table 1:**
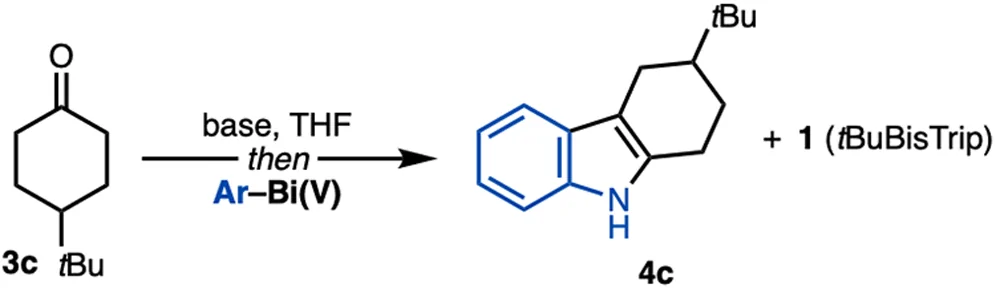
Optimization of the Reaction Conditions^a^

entry	base	additive	Bi(V) reagent	yield of 4c (%)^b^
1	LDA	–	**2**	78
**2**	**LiHMDS**	–	**2**	**81**
3	NaHMDS	–	**2**	65
4	KHMDS	–	**2**	40
5	LiHMDS	MgCl_2_	**2**	62
6	LiHMDS	ZnCl_2_	**2**	65
7	LiHMDS^c^	–	**2**	39
8	*t*-BuOK	–	**2**	63
9	LiHMDS	–	**2a** ^d^	26

a Reaction conditions: ketone **3c** (0.1 mmol, 1.0 equiv), THF (1.0 mL), base (0.11 mmol, 1.1 equiv), –78 °C, 1h; Bi­(V) reagent (0.13 mmol, 1.3 equiv), –78 °C to rt over 15 min.

b Determined by ^1^H NMR.

c LiHMDS (0.22 mmol, 2.0 equiv) was used.

d **2a**: Ph_3_Bi–(*o*-NH_2_)­Ph^+^BF_4_^–^. Entries 1 to 8: > 95% recovery of *t*BuBisTrip; entry 9:21% recovery of Ph_3_Bi.

### Substrate Scope

With the optimized conditions in hand, we investigated the scope of the one-step indole synthesis (Table 2). The reaction proved highly effective across a series of six-membered cyclic ketones possessing different substituents, smoothly delivering the corresponding indoles in 64–77% yield (**4a–4d**, Table 2A). The system demonstrated good compatibility even with a substrate bearing a basic functionality (**4e**, 75%). Cyclopentanone, considered a difficult substrate for α-arylation because of competing self-aldol condensation and polyarylation, underwent efficient coupling with reagent **2** to afford indole **4f** in good yield.^9a,24,25^ Larger cyclic ketones also proved to be effective substrates, delivering the corresponding indoles in high yields (**4g, 4h**, 87% and 85%). It is noteworthy that this methodology provides quick access to the cyclohepta­[*b*]­indole core **4g**. This privileged scaffold recurs in numerous natural products and bioactive molecules, and its direct formation from the parent ketone in a single operation illustrates the practical utility of this new reagent for medicinal chemistry and natural product synthesis.^26,27^ The method also proved compatible with various heterocyclic substrates, providing seamless access to more complex architectures (**4i–4k**) including indole **4l**, which contains an acid-labile acetal that would pose a significant hurdle under classical, strongly acidic Fischer conditions. Linear frameworks are also compatible (Table 2B), although enolates derived from alkyl aryl ketones furnished the corresponding indoles in only fair to modest yields (**4m–4o**, 46–63%). On the other hand, indolization of arylacetophenones proceeded well (**4p–4r**, 72–94%), and even acylated heterocycles were suitable substrates (**4s–4u**, 54–73%). Treatment of the enolates of α-ketoesters **3v** and **3z** delivered the synthetically valuable indole-2-carboxylate motifs in good to modest yields (**4v**, 67%; **4w**, 37%).

**Table 2:**
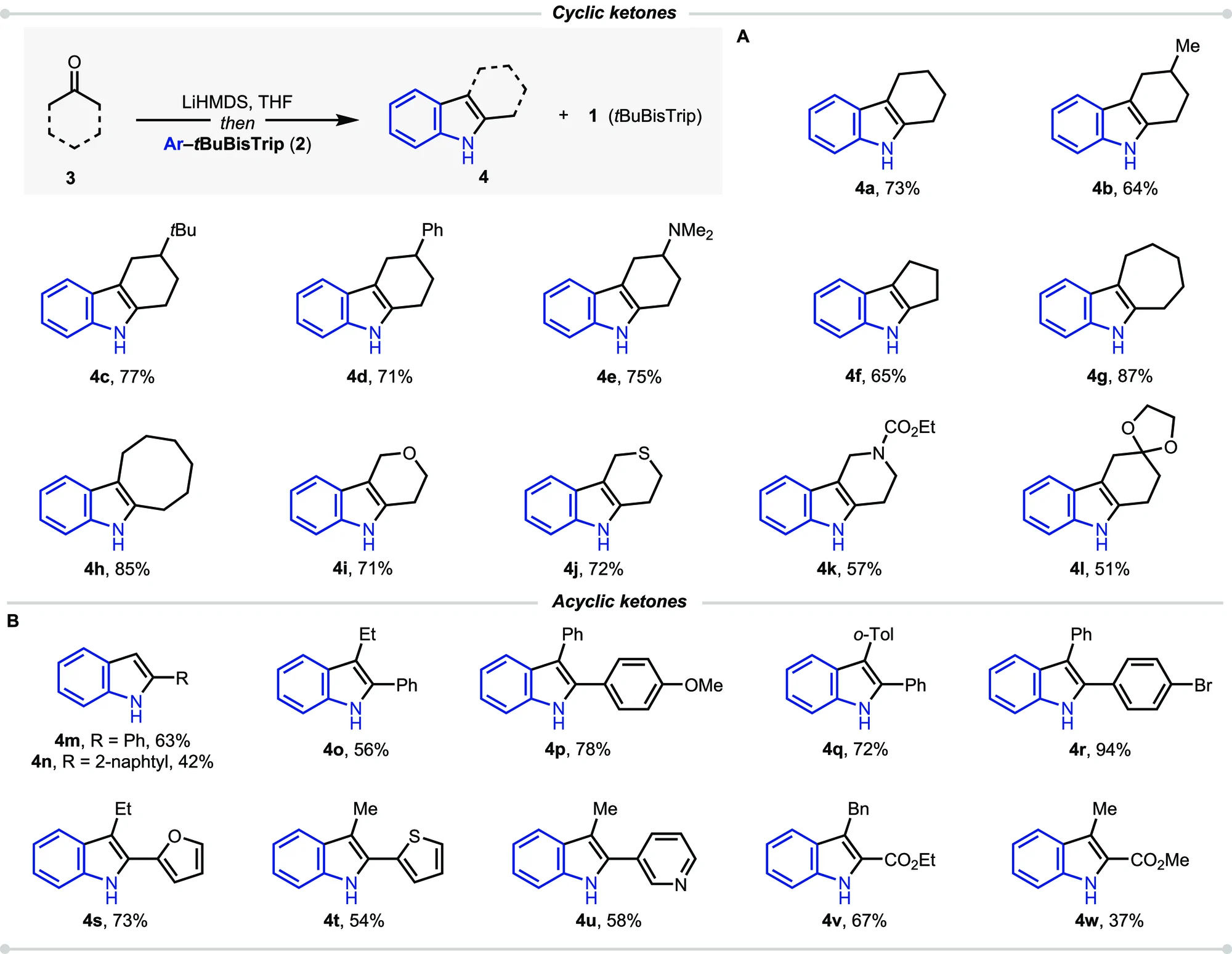
Substrate Scope of the Aryl-*t*BuBisTrip-Enabled One-Step Indole Synthesis^a^

a Reaction conditions (A, B): carbonyl compound (0.1 mmol), LiHMDS (0.11 mmol), aryl-*t*BuBisTrip **2** (0.13 mmol), and THF (0.1 M).

### Additional Enolate Classes and Synthetic Applications

To interrogate the scope of this indole annulation method, we examined additional enolate types and precursors (Table 3). With β-ketoesters, given their higher acidity, indole formation can be effected using the weaker guanidine base BTMG (Barton’s base) rather than LiHMDS (Table 3A). Thus, 3-carboxylated indoles **6a** and **6b** were prepared in good yields (66% and 75%, respectively). The same base also converted cyclohexane-1,3-dione to the corresponding indole **6c**, albeit in lower yield (40%).

**Table 3:**
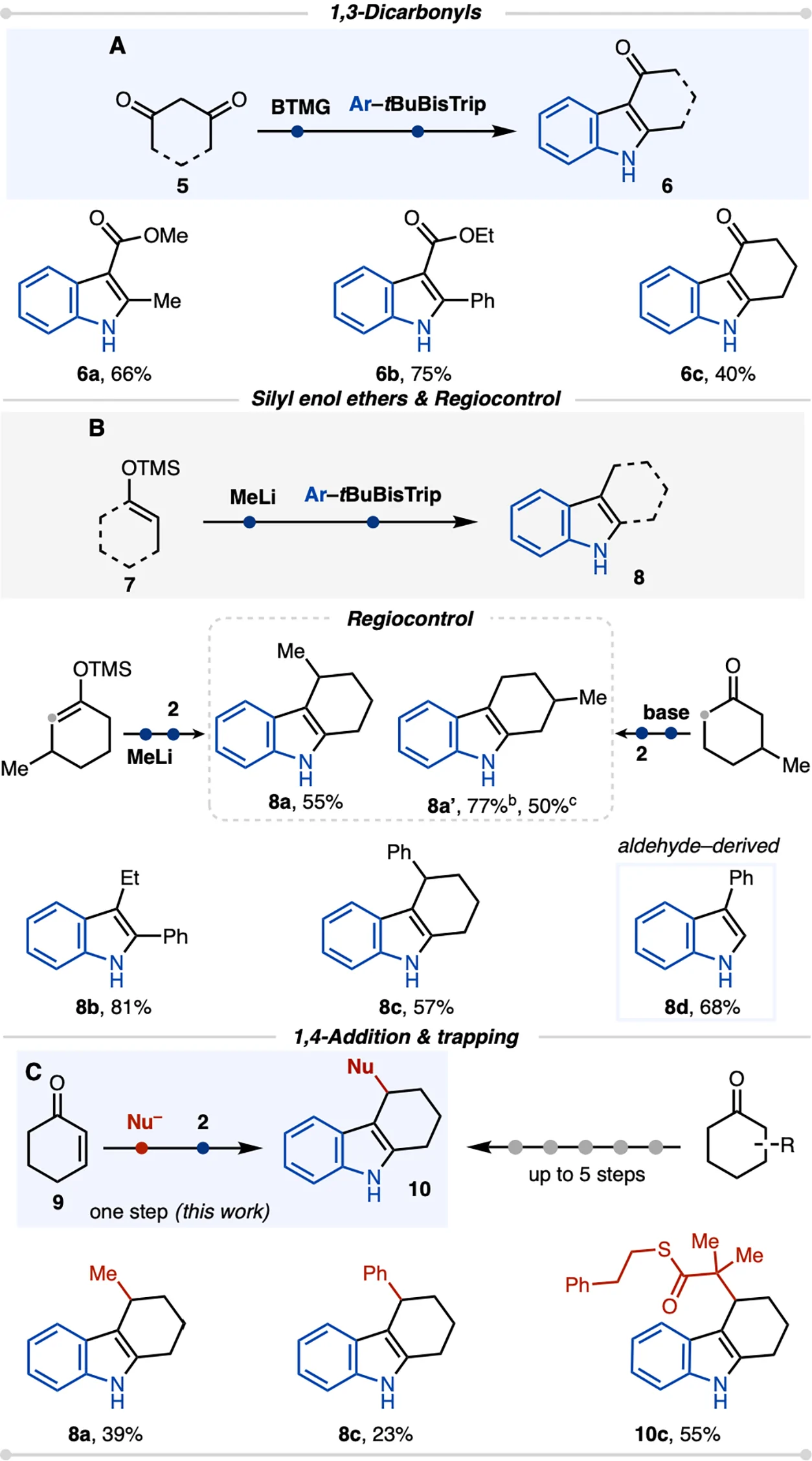
Scope of Ketone Derivative Substrates^a^

a Reaction conditions: (A) 1,3-Dicarbonyl compound (0.1 mmol), BTMG (0.11 mmol), aryl-*t*BuBisTrip **2** (0.13 mmol), THF (0.1 M), 40 °C. (B) Silyl enol ether (0.1 mmol), MeLi (0.11 mmol), **2** (0.13 mmol), THF (0.1 M), RT. (C) Cyclohexenone (0.1 mmol), nucleophile (0.11 mmol), **2** (0.13 mmol), THF (0.1 M).

b LDA was used as the base.

c NaHMDS was used as a base. Isolated yields were reported.

The versatility of the aryl-*t*BuBisTrip platform is further demonstrated by its reaction with enolates generated *in situ* from silyl enol ethers upon reaction with methyl lithium (Table 3B). This strategy provides complementary access to regioisomeric enolates that are challenging to prepare by conventional methods. For unsymmetrical substrates like 3-methylcyclohexanone, direct kinetic deprotonation channels reactivity toward indole **8a**′, whereas the isomeric TMS enol ether, which can be prepared regiospecifically, unlocks the complementary regioisomer **8a**.^28^ Coupling with aryl-tBuBisTrip **2** thus enables regiocontrolled formation of both **8a** and **8a**′ from related ketone precursors. By contrast, existing protocols not only deliver mixtures of regioisomers but also fundamentally lack any operational mechanism for tuning or reversing this selectivity^9b,11c,13a^ Beyond complementing direct kinetic deprotonation, the use of silyl enol ethers provides a clean alternative to direct deprotonation. This was demonstrated by the efficient formation of indoles **8b** (81% yield) and **8c** (57% yield). Importantly, silyl enol ethers grant facile entry into challenging, aldehyde-derived indoles (**8d**), avoiding the competitive self-condensation pathways typical of aldehydes under strongly basic conditions.

**Table 4:**
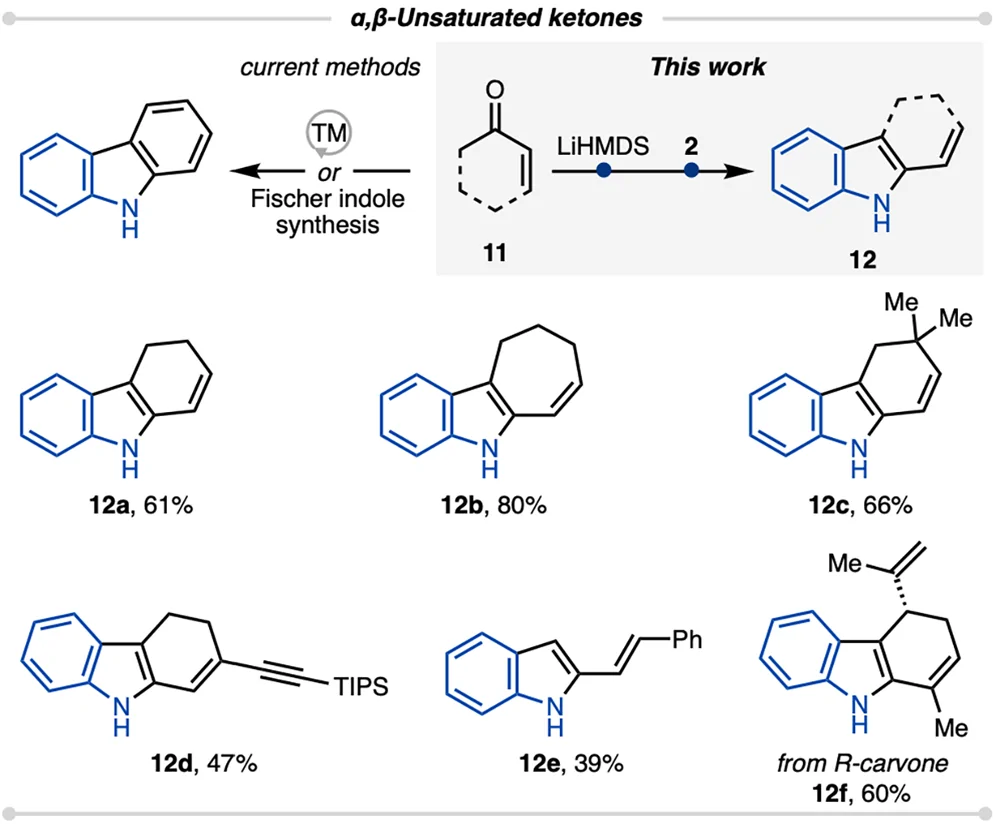
Scope of α,β-Unsaturated Ketones^a^

a Reaction conditions: carbonyl compound (0.1 mmol), LiHMDS (0.11 mmol), aryl-*t*BuBisTrip **2** (0.13 mmol), and THF (0.1 M). Isolated yields were reported.

The effectiveness of the new method suggested the potential for incorporating it in tandem sequences (Table 3C). Notably, 4-substituted tetrahydrocarbazoles (**10**) represent uniquely challenging synthetic targets because existing methods typically rely on lengthy pathways of up to five steps.^11c,18,29^ We envisioned that metal enolates, generated *in situ* via the conjugate addition of organometallic nucleophiles to α,β-unsaturated ketones, could be intercepted by the aryl-*t*BuBisTrip reagent **2** to streamline the assembly of these frameworks. Accordingly, the tandem addition of Ph_2_CuLi or Me_2_CuLi to 2-cyclohexen-1-one, followed by immediate treatment with **2**, gave rise to tetrahydrocarbazoles **8a** and **8c** (23% and 39% yield, respectively). Expanding upon this concept, we demonstrated that the complex enolate generated via the 1,4-addition of a thioester enolate to an enone could also be intercepted by **2**, yielding the functionalized indole **10c** in 55% yield.

Of special interest are α,β-unsaturated ketones, whose direct conversion into indole-annulated derivatives like **12a** (Table 4) remains challenging using established methods. Under classical Fischer conditions, for example, the reaction suffers from premature aromatization of the cyclohexenone-derived ring rather than yielding the desired fused compound.^30^ The employment of Pd-catalysis is also problematic, and the products are typically obtained as a 1:1 mixture with their aromatized counterparts.^11c^ In contrast, the current protocol accomplishes these transformations with good efficacy, providing an array of enone-derived indoles **12b–12e** in modest to good yields (39–80%), without formation of aromatized byproducts. The unique reactivity of aryl-*t*BuBisTrip **2** enabled the direct installation of the indole unit onto (*R*)-carvone, affording the unreported indole **12f** in 60% yield. By contrast, the corresponding Fischer indole synthesis is reported not to progress beyond the hydrazone intermediate.^30^

Given the frequent occurrence of indoles in drug discovery, we selected several scaffolds of pharmaceutical interest to demonstrate the utility of the BisTrip platform (Table 5). We first turned our attention to the H_1_ receptor antagonist ebastine, treatment of the enolate of which afforded indole **13** in 53% yield, with the remaining mass balance accounted for by recovered starting material. We also subjected the enone-containing natural product nootkatone to the aryl-*t*BuBisTrip reagent, which cleanly produced the novel derivative **14** in 73% yield. We examined the method on two intricate steroidal compounds, as the strategic installation of indole motifs onto these scaffolds is known to enhance bioactivity and modulate key physicochemical properties, including lipophilicity.^31,32^ In particular, subjecting cholestenone and a derivative of 18β-glycyrrhetinic acid to the arylation/annulation conditions furnished compounds **15** and **16** in 51% and 70% yield, respectively.^33^ To highlight the late-stage functionalization capability of this platform, the complex alkoxy-enone core of griseofulvin was successfully converted into indole **17** in 25% yield, with the rest of the mass balance consisting of unreacted starting material. Interestingly, subjecting the same substrate to standard Fischer indole conditions resulted in no product formation, with only 57% of griseofulvin recovered.

**Table 5:**
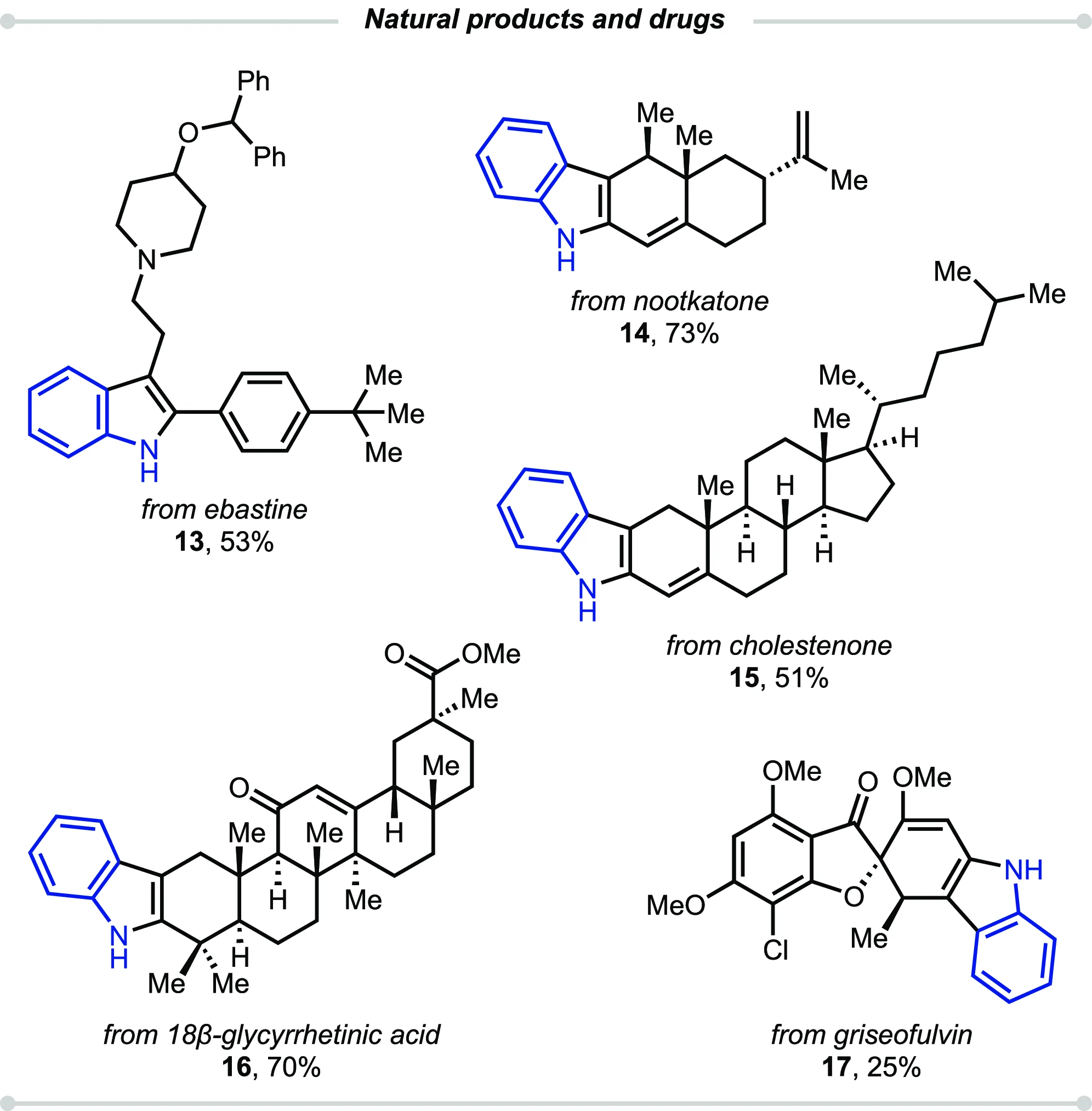
Indole Synthesis with Complex Substrates, Natural Products, and Drugs^a^

a Reaction conditions: carbonyl compound (0.1 mmol), LiHMDS (0.11 mmol), **2** (0.13 mmol), and THF (0.1 M). Isolated yields were reported.

The demonstrated capability of the *t*BuBisTrip platform to engage directly with lithium enolates suggested that asymmetric deprotonation strategies could be translated directly into chiral indole synthesis. To evaluate this concept, we targeted the desymmetrization of a prochiral 4-substituted cyclohexanone and its conversion to the corresponding indole. Enantioselective deprotonation of 4-*tert*-butylcyclohexanone with Simpkins’ base (Figure 3, **18**) followed by silylation afforded the corresponding chiral silyl enol ether **3c**′.^34^ Treatment of compound **3c**′ with MeLi, followed by the addition of reagent **2**, smoothly furnished the desymmetrized indole **4c**′ in 70% yield with a 90% enantiomeric excess, slightly higher than the reported value for the parent enol ether.^34^ The high ee of **4c**′ indicates that enolate generation and the subsequent C–C bond-forming event outcompete proton transfers that could either quench the enolate or scramble its regiochemistry, which would erode the enantiopurity of the final product.

**Figure 3:**
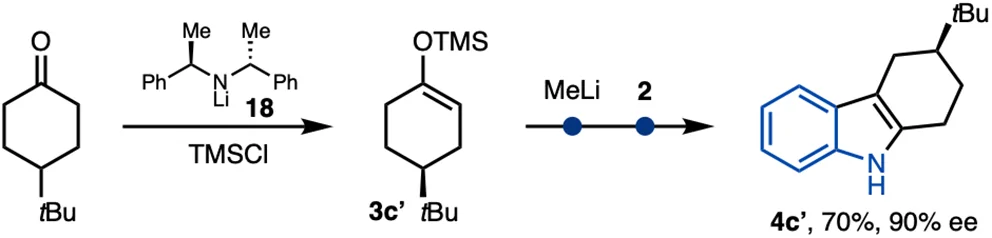
Desymmetrization of 4-substituted ketones with Simpkins’ base.

### Mechanism

Consistent with previous reports on Bi­(V)-mediated arylation of enolates from our laboratory and others,^18,19,35–37^ the proposed mechanism commences with the coordination of enolate **A** to the aryl-*t*BuBisTrip reagent **2**, generating the pentacoordinate bismuth­(V) intermediate **B** (Figure 4). Subsequent reductive ligand coupling restores *t*BuBisTrip **1** and forms the α-arylated species **C**. This intermediate undergoes spontaneous cyclization with the concomitant loss of water to furnish the indole product **D**. Attempts to isolate or observe intermediate **C** were unsuccessful.

**Figure 4:**
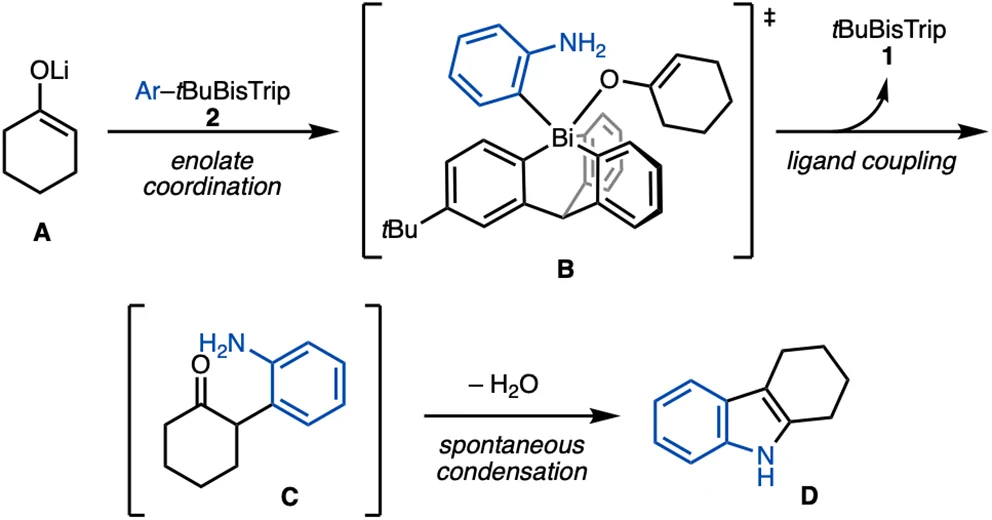
Mechanism of the aryl-BisTrip enabled arylation/annulation reaction.

## Conclusions

We have developed a robust, one-step method for the synthesis of indoles from diverse carbonyl precursors using a unique bismuth­(V) platform. Specifically, 2-aminophenyl-*t*BuBisTrip (**2**) effects α-arylation of lithium enolates followed by spontaneous annulation to furnish indoles with high regiocontrol. The method is effective for a broad range of substrates, including cyclic, acyclic, and heterocyclic ketones, as well as 1,2- and 1,3-dicarbonyl compounds. Particularly noteworthy is the direct indole annulation of enones, a class of ketones that has eluded established Fischer and transition-metal-catalyzed methods. The transformation also supports late-stage functionalization of complex natural product and pharmaceutical scaffolds, underscoring its translational potential in medicinal chemistry. Together, these results establish that the *t*BuBisTrip platform enables a regiocontrolled main-group strategy for indole construction from simple carbonyl precursors, including frameworks that remain challenging by conventional methods.

## Supplementary Material



## Notes

Experimental procedures and characterization data for all new compounds (PDF)
