# Supplementary material for: Regiocontrolled One-Step Indole Synthesis from Carbonyl Compounds via Organobismuth Chemistry
Source: J Am Chem Soc. 2026 Oct 19;148(43):47170–8. doi: 10.1021/jacs.6c15371 (PMC13741823; doi:10.1021/jacs.6c15371)
Supplement: Supplementary file 1 [file ja6c15371_si_001.pdf]

Supporting Information

Regiocontrolled One-Step Indole Synthesis from Carbonyl Compounds via Organobismuth Chemistry

Filippo Carpaneto[1], Li Li[1], Viresh H. Rawal[1]

[1]Department of Chemistry, University of Chicago, Chicago, Illinois 60637, United States

Corresponding author. Email: vrawal@uchicago.edu

Table of contents

1. Materials and Methods S2
2. Table S1. Optimization of the Synthesis of the Aryl-*t*BuBisTrip Reagent S3
3. Synthesis of *t*BuBisTrip and Aryl-*t*BuBisTrip Reagent. S4
4. General Procedures and Substrate Scope S7
5. NMR Spectra S38
6. HPLC Chromatograms S84
7. References S85

1. Materials and Methods

Commercially obtained reagents were used as received, without further purification. Unless stated otherwise, reactions were performed under a nitrogen atmosphere with the use of Schlenk line using oven- or flame-dried glassware and stir bars. Ambient temperature refers to 22–24 °C. Pre-heated oil baths were used to maintain temperatures above ambient. Lower temperatures were maintained using liquid N_2/EtOH (-110 °C), acetone/CO_2(s) (−78 °C), acetonitrile/CO_2(s) (−40 °C), and brine/ice (-10 °C) baths. Tetrahydrofuran (THF) was dried by passage through an activated alumina column purification system (Innovative Technology Inc. Pure Solv TM).

Thin-layer chromatography (TLC) was performed using EMD Millipore silica gel 60 Å plates with UV fluorescence quenching (254 nm) or $KMnO_4$ stain. Flash column chromatography was performed on SiliCycle SiliaFlash P60 (40-63 µm particle size) using ACS or HPLC grade solvents purchased from Fisher Scientific.

^{1}H NMR spectra were recorded on Bruker 500 and Bruker 600 spectrometers (at 500 MHz and 600 MHz, respectively) at 294-297 °K. ^{13}C NMR spectra were recorded on Bruker 600, Bruker 500, and Bruker 400 spectrometers (at 150 MHz, 125 MHz, and 100 MHz respectively) at 294-297 K. ^{1}H spectra were calibrated from internal standard TMS (δ 0.0). ^{13}C spectra were calibrated from solvent resonance ($CDCl_3$: δ 77.16). NMR data are reported as: chemical shift (δ ppm) (multiplicity, coupling constant (Hz), and integration).

Chiral high-performance liquid chromatography (HPLC) analysis was performed using an Agilent analytical chromatography system with commercial Chiralcel® columns equipped with a guard column. High-resolution mass spectral (HRMS) analyses were performed on Agilent Technologies 6224 TOF LC/MS using electrospray ionization (ESI) at the University of Chicago Mass Spectroscopy Core Facility.

2. Table S1. Optimization of the Synthesis of the Aryl-*t*BuBisTrip Reagent[a]

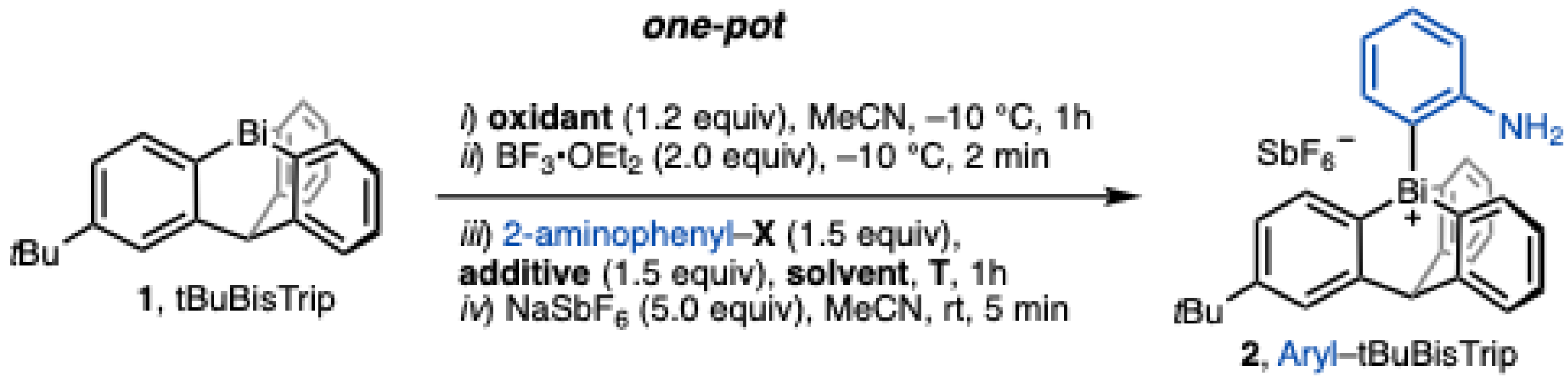

entry	oxidant	-X	Additive	solvent	T (°C)	yield of **2** (%)[c]
1	Selectfluor	$–B(OH)_2$	–	–[b]	–35-rt	35
2	Selectfluor	$–BF_3K$	–	–[b]	–35-rt	trace
3	Selectfluor	$–B(OH)_2$	$BF_3•OEt_2$	MeCN	–35-rt	57
4	Selectfluor	$–BF_3K$	$BF_3•OEt_2$	MeCN	–35-rt	trace
5	Selectfluor	–B(pin)	–	MeCN	–35-rt	0
6	Selectfluor	–B(pin)	$BF_3•OEt_2$	MeCN	–35-rt	0
7	Selectfluor	$–B(OH)_2$	$BF_3•OEt_2$	MeCN	–10-rt	15
8	Selectfluor	$–B(OH)_2$	$BF_3•OEt_2$	MeCN/THF 1:1	–50-rt	51
9	NBS	$–B(OH)_2$	$BF_3•OEt_2$	MeCN	–35-rt	0
10	Br_2	$–B(OH)_2$	$BF_3•OEt_2$	MeCN	–35-rt	0

[a]All reactions ran on 0.1 mmol scale. [b]Added as a solid. [c]Isolated yields

3. Synthesis of *t*BuBisTrip and Aryl-*t*BuBisTrip Reagent.

3.1 3-*tert*-Butyl-9-bismatriptycene (*t*BuBisTrip) 1 synthesis.

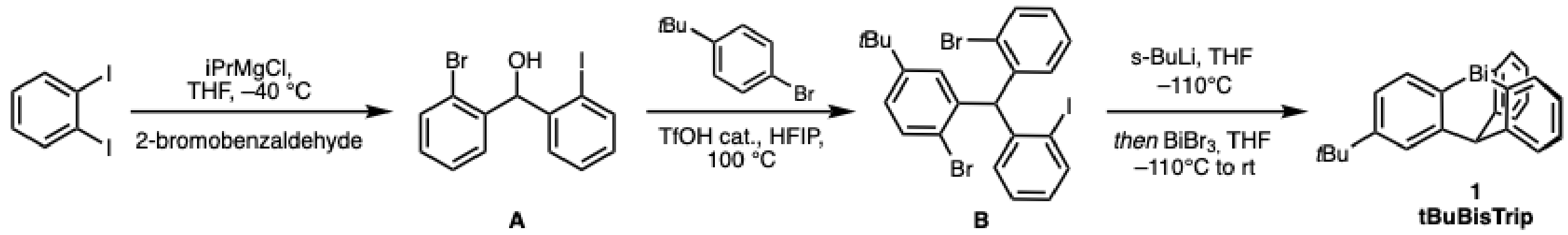

(2-Bromophenyl)(2-iodophenyl)methanol (A)

A was prepared following a modified procedure reported by Berionni [1]. A dry 500 mL flask equipped with a stir bar and maintained under nitrogen was charged with 1,2-diiodobenzene (66.0 g, 0.21 mol, 1.0 equiv) in dry THF (200.0 mL) and cooled to −40 °C. A solution of *i*PrMgCl (2.0 M in THF, 110.0 mL, 0.22 mol, 1.1 equiv) was added dropwise over 10 minutes. The mixture was stirred at −40 °C for 1 hour, followed by dropwise addition of 2-bromobenzaldehyde (37.0 g, 0.20 mol, 1.0 equiv) over 10 minutes. After stirring at −40 °C for an additional 30 minutes, the reaction was warmed to room temperature, left stirring for 4h and quenched with saturated aqueous NH_4Cl (50.0 mL). The mixture was extracted with ethyl acetate (3 × 200.0 mL), and the combined organic layers were dried over anhydrous $MgSO_4$, filtered, and concentrated in vacuo. The crude residue was purified by column chromatography, eluting with hexane:ethyl acetate (85:15) to afford product **A** as a white solid (97% yield, 75.3 g). The compound was fully consistent with literature data.

1-bromo-2-((2-bromophenyl)(2-iodophenyl)methyl)-4-(*tert*-butyl)benzene (B)

B was prepared following a modified procedure reported by Berionni [1]. A dry sealed tube equipped with a stir bar was charged with (2-bromophenyl)(2-iodophenyl)methanol **A** (17.0 g, 43.7 mmol, 1.0 equiv), 1-bromo-4-tert-butylbenzene (30.3 mL, 174.8 mmol, 4.0 equiv), and HFIP (70.0 mL). Neat triflic acid (0.39 mL, 4.37 mmol, 0.10 equiv) was added rapidly to the solution, and the tube was immediately sealed. The mixture was heated at 100 °C for 24 hours. After cooling to room temperature, the reaction mixture was concentrated in vacuo. The crude residue was purified by column chromatography (hexane:DCM = 90:10, Rf = 0.5) to afford product **B** as a solid (80% yield, 20.4 g).

^{1}H NMR (500 MHz, $CDCl_3$) δ 7.91 (dd, *J* = 7.9, 1.3 Hz, 1H), 7.62 (dd, *J* = 7.9, 1.4 Hz, 1H), 7.51 (d, *J* = 8.3 Hz, 1H), 7.25 (td, *J* = 7.3, 1.1 Hz, 1H), 7.22 (td, *J* = 7.5, 1.2 Hz, 1H), 7.18 – 7.12 (m, 2H), 6.97 (td, *J* = 7.6, 1.7 Hz, 1H), 6.73 – 6.67 (m, 3H), 6.17 (s, 1H), 1.14 (s, 9H).

^{13}C NMR (126 MHz, $CDCl_3$) δ 150.52, 144.31, 141.30, 140.40, 140.28, 133.40, 132.74, 131.04, 130.51, 128.60, 128.52, 128.47, 128.15, 127.36, 126.56, 125.52, 123.18, 103.24, 61.20, 34.61, 31.21.

HRMS (ESI) calculated for $C_{23}H_{21}Br_2I$ M^+ m/z: 581.9055, found: 581.8709

3-*tert*-Butyl-9-bismatriptycene (*t*BuBisTrip) (1)

Under a nitrogen atmosphere, a solution of *sec*-butyllithium (64 mL, 1.4 M in pentane, 120.0 mmol, 6.0 equiv) was added dropwise to a solution of 1-bromo-2-((2-bromophenyl)(2-iodophenyl)methyl)-4-(tert-butyl)benzene **B** (8.76 g, 15.0 mmol, 1.0 equiv) in dry THF (150.0 mL) at –110 °C. After stirring at –110 °C for 1 hour, a THF solution (30.0 mL) of $BiBr_3$ (6.73 g, 15.0 mmol, 1.0 equiv) was added dropwise under nitrogen. Upon completion of the addition, the reaction mixture was allowed to warm to room temperature over 1 hour. The reaction was then quenched with NH_4Cl (75 mL) and extracted with DCM (3 × 150 mL). The combined organic layers were dried over anhydrous Na_2SO_4, concentrated under reduced pressure, and purified by silica gel column chromatography (hexane:DCM = 90:10, Rf = 0.3) to afford the desired product **1** (5.51 g, 73%).[2]

^{1}H NMR (500 MHz, $CDCl_3$) δ 8.08 (dd, *J* = 7.1, 1.3 Hz, 2H), 8.01 (d, *J* = 7.6 Hz, 1H), 7.99 – 7.96 (m, 3H), 7.21(td, *J* = 7.2, 1.3 Hz, 2H), 7.08 (dd, *J* = 7.6, 2.1 Hz, 1H), 7.03 (td, *J* = 7.3, 1.3 Hz, 2H), 6.00 (s, 1H), 1.30 (s, 9H).

^{13}C NMR (126 MHz, $CDCl_3$) δ 151.7, 147.5, 146.9, 136.1, 135.7, 128.6, 128.1, 126.3, 125.2, 123.6, 86.0, 34.8, 31.6.

HRMS (ESI) calculated for $[C_{23}H_{21}Bi]^+$ m/z: 506.1447, found: 506.1444

3.2 9-(2-Aminophenyl)- 3-*tert*-butyl-9-bismatriptycene hexafluoroantimonate (2).

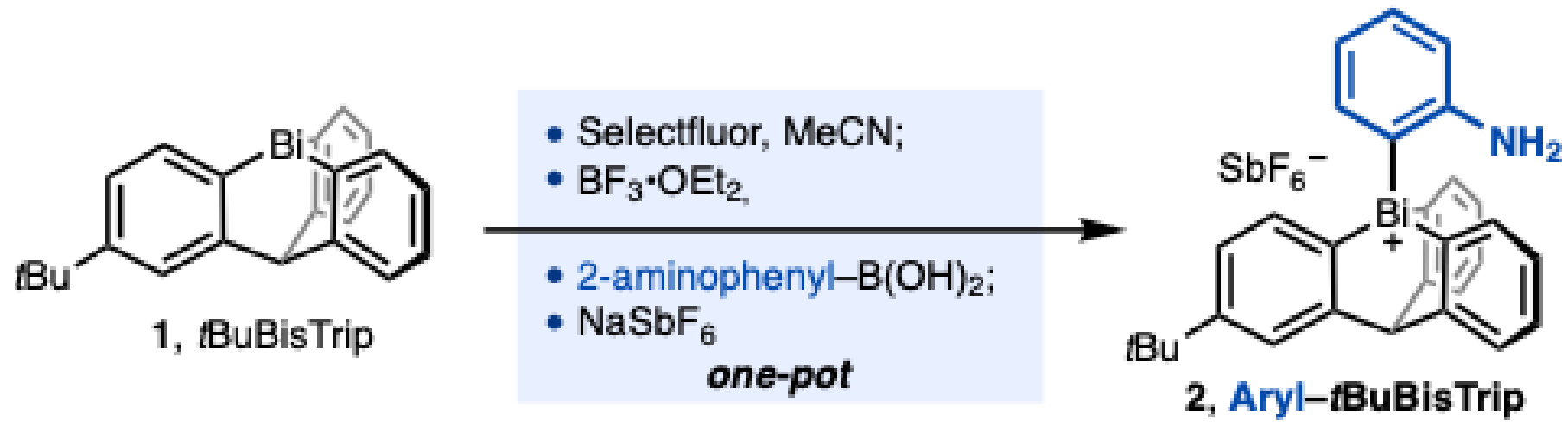

A round-bottomed flask was charged with *t*BuBisTrip **1** (253 mg, 0.50 mmol, 1.0 equiv) and MeCN (25 mL). The reaction mixture was cooled to −10 °C under open air, and Selectfluor® (213 mg, 0.65 mmol, 1.2 equiv) was added. After stirring at −10 °C for 1 h, $BF_3 \cdot OEt_2$ (0.16 mL, 1.0 mmol, 2.0 equiv) was added dropwise, after 30 seconds the mixture was further cooled to −35 °C. 2-Aminophenylboronic acid (103 mg, 0.75 mmol, 1.5 equiv) was dissolved in MeCN (5.0 mL) with $BF_3 \cdot OEt_2$ (0.12 mL, 0.75 mmol, 1.5 equiv) and then added dropwise over 5 minutes. The reaction mixture was allowed to warm to room temperature over 1 h. Subsequently, a solution of $NaSbF_6$ (647 mg, 2.50 mmol, 5.0 equiv) in MeCN (5.0 mL) was added. After stirring at room temperature for 5 min, the reaction mixture was concentrated in vacuo, and the crude product was purified by flash column chromatography (DCM:iPrOH = 100:0 to 95:5, Rf = 0.8) afforded 229 mg (57%) of **2** as a brick-red solid.

Note: On a 3.0 mmol scale, the reaction afforded 1.52 g of **2** (61% yield).

^{1}H NMR (500 MHz, $CDCl_3$) δ 8.00 – 7.92 (m, 6H), 7.79 (d, *J* = 8.1 Hz, 1H), 7.60 (dt, *J* = 13.8, 7.7 Hz, 2H), 7.47 (t, *J* = 7.4 Hz, 1H), 7.38 (dd, *J* = 8.1, 2.1 Hz, 1H), 7.35 – 7.28 (m, 4H), 5.92 (s, 1H), 4.65 (s, 2H), 1.29 (s, 9H).

^{13}C NMR (126 MHz, $CDCl_3$) δ 155.4, 146.5, 144.7, 144.6, 144.3, 141.3, 135.2, 134.6, 134.3, 133.9, 133.5, 131.4, 130.8, 129.2, 127.7, 127.4, 127.0, 126.5, 65.4, 35.3, 31.3.

^{19}F NMR (470 MHz, Acetone-*d*$_6$) δ -112.93 – -133.53 (sextet, *J* = 1939 Hz, $^{1}J(^{121}Sb, ^{19}F)$), -115.41 – -131.05 (octet, J = 1050 Hz, $^{1}J(^{123}Sb, ^{19}F)$).

HRMS (ESI) calculated for $[C_{29}H_{27}BiN]^+$ m/z: 598.1942, found: 598.1963

4. General Procedures and Substrate Scope

4.1. General Procedures

General Procedure A

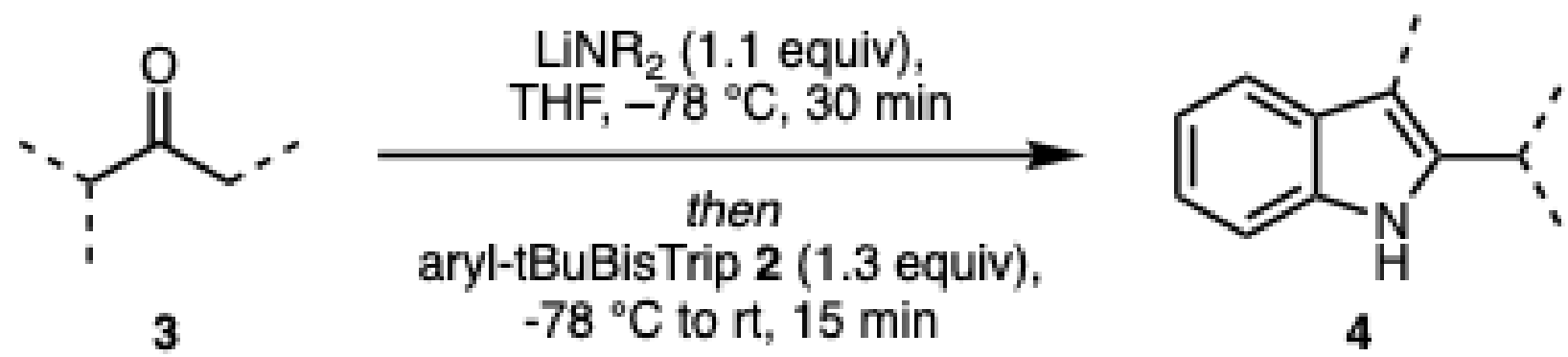

A dry 5 mL screw-cap vial equipped with a stir bar and maintained under nitrogen was charged with substrate **3** (0.10 mmol, 1.0 equiv) and dry THF (1.0 mL). The solution was cooled to –78 °C, and a solution of LiHMDS (1.0 M THF, 0.11 mmol, 1.1 equiv) or LDA (1.0 M THF/hexanes, 0.11 mmol, 1.1 equiv) was added dropwise. After stirring for 30 minutes, aryl-tBuBisTrip **2** (0.13 mmol, 1.3 equiv) was added in one portion as a solid, and the resulting mixture was stirred at –78 °C for 2 minutes. The reaction was then allowed to warm to room temperature and stirred for an additional 15 min before being quenched with H_2O (0.5 mL). The mixture was extracted with DCM (3 × 5.0 mL), and the combined organic phases were dried over anhydrous Na_2SO_4, filtered, and concentrated in vacuo. The resulting crude was purified via column chromatography, eluting first with hexane:DCM (4:1) to recover tBuBisTrip (**1**) (91% to quantitative) followed by an appropriate eluent to afford the final product **4**.

General Procedure B

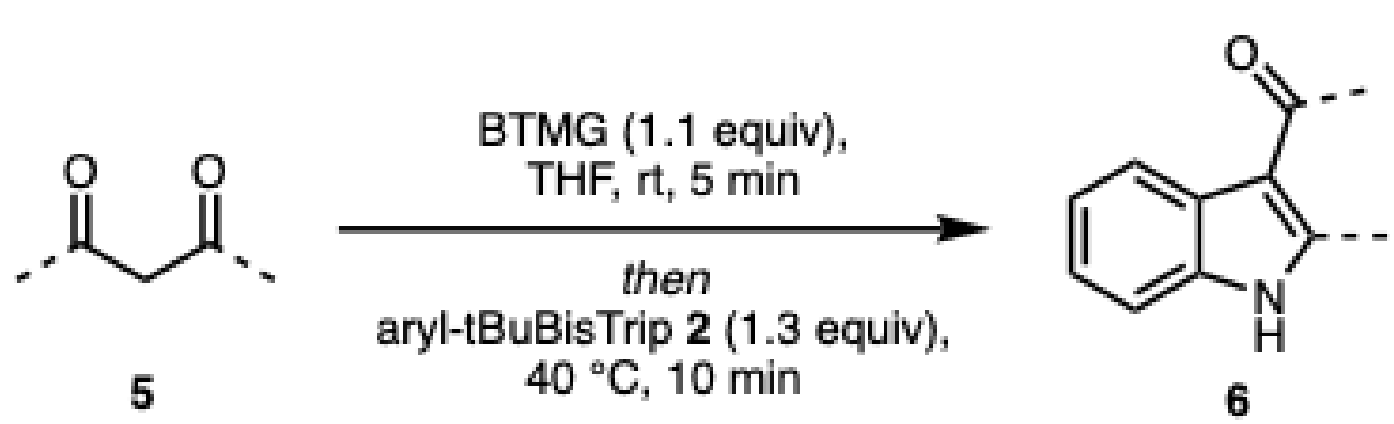

A dry 5 mL screw-cap vial equipped with a stir bar and maintained under nitrogen was charged with 1,3-dicarbonyl compound **5** (0.10 mmol, 1.0 equiv) and dry THF (1.0 mL). BTMG (22 µL, 0.11 mmol, 1.1 equiv) was added to the solution at room temperature. After stirring for 5 min, the reaction was heated to 40 °C, and aryl-tBuBisTrip **2** (0.13 mmol, 1.3 equiv) was added in one portion as a solid. The resulting mixture was stirred for 10 minutes, then allowed to cool down to room temperature before being quenched with H_2O (0.5 mL). The mixture was extracted with

DCM (3 × 5.0 mL), and the combined organic phases were dried over anhydrous Na_2SO_4, filtered, and concentrated in vacuo. The resulting crude was purified via column chromatography, eluting first with hexane:DCM (4:1) to recover tBuBisTrip (**1**) (95% to quantitative) followed by an appropriate eluent to afford the final product **6**.

General Procedure C

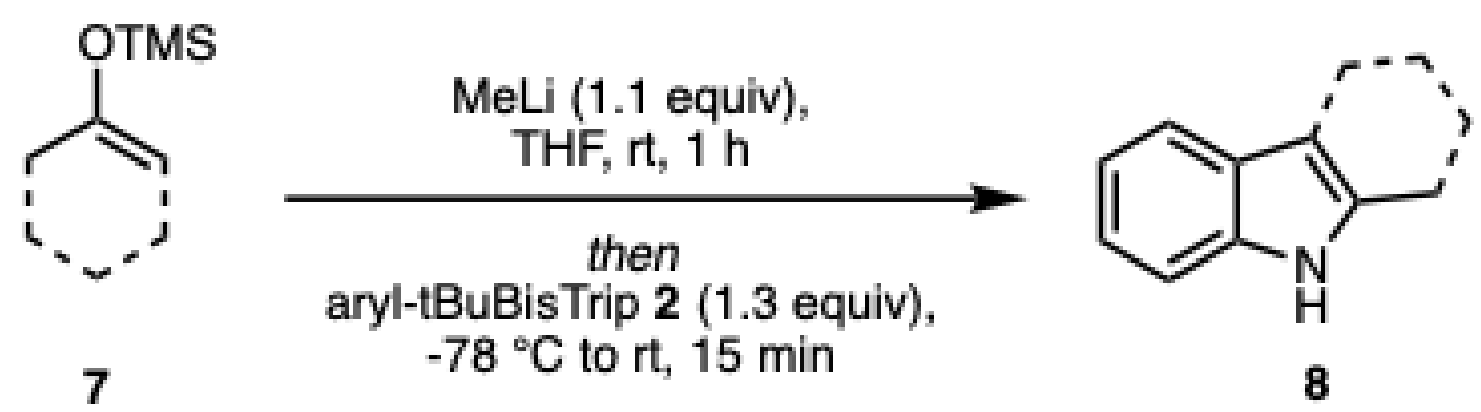

A dry 5 mL screw-cap vial equipped with a stir bar and maintained under nitrogen was charged with silyl enol ether **7** (0.10 mmol, 1.0 equiv) and dry THF (1.0 mL). MeLi (70 µL, 1.6 M Et_2O, 0.11 mmol, 1.1 equiv) was added dropwise to the solution at room temperature. After stirring for 1 h, the reaction was cooled to –78 °C, and aryl-tBuBisTrip **2** (0.13 mmol, 1.3 equiv) was added in one portion as a solid. The resulting mixture was stirred at –78 °C for 2 minutes, then allowed to warm to room temperature and stirred for an additional 15 min before being quenched with H_2O (0.5 mL). The mixture was extracted with DCM (3 × 5.0 mL), and the combined organic phases were dried over anhydrous Na_2SO_4, filtered, and concentrated in vacuo. The resulting crude was purified via column chromatography, eluting first with hexane:DCM (4:1) to recover tBuBisTrip (**1**) (97% to quantitative) followed by an appropriate eluent to afford the final product **8**.

General Procedure D

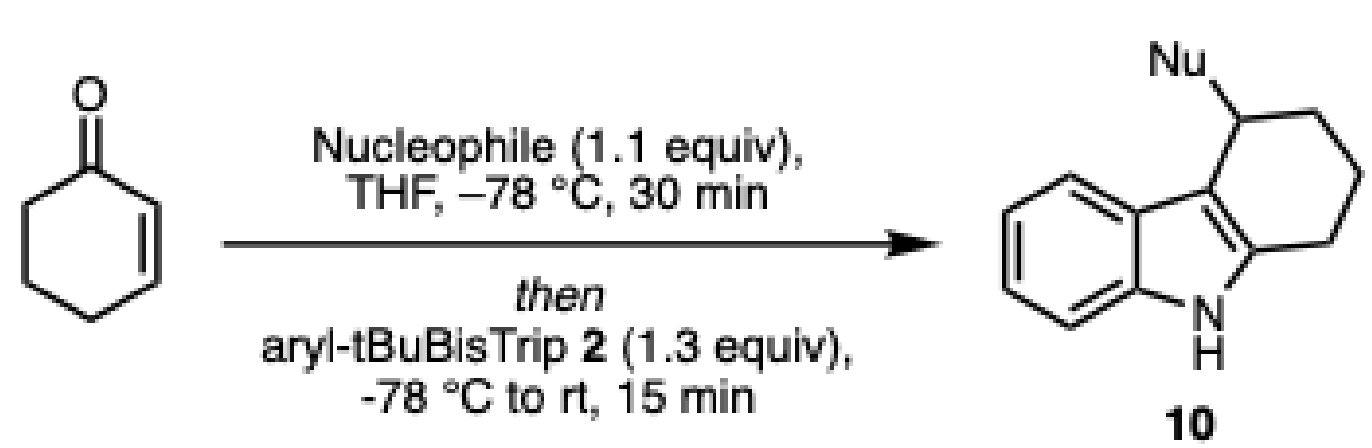

A dry 5 mL screw-cap vial equipped with a stir bar and maintained under nitrogen was charged with 2-cyclohexen-1-one (9.6 mg, 0.10 mmol, 1.0 equiv) and dry THF (1.0 mL). The solution was cooled to –78 °C, and a solution of nucleophile (0.11 mmol, 1.1 equiv) was added dropwise. After

stirring for 30 minutes, aryl-tBuBisTrip **2** (0.13 mmol, 1.3 equiv) was added in one portion as a solid, and the resulting mixture was stirred at –78 °C for 2 minutes. The reaction was then allowed to warm to room temperature and stirred for an additional 15 min before being quenched with H_2O (0.5 mL). The mixture was extracted with DCM (3 × 5.0 mL), and the combined organic phases were dried over anhydrous Na_2SO_4, filtered, and concentrated in vacuo. The resulting crude was purified via column chromatography, eluting first with hexane:DCM (4:1) to recover tBuBisTrip (**1**) (85% to quantitative) followed by an appropriate eluent to afford the final product **10**.

4.2. Substrate Scope

2,3,4,9-tetrahydro-1*H*-carbazole (4a)

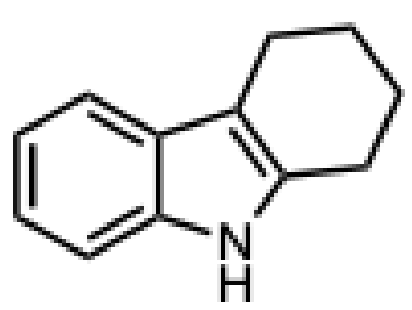

The compound was synthesized according to the **general procedure A**, employing cyclohexanone (9.8 mg, 0.10 mmol, 1.0 equiv), LiHMDS (0.11 mL, 1.0 M THF, 0.11 mmol, 1.1 equiv), and aryl-tBuBisTrip **2** (108 mg, 0.13 mmol, 1.3 equiv). Purification by flash column chromatography (hexane:DCM = 4:1, followed by hexane:ethyl acetate = 95:5) afforded 12.4 mg (73%) of **4a** as a yellow solid. The compound was fully consistent with literature data [3].

^{1}H NMR (600 MHz, $CDCl_3$) δ 7.66 (s, 1H), 7.46 (d, *J* = 7.6 Hz, 1H), 7.28 (d, *J* = 7.9 Hz, 1H), 7.12 (td, *J* = 7.3, 1.2 Hz, 1H), 7.08 (td, *J* = 7.5, 1.2 Hz, 1H), 2.76 – 2.69 (m, 4H), 1.96 – 1.85 (m, 4H).

^{13}C NMR (150 MHz, $CDCl_3$) δ 35.8, 134.2, 128.0, 121.1, 119.2, 117.9, 110.5, 110.3, 23.4, 23.4, 23.4, 21.1.

HRMS (ESI) calculated for $[C_{12}H_{13}N+H]^+$ 172.1121, found:172.1121

3-methyl-2,3,4,9-tetrahydro-1*H*-carbazole (4b)

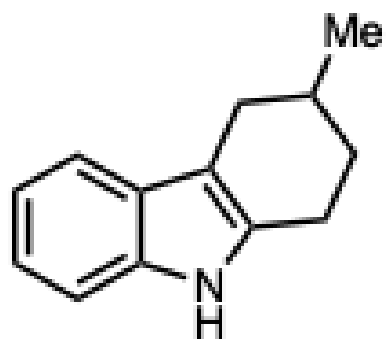

The compound was synthesized according to the **general procedure A**, employing 4-methylcyclohexan-1-one (12.1 mg, 0.10 mmol, 1.0 equiv), LiHMDS (0.11 mL, 1.0 M THF, 0.11 mmol, 1.1 equiv), and aryl-tBuBisTrip **2** (108 mg, 0.13 mmol, 1.3 equiv). Purification by flash column chromatography (hexane:DCM = 4:1, followed by hexane:ethyl acetate = 95:5, Rf = 0.4) afforded 11.8 mg (64%) of **4b** as a yellow solid. The compound was fully consistent with literature data [4].

^{1}H NMR (500 MHz, $CDCl_3$) δ 7.63 (s, 1H), 7.47 (d, *J* = 7.6 Hz, 1H), 7.27 (d, *J* = 7.8 Hz, 1H), 7.19 – 7.02 (m, 2H), 2.87 (dd, *J* = 15.3, 5.0 Hz, 1H), 2.76 (m, 2H), 2.30 (dd, *J* = 15.4, 9.5 Hz, 1H), 2.02 – 1.93 (m, 2H), 1.65 – 1.54 (m, 1H), 1.16 (d, *J* = 6.5 Hz, 3H).

^{13}C NMR (126 MHz, $CDCl_3$) δ 136.1, 134.0, 127.9, 121.1, 119.2, 117.8, 110.5, 110.3, 31.5, 29.8, 29.5, 23.0, 21.9.

HRMS (ESI) calculated for $[C_{13}H_{15}N+H]^+$ m/z: 186.1277, found:186.1271

3-(*tert*-butyl)-2,3,4,9-tetrahydro-1*H*-carbazole (4c)

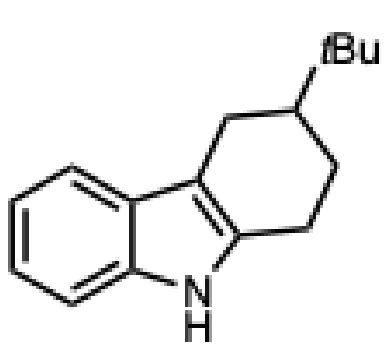

The compound was synthesized according to the **general procedure A**, employing 4-*tert*-butylcyclohexan-1-one (15.4 mg, 0.10 mmol, 1.0 equiv), LiHMDS (0.11 mL, 1.0 M THF, 0.11 mmol, 1.1 equiv), and aryl-tBuBisTrip **2** (108 mg, 0.13 mmol, 1.3 equiv). Purification by flash column chromatography (hexane:DCM = 4:1, followed by hexane:ethyl acetate = 95:5, Rf = 0.4)

afforded 17.1 mg (75%) of **4c** as a yellow solid. The compound was fully consistent with literature data [3].

^{1}H NMR (500 MHz, $CDCl_3$) δ 7.65 (s, 1H), 7.48 (dd, *J* = 7.5, 1.4 Hz, 1H), 7.28 (d, *J* = 1.0 Hz, 1H), 7.12 (td, *J* = 7.3, 1.4 Hz, 1H), 7.12 (td, *J* = 7.6, 1.4 Hz, 1H), 2.87 – 2.81 (m, 1H), 2.76 (tt, *J* = 8.3, 2.0 Hz, 2H), 2.46 – 2.37 (m, 1H), 2.15 – 2.07 (m, 1H), 1.60 – 1.50 (m, 3H), 1.01 (s, 9H).

^{13}C NMR (126 MHz, $CDCl_3$) δ 136.2, 134.3, 128.1, 121.1, 119.2, 117.8, 110.7, 110.5, 45.6, 32.8, 27.7, 24.9, 24.3, 22.4.

HRMS (ESI) calculated for $[C_{16}H_{21}N+H]^+$ m/z: 228.1747, found: 228.1744

3-phenyl-2,3,4,9-tetrahydro-1*H*-carbazole (4d)

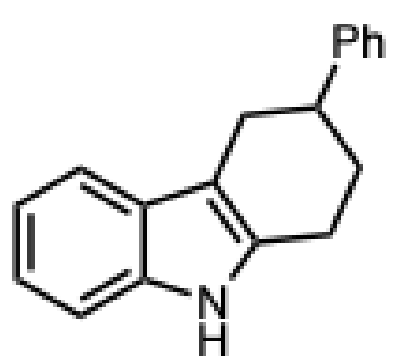

The compound was synthesized according to the **general procedure A**, employing 4-phenylcyclohexan-1-one (17.4 mg, 0.10 mmol, 1.0 equiv), LiHMDS (0.11 mL, 1.0 M THF, 0.11 mmol, 1.1 equiv), and aryl-tBuBisTrip **2** (108 mg, 0.13 mmol, 1.3 equiv). Purification by flash column chromatography (hexane:DCM = 4:1, followed by hexane:ethyl acetate = 95:5, Rf = 0.4) afforded 17.5 mg (71%) of **4d** as a yellow solid. The compound was fully consistent with literature data [5].

^{1}H NMR (500 MHz, $CDCl_3$) δ 7.70 (s, 1H), 7.50 (d, *J* = 7.7 Hz, 1H), 7.39 (m, 4H), 7.31 (m, 2H), 7.19 (t, *J* = 7.2 Hz, 1H), 7.14 (t, *J* = 7.4 Hz, 1H), 3.18 – 3.09 (m, 2H), 2.99 – 2.81 (m, 3H), 2.26 (m, 1H), 2.22 – 2.12 (m, 1H).

^{13}C NMR (126 MHz, $CDCl_3$) δ 146.8, 136.1, 133.8, 128.6, 127.7, 127.2, 126.4, 121.3, 119.4, 117.9, 110.6, 110.2, 41.3, 30.5, 29.4, 23.5.

HRMS (ESI) calculated for $[C_{18}H_{17}N+H]^+$ m/z: 248.1434, found: 248.1435

_N_,_N_-dimethyl-2,3,4,9-tetrahydro-1_H_-carbazol-3-amine (4e)

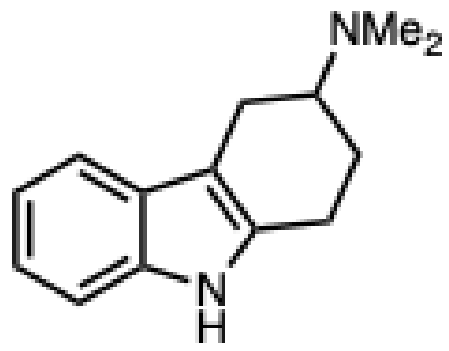

The compound was synthesized according to the **general procedure A**, employing 4-(dimethylamino)cyclohexan-1-one (14.1 mg, 0.10 mmol, 1.0 equiv), LiHMDS (0.11 mL, 1.0 M THF, 0.11 mmol, 1.1 equiv), and aryl-tBuBisTrip **2** (108 mg, 0.13 mmol, 1.3 equiv). Purification by flash column chromatography (hexane:DCM = 4:1, followed by DCM:MeOH = 90:10, Rf = 0.15) afforded 16.0 mg (75%) of **4fe**as an off-white solid. The compound was fully consistent with literature data [6].

^{1}H NMR (500 MHz, $CDCl_3$) δ 7.95 (s, 1H), 7.45 (d, *J* = 7.7 Hz, 1H), 7.28 (d, *J* = 7.9 Hz, 1H), 7.13 (td, *J* = 7.2, 1.4 Hz, 1H), 7.08 (td, *J* = 7.4, 1.2 Hz, 1H), 3.01 (dd, *J* = 14.4, 4.9 Hz, 1H), 2.94 (q, *J* = 8.0 Hz, 1H), 2.89 – 2.81 (m, 2H), 2.74 – 2.66 (m, 1H), 2.52 (s, 6H), 2.32 – 2.25 (m, 1H), 1.85 (m, 1H).

^{13}C NMR (126 MHz, $CDCl_3$) δ 136.5, 133.2, 127.8, 121.5, 119.5, 117.7, 110.7, 108.3, 61.8, 41.4, 26.1, 23.0, 22.5.

HRMS (ESI) calculated for $[C_{14}H_{18}N_2]^+$ 214.1470, found: 214.1442

1,2,3,4-tetrahydrocyclopenta[_b_]indole (4f)

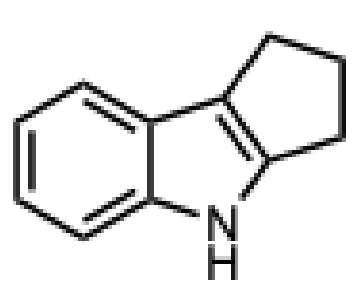

The compound was synthesized according to the **general procedure A**, employing cyclopentanone (8.4 mg, 0.10 mmol, 1.0 equiv), LiHMDS (0.11 mL, 1.0 M THF, 0.11 mmol, 1.1 equiv), and aryl-tBuBisTrip **2** (108 mg, 0.13 mmol, 1.3 equiv). Purification by flash column chromatography (hexane:DCM = 4:1, followed by hexane:ethyl acetate = 95:5, Rf = 0.35) afforded 10.2 mg (65%) of **4f** as a yellow solid. The compound was fully consistent with literature data [3].

^{1}H NMR (500 MHz, $CDCl_3$) δ 7.82 (s, 1H), 7.46 – 7.41 (m, 1H), 7.32 – 7.28 (m, 1H), 7.08 (tt, J = 7.2, 5.5 Hz, 2H), 2.89 – 2.81 (m, 4H), 2.54 (tt, J = 7.8, 6.3 Hz, 2H).

^{13}C NMR (126 MHz, $CDCl_3$) δ 143.8, 141.1, 124.9, 120.6, 120.0, 119.6, 118.6, 111.4, 28.8, 26.0, 24.6.

HRMS (ESI) calculated for $[C_{11}H_{11}N+H]^+$ 158.0964, found: 158.0965

5,6,7,8,9,10-hexahydrocyclohepta[*b*]indole (4g)

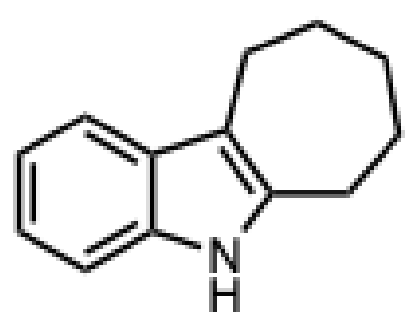

The compound was synthesized according to the **general procedure A**, employing cycloheptanone (11.2 mg, 0.10 mmol, 1.0 equiv), LiHMDS (0.11 mL, 1.0 M THF, 0.11 mmol, 1.1 equiv), and aryl-tBuBisTrip **2** (108 mg, 0.13 mmol, 1.3 equiv). Purification by flash column chromatography (hexane:DCM = 4:1, followed by hexane:ethyl acetate = 95:5, Rf = 0.4) afforded 16.1 mg (87%) of **4g** as a white solid. The compound was fully consistent with literature data [3].

^{1}H NMR (600 MHz, $CDCl_3$) δ 7.66 (s, 1H), 7.48 (d, *J* = 7.2 Hz 1H), 7.29 – 7.23 (m, 1H), 7.14 – 7.05 (m, 2H), 2.88 – 2.77 (m, 4H), 1.91 (d, *J* = 5.5 Hz, 2H), 1.79 (d, *J* = 5.5 Hz, 4H).

^{13}C NMR (150 MHz, $CDCl_3$) δ 137.5, 134.4, 129.4, 120.7, 119.1, 117.8, 113.9, 110.3, 31.9, 29.7, 28.9, 27.7, 24.8.

HRMS (ESI) calculated for $[C_{13}H_{15}N+H]^+$ 186.1277, found:186.1274

2,3,4,9-tetrahydro-1*H*-carbazole (4h)

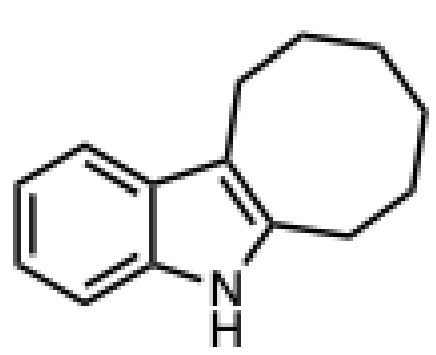

The compound was synthesized according to the **general procedure A**, employing cyclooctanone (12.6 mg, 0.10 mmol, 1.0 equiv), LiHMDS (0.11 mL, 1.0 M THF, 0.11 mmol, 1.1 equiv), and aryl-tBuBisTrip **2** (108 mg, 0.13 mmol, 1.3 equiv). Purification by flash column chromatography (hexane:DCM = 4:1, followed by hexane:ethyl acetate = 95:5, Rf = 0.4) afforded 17.0 mg (85%) of **4h** as a yellow solid. The compound was fully consistent with literature data [3].

^{1}H NMR (500 MHz, $CDCl_3$) δ 7.67 (s, 1H), 7.50 (d, *J* = 7.5 Hz, 1H), 7.32 – 7.26 (m, 1H), 7.16 – 7.05 (m, 2H), 2.93 – 2.80 (m, 4H), 1.76 (dt, *J* = 13.1, 6.4 Hz, 4H), 1.52 – 1.40 (m, 4H).

^{13}C NMR (126 MHz, $CDCl_3$) δ 135.7, 135.2, 128.8, 120.8, 119.1, 117.8, 111.9, 110.4, 29.7, 29.6, 26.2, 26.0, 22.3.

HRMS (ESI) calculated for $[C_{14}H_{17}N+H]^+$ 200.1434, found: 200.1434

1,3,4,5-tetrahydropyrano[4,3-*b*]indole (4i)

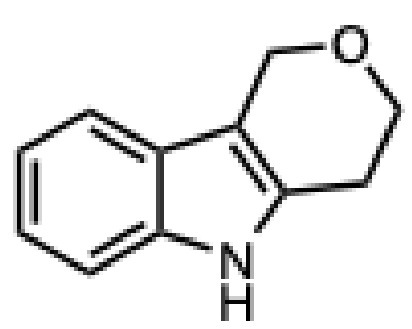

The compound was synthesized according to the **general procedure A**, employing tetrahydro-4*H*-pyran-4-one (10.0 mg, 0.10 mmol, 1.0 equiv), LiHMDS (0.11 mL, 1.0 M THF, 0.11 mmol, 1.1 equiv), and aryl-tBuBisTrip **2** (108 mg, 0.13 mmol, 1.3 equiv). Purification by flash column chromatography (hexane:DCM = 4:1, followed by hexane:ethyl acetate = 80:20, Rf = 0.3) afforded 12.3 mg (71%) of **4i** as a yellow solid.

^{1}H NMR (500 MHz, $CDCl_3$) δ 7.89 (s, 1H), 7.39 (d, *J* = 7.8 Hz, 1H), 7.32 (d, *J* = 8.0 Hz, 1H), 7.16 (td, *J* = 7.2, 1.2 Hz, 1H), 7.10 (td, *J* = 7.5, 1.1 Hz, 1H), 4.94 (m, 2H), 4.07 (t, *J* = 5.5 Hz, 2H), 2.86 (m, 2H).

^{13}C NMR (150 MHz, $CDCl_3$) δ 135.7, 130.9, 125.2, 121.6, 119.7, 117.8, 110.9, 108.9, 64.7, 64.4, 24.3.

HRMS (ESI) calculated for $[C_{11}H_{11}NO+H]^+$ 174.0913, found: 174.0910

1,3,4,5-tetrahydrothiopyrano[4,3-*b*]indole (4j)

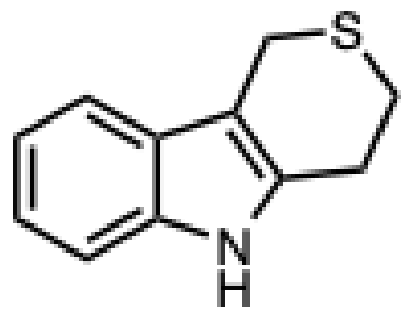

The compound was synthesized according to the **general procedure A**, employing tetrahydro-4*H*-thiopyran-4-one (11.6 mg, 0.10 mmol, 1.0 equiv), LiHMDS (0.11 mL, 1.0 M THF, 0.11 mmol, 1.1 equiv), and aryl-tBuBisTrip **2** (108 mg, 0.13 mmol, 1.3 equiv). Purification by flash column chromatography (hexane:DCM = 4:1, followed by hexane:ethyl acetate = 90:10, Rf = 0.3) afforded 13.6 mg (72%) of **4j** as an off-white solid. The compound was fully consistent with literature data [4].

^{1}H NMR (600 MHz, $CDCl_3$) δ 7.73 (s, 1H), 7.46 (d, *J* = 7.7 Hz, 1H), 7.28 (d, *J* = 8.0 Hz, 1H), 7.17 (t, *J* = 7.5 Hz, 1H), 7.12 (t, *J* = 7.4 Hz, 1H), 3.88 (s, 2H), 3.01 (s, 4H).

^{13}C NMR (150 MHz, $CDCl_3$) δ 134.6, 133.3, 127.1, 121.8, 119.7, 117.7, 110.6, 106.9, 25.8, 25.3, 22.9.

HRMS (ESI) calculated for $[C_{11}H_{11}NS+H]^+$ 190.0685, found: 190.0684

ethyl 1,3,4,5-tetrahydro-2*H*-pyrido[4,3-*b*]indole-2-carboxylate (4k)

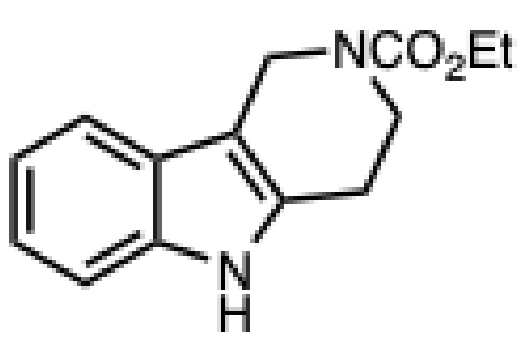

The compound was synthesized according to the **general procedure A**, employing ethyl 4-oxopiperidine-1-carboxylate (17.1 mg, 0.10 mmol, 1.0 equiv), LiHMDS (0.11 mL, 1.0 M THF, 0.11 mmol, 1.1 equiv), and aryl-tBuBisTrip **2** (108 mg, 0.13 mmol, 1.3 equiv). Purification by flash column chromatography (hexane:DCM = 4:1, followed by hexane:ethyl acetate = 1:1, Rf = 0.2) afforded 13.9 mg (57%) of **4k** as a slightly yellow solid. The compound was fully consistent with literature data [7].

^{1}H NMR (500 MHz, $CDCl_3$) δ 7.93 (s, 1H), 7.46 (d, J = 7.7 Hz, 1H), 7.31 (d, J = 8.0 Hz, 1H), 7.21 – 7.05 (m, 2H), 4.79 – 4.63 (m, 2H), 4.21 (q, J = 7.1 Hz, 2H), 3.96 – 3.80 (m, 2H), 2.91 –2.78 (m, 2H), 1.31 (t, J = 7.1 Hz, 3H).

^{13}C NMR (126 MHz, $CDCl_3$) δ 156.2, 135.9, 132.0, 125.6, 121.7, 119.7, 117.6, 110.8, 107.3, 61.6, 43.1, 41.3, 23.4, 14.8.

HRMS (ESI) calculated for $[C_{14}H_{16}N_2O_2+H]^+$ 245.1285, found: 245.1287

1,2,4,9-tetrahydrospiro[carbazole-3,2'-[1,3]dioxolane] (4l)

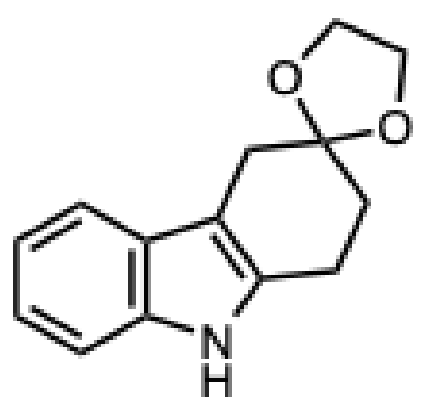

The compound was synthesized according to the **general procedure A**, employing 1,4-dioxaspiro[4.5]decan-8-one (15.6 mg, 0.10 mmol, 1.0 equiv), LiHMDS (0.11 mL, 1.0 M THF, 0.11 mmol, 1.1 equiv), and aryl-tBuBisTrip **2** (108 mg, 0.13 mmol, 1.3 equiv). Purification by flash column chromatography (hexane:DCM = 4:1, followed by hexane:acetone = 80:20, Rf = 0.2) afforded 11.8 mg (51%) of **4l** as a white solid. The compound was fully consistent with literature data [3].

^{1}H NMR (500 MHz, $CDCl_3$) δ 7.75 (s, 1H), 7.41 (dd, J = 7.7, 1.2 Hz, 1H), 7.28 – 7.25 (m, 2H), 7.11 (ddd, J = 8.0, 7.0, 1.3 Hz, 1H), 7.06 (ddd, J = 8.1, 7.2, 1.2 Hz, 1H), 4.11 – 4.02 (m, 4H), 2.97 (s, 2H), 2.95 (td, J = 6.5, 3.3 Hz, 2H), 2.09 (t, J = 6.5 Hz, 2H).

^{13}C NMR (126 MHz, $CDCl_3$) δ 136.8, 132.4, 127.9, 121.5, 119.4, 117.8, 110.6, 109.3, 108.5, 64.8, 32.1, 32.0, 21.7.

HRMS (ESI) calculated for $[C_{14}H_{15}NO_2+H]^+$ 230.1176, found:230.1179

2-phenyl-1*H*-indole (4m)

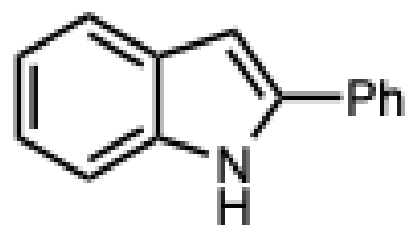

The compound was synthesized according to the **general procedure A**, employing acetophenone (12.0 mg, 0.10 mmol, 1.0 equiv), LiHMDS (0.11 mL, 1.0 M THF, 0.11 mmol, 1.1 equiv), and aryl-tBuBisTrip **2** (108 mg, 0.13 mmol, 1.3 equiv). Purification by flash column chromatography (hexane:DCM = 4:1, followed by hexane:ethyl acetate = 95:5, Rf = 0.35) afforded 12.3 mg (63%) of **4m** as a yellow solid. The compound was fully consistent with literature data [8].

^{1}H NMR (500 MHz, $CDCl_3$) δ 8.33 (s, 1H), 7.71 – 7.62 (m, 3H), 7.46 (t, *J* = 7.8 Hz, 2H), 7.41 (d, *J* = 8.1 Hz, 1H), 7.37 – 7.32 (m, 1H), 7.22 (ddd, *J* = 8.2, 7.0, 1.2 Hz, 1H), 7.15 (td, *J* = 7.5, 1.0 Hz, 1H), 6.85 (d, *J* = 2.2 Hz, 1H).

^{13}C NMR (126 MHz, $CDCl_3$) δ 138.0, 137.0, 132.5, 129.4, 129.2, 127.9, 125.3, 122.5, 120.8, 120.4, 111.0, 100.1.

HRMS (ESI) calculated for $[C_{14}H_{11}N+H]^+$ 194.0964, found:194.0966

2-(naphthalen-2-yl)-1H-indole (4n)

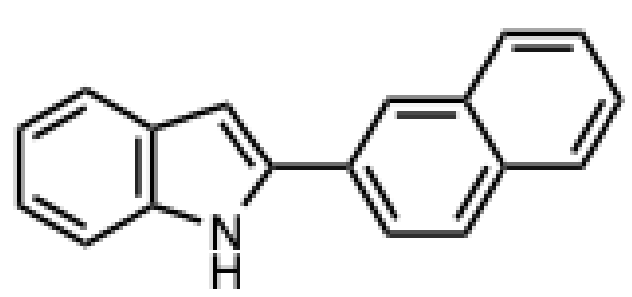

The compound was synthesized according to the **general procedure A**, employing 1-(naphthalen-2-yl)ethan-1-one (17.0 mg, 0.10 mmol, 1.0 equiv), LiHMDS (0.11 mL, 1.0 M THF, 0.11 mmol, 1.1 equiv), and aryl-tBuBisTrip **2** (108 mg, 0.13 mmol, 1.3 equiv). Purification by flash column chromatography (hexane:DCM = 4:1, followed by hexane:ethyl acetate = 95:5, Rf = 0.3) afforded 10.1 mg (42%) of **4n** as a white solid. The compound was fully consistent with literature data [8].

^{1}H NMR (500 MHz, $CDCl_3$) δ 8.49 (s, 1H), 8.07 (d, J = 1.3 Hz, 1H), 7.94 – 7.83 (m, 4H), 7.68 (dd, J = 7.9, 1.0 Hz, 1H), 7.51 (dddd, J = 21.1, 8.1, 6.8, 1.4 Hz, 2H), 7.45 (dd, J = 8.1, 0.9 Hz, 1H), 7.24 (ddd, J = 8.1, 7.0, 1.2 Hz, 1H), 7.16 (ddd, J = 8.0, 7.0, 1.0 Hz, 1H), 6.98 (dd, J = 2.2, 0.9 Hz, 1H).

^{13}**C NMR** (150 MHz, $CDCl_3$) δ 138.0, 137.2, 133.8, 133.0, 129.8, 129.5, 128.9, 128.1, 128.0, 126.8, 126.3, 123.9, 123.2, 122.7, 120.9, 120.5, 111.1, 100.8.

HRMS (ESI) calculated for $[C_{18}H_{13}N+H]^+$ 244.1121, found: 244.1121

3-ethyl-2-phenyl-1*H*-indole (4o)

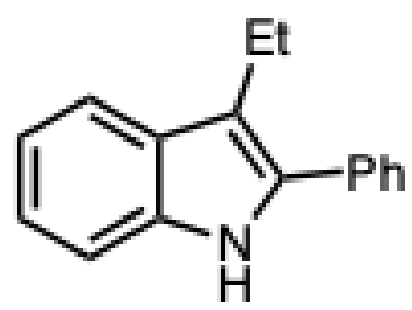

The compound was synthesized according to the **general procedure A**, employing butyrophenone (14.8 mg, 0.10 mmol, 1.0 equiv), LiHMDS (0.11 mL, 1.0 M THF, 0.11 mmol, 1.1 equiv), and aryl-tBuBisTrip **2** (108 mg, 0.13 mmol, 1.3 equiv). Purification by flash column chromatography (hexane:DCM = 4:1, followed by hexane:ethyl acetate = 95:5, Rf = 0.3) afforded 12.1 mg (56%) of **4o** as a yellow solid. The compound was fully consistent with literature data [9].

^{1}**H NMR** (500 MHz, $CDCl_3$) δ 7.98 (s, 1H), 7.66 (d, J = 7.9 Hz, 1H), 7.56 (t, J = 7.6 Hz, 2H), 7.48 (t, J = 7.6 Hz, 2H), 7.41 – 7.34 (m, 2H), 7.21 (t, J = 7.3 Hz, 1H), 7.15 (t, J = 7.4 Hz, 1H), 2.93 (q, J = 7.6 Hz, 2H), 1.36 (t, J = 7.5 Hz, 3H).

^{13}**C NMR** (126 MHz, $CDCl_3$) δ 136.1, 133.9, 133.6, 129.2, 129.0, 128.0, 127.6, 122.4, 119.6, 119.4, 115.6, 110.9, 17.9, 15.8.

HRMS (ESI) calculated for $[C_{16}H_{15}N+H]^+$ 222.1277, found: 222.1284

2-(4-methoxyphenyl)-3-phenyl-1*H*-indole (4p)

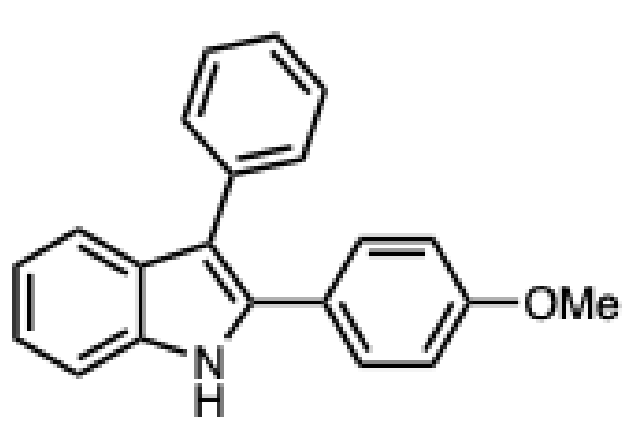

The compound was synthesized according to the **general procedure A**, employing 1-(4-methoxyphenyl)-2-phenylethan-1-one (22.6 mg, 0.10 mmol, 1.0 equiv), LiHMDS (0.11 mL, 1.0

M THF, 0.11 mmol, 1.1 equiv), and aryl-tBuBisTrip **2** (108 mg, 0.13 mmol, 1.3 equiv). Purification by flash column chromatography (hexane:DCM = 4:1, followed by hexane:ethyl acetate = 90:10, Rf = 0.25) afforded 23.3 mg (78%) of **4p** as a dark yellow solid. The compound was fully consistent with literature data [10].

^{1}H NMR (600 MHz, $CDCl_3$) δ 8.16 (s, 1H), 7.70 (d, *J* = 7.9 Hz, 1H), 7.46 (d, *J* = 7.5 Hz, 2H), 7.44 – 7.34 (m, 5H), 7.30 (t, *J* = 7.3 Hz, 1H), 7.24 (d, *J* = 7.7 Hz, 1H), 7.17 (t, *J* = 7.5 Hz, 1H), 6.88 (d, *J* = 8.3 Hz, 2H), 3.82 (s, 3H).

^{13}C NMR (150 MHz, $CDCl_3$) δ 159.4, 135.9, 135.4, 134.3, 130.3, 129.6, 129.0, 128.6, 126.2, 125.3, 122.5, 120.5, 119.6, 114.3, 110.9, 55.4.

HRMS (ESI) calculated for $[C_{21}H_{17}NO+H]^+$ 300.1383, found: 300.1379

2-phenyl-3-(*p*-tolyl)-1*H*-indole (4q)

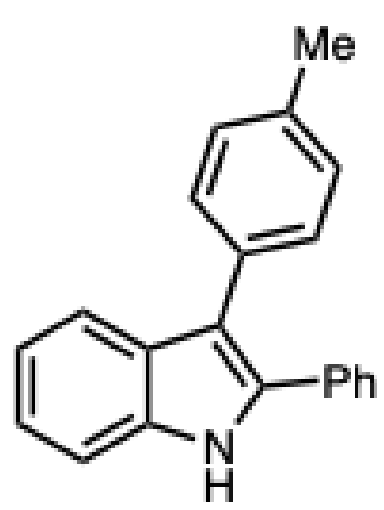

The compound was synthesized according to the **general procedure A**, employing 1-phenyl-2-(*p*-tolyl)ethan-1-one (21.7 mg, 0.10 mmol, 1.0 equiv), LiHMDS (0.11 mL, 1.0 M THF, 0.11 mmol, 1.1 equiv), and aryl-tBuBisTrip **2** (108 mg, 0.13 mmol, 1.3 equiv). Purification by flash column chromatography (hexane:DCM = 4:1, followed by hexane:ethyl acetate = 95:5, Rf = 0.35) afforded 20.3 mg (72%) of **4q** as a white solid. The compound was fully consistent with literature data [11].

^{1}H NMR (500 MHz, $CDCl_3$) δ 8.20 (s, 1H), 7.68 (d, *J* = 8.0 Hz, 1H), 7.44 (t, *J* = 8.6 Hz, 3H), 7.37 – 7.27 (m, 5H), 7.26 – 7.22 (m, 1H), 7.20 (d, *J* = 7.7 Hz, 2H), 7.15 (t, *J* = 7.5 Hz, 1H), 2.40 (s, 3H).

^{13}C NMR (126 MHz, $CDCl_3$) δ 135.9, 135.8, 133.9, 132.9, 132.0, 130.0, 129.3, 128.9, 128.7, 128.1, 127.6, 122.7, 120.4, 119.8, 115.0, 110.9, 21.3.

HRMS (ESI) calculated for $[C_{21}H_{17}N+H]^+$ 284.1434, found: 284.1430

2-(4-bromophenyl)-3-phenyl-1*H*-indole (4r)

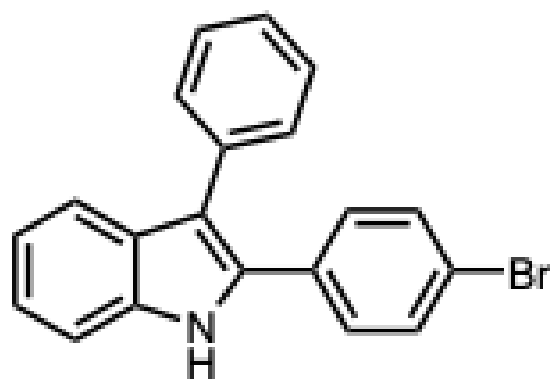

The compound was synthesized according to the **general procedure A**, employing 1-(4-bromophenyl)-2-phenylethan-1-one (27.5 mg, 0.10 mmol, 1.0 equiv), LiHMDS (0.11 mL, 1.0 M THF, 0.11 mmol, 1.1 equiv), and aryl-tBuBisTrip **2** (108 mg, 0.13 mmol, 1.3 equiv). Purification by flash column chromatography (hexane:DCM = 4:1, followed by hexane:ethyl acetate = 95:5, Rf = 0.35) afforded 32.7 mg (94%) of **4r** as an off-white solid. The compound was fully consistent with literature data [12].

^{1}H NMR (500 MHz, $CDCl_3$) δ 8.16 (s, 1H), 7.68 (d, *J* = 7.9 Hz, 1H), 7.50 – 7.36 (m, 7H), 7.36 – 7.26 (m, 4H), 7.18 (t, *J* = 7.5 Hz, 1H).

^{13}C NMR (126 MHz, $CDCl_3$) δ 136.0, 134.7, 132.8, 131.9, 131.6, 130.1, 129.6, 128.8, 128.7, 126.5, 123.1, 121.8, 120.7, 119.8, 115.7, 111.0, 77.1.

HRMS (ESI) calculated for $[C_{20}H_{14}BrN+H]^+$ 348.0382, found: 348.0382

3-ethyl-2-(furan-2-yl)-1*H*-indole (4s)

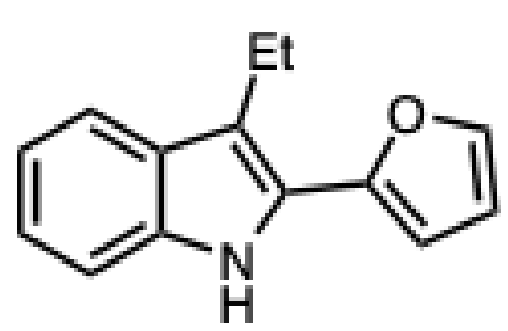

The compound was synthesized according to the **general procedure A**, employing 1-(furan-2-yl)butan-1-one (13.8 mg, 0.10 mmol, 1.0 equiv), LiHMDS (0.11 mL, 1.0 M THF, 0.11 mmol, 1.1 equiv), and aryl-tBuBisTrip **2** (108 mg, 0.13 mmol, 1.3 equiv). Purification by flash column chromatography (hexane:DCM = 4:1, followed by hexane:acetone = 95:5, Rf = 0.3) afforded 15.4 mg (73%) of **4s** as a yellow gel.

^{1}**H NMR** (500 MHz, $CDCl_3$) δ 8.31 (s, 1H), 7.63 (d, *J* = 7.9 Hz, 1H), 7.50 (s, 1H), 7.36 (d, *J* = 8.0 Hz, 1H), 7.21 (t, *J* = 7.5 Hz, 1H), 7.14 (t, *J* = 7.4 Hz, 1H), 6.68 – 6.49 (m, 2H), 2.99 (q, *J* = 7.6 Hz, 2H), 1.32 (t, *J* = 7.5 Hz, 3H).

^{13}**C NMR** (126 MHz, $CDCl_3$) δ 147.8, 141.4, 135.8, 128.8, 124.9, 122.7, 119.7, 119.1, 115.5, 112.0, 110.9, 106.1, 18.0, 14.8.

HRMS (ESI) calculated for $[C_{14}H_{13}NO+H]^+$ 212.1070, found: 212.1071

3-methyl-2-(thiophen-2-yl)-1*H*-indole (4t)

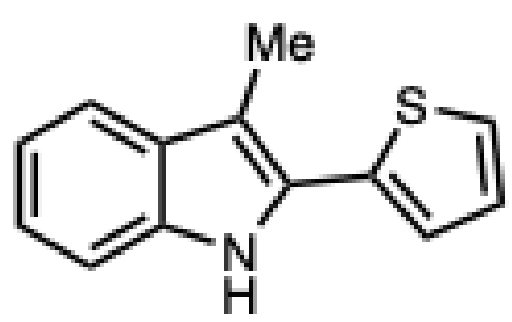

The compound was synthesized according to the **general procedure A**, employing 1-(thiophen-2-yl)propan-1-one (14.0 mg, 0.10 mmol, 1.0 equiv), LiHMDS (0.11 mL, 1.0 M THF, 0.11 mmol, 1.1 equiv), and aryl-tBuBisTrip **2** (108 mg, 0.13 mmol, 1.3 equiv). Purification by flash column chromatography (hexane:DCM = 4:1, followed by hexane:ethyl acetate = 95:5, Rf = 0.3) afforded 11.5 mg (54%) of **4t** as a yellow solid.

^{1}**H NMR** (600 MHz, $CDCl_3$) δ 8.02 (s, 1H), 7.60 (d, *J* = 7.9 Hz, 1H), 7.40 – 7.33 (m, 2H), 7.29 – 7.20 (m, 2H), 7.19 – 7.14 (m, 2H), 2.52 (s, 3H).

^{13}**C NMR** (150 MHz, $CDCl_3$) δ 136.0, 135.5, 130.0, 128.5, 127.8, 124.9, 124.2, 122.8, 119.9, 119.1, 110.7, 109.6, 9.9.

HRMS (ESI) calculated for $[C_{13}H_{11}NS+H]^+$ 214.0685, found: 214.0682

3-methyl-2-(pyridin-3-yl)-1*H*-indole (4u)

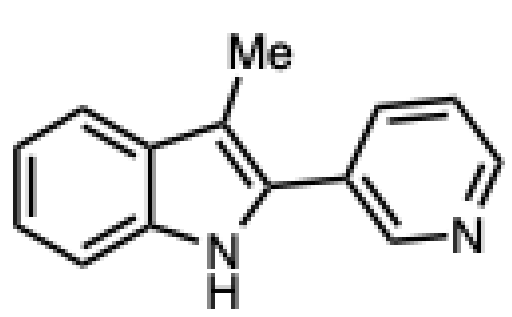

The compound was synthesized according to the **general procedure A**, employing 1-(pyridin-3-yl)propan-1-one (13.5 mg, 0.10 mmol, 1.0 equiv), LiHMDS (0.11 mL, 1.0 M THF, 0.11 mmol, 1.1 equiv), and aryl-tBuBisTrip **2** (108 mg, 0.13 mmol, 1.3 equiv). Purification by flash column chromatography (hexane:DCM = 4:1, followed by hexane:acetone = 80:20, Rf = 0.2) afforded 12.0 mg (58%) of **4u** as an off-white solid.

^{1}H NMR (500 MHz, $CDCl_3$) δ 8.87 (d, *J* = 2.3 Hz, 1H), 8.59 (d, *J* = 4.9 Hz, 1H), 8.40 (s, 1H), 7.89 (dd, *J* = 8.0, 2.1 Hz, 1H), 7.63 (d, *J* = 7.8 Hz, 1H), 7.45 – 7.36 (m, 2H), 7.25 (d, *J* = 6.6 Hz, 1H), 7.18 (t, *J* = 7.4 Hz, 1H), 2.48 (s, 3H).

^{13}C NMR (126 MHz, $CDCl_3$) δ 148.8, 148.3, 136.4, 135.0, 130.7, 129.9, 129.6, 123.8, 123.1, 119.9, 119.3, 111.0, 110.4, 9.7.

HRMS (ESI) calculated for $[C_{14}H_{12}N_2+H]^+$ 209.1073, found: 209.1077

ethyl 3-benzyl-1*H*-indole-2-carboxylate (4v)

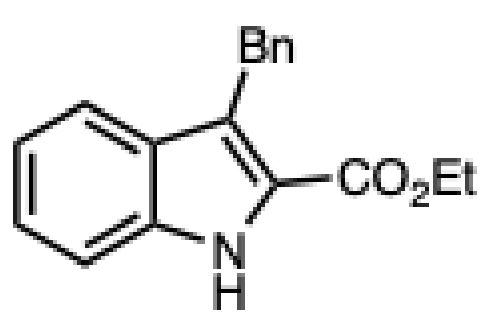

The compound was synthesized according to the **general procedure A**, employing methyl 2-oxo-4-phenylbutanoate (22.4 mg, 0.10 mmol, 1.0 equiv, 92% purity), LiHMDS (0.11 mL, 1.0 M THF, 0.11 mmol, 1.1 equiv), and aryl-tBuBisTrip **2** (108 mg, 0.13 mmol, 1.3 equiv). Purification by flash column chromatography (hexane:DCM = 4:1, followed by hexane:ethyl acetate = 90:10, Rf = 0.25) afforded 18.7 mg (67%) of **4v** as a white solid. The compound was fully consistent with literature data [13].

^{1}H NMR (500 MHz, $CDCl_3$) ^{1}H NMR (500 MHz, $CDCl_3$) δ 8.79 (s, 1H), 7.62 (dd, *J* = 8.1, 1.1 Hz, 1H), 7.41 – 7.37 (m, 1H), 7.32 (td, *J* = 7.0, 1.2 Hz, 1H), 7.29 – 7.26 (m, 2H), 7.23 (t, *J* = 7.7 Hz, 2H), 7.17 – 7.13 (m, 1H), 7.10 (ddd, *J* = 8.0, 6.9, 1.0 Hz, 1H), 4.52 (s, 2H), 4.41 (q, *J* = 7.1 Hz, 2H), 1.38 (t, *J* = 7.1 Hz, 3H).

^{13}C NMR (126 MHz, $CDCl_3$) δ 162.5, 141.1, 136.1, 128.6, 128.4, 128.3, 126.0, 125.8, 123.8, 122.8, 121.4, 120.5, 111.9, 61.0, 30.7, 14.5.

HRMS (ESI) calculated for $[C_{18}H_{17}NO_2+H]^+$ 280.1332, found: 280.1333

methyl 3-methyl-1*H*-indole-2-carboxylate (4w)

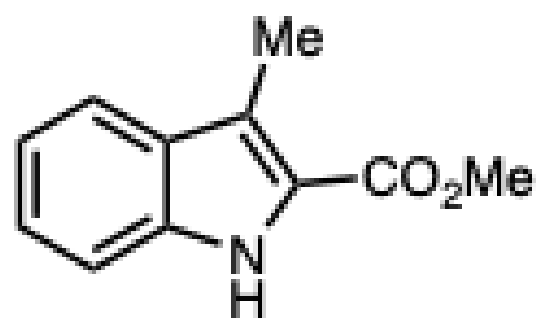

The compound was synthesized according to the **general procedure A**, employing methyl 2-oxobutanoate (11.6 mg, 0.10 mmol, 1.0 equiv), LiHMDS (0.11 mL, 1.0 M THF, 0.11 mmol, 1.1 equiv), and aryl-tBuBisTrip **2** (108 mg, 0.13 mmol, 1.3 equiv). Purification by flash column chromatography (hexane:DCM = 4:1, followed by hexane:ethyl acetate = 90:10, Rf = 0.35) afforded 16.9 mg (37%) of **4w** as a yellow solid. The compound was fully consistent with literature data [14].

^{1}H NMR (500 MHz, $CDCl_3$) δ 8.69 (s, 1H), 7.71 – 7.64 (m, 1H), 7.39 – 7.30 (m, 2H), 7.15 (ddd, J = 8.1, 6.7, 1.2 Hz, 1H), 3.96 (s, 3H), 2.62 (s, 3H).

^{13}C NMR (126 MHz, $CDCl_3$) δ 163.2, 136.0, 128.7, 125.8, 123.3, 121.0, 120.5, 120.1, 111.8, 51.8, 10.1.

methyl 2-methyl-1*H*-indole-3-carboxylate (6a)

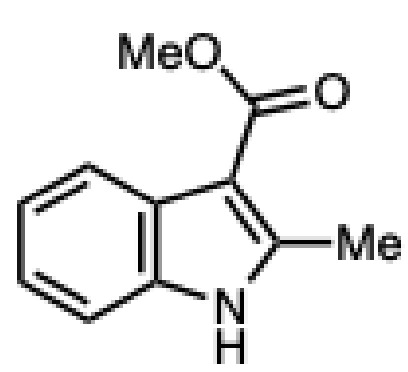

The compound was synthesized according to the **general procedure B**, employing methyl 3-oxobutanoate (11.6 mg, 0.10 mmol, 1.0 equiv), BTMG (22 μL, 0.11 mmol, 1.1 equiv), and aryl-tBuBisTrip **2** (108 mg, 0.13 mmol, 1.3 equiv). Purification by flash column chromatography (hexane:DCM = 4:1, followed by hexane:ethyl acetate = 85:15, Rf = 0.2) afforded 12.5 mg (66%) of **6a** as a yellow solid. The compound was fully consistent with literature data [14].

^{1}H NMR (600 MHz, $CDCl_3$) ^{1}H NMR (600 MHz, $CDCl_3$) δ 8.46 (s, 1H), 8.09 (d, *J* = 7.7 Hz, 1H), 7.30 (d, *J* = 7.7 Hz, 1H), 7.21 (dq, *J* = 14.8, 7.2 Hz, 2H), 3.94 (s, 3H), 2.74 (s, 3H).

^{13}C NMR (150 MHz, $CDCl_3$) δ 166.7, 144.2, 134.6, 127.3, 122.5, 121.9, 121.4, 110.6, 104.6, 50.9, 14.4.

ethyl 2-phenyl-1*H*-indole-3-carboxylate (6b)

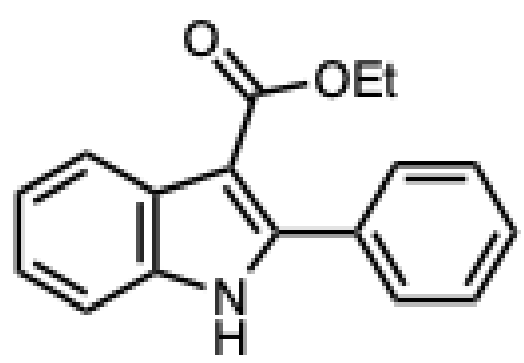

The compound was synthesized according to the **general procedure B**, employing methyl 3-oxo-3-phenylpropanoate (17.8 mg, 0.10 mmol, 1.0 equiv), BTMG (22 µL, 0.11 mmol, 1.1 equiv), and aryl-tBuBisTrip **2** (108 mg, 0.13 mmol, 1.3 equiv). Purification by flash column chromatography (hexane:DCM = 4:1, followed by hexane:ethyl acetate = 90:10, Rf = 0.3) afforded 19.9 mg (75%) of **6b** as a slightly yellow solid. The compound was fully consistent with literature data [9].

^{1}H NMR (500 MHz, $CDCl_3$) δ 8.54 (s, 1H), 8.28 – 8.21 (m, 1H), 7.71 – 7.62 (m, 2H), 7.49 – 7.41 (m, 3H), 7.41 – 7.36 (m, 1H), 7.32 – 7.36 (m, 2H), 4.31 (q, *J* = 7.2 Hz, 2H), 1.32 (t, *J* = 7.1 Hz, 3H).

^{13}C NMR (126 MHz, $CDCl_3$) δ 165.5, 144.6, 135.3, 132.2, 129.8, 129.3, 128.3, 127.8, 123.4, 122.4, 122.2, 111.1, 104.9, 59.8, 14.5.

HRMS (ESI) calculated for $[C_{17}H_{15}NO_2+H]^+$ 266.1176, found: 266.1179

1,2,3,9-tetrahydro-4*H*-carbazol-4-one (6c)

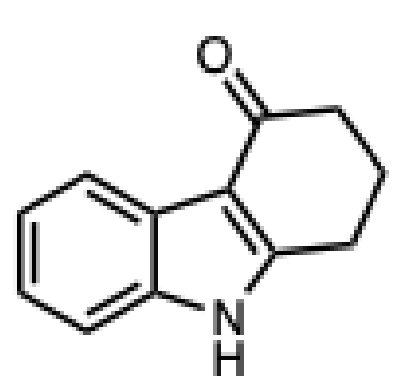

The compound was synthesized according to the **general procedure B**, employing cyclohexane-1,3-dione (11.2 mg, 0.10 mmol, 1.0 equiv), BTMG (22 µL, 0.11 mmol, 1.1 equiv), and aryl-tBuBisTrip **2** (108 mg, 0.13 mmol, 1.3 equiv). Purification by flash column chromatography (hexane:DCM = 4:1, followed by hexane:acetone = 1:1, Rf = 0.5) afforded 7.4 mg (40%) of **6c** as a white solid. The compound was fully consistent with literature data [4].

^{1}H NMR (500 MHz, $CDCl_3$) δ 8.36 (s, 1H), 8.26 – 8.20 (m, 1H), 7.36 – 7.31 (m, 1H), 7.26 – 7.22 (m, 2H), 2.99 (t, J = 6.2 Hz, 2H), 2.60 (dd, J = 7.2, 5.7 Hz, 2H), 2.26 (p, J = 6.3 Hz, 2H).z

^{13}C NMR (101 MHz, $CDCl_3$) δ 194.4, 151.0, 135.7, 125.0, 123.5, 122.7, 121.8, 113.7, 110.9, 38.3, 29.9, 23.9, 23.7.

HRMS (ESI) calculated for $[C_{12}H_{11}NO+H]^+$ 186.0913, found: 186.0912

4-methyl-2,3,4,9-tetrahydro-1*H*-carbazole (8a)

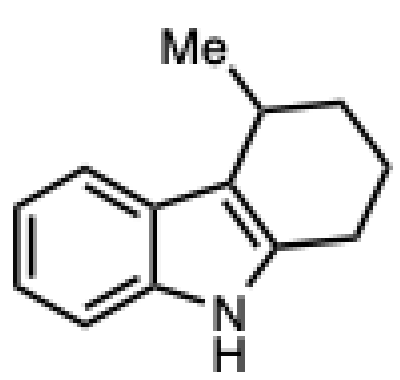

The compound was synthesized according to the **general procedure C**, employing trimethyl((3-methylcyclohex-1-en-1-yl)oxy)silane (18.4 mg, 0.10 mmol, 1.0 equiv), MeLi (70 µL, 1.6 M Et_2O, 0.11 mmol, 1.1 equiv), and aryl-tBuBisTrip **2** (108 mg, 0.13 mmol, 1.3 equiv). Purification by flash column chromatography (hexane:DCM = 4:1, followed by hexane:ethyl acetate = 95:5, Rf = 0.5) afforded 10.1 mg (55%) of **8a** as a yellow solid. The product is spectroscopically identical to compound made using alternate procedure D. The compound was fully consistent with literature data [15].

^{1}H NMR (500 MHz, $CDCl_3$) δ 7.64 (s, 1H), 7.57 (d, *J* = 7.6 Hz, 1H), 7.28 (d, *J* = 7.8 Hz, 1H), 7.15 – 7.04 (m, 2H), 3.17 – 3.08 (m, 1H), 2.74 – 2.64 (m, 2H), 2.05 – 1.94 (m, 2H), 1.87 – 1.76 (m, 1H), 1.64 –1.55 (m, 1H), 1.38 (d, *J* = 6.9 Hz, 3H).

^{13}C NMR (126 MHz, $CDCl_3$) δ 135.9, 133.9, 127.5, 120.9, 119.1, 118.8, 115.2, 110.6, 32.2, 27.3, 23.6, 21.4, 20.5.

HRMS (ESI) calculated for $[C_{13}H_{15}N+H]^+$ 186.1277, found: 186.1278

methyl-2,3,4,9-tetrahydro-1*H*-carbazole (8a')

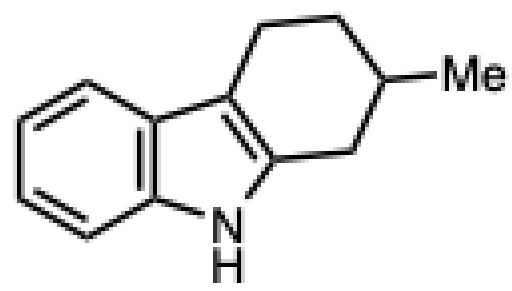

The compounds were synthesized according to the **general procedure A**, employing 3-methylcyclohexan-1-one (11.2 mg, 0.10 mmol, 1.0 equiv), LDA (0.11 mL, 1.0 M THF, 0.11 mmol, 1.1 equiv), and aryl-tBuBisTrip **2** (108 mg, 0.13 mmol, 1.3 equiv). Purification by flash column chromatography (hexane:DCM = 4:1, followed by hexane:ethyl acetate =97:3, Rf = 0.4) afforded 13.7 mg (77%) of **8a'** (and regioisomer, 3:1) as a yellow gel. The compound was fully consistent with literature data [16].

Note: When NaHMDS (0.11 mL, 1.0 M, 0.11 mmol, 1.1 equiv) was used the reaction afforded a 9.5:1 mixture of the same regioisomers with a combined yield of 50%.

^{1}H NMR (500 MHz, $CDCl_3$) δ 7.63 (s, 1H), 7.47 (d, J = 7.5 Hz, 1H), 7.30 – 7.26 (m, 1H), 7.15 – 7.04 (m, 2H), 2.80 (td, J = 14.1, 4.7 Hz, 1H), 2.70 (qt, J = 9.7, 5.1 Hz, 1H), 2.38 (ddt, J = 16.0, 9.7, 2.0 Hz, 1H), 2.10 – 2.02 (m, 1H), 2.02 – 1.93 (m, 1H), 1.54 – 1.47 (m, 1H), 1.38 (d, J = 6.9 Hz, 1H), 1.14 (d, J = 6.7 Hz, 2H).

^{13}C NMR (126 MHz, $CDCl_3$) δ 136.0, 135.9, 134.2, 133.9, 127.8, 127.5, 121.1, 120.9, 119.2, 119.1, 118.8, 117.9, 115.2, 110.6, 110.5, 110.0, 32.2, 31.8, 31.6, 29.8, 27.3, 23.6, 21.8, 21.4, 20.6, 20.5.

HRMS (ESI) calculated for $[C_{13}H_{15}N+H]^+$ 186.1277, found: 186.1275

3-ethyl-2-phenyl-1*H*-indole (8b)

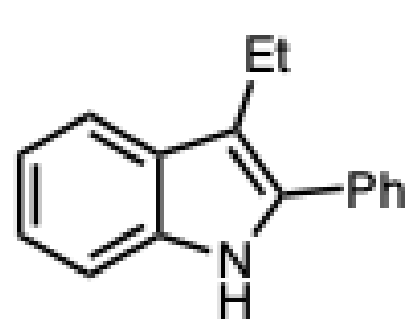

The compound was synthesized according to the **general procedure C**, employing trimethyl((1-phenylbut-1-en-1-yl)oxy)silane (22.0 mg, 0.10 mmol, 1.0 equiv), MeLi (70 µL, 1.6 M Et_2O, 0.11 mmol, 1.1 equiv), and aryl-tBuBisTrip **2** (108 mg, 0.13 mmol, 1.3 equiv). Purification by flash column chromatography (hexane:DCM = 4:1, followed by hexane:ethyl acetate = 95:5, Rf = 0.3) afforded 18.0 mg (81%) of **8b** as a yellow solid. The product is spectroscopically identical to compound made using alternate procedure (see compound **4o**).

^{1}H NMR (500 MHz, $CDCl_3$) δ 7.98 (s, 1H), 7.66 (d, *J* = 7.9 Hz, 1H), 7.56 (t, *J* = 7.6 Hz, 2H), 7.48 (t, *J* = 7.6 Hz, 2H), 7.41 – 7.34 (m, 2H), 7.21 (t, *J* = 7.3 Hz, 1H), 7.15 (t, *J* = 7.4 Hz, 1H), 2.93 (q, *J* = 7.6 Hz, 2H), 1.36 (t, *J* = 7.5 Hz, 3H).

^{13}C NMR (126 MHz, $CDCl_3$) δ 136.1, 133.9, 133.6, 129.2, 129.0, 128.0, 127.6, 122.4, 119.6, 119.4, 115.6, 110.9, 17.9, 15.8.

HRMS (ESI) calculated for $[C_{16}H_{15}N+H]^+$ 222.1277, found: 222.1284

4-phenyl-2,3,4,9-tetrahydro-1*H*-carbazole (8c)

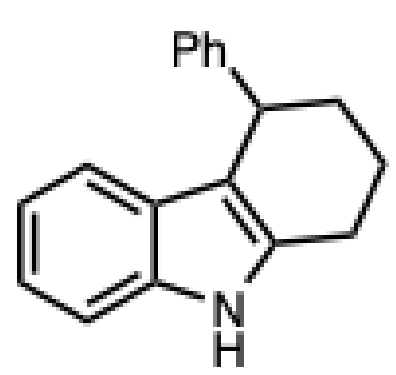

The compound was synthesized according to the **general procedure C**, employing trimethyl((3-phenylcyclohex-1-en-1-yl)oxy)silane (18.4 mg, 0.10 mmol, 1.0 equiv), MeLi (70 µL, 1.6 M Et_2O, 0.11 mmol, 1.1 equiv), and aryl-tBuBisTrip **2** (108 mg, 0.13 mmol, 1.3 equiv). Purification by flash column chromatography (hexane:DCM = 4:1, followed by hexane:ethyl acetate = 90:10, Rf = 0.35) afforded 14.1 mg (57%) of **8c** as a yellow solid. The product is spectroscopically identical to compound made using alternate procedure D. The compound was fully consistent with literature data [17].

^{1}H NMR (500 MHz, $CDCl_3$) δ 7.77 (s, 1H), 7.29 (t, *J* = 7.7 Hz, 3H), 7.22 (q, *J* = 2.7 Hz, 3H), 7.12 – 7.05 (m, 1H), 6.87 (dt, *J* = 15.8, 7.8 Hz, 2H), 4.29 – 4.18 (m, 1H), 2.92 – 2.75 (m, 2H), 2.27 (ddd, *J* = 10.4, 8.1, 5.7 Hz, 1H), 2.05 – 1.97 (m, 1H), 1.86 (qd, *J* = 10.0, 5.9 Hz, 2H).

^{13}C NMR (126 MHz, $CDCl_3$) δ 146.3, 136.0, 135.6, 128.3, 128.3, 127.5, 126.1, 121.1, 119.3, 119.2, 112.1, 110.4, 39.9, 34.6, 23.5, 21.2.

HRMS (ESI) calculated for $[C_{18}H_{17}N+H]^+$ 248.1434, found: 248.1427

3-phenyl-1H-indole (8d)

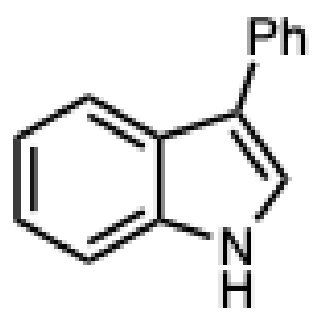

The compound was synthesized according to the **general procedure C**, employing trimethyl(styryloxy)silane (19.2 mg, 0.10 mmol, 1.0 equiv)[18], MeLi (70 µL, 1.6 M Et_2O, 0.11 mmol, 1.1 equiv), and aryl-tBuBisTrip **2** (108 mg, 0.13 mmol, 1.3 equiv). Purification by flash column chromatography (hexane:DCM = 4:1, followed by hexane:ethyl acetate = 90:10) afforded 13.1 mg (68%) of **8d** as a yellow solid. The compound was fully consistent with literature data [19].

^{1}H NMR (500 MHz, $CDCl_3$) δ 8.23 (s, 1H), 7.96 (dd, J = 8.0, 1.1 Hz, 1H), 7.69 (dd, J = 8.2, 1.3 Hz, 2H), 7.45 (dt, J = 8.2, 7.2 Hz, 3H), 7.38 (d, J = 2.5 Hz, 1H), 7.33 – 7.24 (m, 3H), 7.21 (ddd, J = 8.1, 7.0, 1.1 Hz, 1H).

^{13}C NMR (126 MHz, $CDCl_3$) δ 136.8, 135.7, 128.9, 127.6, 126.1, 125.9, 122.6, 121.9, 120.5, 120.0, 118.6, 111.5.

HRMS (ESI) calculated for $[C_{14}H_{11}N+H]^+$ 194.0964, found: 194.0958

***S*-phenethyl 2-methyl-2-(2,3,4,9-tetrahydro-1*H*-carbazol-4-yl)propanethioate (10c)**

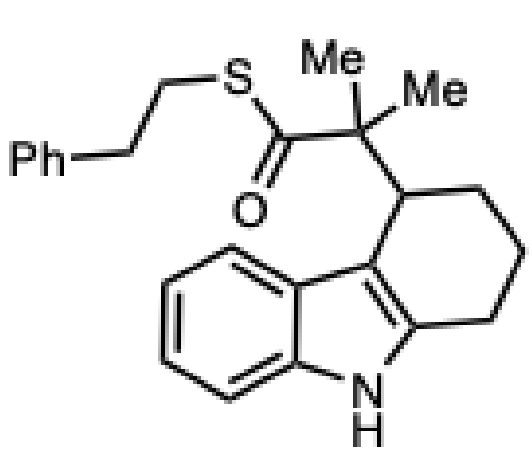

The compound was synthesized according to a ***modified*** **general procedure D**. A dry 5 mL screw-cap vial equipped with a stir bar and maintained under nitrogen was charged with *S*-phenethyl 2-methylpropanethioate (22.9 mg, 0.11 mmol, 1.1 equiv) and dry THF (1.0 mL). The solution was cooled to –78 °C, and LDA (0.11 mL, 1.0 M THF/hexanes, 0.11 mmol, 1.1 equiv) was added dropwise. After stirring for 30 minutes, the solution was warmed to –20 °C and cyclohex-2-en-1-one (9.6 mg, 0.10 mmol, 1.0 equiv) was added. After 20 minutes, the solution was cooled to –78 °C again and aryl-tBuBisTrip **2** (0.13 mmol, 1.3 equiv) was added in one portion as a solid, and the resulting mixture was stirred at –78 °C for 2 minutes. The reaction was then allowed to warm to room temperature and stirred for an additional 15 min before being quenched with H_2O (0.5 mL). The mixture was extracted with DCM (3 × 5.0 mL), and the combined organic phases were dried over anhydrous Na_2SO_4, filtered, and concentrated in vacuo. The resulting crude was purified via column chromatography (hexane:DCM = 4:1, followed by hexane:ethyl acetate = 90:10, Rf = 0.25) afforded 20.7 mg (55%) of **10c** as a yellow solid.

^{1}H NMR (500 MHz, $CDCl_3$) δ 7.81 (s, 1H), 7.56 (d, *J* = 7.9 Hz, 1H), 7.35 – 7.30 (m, 2H), 7.29 – 7.22 (m, 4H), 7.10 (td, *J* = 7.2, 1.2 Hz, 1H), 7.05 (td, *J* = 7.5, 1.2 Hz, 1H), 3.7 – 3.6 (m, 1H), 3.23 – 3.13 (m, 2H), 2.96 – 2.87 (m, 2H), 2.80 – 2.64 (m, 2H), 2.17 – 2.05 (m, 1H), 1.89 – 1.82 (m, 1H), 1.82 – 1.72 (m, 2H), 1.40 (s, 3H), 1.19 (s, 3H).

^{13}C NMR (126 MHz, $CDCl_3$) δ 207.6, 140.4, 136.8, 135.9, 128.8, 128.6, 128.5, 126.6, 121.0, 120.3, 119.2, 110.4, 110.2, 55.9, 39.6, 36.0, 30.3, 28.0, 26.4, 22.8, 22.7, 20.2.

HRMS (ESI) calculated for $[C_{24}H_{27}NOS+H]^+$ 378.1886, found: 378.1888

4,9-dihydro-3*H*-carbazole (12a)

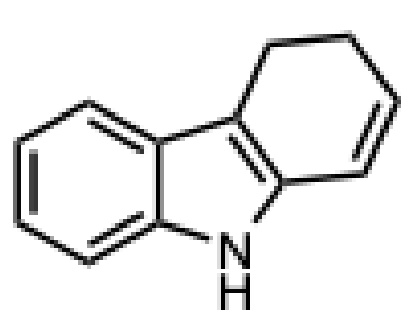

The compound was synthesized according to the **general procedure A**, employing cyclohex-2-en-1-one (9.6 mg, 0.10 mmol, 1.0 equiv), LiHMDS (0.11 mL, 1.0 M THF, 0.11 mmol, 1.1 equiv), and aryl-tBuBisTrip **2** (108 mg, 0.13 mmol, 1.3 equiv). Purification by flash column chromatography (hexane:DCM = 4:1, followed by hexane:ethyl acetate = 95:5, Rf = 0.45) afforded

10.3 mg (61%) of **12a** as an off-white solid. The compound was fully consistent with literature data [15].

Note: The compound is unstable and undergoes rapid aromatization to carbazole at room temperature, as observed in the NMR spectrum.

^{1}H NMR (500 MHz, $CDCl_3$) δ 7.78 (s, 1H), 7.52 – 7.46 (m, 1H), 7.32 – 7.28 (m, 1H), 7.16 – 7.07 (m, 2H), 6.42 (dt, *J* = 9.8, 2.1 Hz, 1H), 5.98 (dt, *J* = 9.4, 4.4 Hz, 1H), 2.90 (t, *J* = 9.2 Hz, 2H), 2.56 – 2.48 (m, 2H).

^{13}C NMR (126 MHz, $CDCl_3$) δ 136.1, 133.1, 128.3, 127.2, 121.5, 119.9, 118.7, 118.4, 111.0, 109.3, 24.5, 19.3.

HRMS (ESI) calculated for $[C_{12}H_{11}N+H]^+$ 170.0964, found: 170.0963

5,8,9,10-tetrahydrocyclohepta[b]indole (12b)

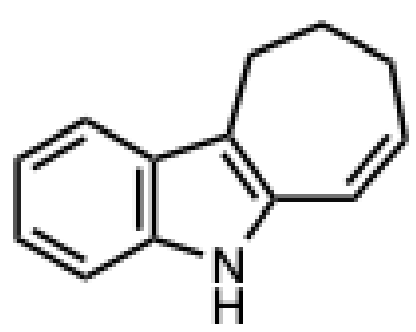

The compound was synthesized according to the **general procedure A**, employing 2-cyclohepten-1-one (11.0 mg, 0.10 mmol, 1.0 equiv), LiHMDS (0.11 mL, 1.0 M THF, 0.11 mmol, 1.1 equiv), and aryl-tBuBisTrip **2** (108 mg, 0.13 mmol, 1.3 equiv). Purification by flash column chromatography (hexane:DCM = 4:1, followed by hexane:ethyl acetate = 95:5, Rf = 0.45) afforded 14.6 mg (80%) of **12b** as a white solid.

^{1}H NMR (600 MHz, $CDCl_3$) δ 7.67 (s, 1H), 7.50 (d, J = 7.8 Hz, 1H), 7.27 (d, J = 8.7 Hz, 1H), 7.14 (td, J = 7.4, 1.2 Hz, 1H), 7.09 (td, J = 7.5, 1.1 Hz, 1H), 6.26 (dt, J = 11.5, 1.7 Hz, 1H), 5.97 – 5.86 (m, 1H), 3.08 – 3.00 (m, 2H), 2.56 (q, J = 5.2 Hz, 2H), 2.10 – 2.01 (m, 2H).

^{13}C NMR (150 MHz, $CDCl_3$) δ 135.4, 132.7, 132.2, 129.4, 122.1, 120.3, 119.5, 118.4, 115.0, 110.6, 31.8, 26.0, 24.3.

HRMS (ESI) calculated for $[C_{13}H_{13}N+H]^+$ 184.1121, found:184.1143

3,3-dimethyl-4,9-dihydro-3*H*-carbazole (12c)

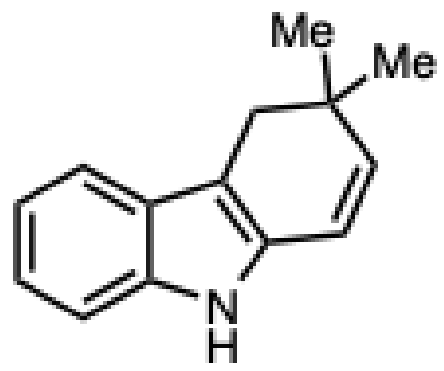

The compound was synthesized according to the **general procedure A**, employing 4,4-dimethylcyclohex-2-en-1-one (12.4 mg, 0.10 mmol, 1.0 equiv), LiHMDS (0.11 mL, 1.0 M THF, 0.11 mmol, 1.1 equiv), and aryl-tBuBisTrip **2** (108 mg, 0.13 mmol, 1.3 equiv). Purification by flash column chromatography (hexane:DCM = 4:1, followed by hexane:ethyl acetate = 90:10, Rf = 0.45) afforded 13.0 mg (66%) of **12c** as a yellow solid.

^{1}H NMR (500 MHz, $CDCl_3$) δ 7.76 (s, 1H), 7.52 – 7.47 (m, 1H), 7.33 – 7.27 (m, 1H), 7.17 – 7.06 (m, 2H), 6.30 (d, *J* = 9.8 Hz, 1H), 5.71 (d, *J* = 9.8 Hz, 1H), 2.79 (s, 2H), 1.17 (s, 6H).

^{13}C NMR (126 MHz, $CDCl_3$) δ 139.9, 136.4, 132.3, 127.7, 121.4, 119.8, 118.3, 115.9, 111.1, 108.7, 34.5, 33.9, 28.8.

HRMS (ESI) calculated for $[C_{14}H_{15}N+H]^+$ 198.1277, found: 198.1275

2-((triisopropylsilyl)ethynyl)-4,9-dihydro-3*H*-carbazole (12d)

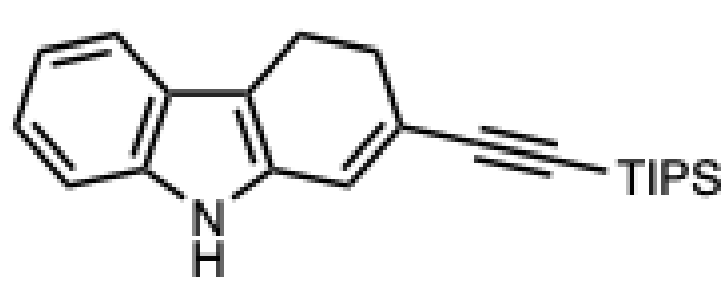

The compound was synthesized according to the **general procedure A**, employing 3-((triisopropylsilyl)ethynyl)cyclohex-2-en-1-one (27.7 mg, 0.10 mmol, 1.0 equiv), LiHMDS (0.11 mL, 1.0 M THF, 0.11 mmol, 1.1 equiv), and aryl-tBuBisTrip **2** (108 mg, 0.13 mmol, 1.3 equiv). Purification by flash column chromatography (hexane:DCM = 4:1, followed by hexane:ethyl acetate = 95:5, Rf = 0.35) afforded 15.7 mg (47%) of **12d** as a white solid.

^{1}H NMR (600 MHz, $CDCl_3$) δ 7.79 (s, 1H), 7.47 (d, *J* = 7.7 Hz, 1H), 7.30 (d, *J* = 8.0 Hz, 1H), 7.16 – 7.07 (m, 2H), 6.75 (s, 1H), 2.94 (t, *J* = 9.1 Hz, 2H), 2.65 (t, *J* = 9.1 Hz, 2H), 1.12 (s, 21H).

^{13}C NMR (150 MHz, $CDCl_3$) δ 136.9, 133.1, 126.9, 124.0, 122.3, 120.2, 119.9, 118.8, 111.2, 110.8, 109.0, 93.6, 29.4, 19.5, 18.9, 11.5.

HRMS (ESI) calculated for $[C_{23}H_{31}NSi+H]^+$ 350.2299, found: 350.2284

(*E*)-2-styryl-1*H*-indole (12e)

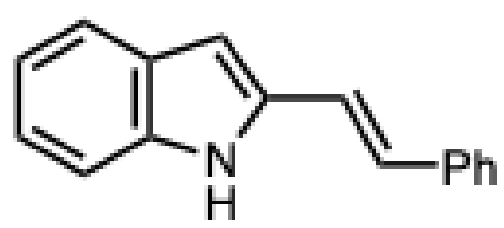

The compound was synthesized according to the **general procedure A**, employing (*E*)-4-phenylbut-3-en-2-one (14.6 mg, 0.10 mmol, 1.0 equiv), LiHMDS (0.11 mL, 1.0 M THF, 0.11 mmol, 1.1 equiv), and aryl-tBuBisTrip **2** (108 mg, 0.13 mmol, 1.3 equiv). Purification by flash column chromatography (hexane:DCM = 4:1, followed by hexane:acetone = 95:5, Rf = 0.3) afforded 8.6 mg (39%) of **12e** as a white solid. The compound was fully consistent with literature data [20].

^{1}H NMR (600 MHz, $CDCl_3$) δ 8.25 (s, 1H), 7.58 (d, J = 7.9 Hz, 1H), 7.53 – 7.48 (m, 2H), 7.40 – 7.33 (m, 3H), 7.28 (d, J = 7.4 Hz, 1H), 7.19 (ddd, J = 8.2, 7.0, 1.2 Hz, 1H), 7.15 – 7.08 (m, 2H), 6.91 (d, J = 16.5 Hz, 1H), 6.64 – 6.60 (m, 1H).

^{13}C NMR (150 MHz, $CDCl_3$) δ 137.1, 137.0, 136.4, 129.1, 128.9, 127.9, 127.3, 126.4, 123.1, 120.8, 120.4, 119.2, 110.7, 104.1.

HRMS (ESI) calculated for $[C_{16}H_{13}N+H]^+$ 220.1121, found: 220.1121

(*S*)-1-methyl-4-(prop-1-en-2-yl)-4,9-dihydro-3*H*-carbazole (12f)

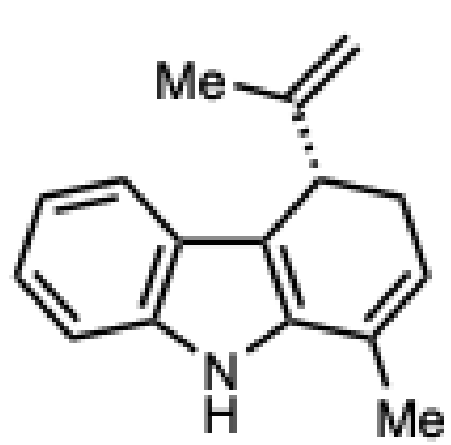

The compound was synthesized according to the **general procedure A**, employing *R*-carvone (15.0 mg, 0.10 mmol, 1.0 equiv), LiHMDS (0.11 mL, 1.0 M THF, 0.11 mmol, 1.1 equiv), and aryl-

tBuBisTrip **2** (108 mg, 0.13 mmol, 1.3 equiv). Purification by flash column chromatography (hexane:DCM = 4:1, followed by hexane:ethyl acetate = 95:5, Rf = 0.3) afforded 13.4 mg (60%) of **12f** as a white solid.

^{1}H NMR (500 MHz, $CDCl_3$) δ 7.91 (s, 1H), 7.54 (d, *J* = 7.8 Hz, 1H), 7.33 (d, *J* = 8.0 Hz, 1H), 7.15 – 7.03 (m, 2H), 5.64 (q, *J* = 2.3 Hz, 1H), 4.88 – 4.77 (m, 2H), 3.85 (t, *J* = 8.8 Hz, 1H), 2.65 – 2.48 (m, 2H), 2.07 (d, *J* = 1.9 Hz, 3H), 1.80 (s, 3H).

^{13}C NMR (126 MHz, $CDCl_3$) δ 148.0, 135.9, 135.3, 127.3, 125.2, 122.9, 121.4, 120.0, 119.2, 111.2, 111.0, 110.5, 40.6, 30.7, 19.8, 17.3.

HRMS (ESI) calculated for $[C_{16}H_{17}N+H]^+$ 224.1434, found: 224.1432

3-(2-(4-(benzhydryloxy)piperidin-1-yl)ethyl)-2-(4-(*tert*-butyl)phenyl)-1*H*-indole (13)

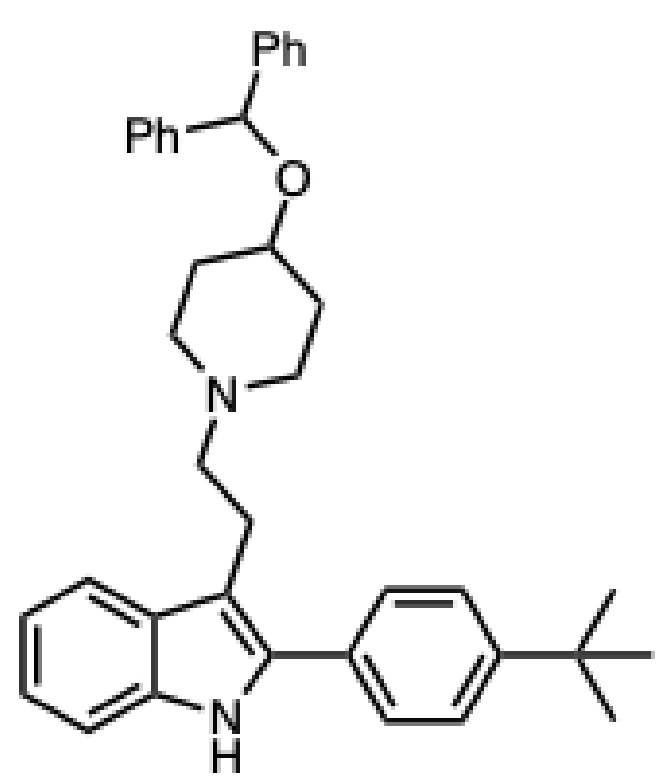

The compound was synthesized according to the **general procedure A**, employing ebastine (47.0 mg, 0.10 mmol, 1.0 equiv), LiHMDS (0.11 mL, 1.0 M THF, 0.11 mmol, 1.1 equiv), and aryl-tBuBisTrip **2** (108 mg, 0.13 mmol, 1.3 equiv). Purification by flash column chromatography (hexane:DCM = 4:1, followed by hexane:acetone = 80:20 to 40:60, Rf = 0.45 in 40:60) afforded 29.0 mg (53%) of **13** as a yellow solid.

^{1}H NMR (600 MHz, $CDCl_3$) δ 8.04 (s, 1H), 7.64 (d, *J* = 7.8 Hz, 1H), 7.52 – 7.47 (m, 4H), 7.39 – 7.30 (m, 10H), 7.26 – 7.24 (m, 1H), 7.20 (t, *J* = 7.5 Hz, 1H), 7.13 (t, *J* = 7.5 Hz, 1H), 5.54 (s, 1H), 3.55 – 3.44 (m, 1H), 3.11 (t, *J* = 8.3 Hz, 2H), 2.98 – 2.84 (m, 2H), 2.72 (t, *J* = 8.4 Hz, 2H), 2.42 – 2.21 (m, 2H), 2.00 – 1.91 (m, 2H), 1.85 – 1.77 (m, 2H), 1.38 (s, 9H).

^{13}C NMR (150 MHz, $CDCl_3$) δ 150.9, 143.0, 136.0, 134.9, 130.3, 129.4, 128.5, 127.6, 127.5, 127.3, 126.0, 122.3, 119.7, 119.1, 110.9, 80.2, 59.4, 51.3, 34.8, 31.5, 29.9, 29.4, 22.7.

HRMS (ESI) calculated for $[C_{38}H_{42}N_2O]^+$ 542.3297, found: 542.3360

(9R,10aS,11S)-10a,11-dimethyl-9-(prop-1-en-2-yl)-7,8,9,10,10a,11-hexahydro-5H-benzo[b]carbazole (14)

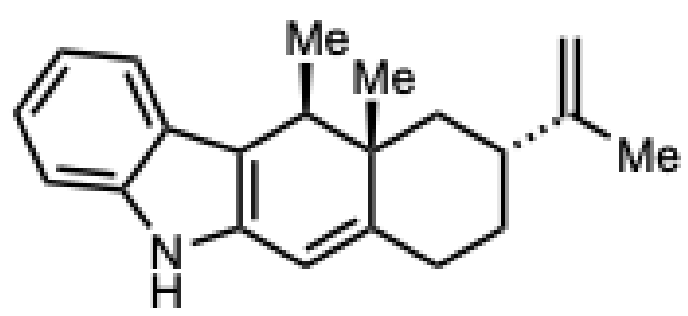

The compound was synthesized according to the **general procedure A**, employing (+)-nootkatone (21.8 mg, 0.10 mmol, 1.0 equiv), LiHMDS (0.11 mL, 1.0 M THF, 0.11 mmol, 1.1 equiv), and aryl-tBuBisTrip **2** (108 mg, 0.13 mmol, 1.3 equiv). Purification by flash column chromatography (hexane:DCM = 4:1, followed by hexane:ethyl acetate = 95:5, Rf = 0.45) afforded 21.3 mg (73%) of **14** as a white solid.

^{1}H NMR (500 MHz, $CDCl_3$) δ 7.79 – 7.74 (m, 1H), 7.72 (s, 1H), 7.32 – 7.26 (m, 1H), 7.11 – 7.02 (m, 2H), 6.08 (d, *J* = 2.3 Hz, 1H), 4.82 – 4.73 (m, 2H), 3.08 (q, *J* = 7.2 Hz, 1H), 2.54 – 2.40 (m, 2H), 2.25 (tt, *J* = 12.4, 3.1 Hz, 1H), 2.10 (dt, *J* = 12.9, 2.8 Hz, 1H), 1.90 – 1.83 (m, 1H), 1.80 (s, 3H), 1.64 (d, *J* = 7.2 Hz, 3H), 1.45 – 1.25 (m, 4H), 0.93 (s, 3H).

^{13}C NMR (126 MHz, $CDCl_3$) δ 150.4, 147.9, 136.3, 133.0, 127.8, 120.4, 119.7, 119.5, 112.2, 111.1, 111.1, 108.9, 45.0, 42.1, 41.7, 41.5, 32.5, 31.6, 21.1, 15.8, 12.5.

HRMS (ESI) calculated for $[C_{21}H_{25}N+H]^+$ 292.2060, found: 292.2056

(1*R*,3a*S*,3b*S*,12a*R*,12b*S*,14a*R*)-12a,14a-dimethyl-1-((*R*)-6-methylheptan-2-yl)-1,2,3,3a,3b,4,5,7,12,12a,12b,13,14,14a-tetradecahydrocyclopenta[5,6]naphtho[2,1-*b*]carbazole (15)

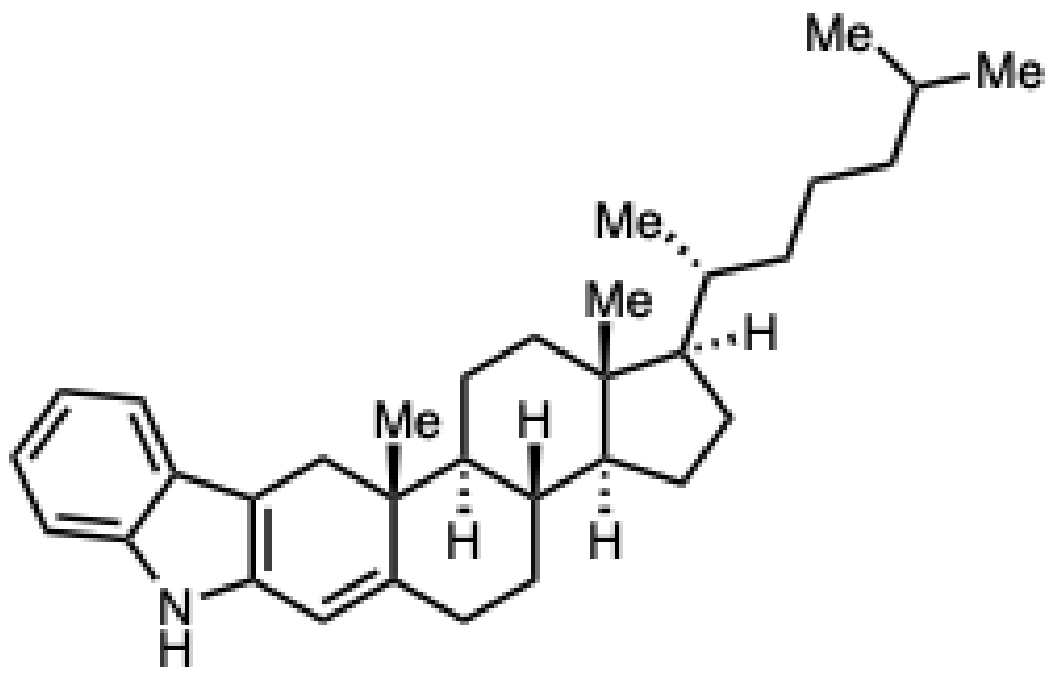

The compound was synthesized according to the **general procedure A**, employing cholest-4-en-3-one (38.4 mg, 0.10 mmol, 1.0 equiv), LDA (0.11 mL, 1.0 M THF/hexanes, 0.11 mmol, 1.1 equiv), and aryl-tBuBisTrip **2** (108 mg, 0.13 mmol, 1.3 equiv). Purification by flash column chromatography (hexane:DCM = 4:1, followed by hexane:ethyl acetate = 95:5, Rf = 0.4) afforded 23.2 mg (51%) of **15** as a slightly yellow solid.

^{1}H NMR (500 MHz, $CDCl_3$) δ 7.66 (s, 1H), 7.47 – 7.42 (m, 1H), 7.27 (d, *J* = 5.4 Hz, 1H), 7.11 – 7.05 (m, 2H), 6.02 (d, *J* = 2.4 Hz, 1H), 3.01 (d, *J* = 15.6 Hz, 1H), 2.62 (d, *J* = 15.6 Hz, 1H), 2.50 – 2.41 (m, 1H), 2.38 – 2.31 (m, 1H), 2.08 (dt, *J* = 12.7, 3.5 Hz, 1H), 1.92 – 1.82 (m, 1H), 1.79 (dt, *J* = 12.5, 2.5 Hz, 1H), 1.70 – 1.72 (m, 2H), 1.55 – 1.44 (m, 3H), 1.43 – 1.34 (m, 3H), 1.32 – 1.24 (m, 3H), 1.22 – 1.07 (m, 8H), 1.03 (s, 3H), 0.95 (d, *J* = 6.5 Hz, 3H), 0.88 (dd, *J* = 6.6, 2.2 Hz, 6H), 0.74 (s, 3H).

^{13}C NMR (126 MHz, $CDCl_3$) δ 149.0, 136.2, 132.7, 128.0, 120.7, 119.6, 117.8, 111.3, 110.9, 106.9, 56.4, 56.2, 54.8, 42.7, 40.6, 40.2, 39.7, 36.7, 36.3, 36.0, 35.0, 32.5, 31.8, 28.4, 28.2, 24.6, 24.0, 23.0, 22.7, 21.9, 18.9, 18.8, 12.1.

HRMS (ESI) calculated for $[C_{33}H_{47}N]^+$ 457.3709, found: 457.3530

Methyl (2*S*,4a*S*,6a*S*,6b*R*,8a*R*,15a*S*,15b*R*,17b*R*)-2,4a,6a,6b,9,9,15a-heptamethyl-16-oxo-2,3,4,4a,5,6,6a,6b,7,8,8a,9,10,15,15a,15b,16,17b-octadecahydro-1*H*-chryseno[2,1*b*]carbazole -2-carboxylate (16)

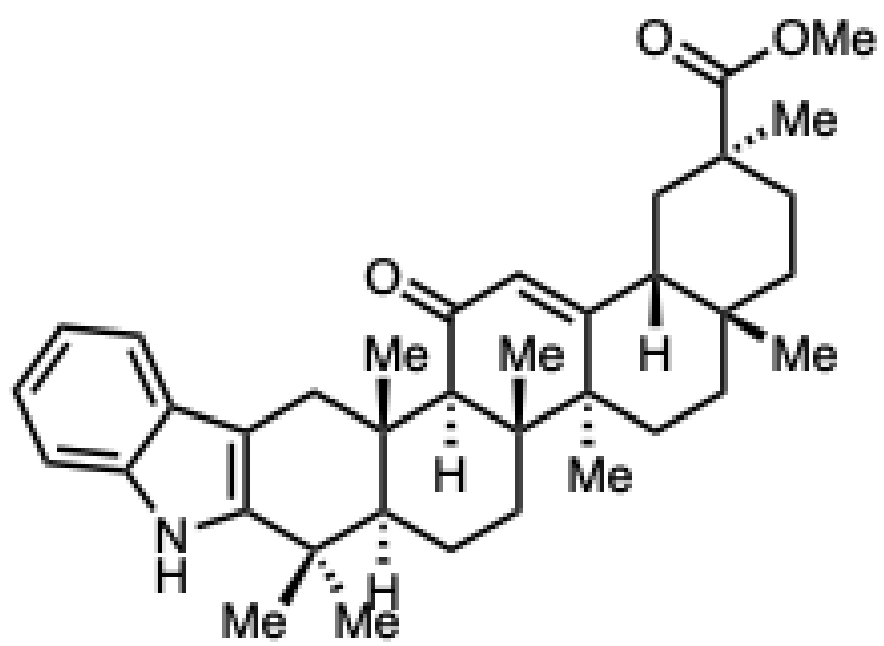

The compound was synthesized according to the **general procedure A**, employing methyl (2*S*,4a*S*,6a*S*,6b*R*,8a*R*,12a*S*,12b*R*,14b*R*)-2,4a,6a,6b,9,9,12a-heptamethyl-10,13-dioxo-1,2,3,4,4a,5,6,6a,6b,7,8,8a,9,10,11,12,12a,12b,13,14b-icosahydropicene-2-carboxylate (48.2 mg, 0.10 mmol, 1.0 equiv), LDA (0.11 mL, 1.0 M THF/hexanes, 0.11 mmol, 1.1 equiv), and aryl-tBuBisTrip **2** (108 mg, 0.13 mmol, 1.3 equiv). Purification by flash column chromatography (hexane:DCM = 4:1, followed by hexane:ethyl acetate = 85:15, Rf = 0.3) afforded 39.1 mg (70%) of **16** as a white solid.

^{1}H NMR (600 MHz, $CDCl_3$) δ 7.77 (s, 1H), 7.50 (d, *J* = 7.7 Hz, 1H), 7.29 (d, *J* = 7.9 Hz, 1H), 7.11 (t, *J* = 7.5 Hz, 1H), 7.06 (t, *J* = 7.4 Hz, 1H), 5.78 (s, 1H), 3.97 (d, *J* = 15.5 Hz, 1H), 3.72 (s, 3H), 2.68 (s, 1H), 2.27 (d, *J* = 15.5 Hz, 1H), 2.15 (dd, *J* = 14.2, 3.9 Hz, 1H), 2.07 (dd, *J* = 13.6, 4.5 Hz, 1H), 2.03 – 1.99 (m, 1H), 1.97 (dt, *J* = 13.5, 3.6 Hz, 1H), 1.89 (td, *J* = 13.7, 4.6 Hz, 1H), 1.79 (td, *J* = 12.8, 3.9 Hz, 1H), 1.70 (dd, *J* = 14.5, 4.1 Hz, 1H), 1.67 – 1.63 (m, 1H), 1.62 – 1.58 (m, 1H), 1.53 (dt, *J* = 12.7, 3.3 Hz, 1H), 1.42 (s, 5H), 1.35 (d, *J* = 10.3 Hz, 2H), 1.33 (s, 3H), 1.27 – 1.24 (m, 4H), 1.23 (s, 3H), 1.19 (s, 3H), 1.17 (s, 3H), 1.07 – 1.03 (dt, *J* = 13.6, 2.4 Hz, 1H), 0.85 (s, 3H).

^{13}C NMR (150 MHz, $CDCl_3$) δ 200.0, 177.1, 169.4, 140.2, 136.4, 128.9, 128.4, 121.1, 119.0, 118.7, 110.3, 107.3, 60.7, 53.2, 52.0, 48.6, 45.5, 44.2, 43.5, 41.4, 38.2, 37.9, 37.9, 37.7, 34.2, 32.2, 32.0, 31.3, 31.2, 28.8, 28.5, 26.7, 26.6, 23.6, 23.4, 18.7, 18.5, 16.2.

HRMS (ESI) calculated for $[C_{37}H_{49}NO_3+H]^+$ 556.3785, found: 556.3779

(2S,4'R)-7-chloro-2',4,6-trimethoxy-4'-methyl-4',9'-dihydro-3H-spiro[benzofuran-2,3'-carbazol]-3-one (17)

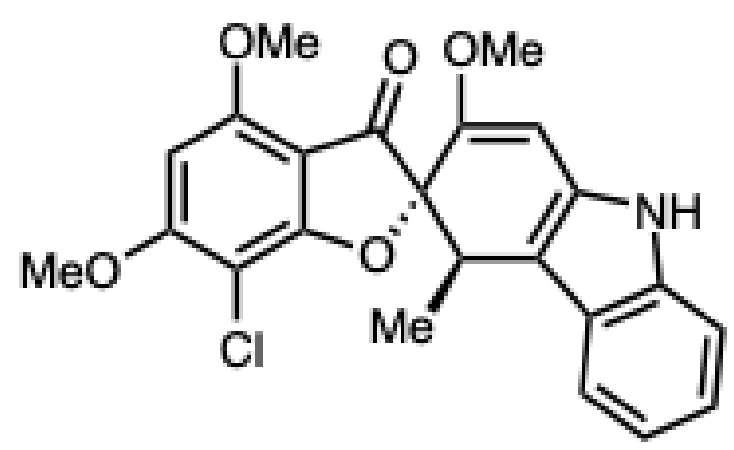

The compound was synthesized according to the **general procedure A**, employing griseofulvin (35.2 mg, 0.10 mmol, 1.0 equiv), LiHMDS (0.11 mL, 1.0 M THF, 0.11 mmol, 1.1 equiv), and aryl-tBuBisTrip **2** (108 mg, 0.13 mmol, 1.3 equiv). Purification by flash column chromatography (hexane:DCM = 4:1, followed by hexane:acetone =70:30, Rf = 0.2) afforded 10.6 mg (25%) of **17** as a yellow solid.

^{1}H NMR (500 MHz, $CDCl_3$) δ 7.91 (s, 1H), 7.48 – 7.42 (m, 1H), 7.30 – 7.27 (m, 1H), 7.09 – 7.02 (m, 2H), 6.10 (s, 1H), 5.83 (s, 1H), 3.98 (d, J = 11.2 Hz, 6H), 3.63 (q, J = 7.3 Hz, 1H), 3.62 (s, 3H), 1.41 (d, J = 7.2 Hz, 3H).

^{13}C NMR (126 MHz, $CDCl_3$) δ 193.8, 168.7, 164.2, 157.9, 154.5, 136.6, 130.8, 126.8, 120.7, 120.0, 117.9, 111.1, 107.4, 106.0, 97.5, 94.1, 93.9, 89.2, 57.0, 56.5, 56.0, 37.6, 15.1.

HRMS (ESI) calculated for $[C_{23}H_{20}ClNO_5+H]^+$ 426.1103, found: 426.1101

(S)-3-(tert-butyl)-2,3,4,9-tetrahydro-1H-carbazole (4d')

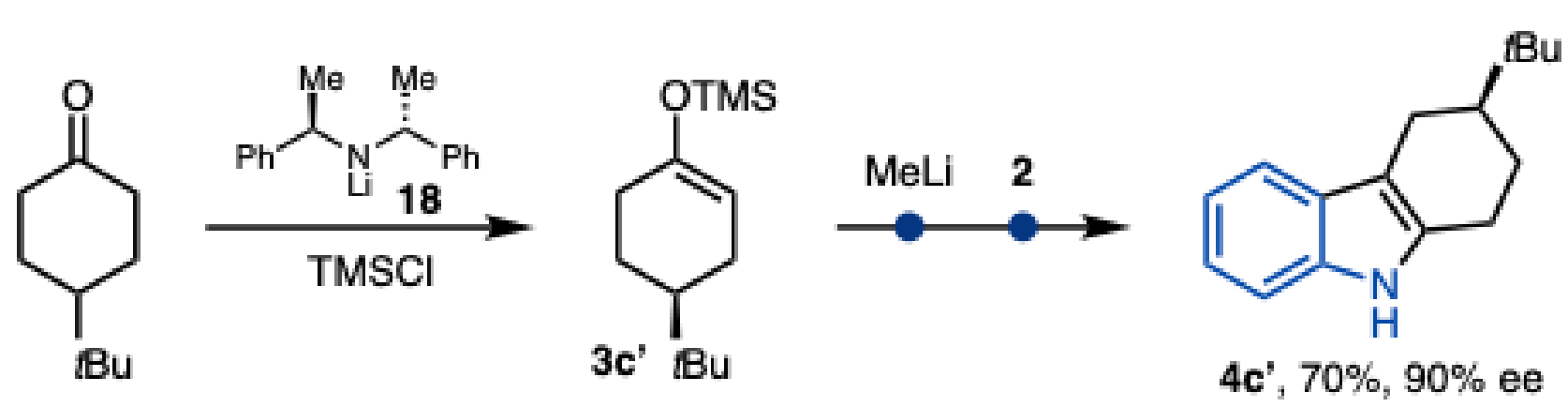

The compound was synthesized according to the **general procedure C**, employing (((S)-4-(tert-butyl)cyclohex-1-en-1-yl)oxy)trimethylsilane **3c'** (22.6 mg, 0.10 mmol, 1.0 equiv) (prepared according to previously reported procedure [21]), MeLi (70 µL, 1.6 M Et_2O, 0.11 mmol, 1.1 equiv), and aryl-tBuBisTrip **2** (108 mg, 0.13 mmol, 1.3 equiv). Purification by flash column chromatography (hexane:DCM = 4:1, followed by hexane:ethyl acetate = 95:5, Rf = 0.4) afforded 15.9 mg (70%) of **4c'** as a yellow solid. The product is spectroscopically identical to compound made using alternate procedure (see compound **4c**).

5. NMR Spectra

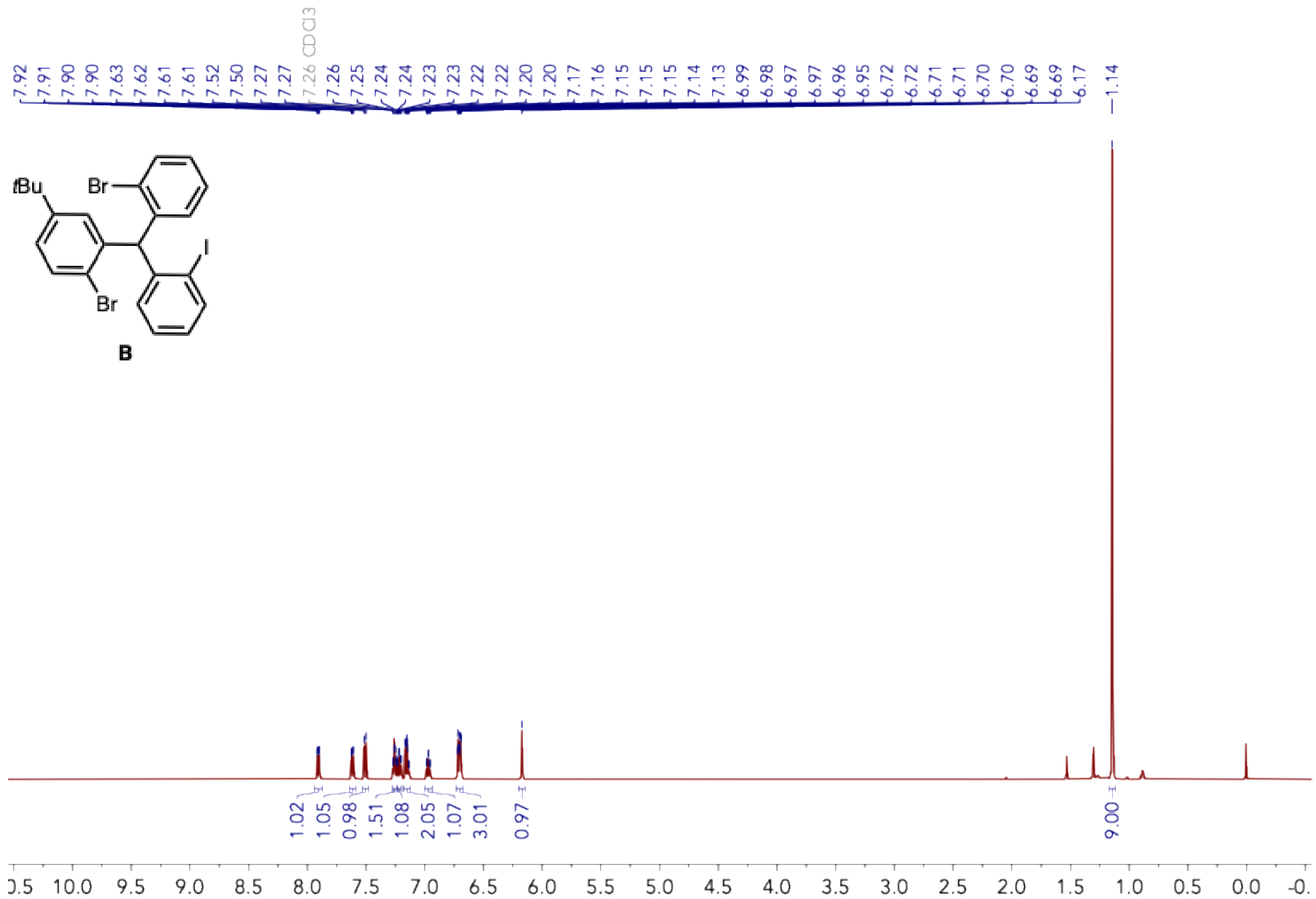

^{1}**H NMR** of **B** (500 MHz, $CDCl_3$)

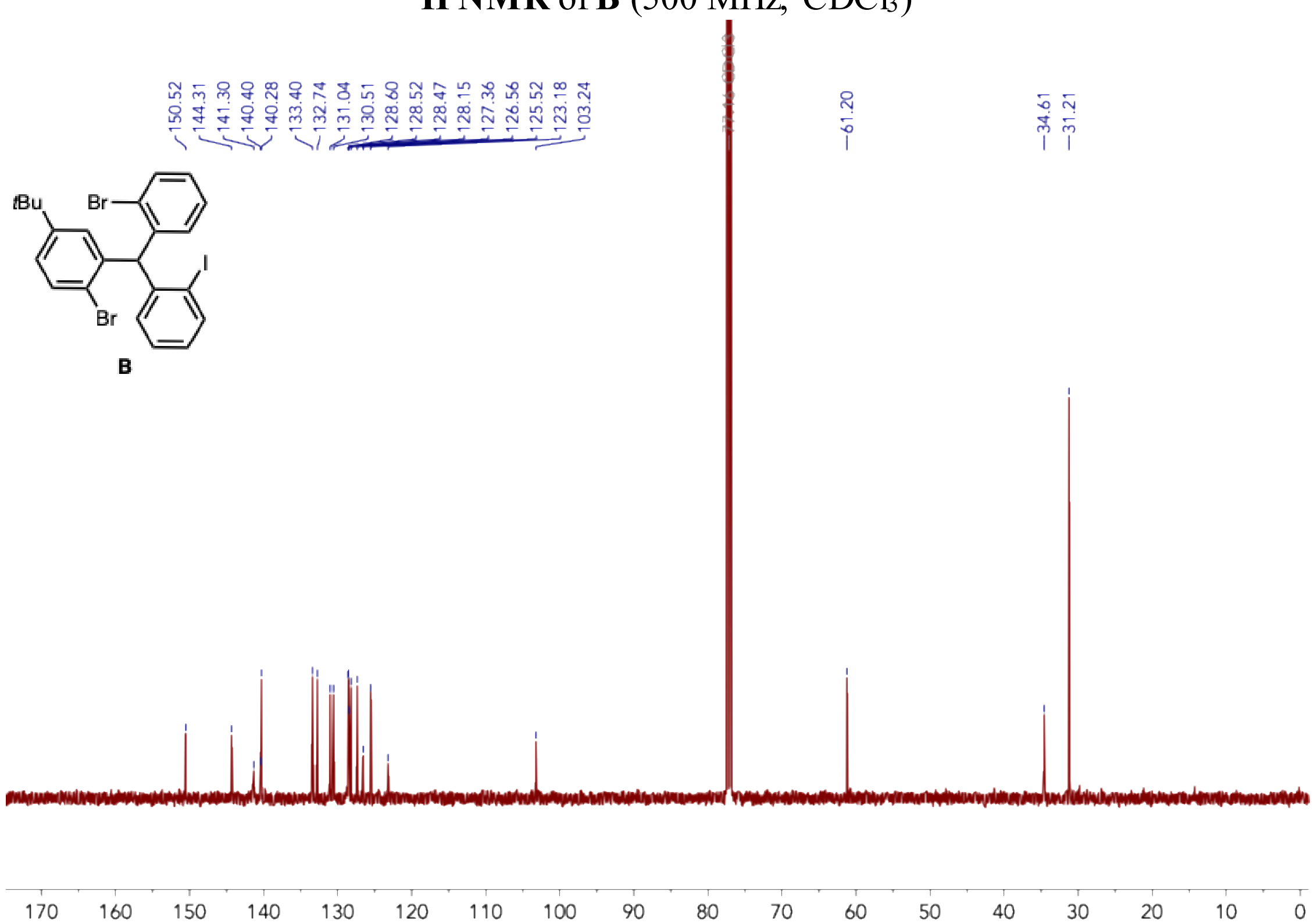

^{13}**C NMR** of **B** (126 MHz, $CDCl_3$)

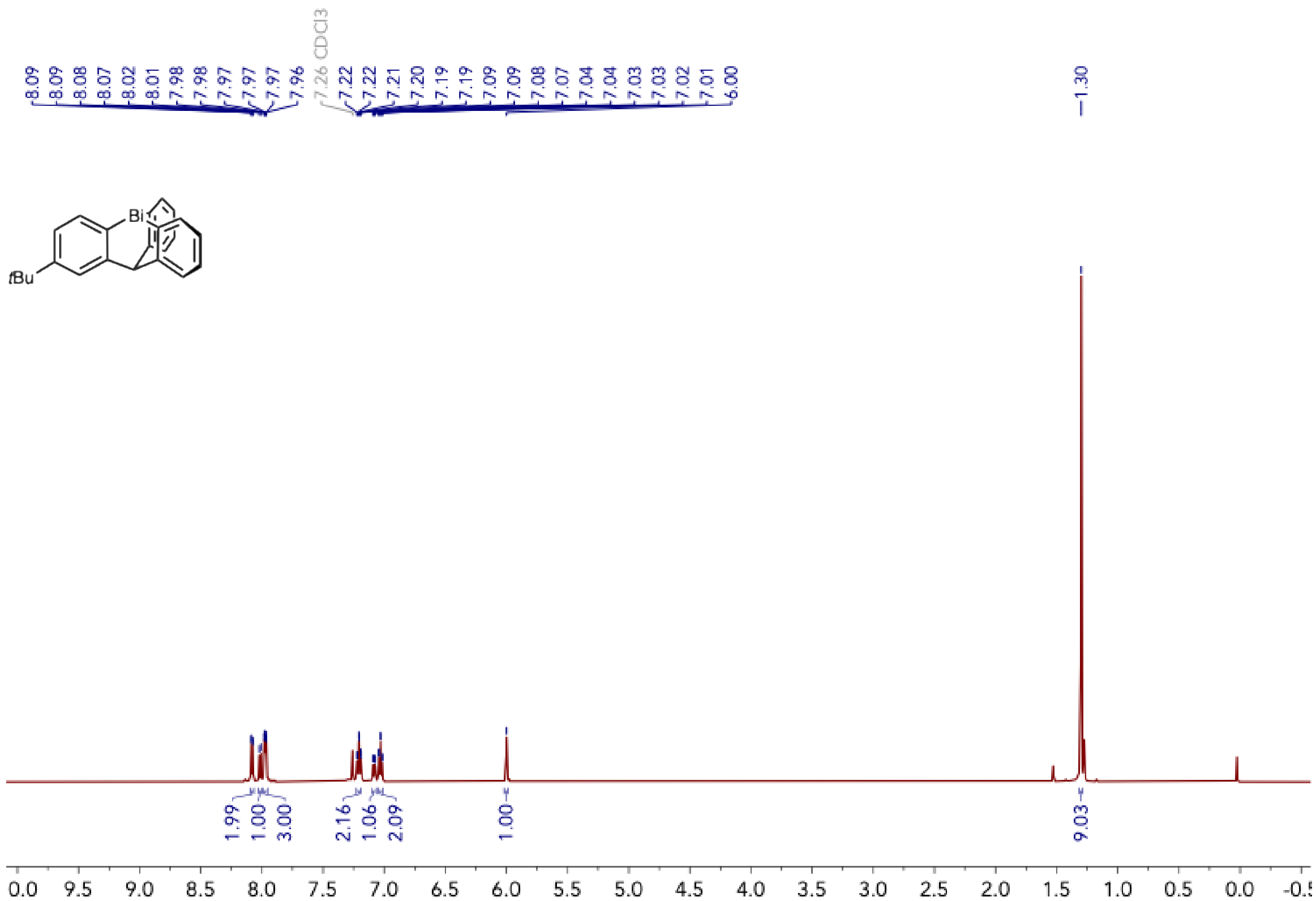

^{1}**H NMR** of **1** (500 MHz, $CDCl_3$)

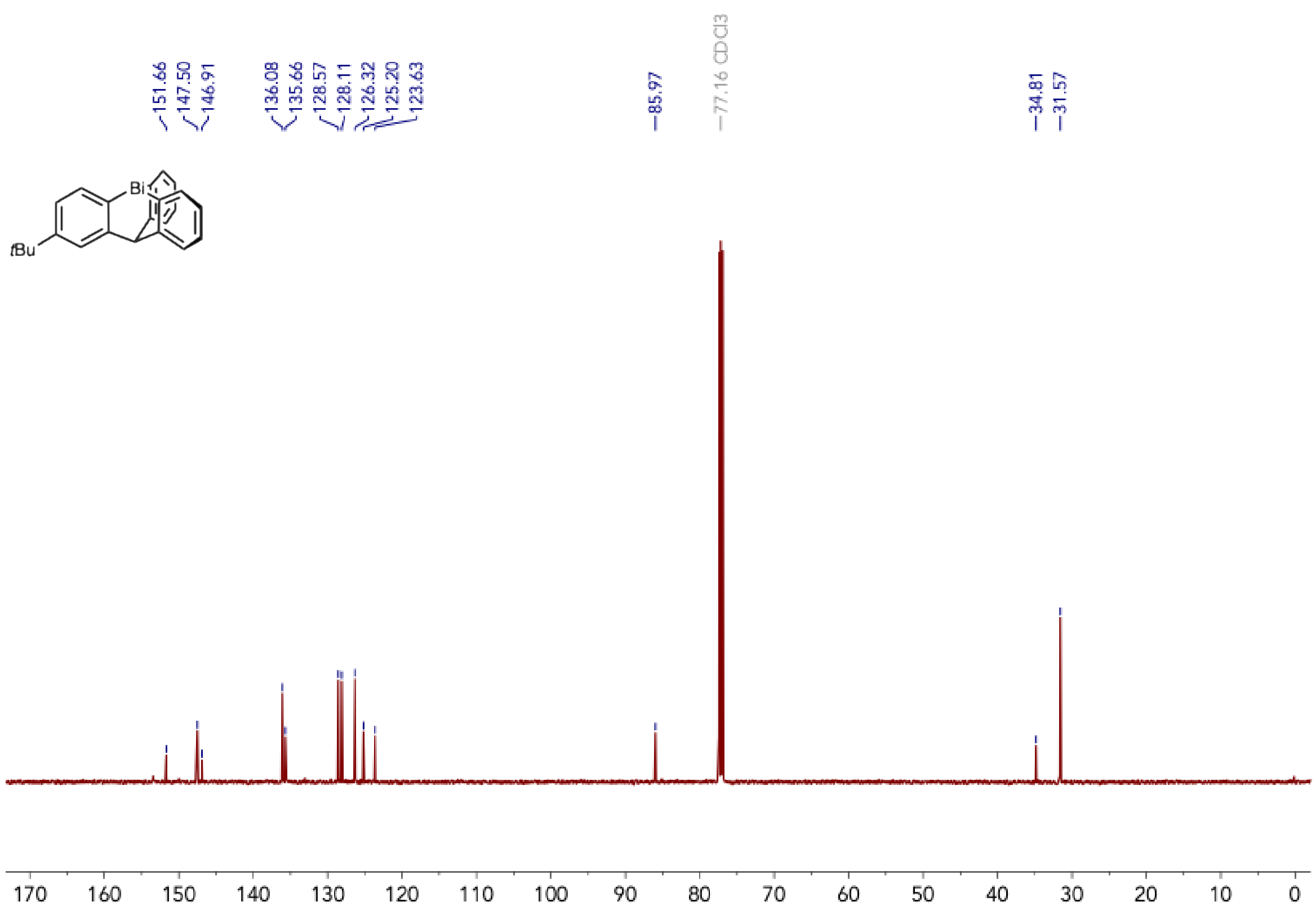

^{13}**C NMR** of **1** (126 MHz, $CDCl_3$)

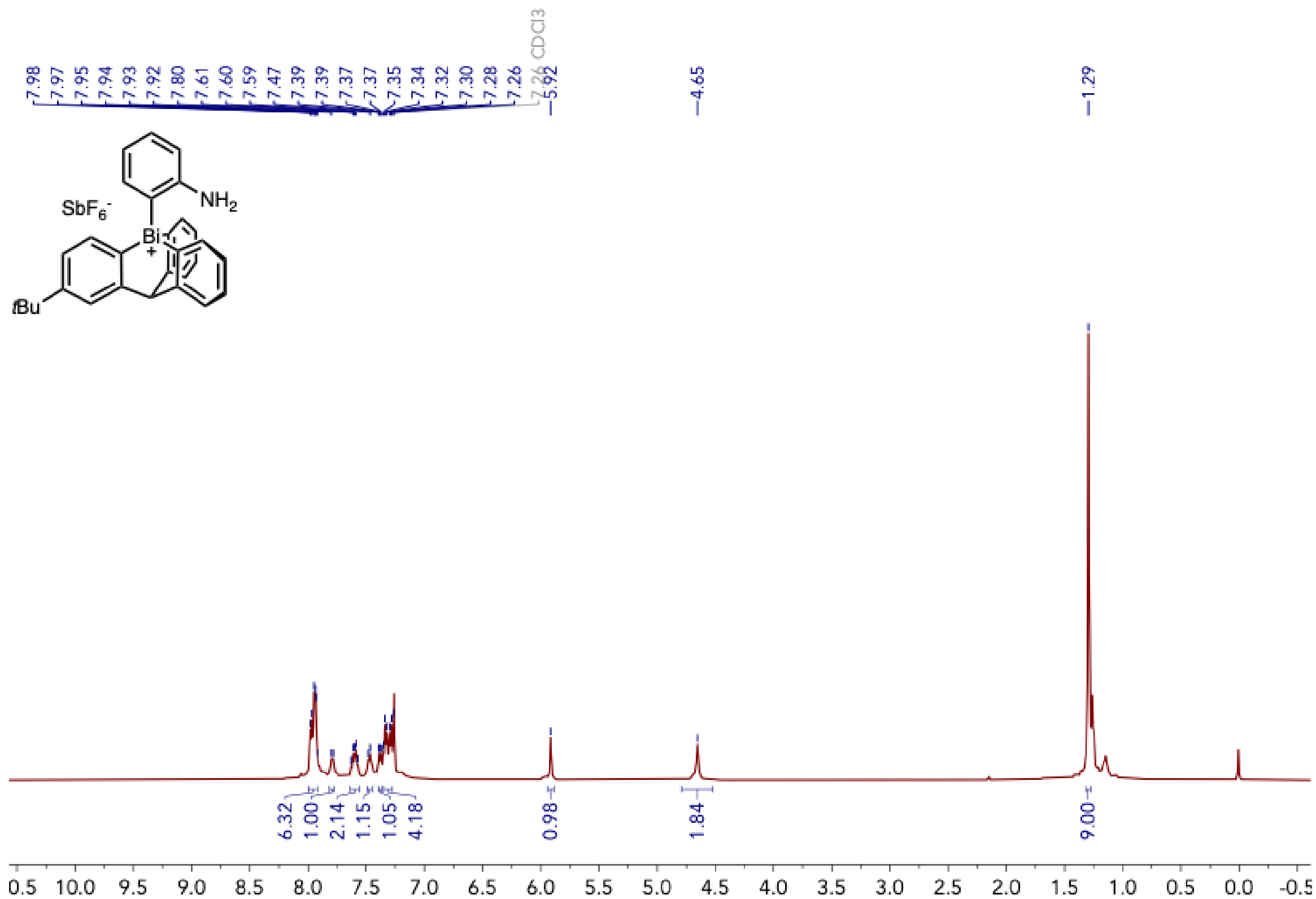

^{1}H NMR of **2** (500 MHz, $CDCl_3$)

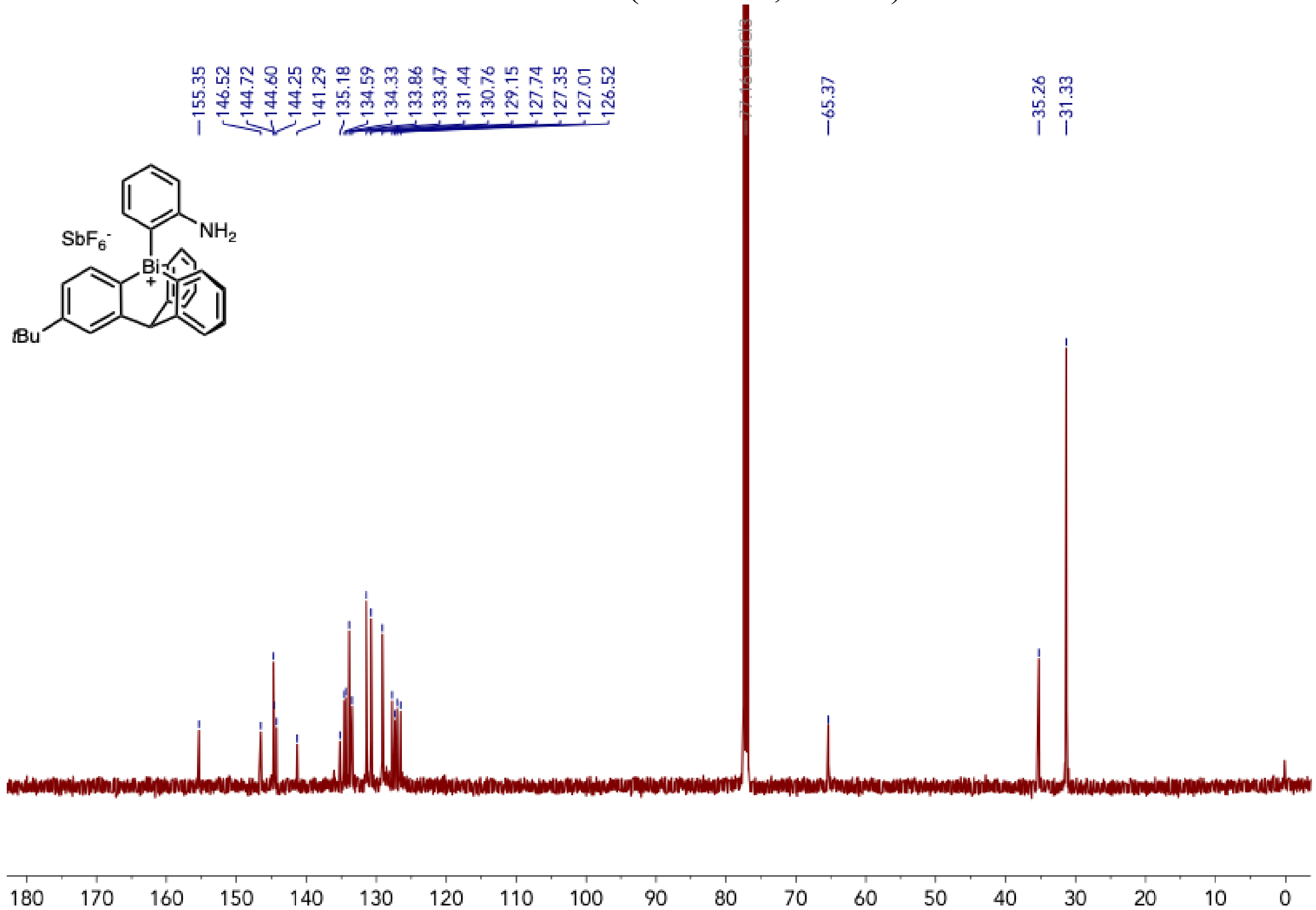

^{13}C NMR of **2** (126 MHz, $CDCl_3$)

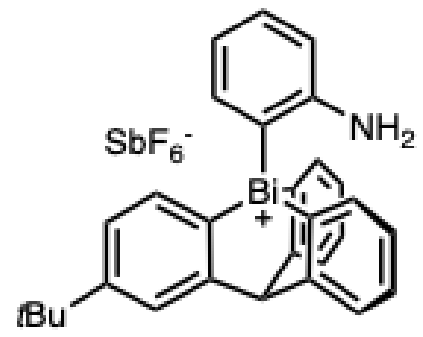

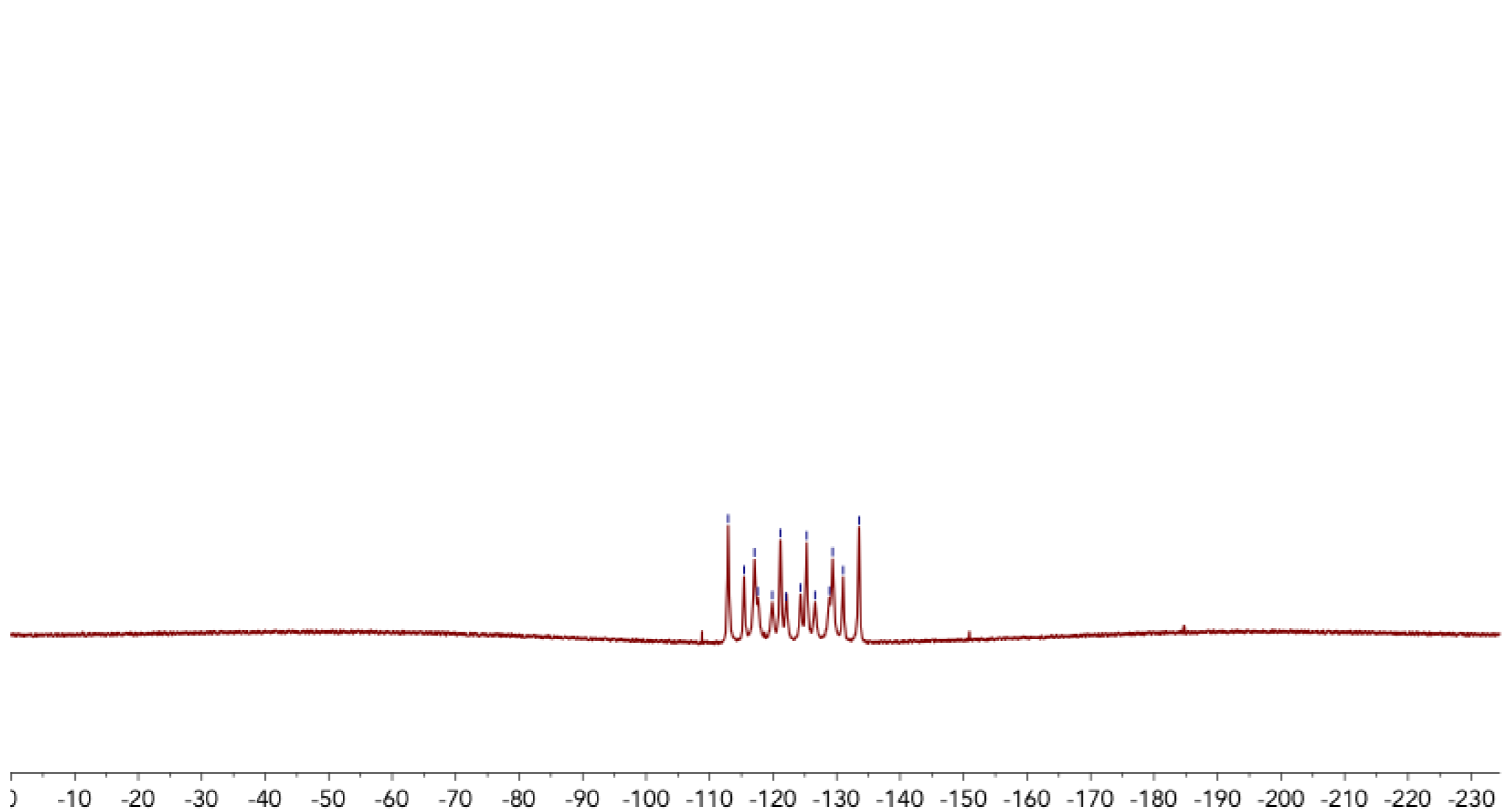

^{19}F NMR of **2** (470 MHz, Acetone-d_6)

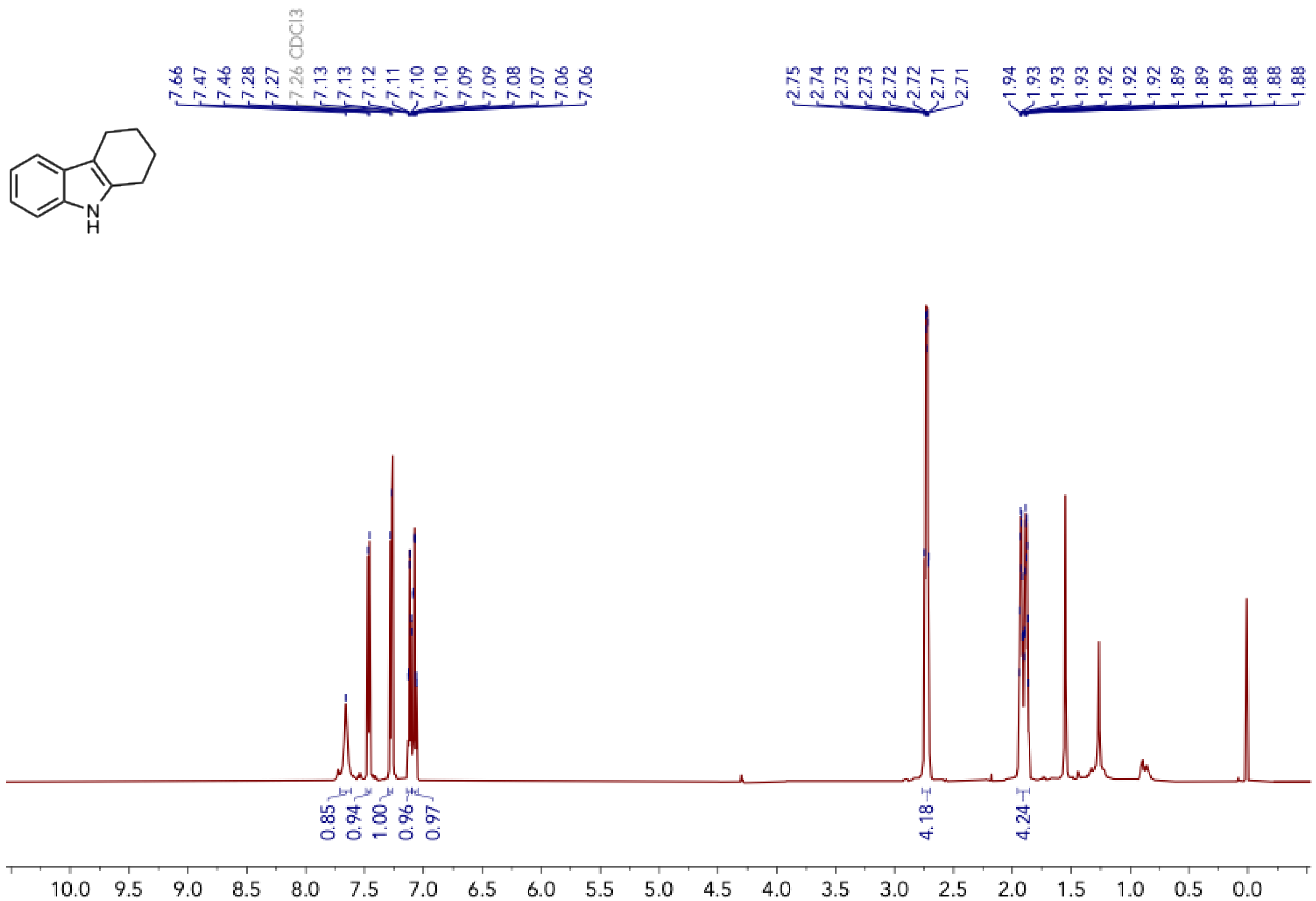

^{1}**H NMR** of **4a** (600 MHz, $CDCl_3$)

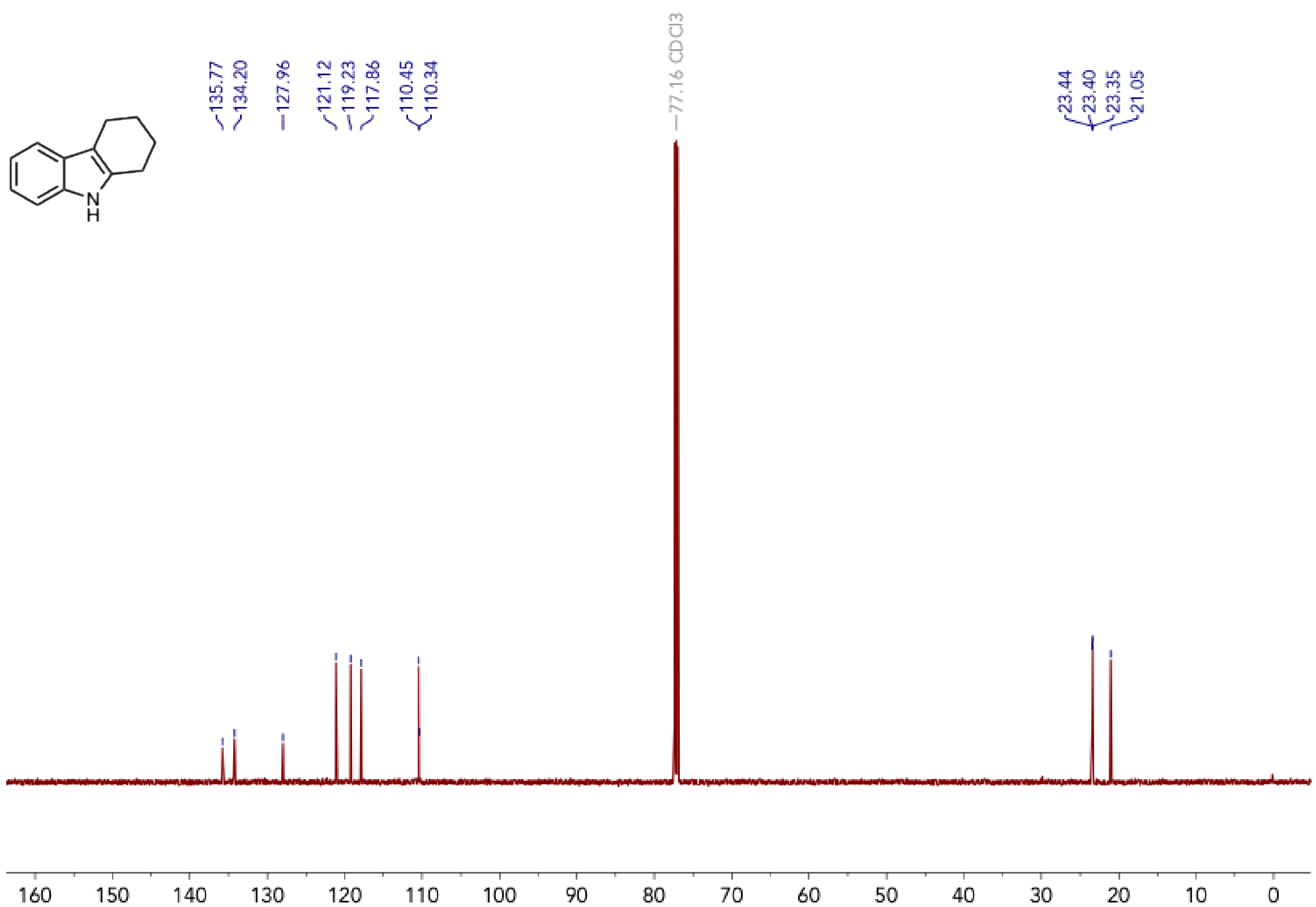

^{13}**C NMR** of **4a** (150 MHz, $CDCl_3$)

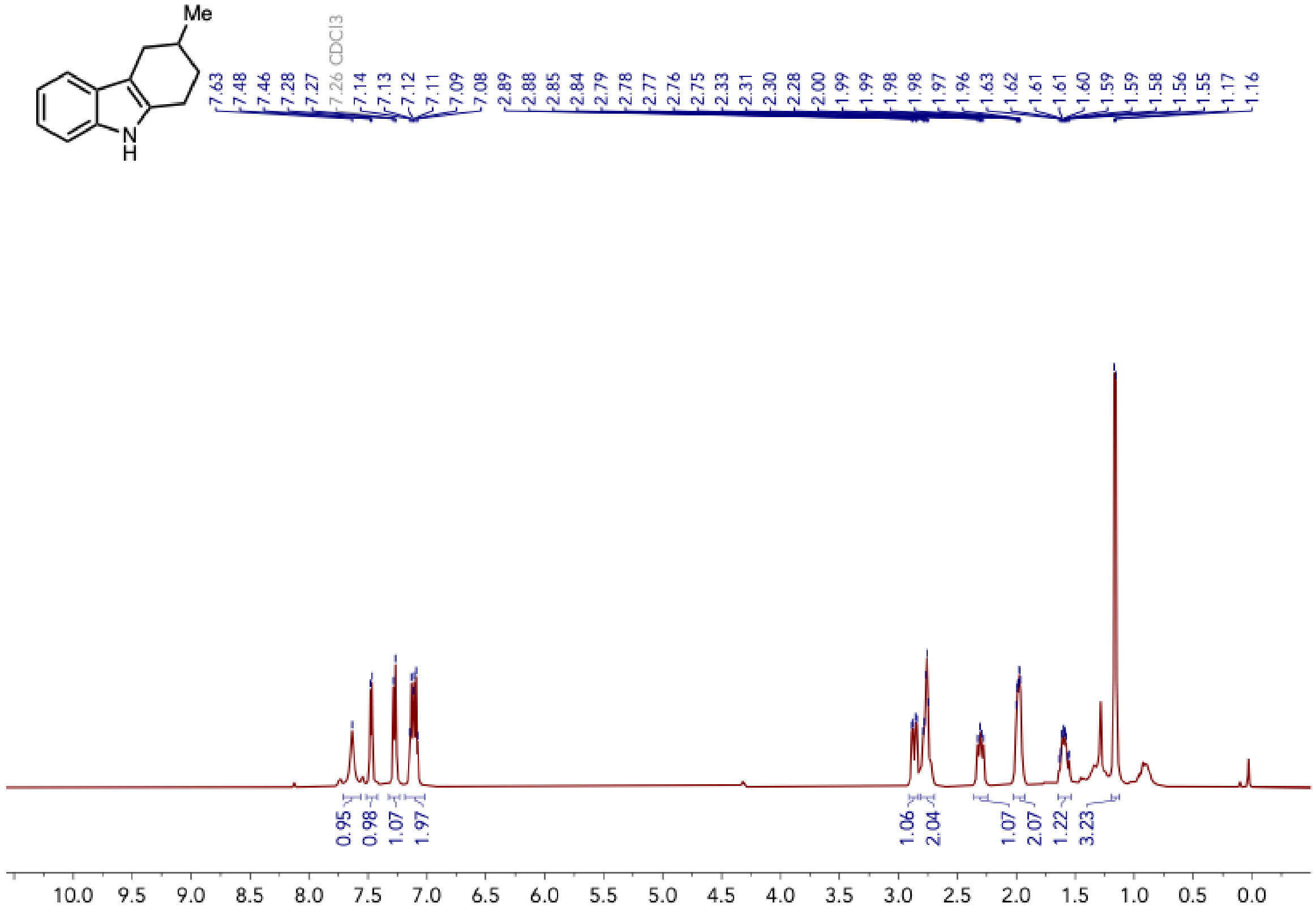

¹H NMR of **4b** (500 MHz, $CDCl_3$)

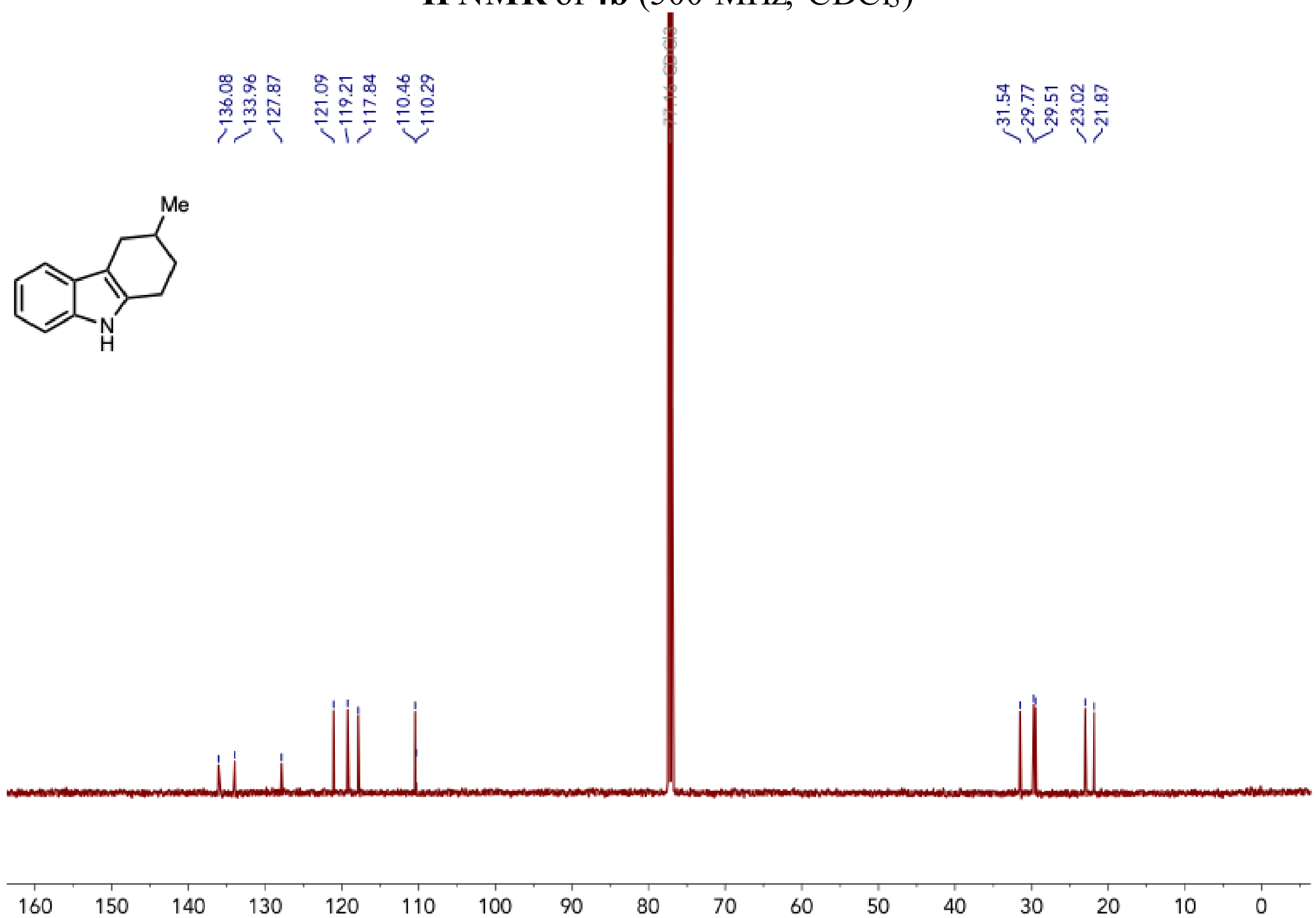

¹³C NMR of **4b** (126 MHz, $CDCl_3$)

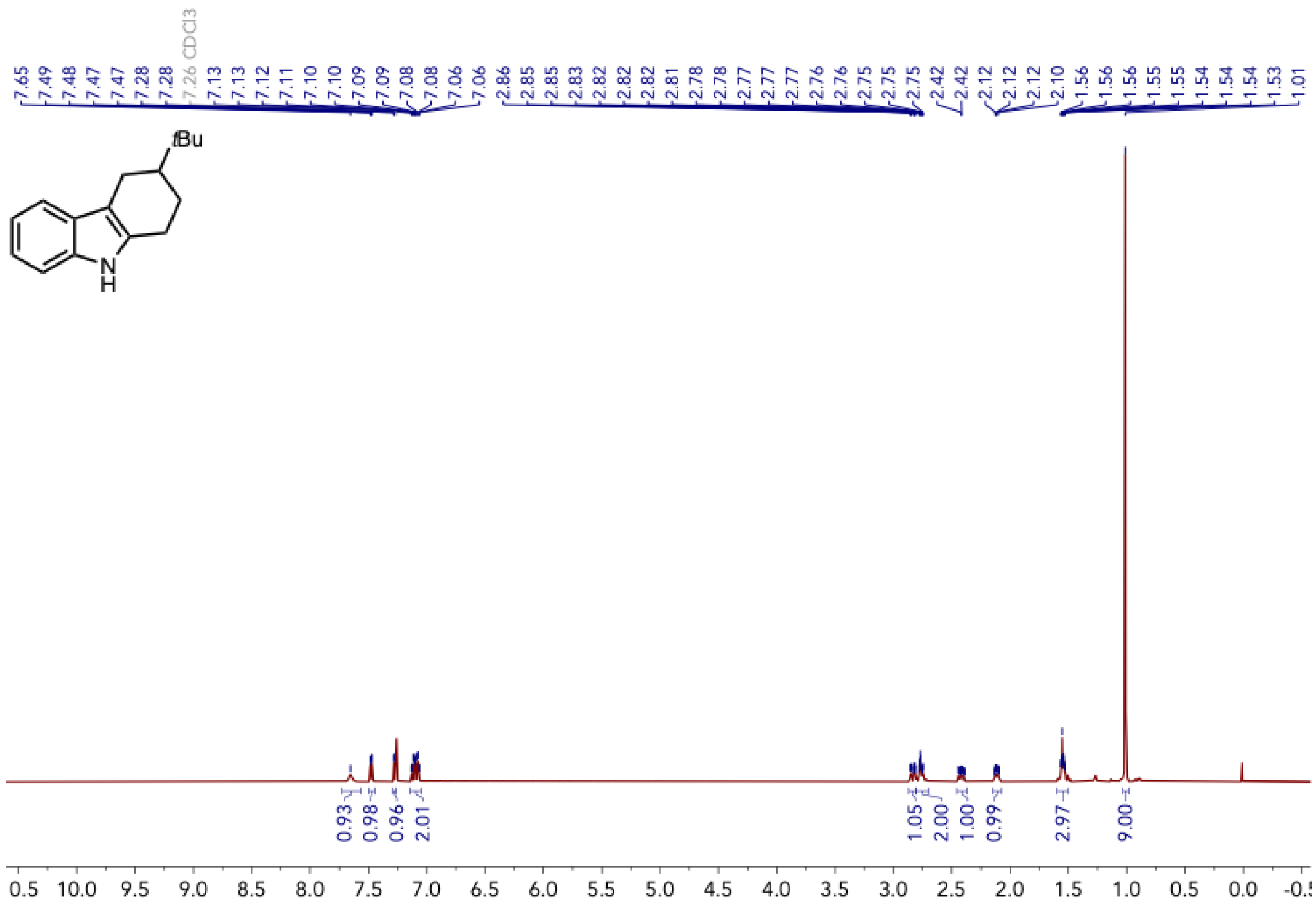

^{1}H NMR of **4c** (500 MHz, $CDCl_3$)

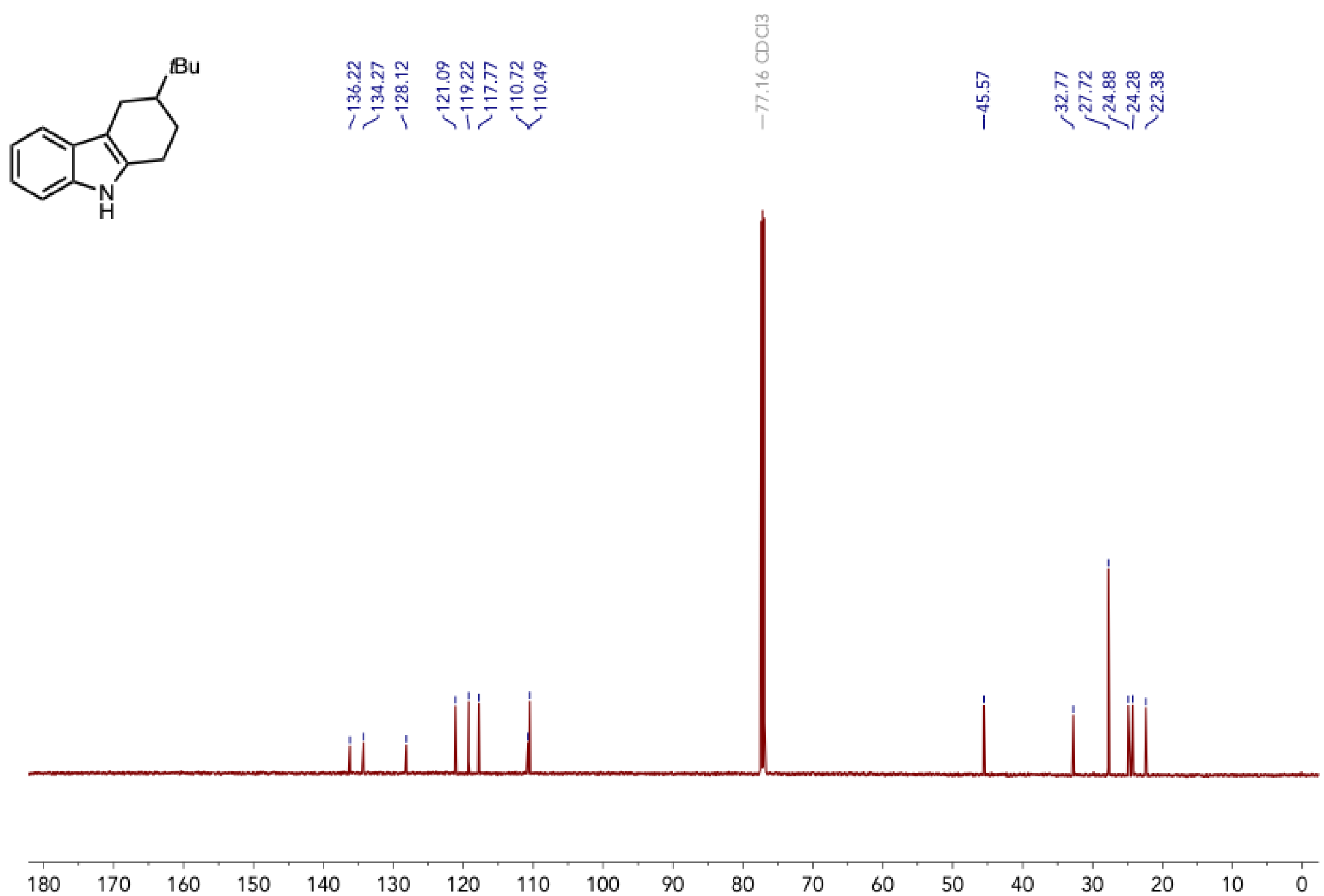

^{13}C NMR of **4c** (126 MHz, $CDCl_3$)

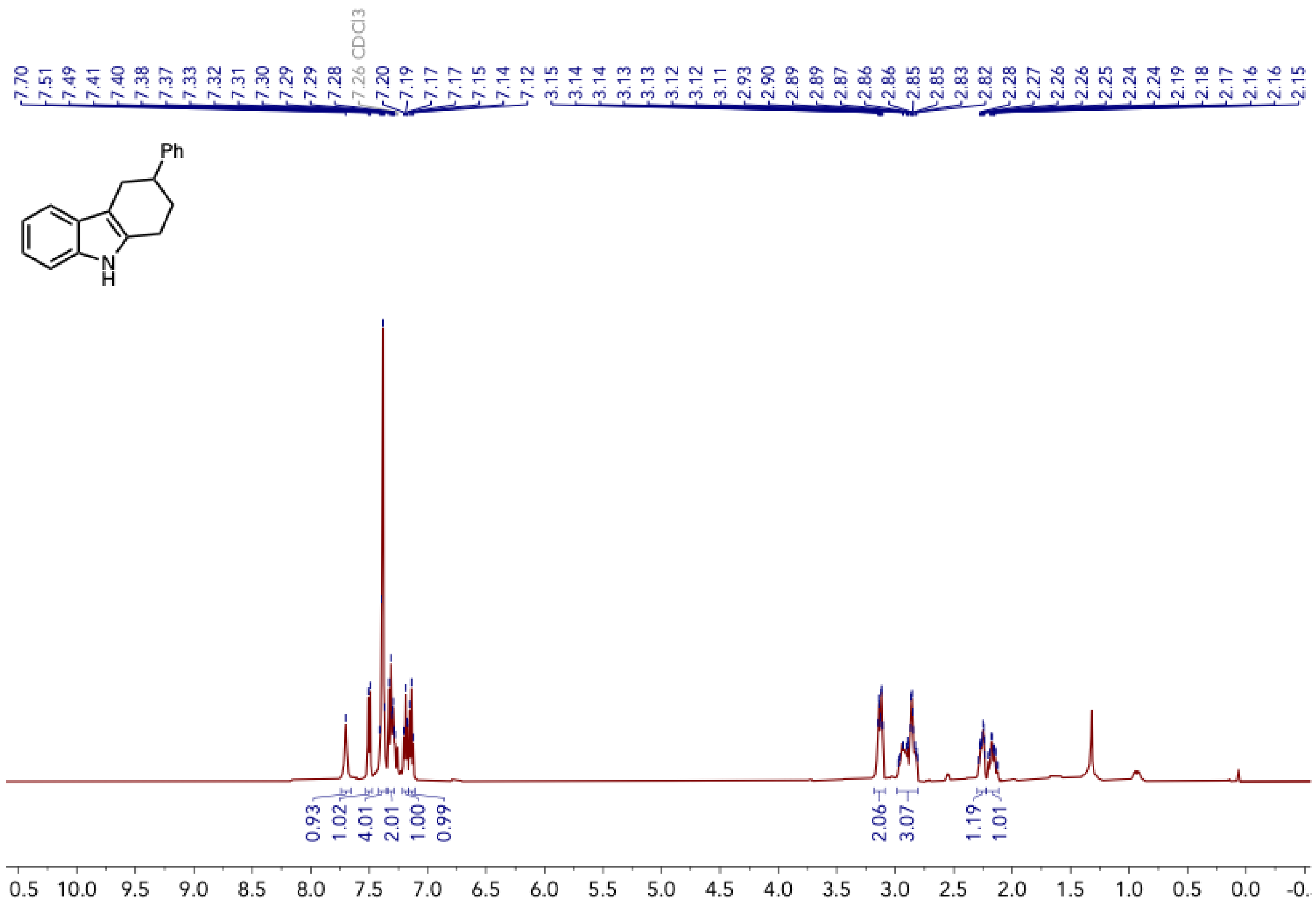

^{1}H NMR of **4d** (500 MHz, $CDCl_3$)

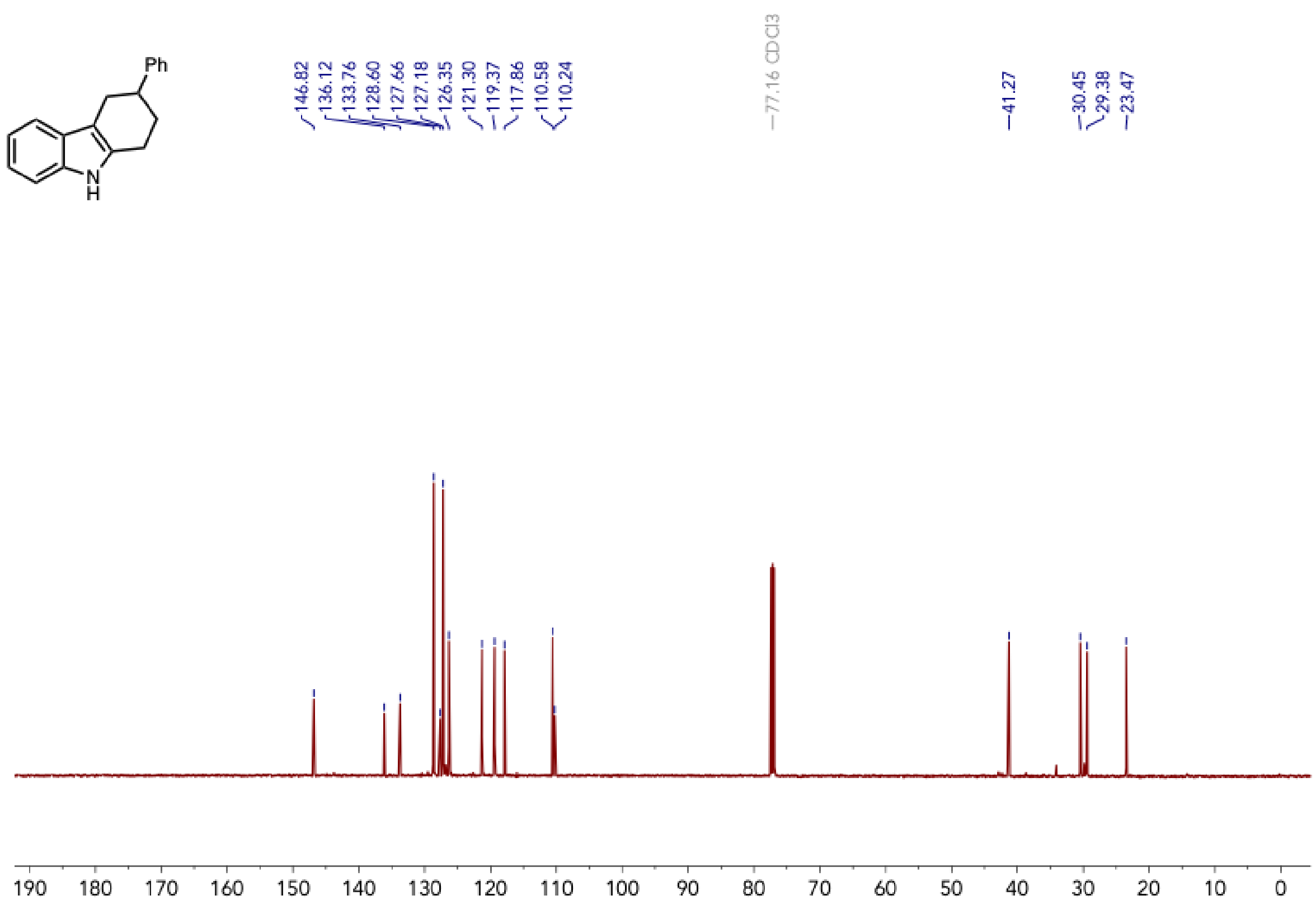

^{13}C NMR of **4d** (126 MHz, $CDCl_3$)

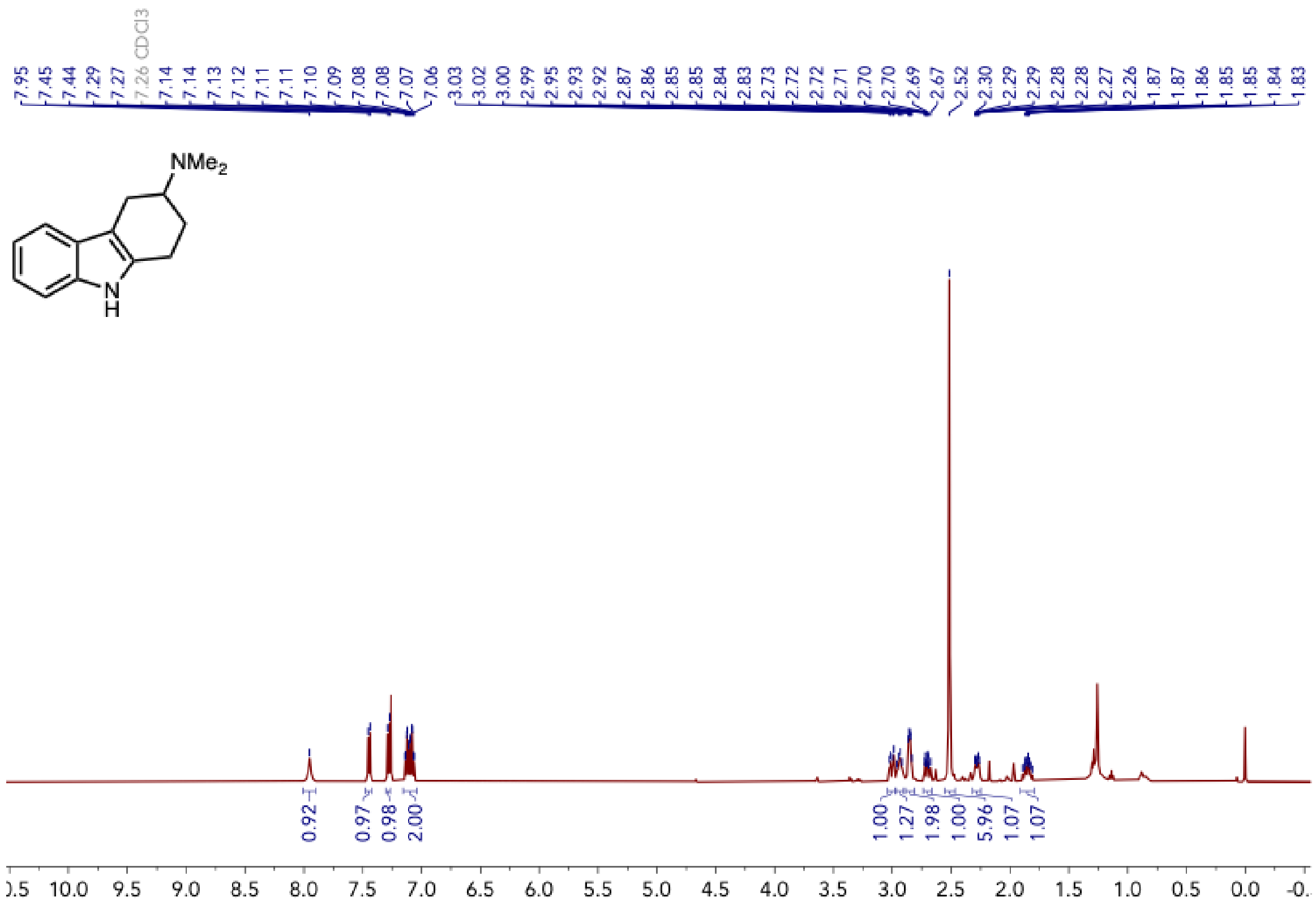

^{1}**H NMR** of **4e** (500 MHz, $CDCl_3$)

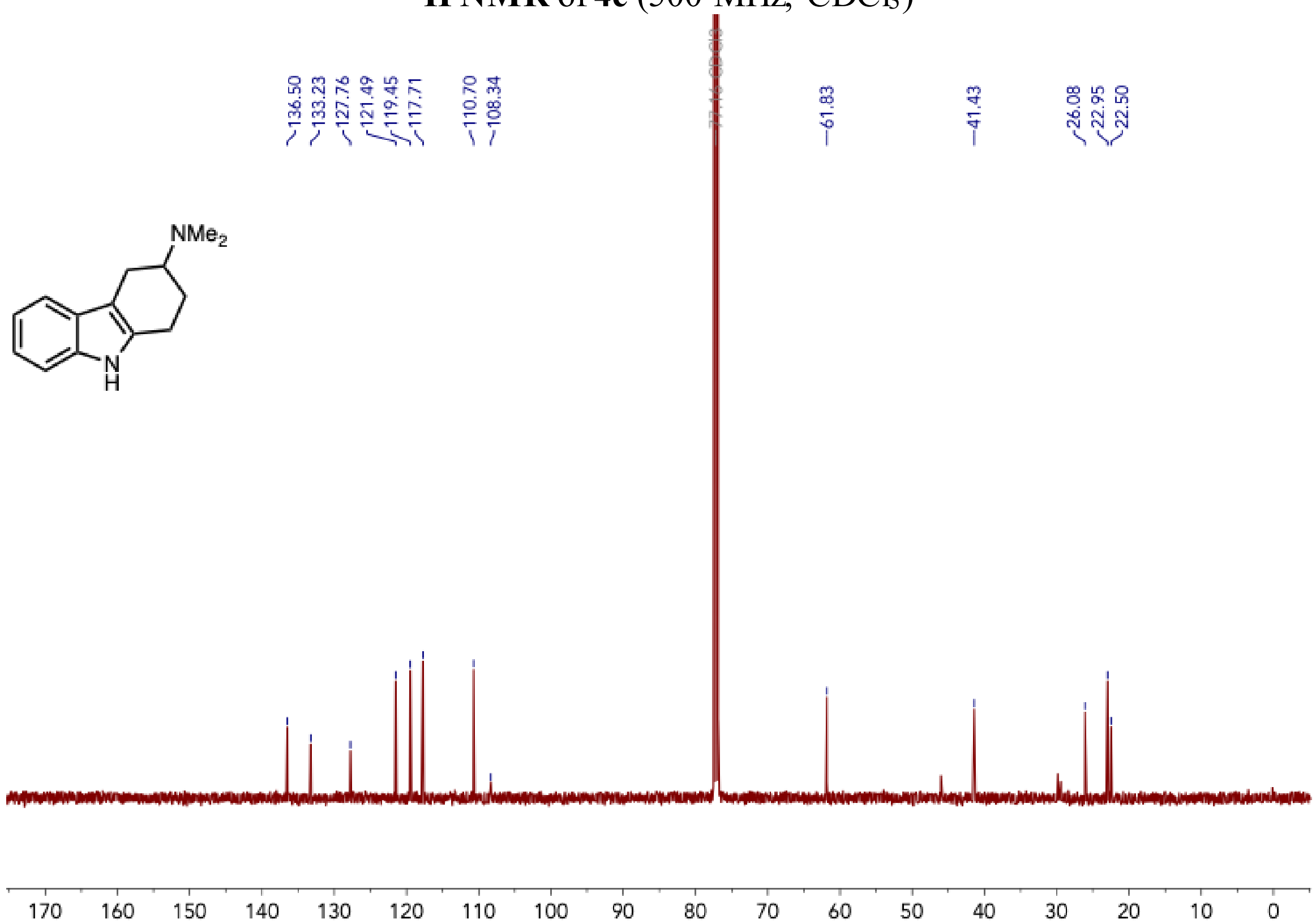

^{13}**C NMR** of **4e** (126 MHz, $CDCl_3$)

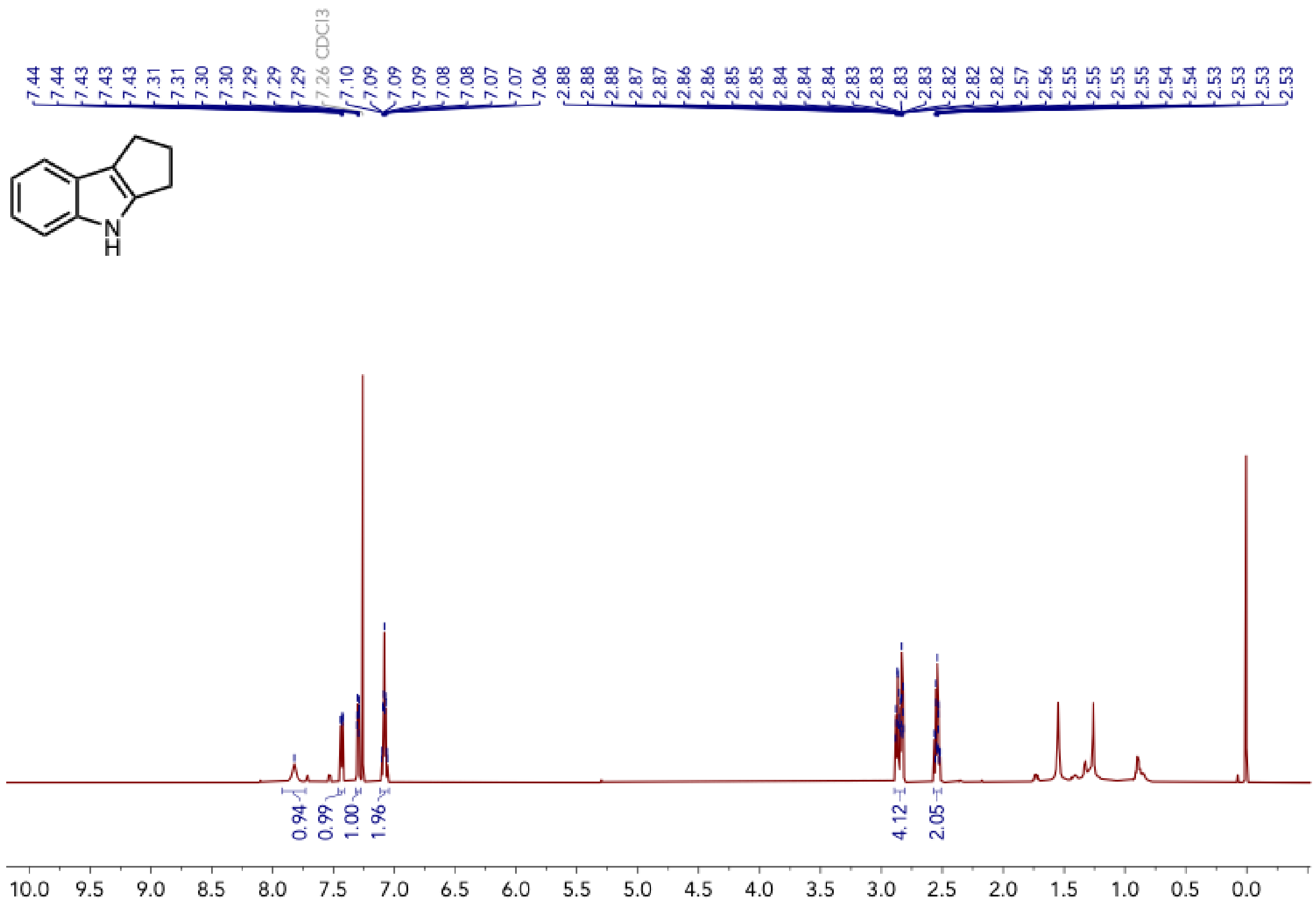

^{1}H NMR of **4f** (500 MHz, $CDCl_3$)

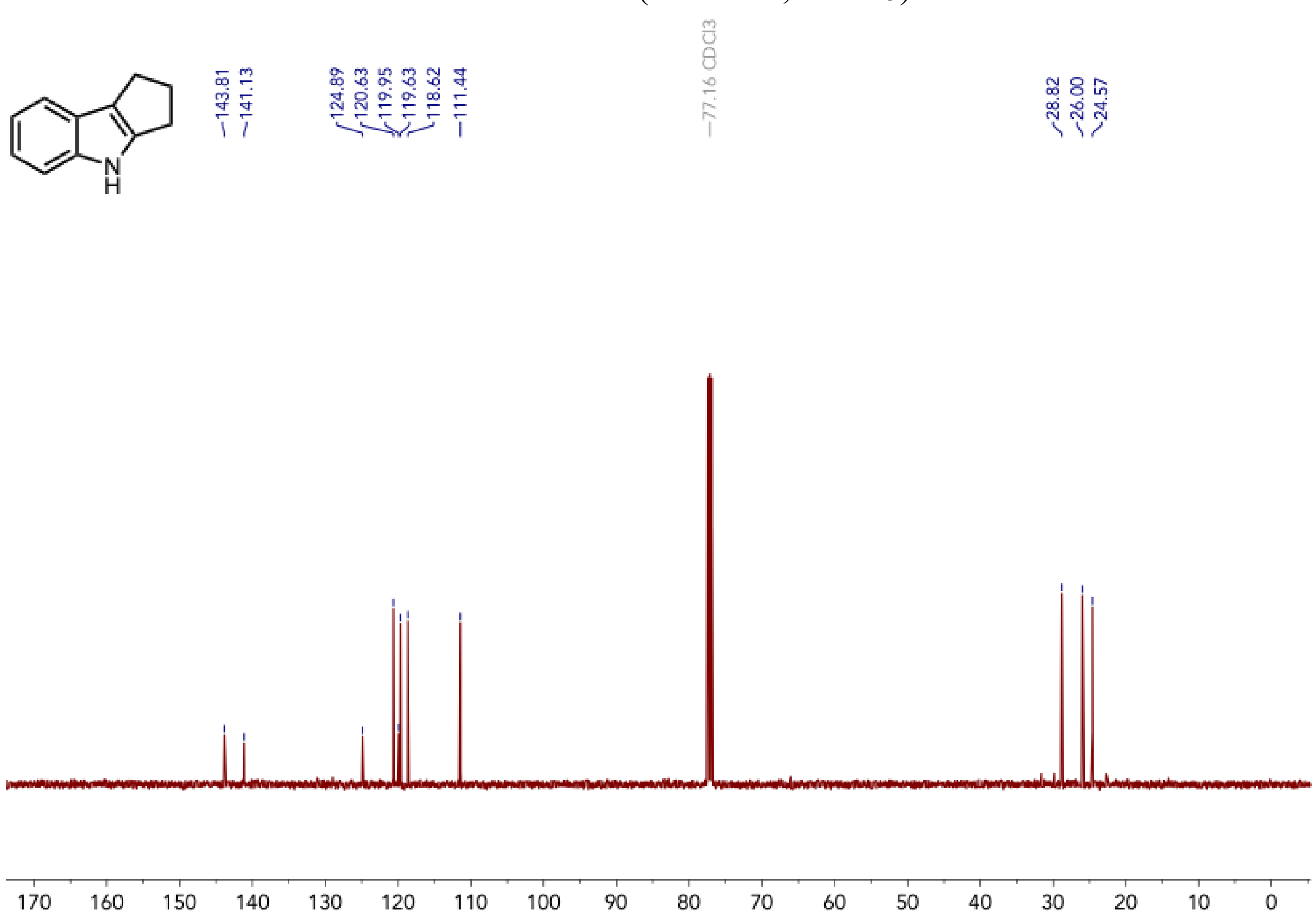

^{13}C NMR of **4f** (126 MHz, $CDCl_3$)

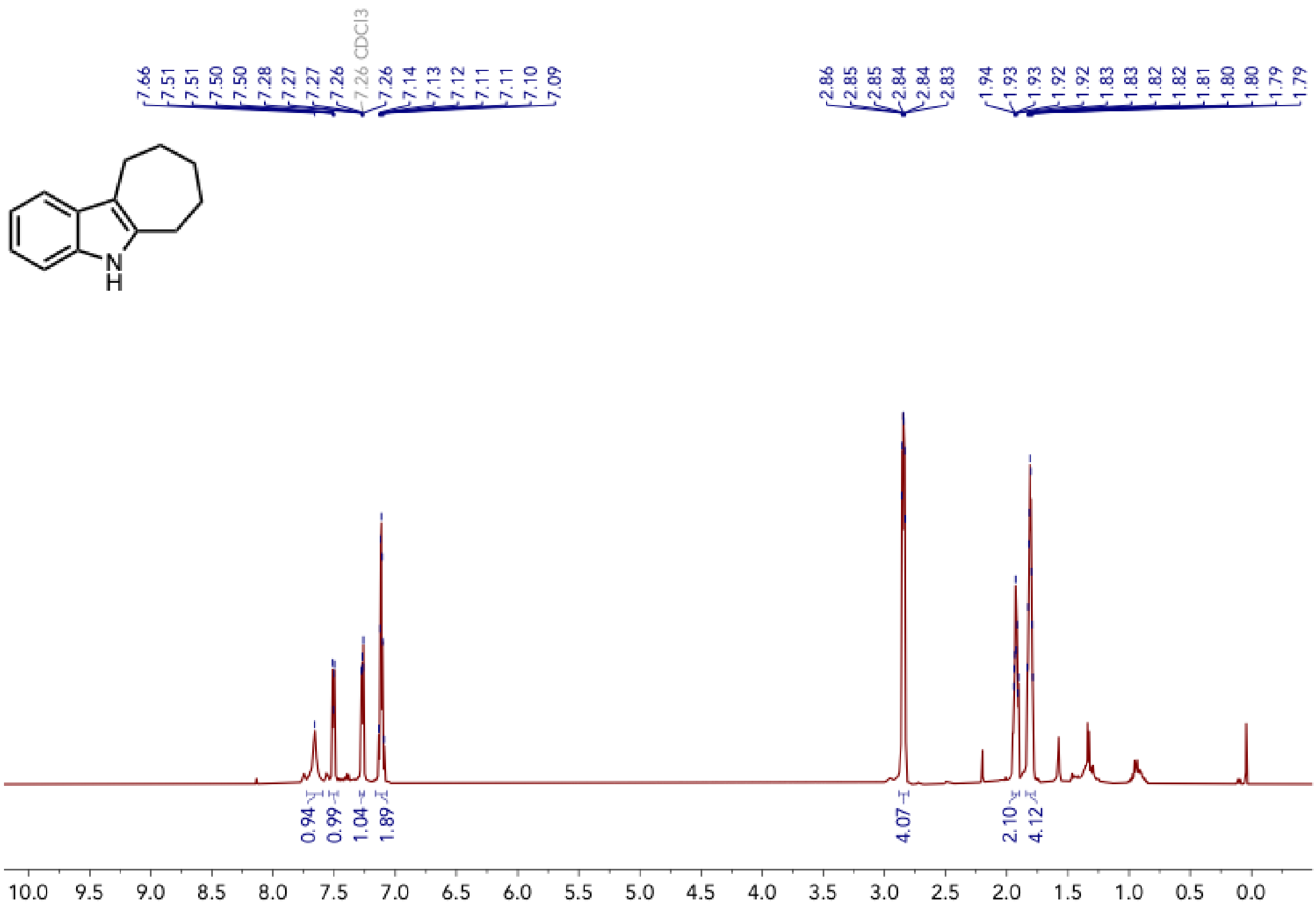

^{1}H NMR of **4g** (600 MHz, $CDCl_3$)

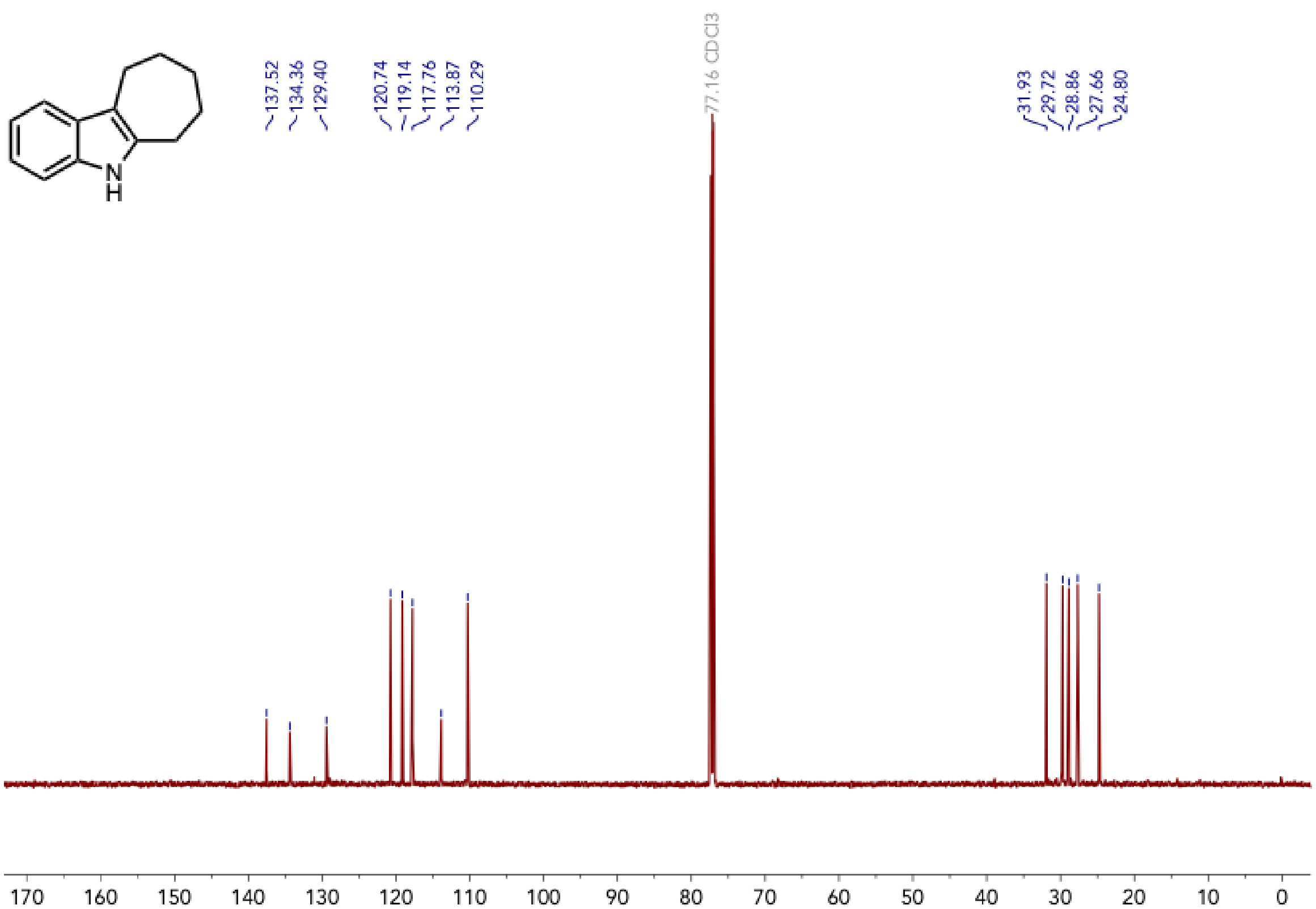

^{13}C NMR of **4g** (150 MHz, $CDCl_3$)

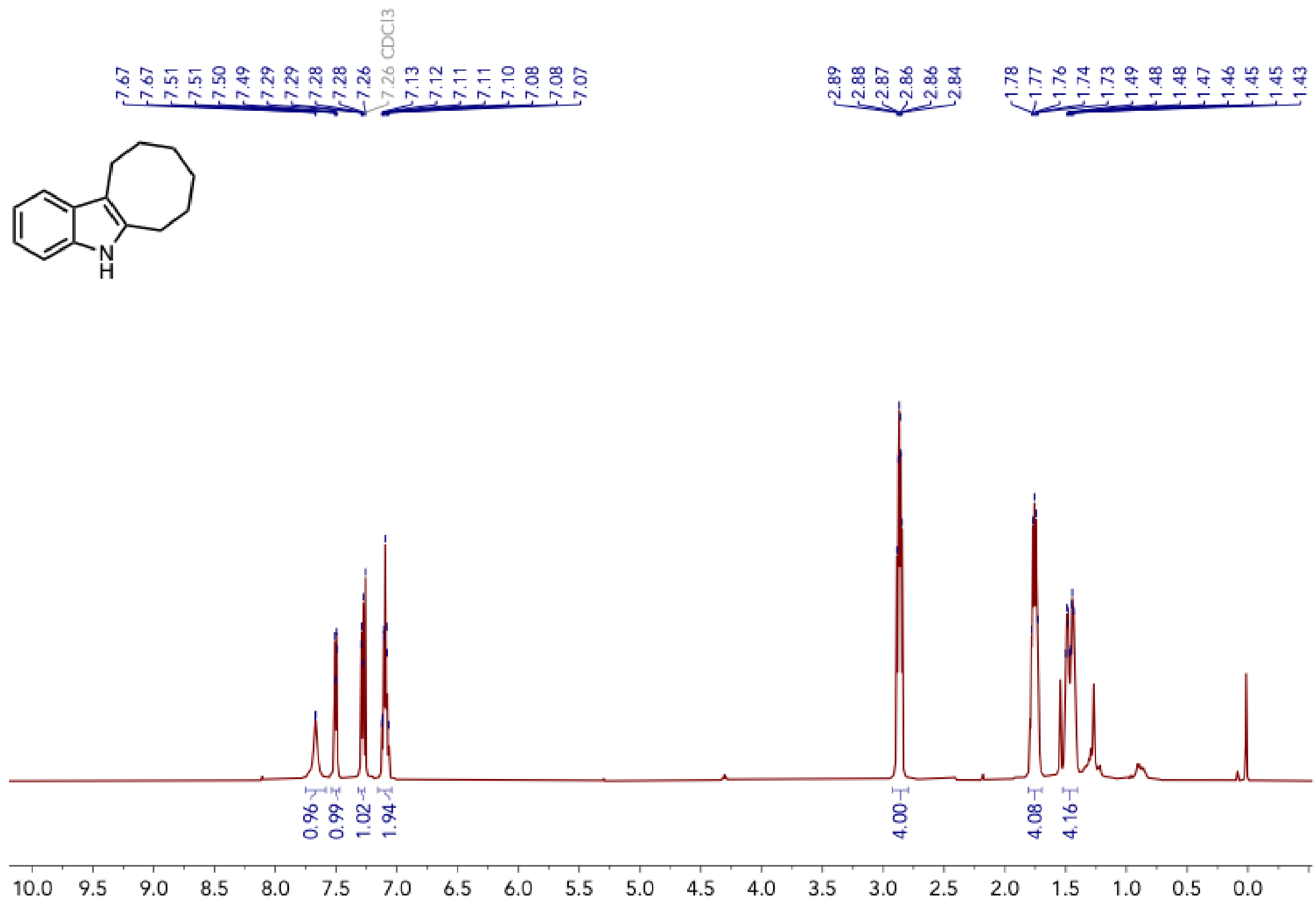

¹H NMR of **4h** (500 MHz, $CDCl_3$)

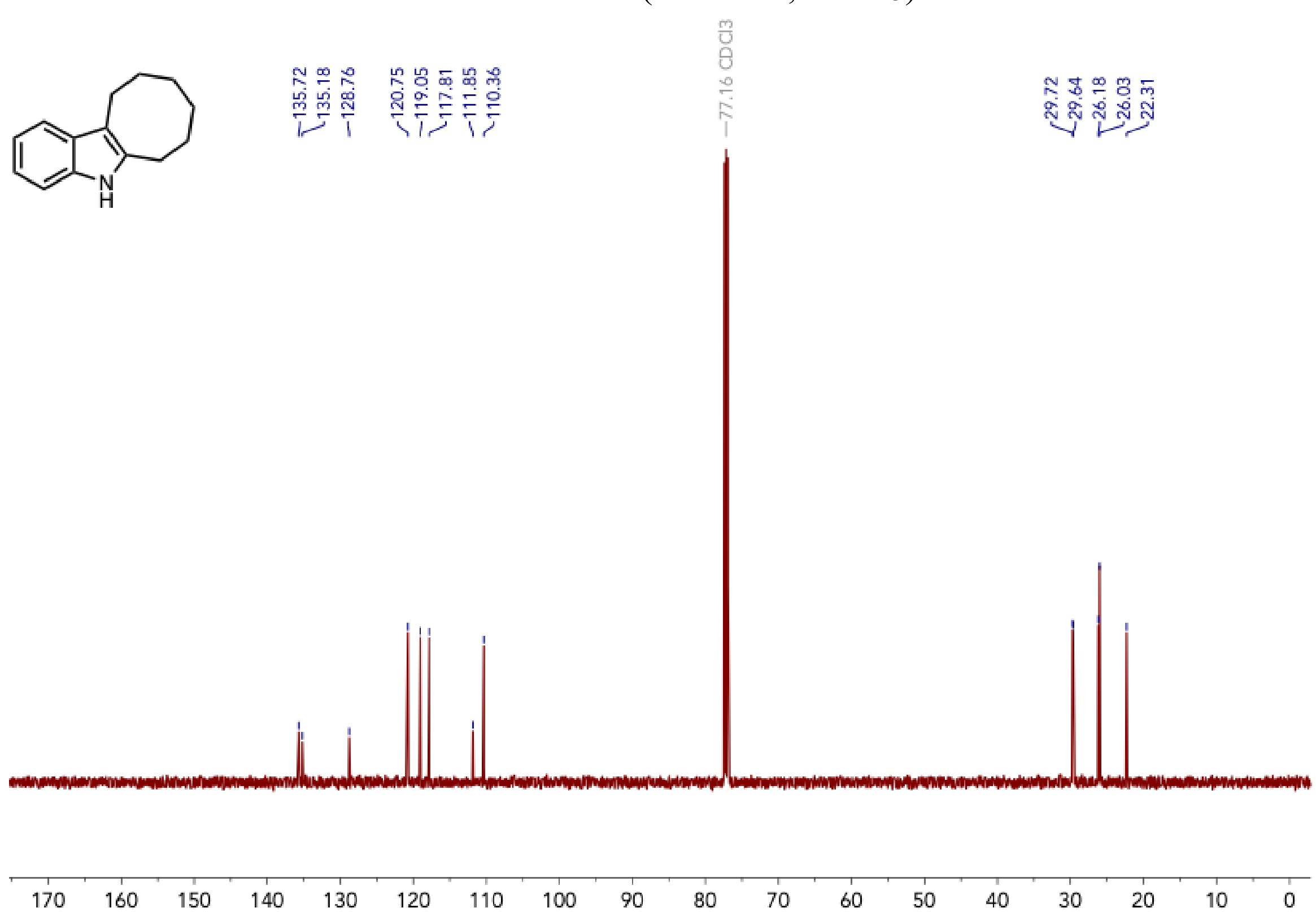

¹³C NMR of **4h** (126 MHz, $CDCl_3$)

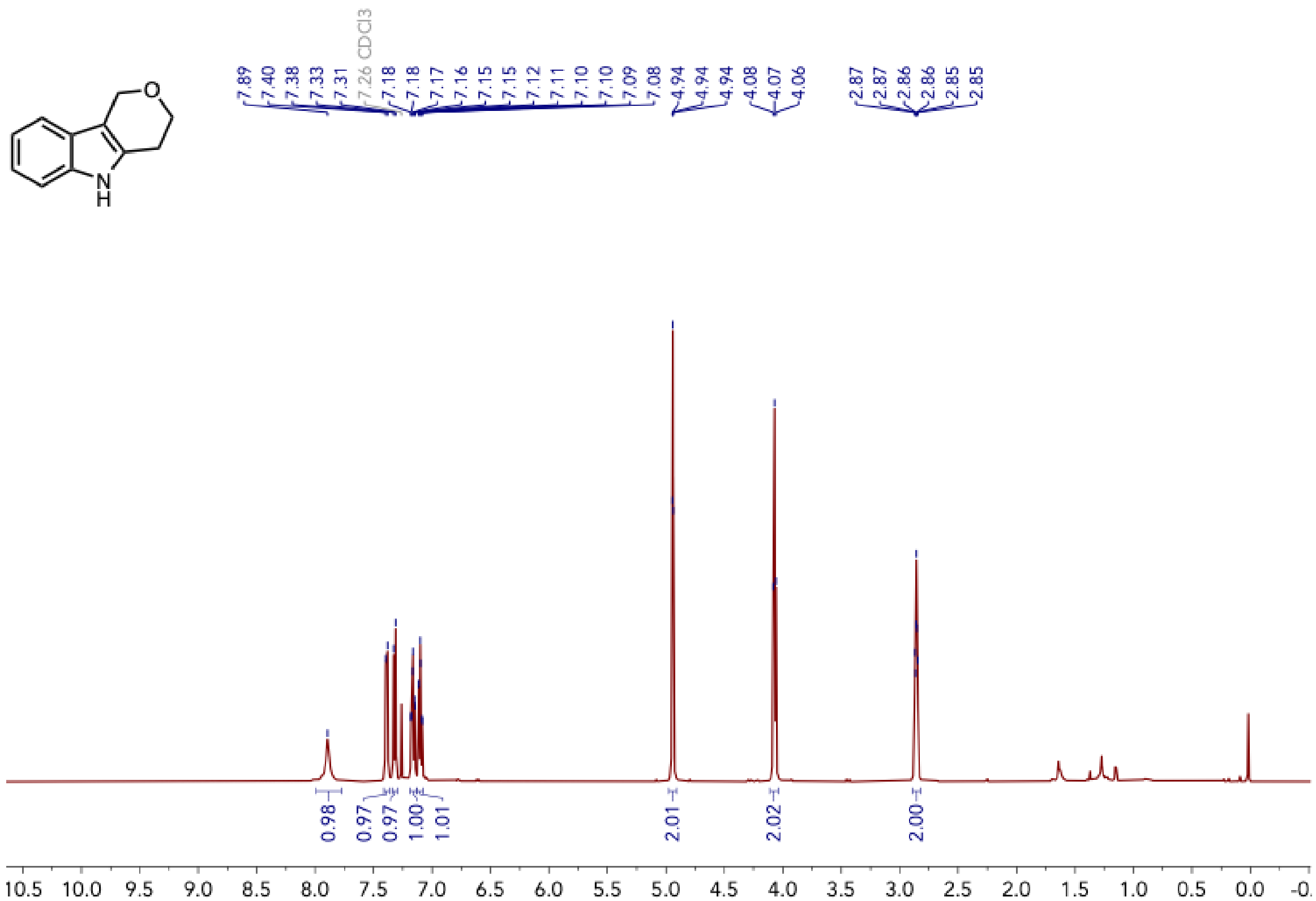

^{1}H NMR of **4i** (500 MHz, $CDCl_3$)

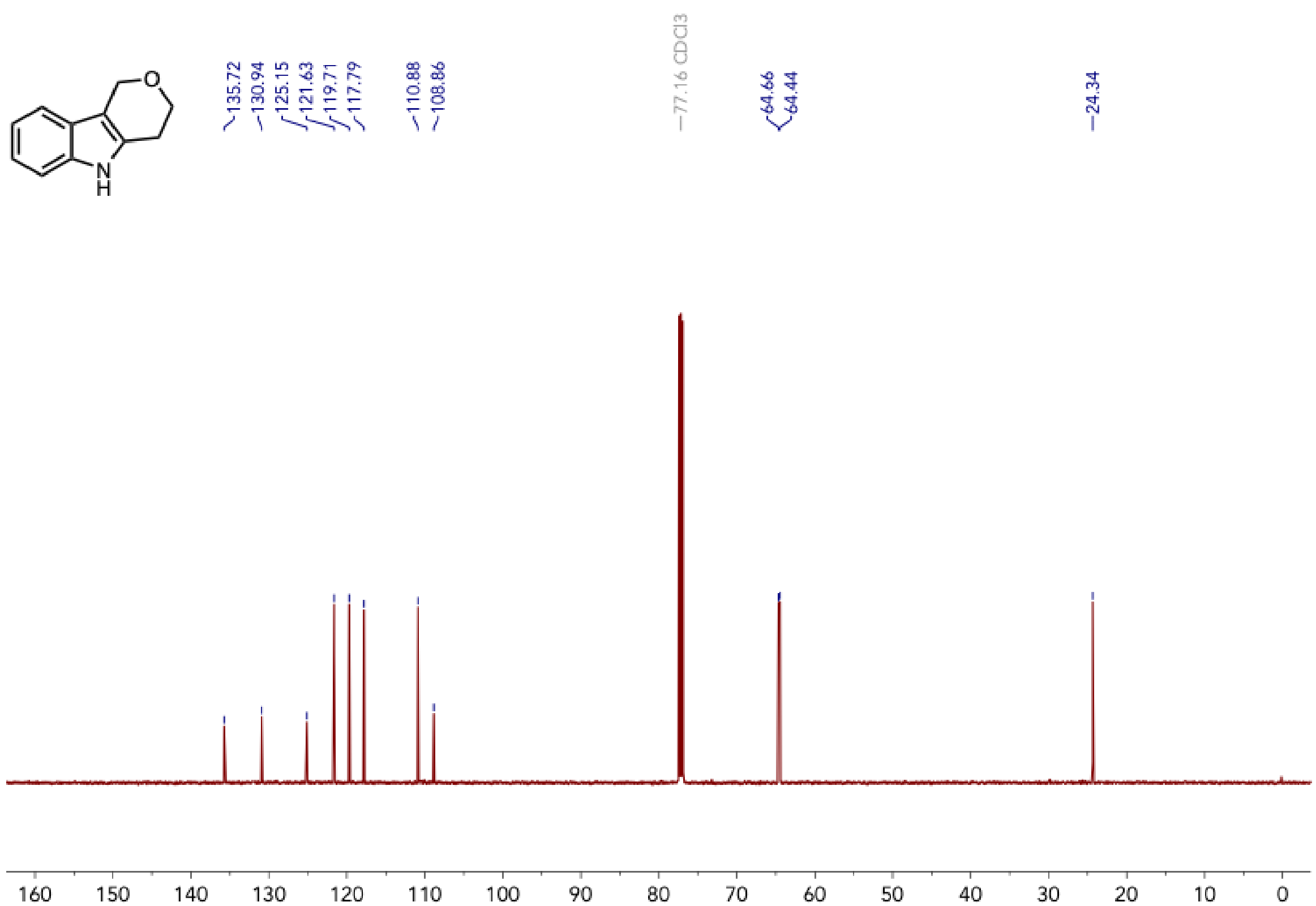

^{13}C NMR of **4i** (150 MHz, $CDCl_3$)

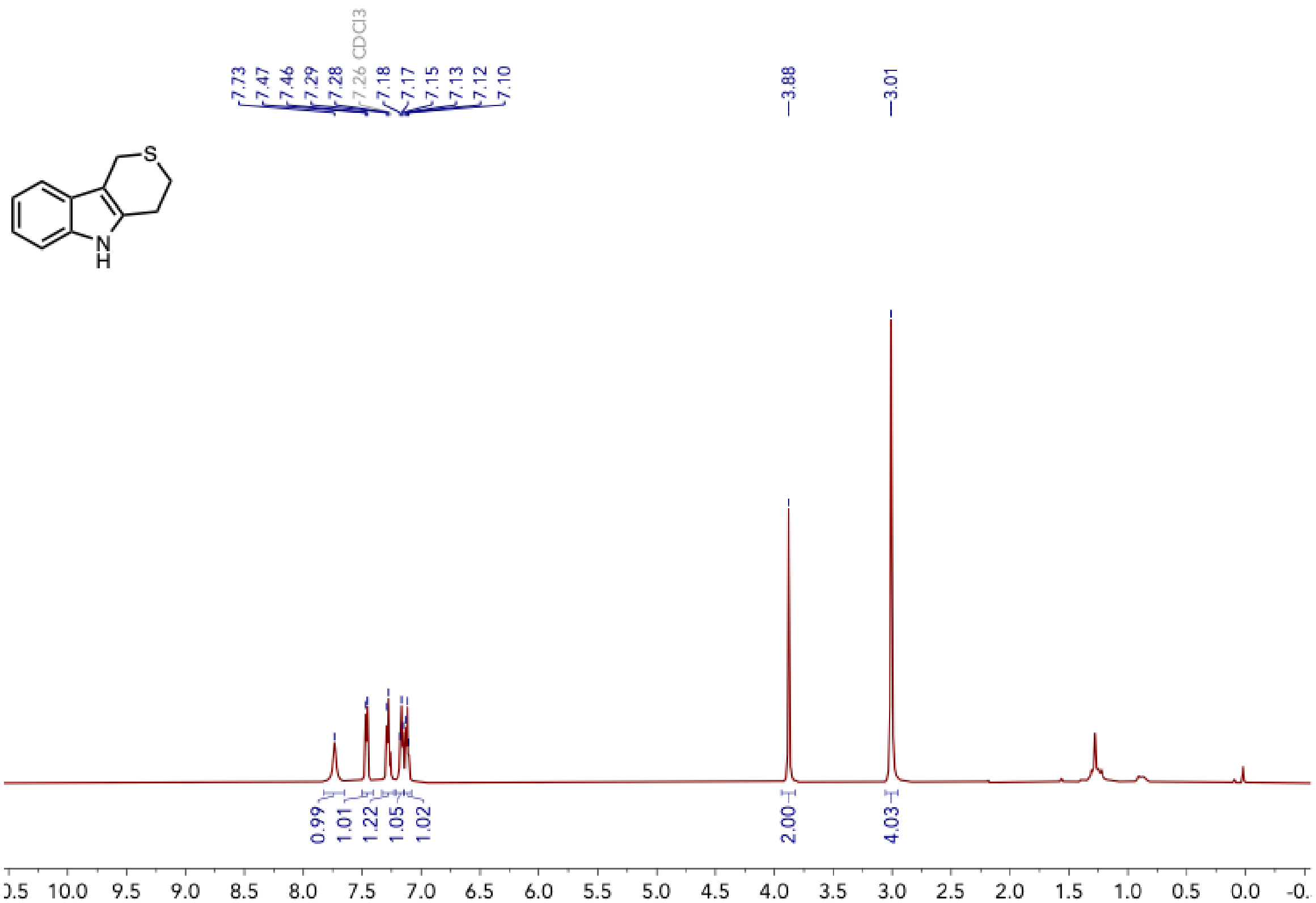

^{1}H NMR of **4j** (500 MHz, $CDCl_3$)

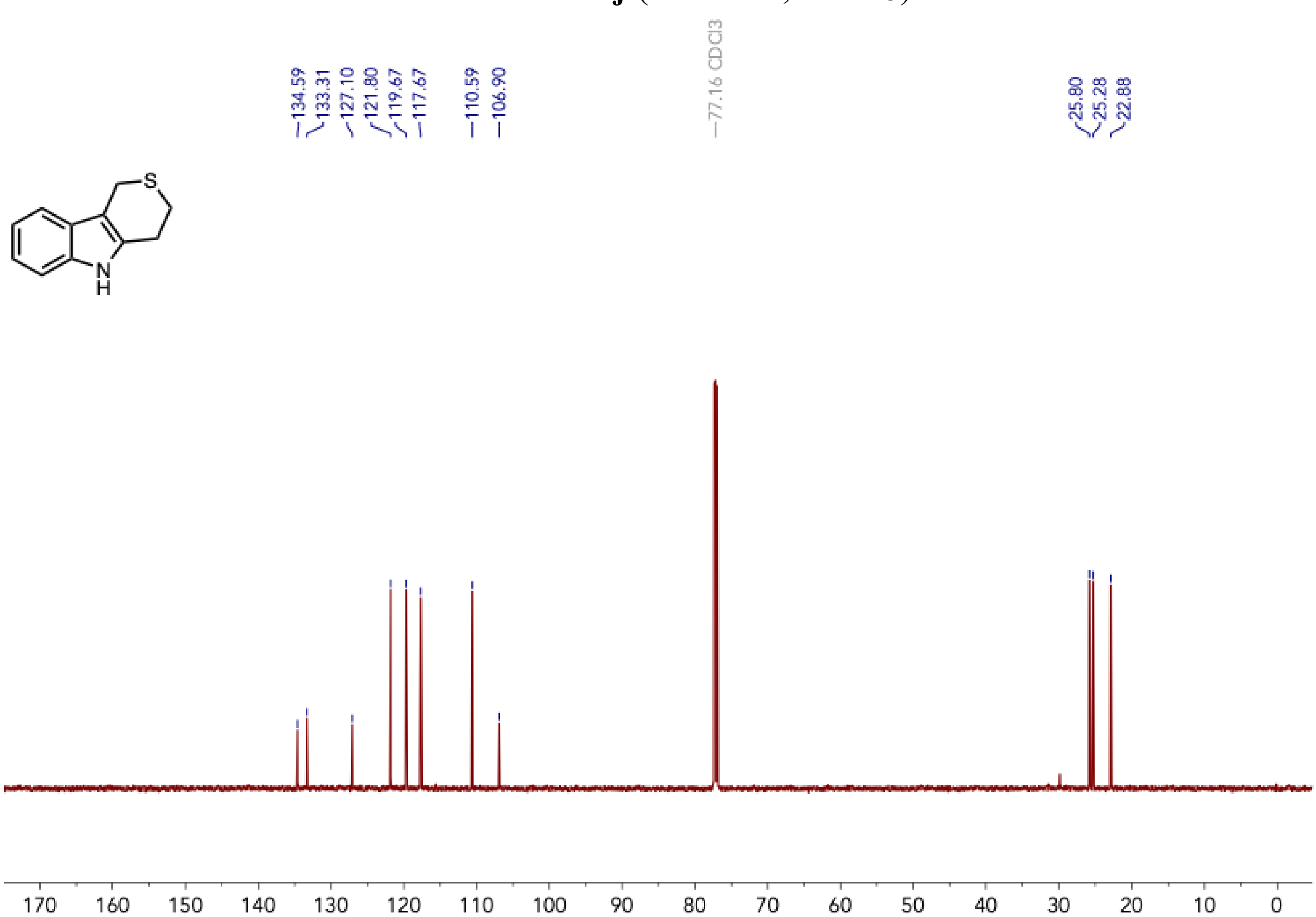

^{13}C NMR of **4j** (126 MHz, $CDCl_3$)

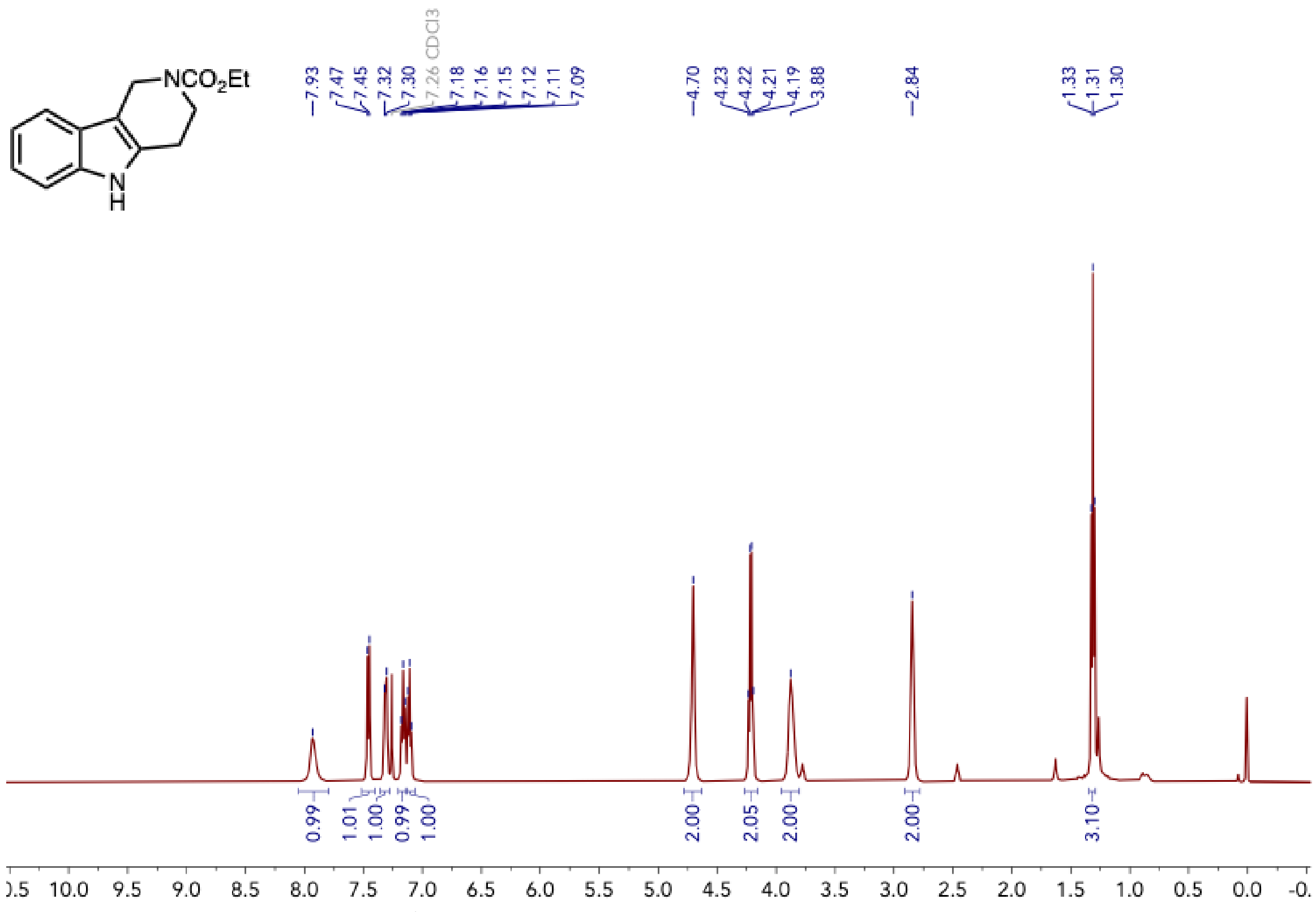

^{1}**H NMR** of **4k** (500 MHz, $CDCl_3$)

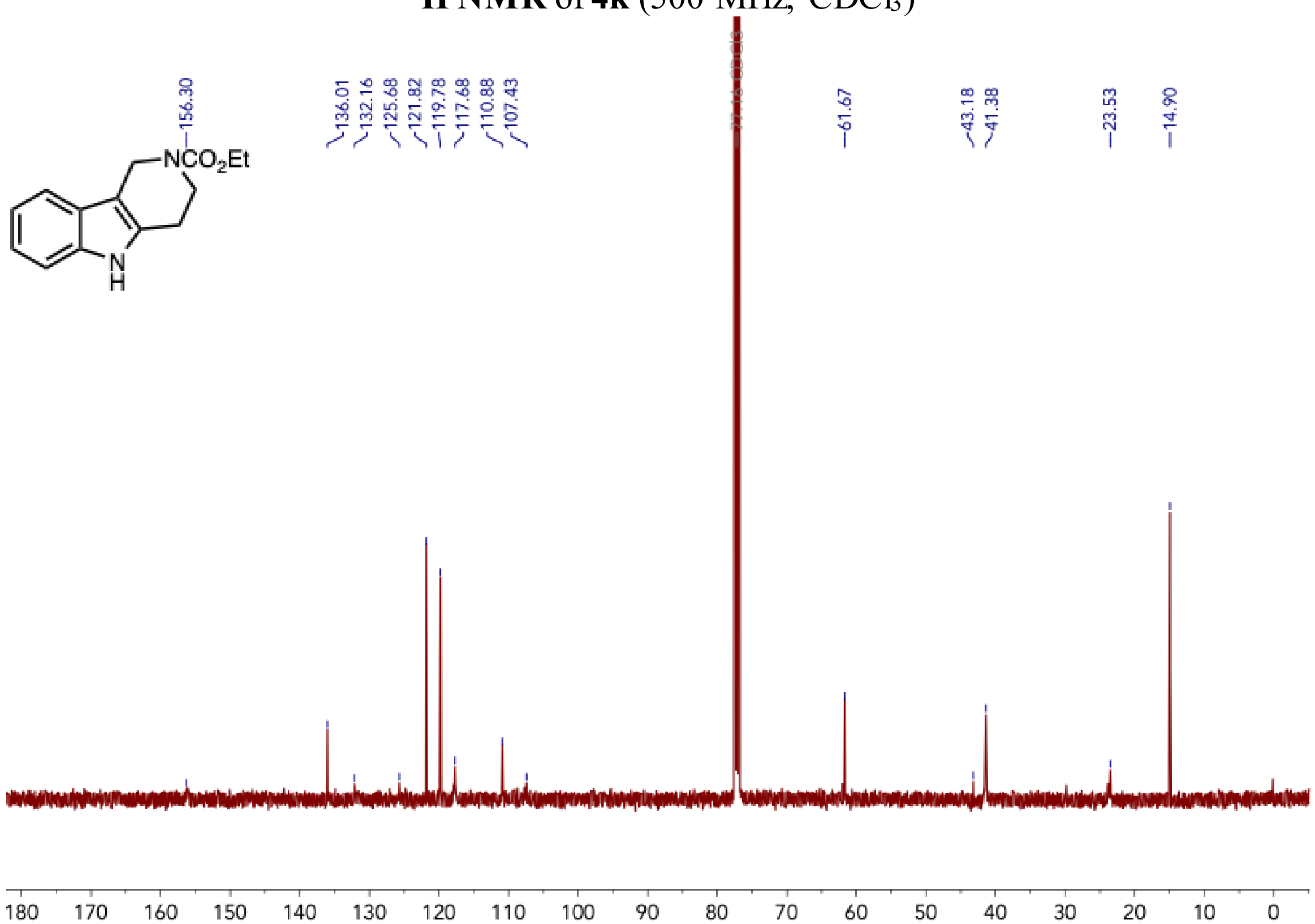

^{13}**C NMR** of **4k** (126 MHz, $CDCl_3$)

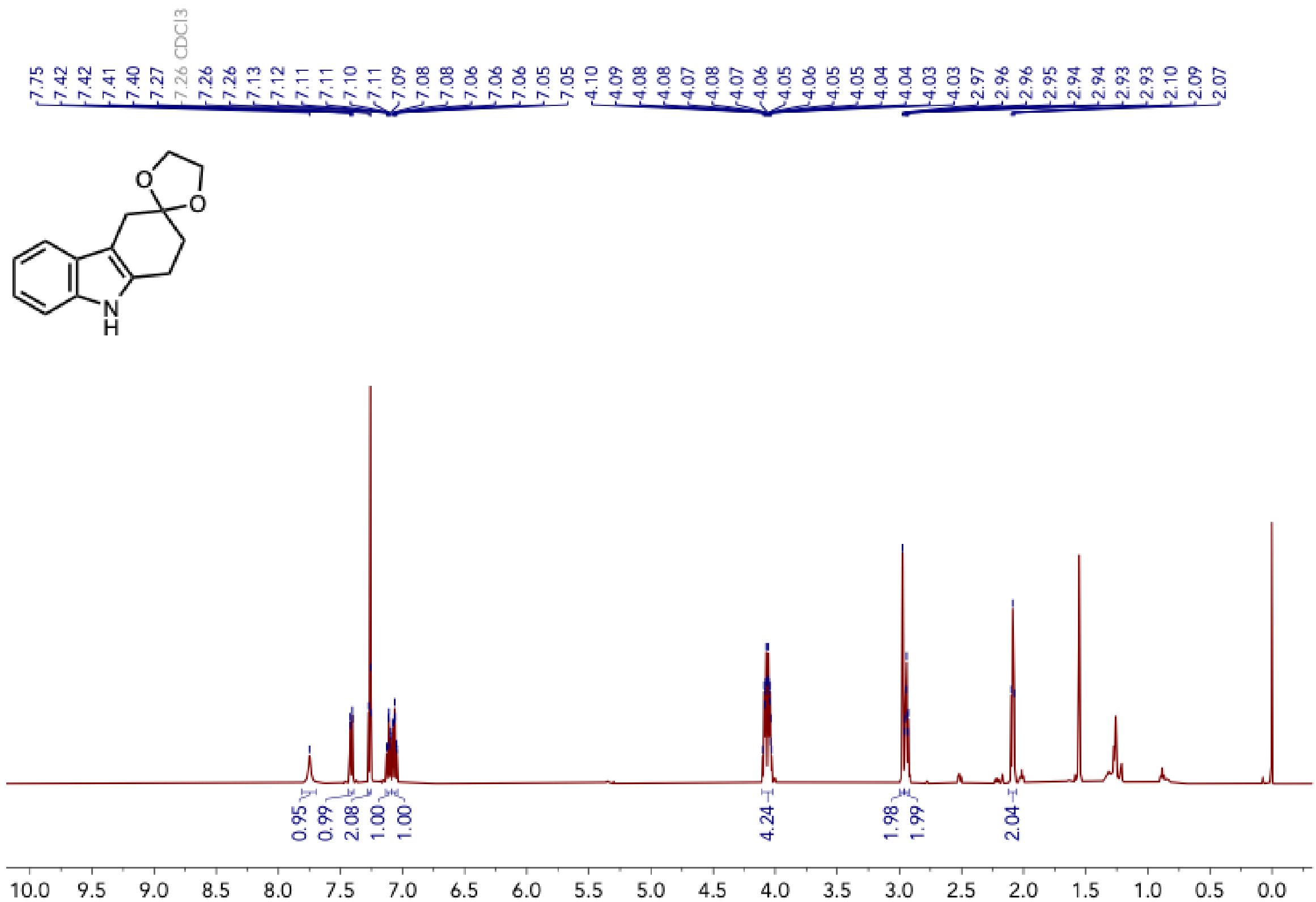

¹H NMR of **4l** (500 MHz, $CDCl_3$)

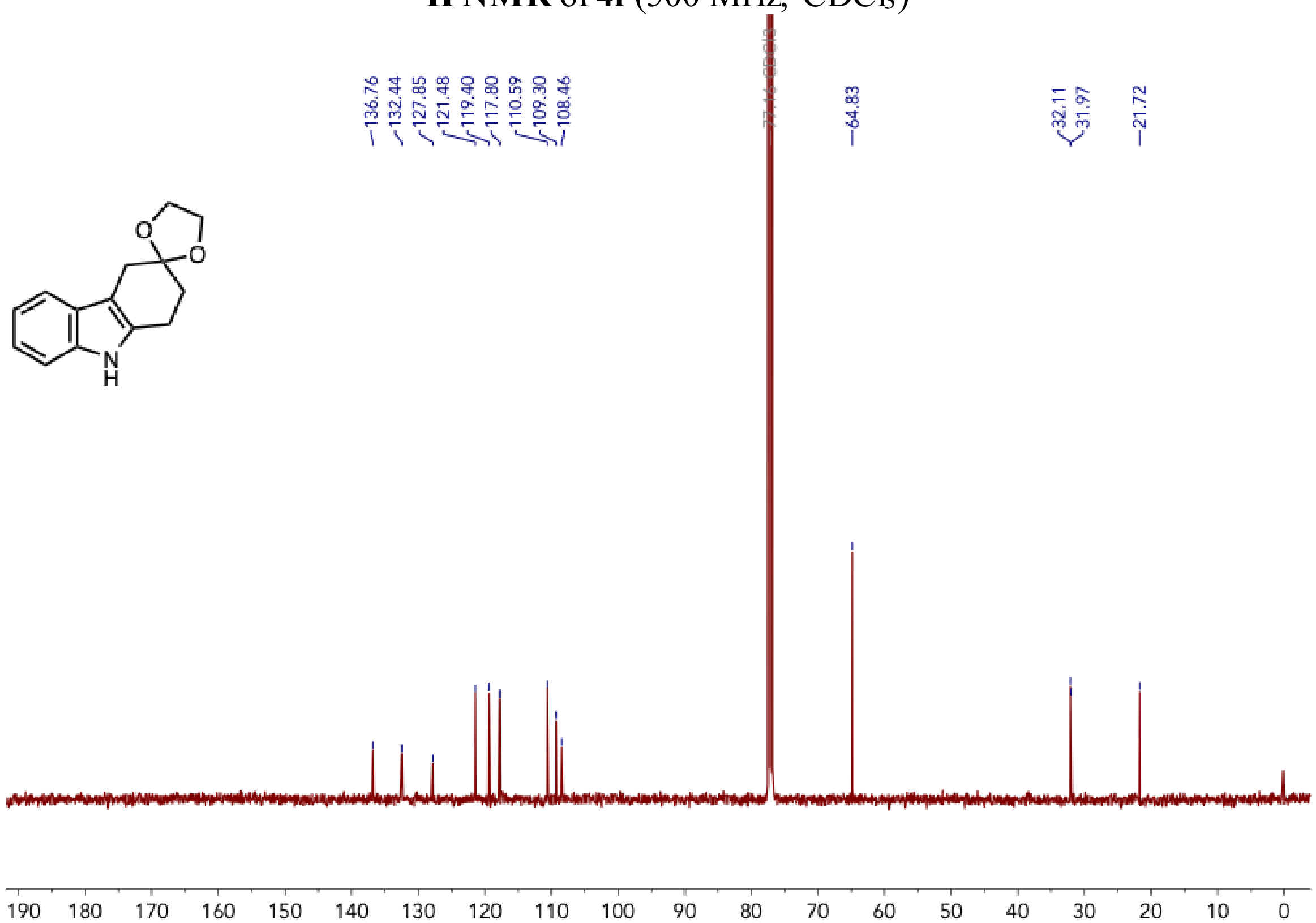

¹³C NMR of **4l** (126 MHz, $CDCl_3$)

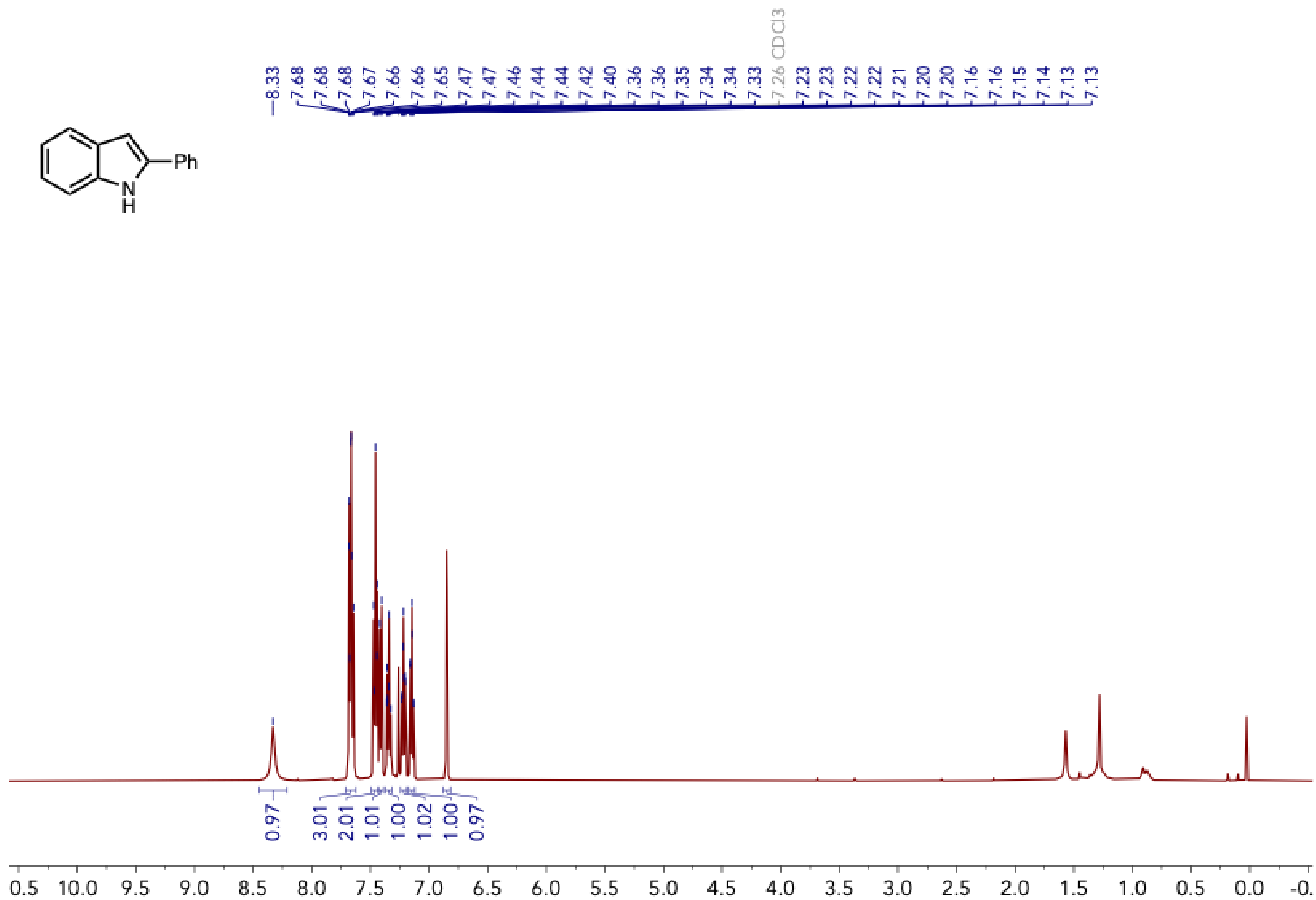

^{1}H NMR of **4m** (500 MHz, $CDCl_3$)

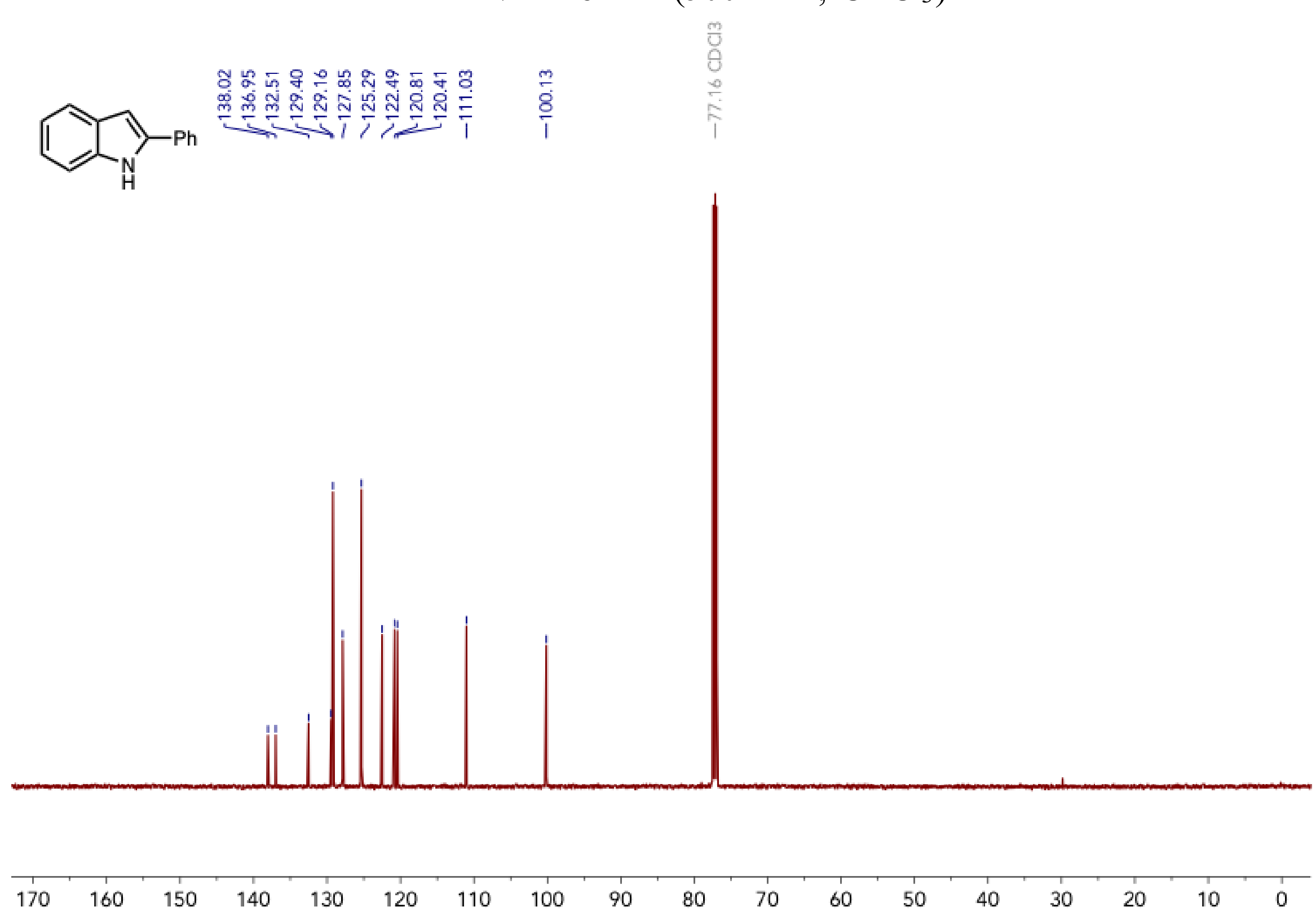

^{13}C NMR of **4m** (126 MHz, $CDCl_3$)

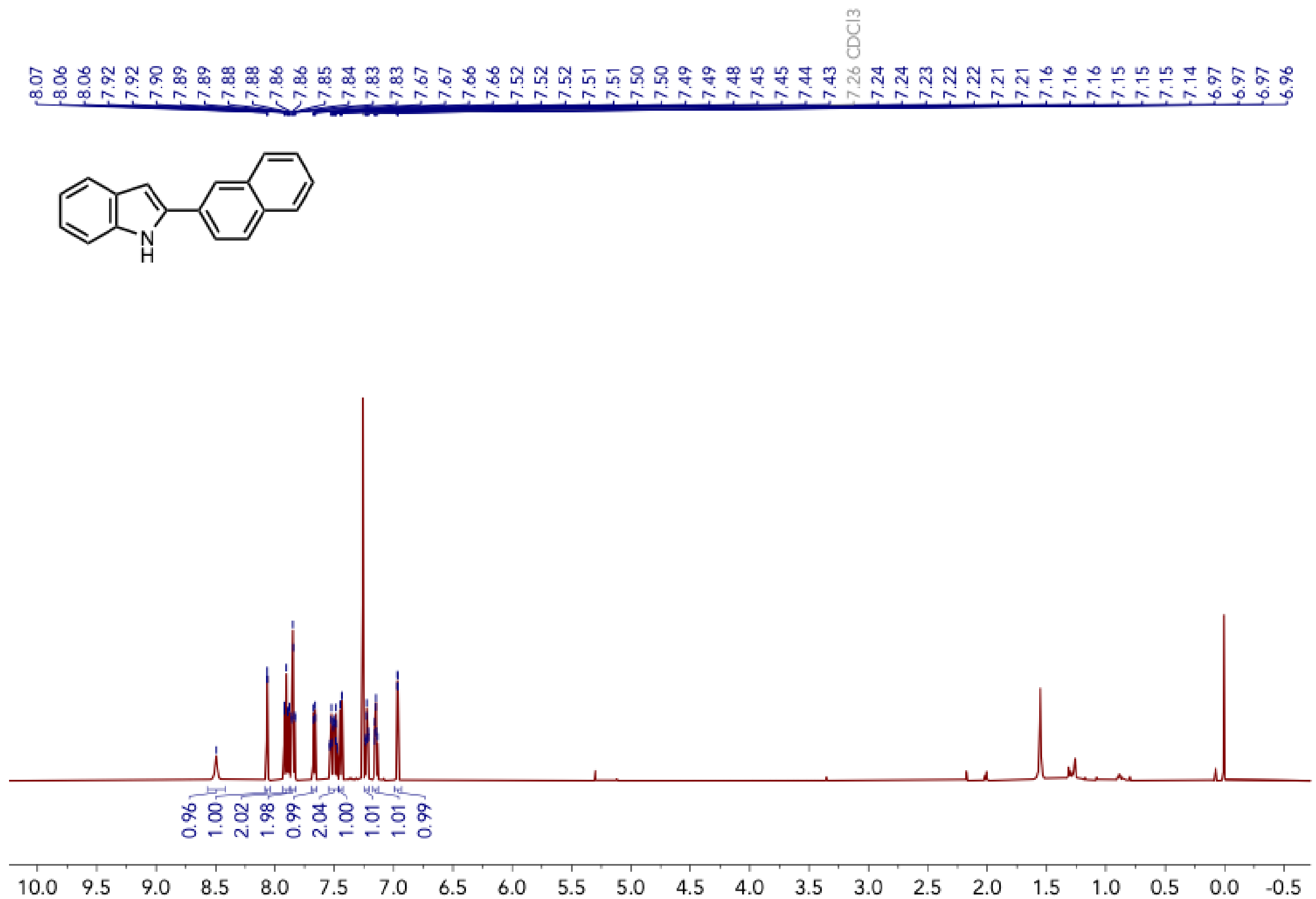

^{1}H NMR of **4n** (500 MHz, $CDCl_3$)

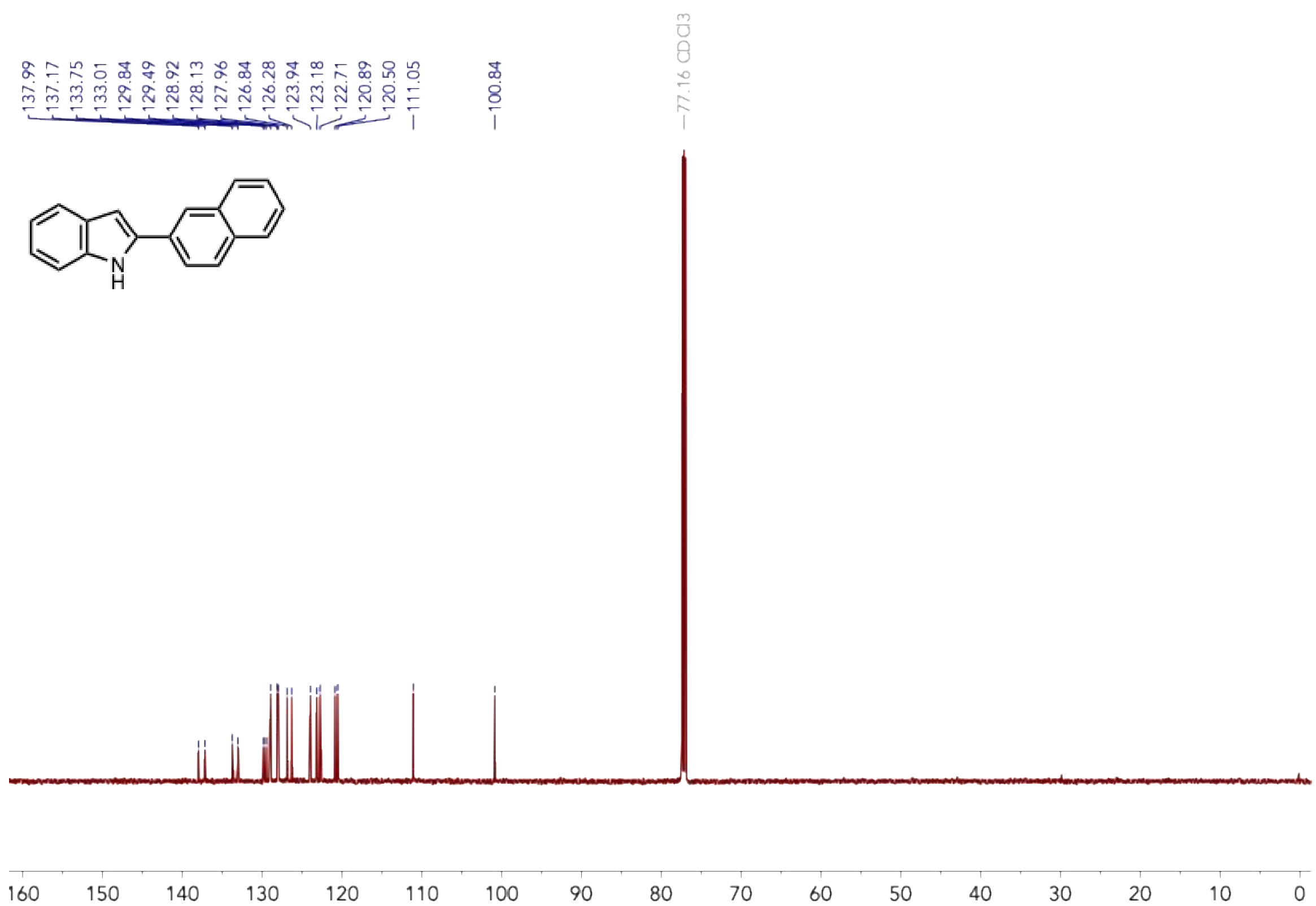

^{13}C NMR of **4n** (150 MHz, $CDCl_3$)

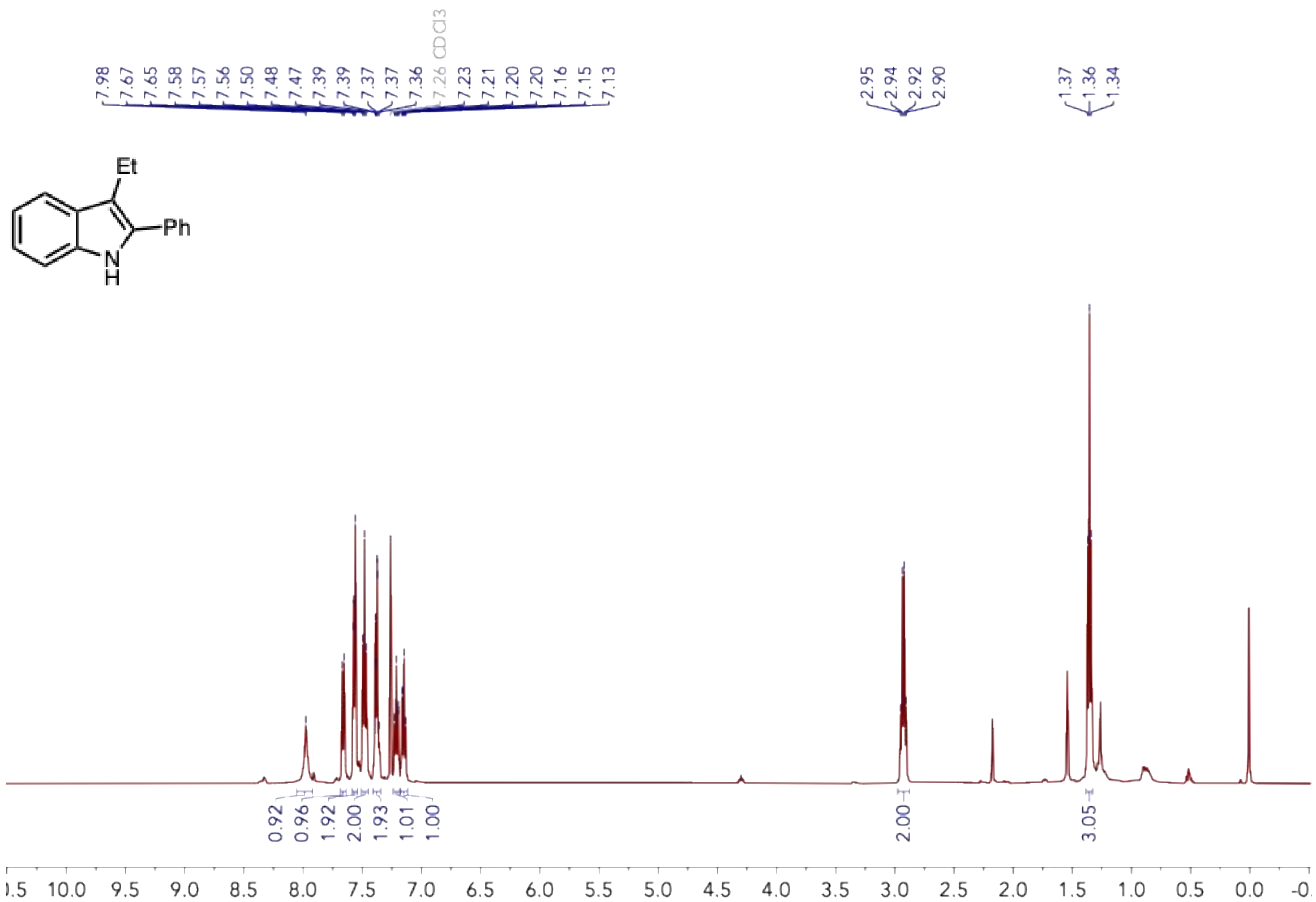

^{1}H NMR of **4o/8b** (500 MHz, $CDCl_3$)

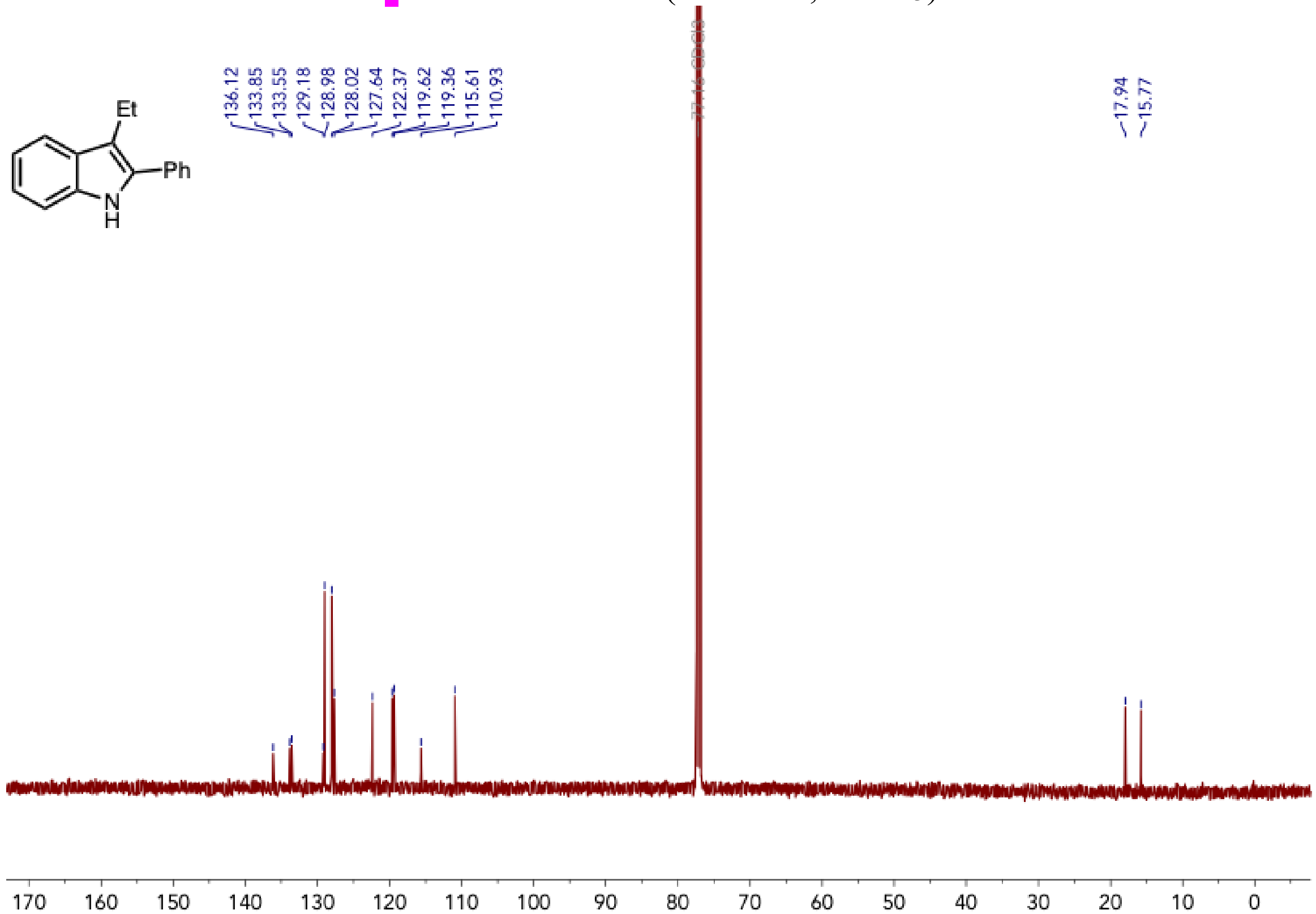

^{13}C NMR of **4o/8b** (126 MHz, $CDCl_3$)

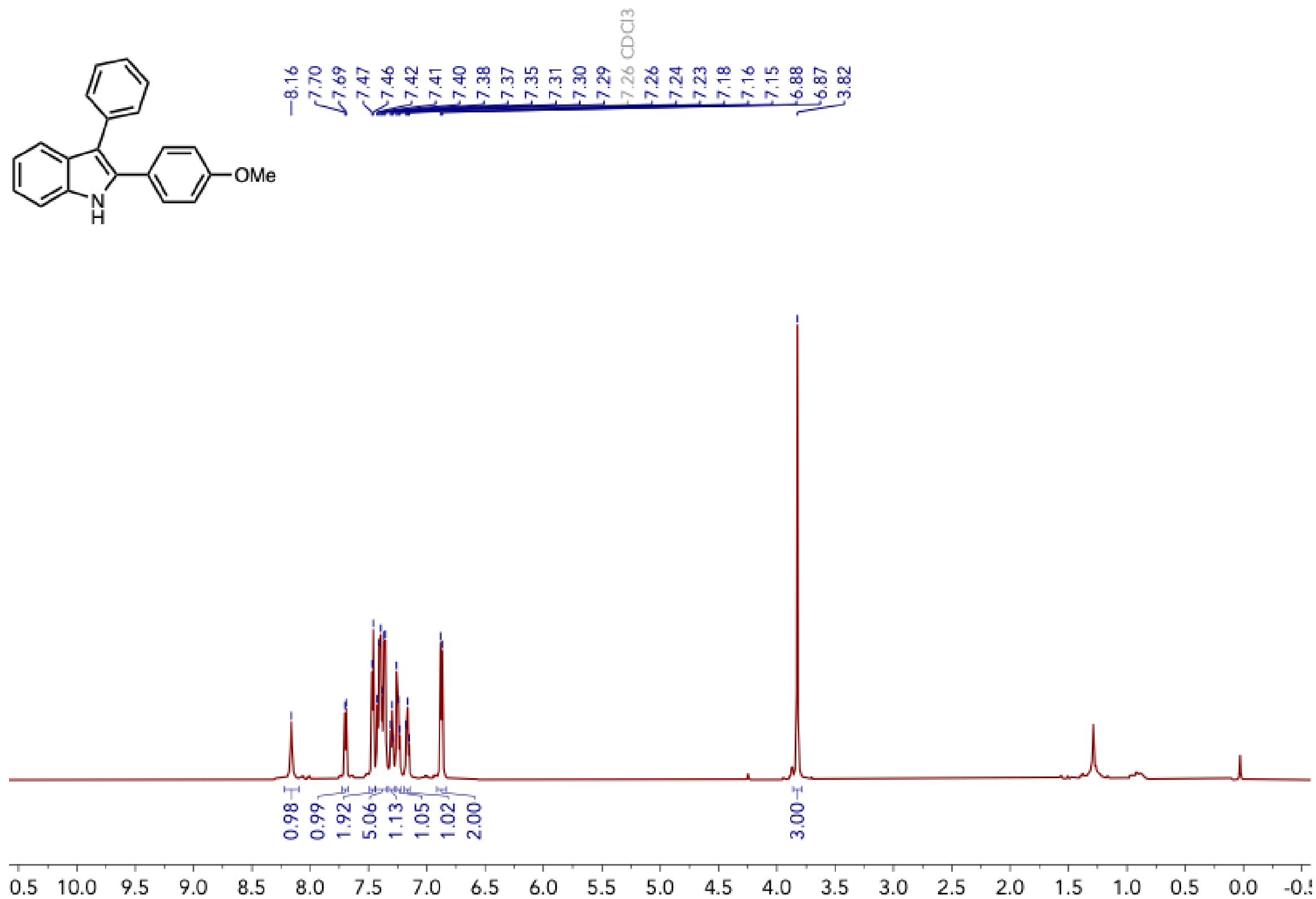

^{1}H NMR of **4p** (600 MHz, $CDCl_3$)

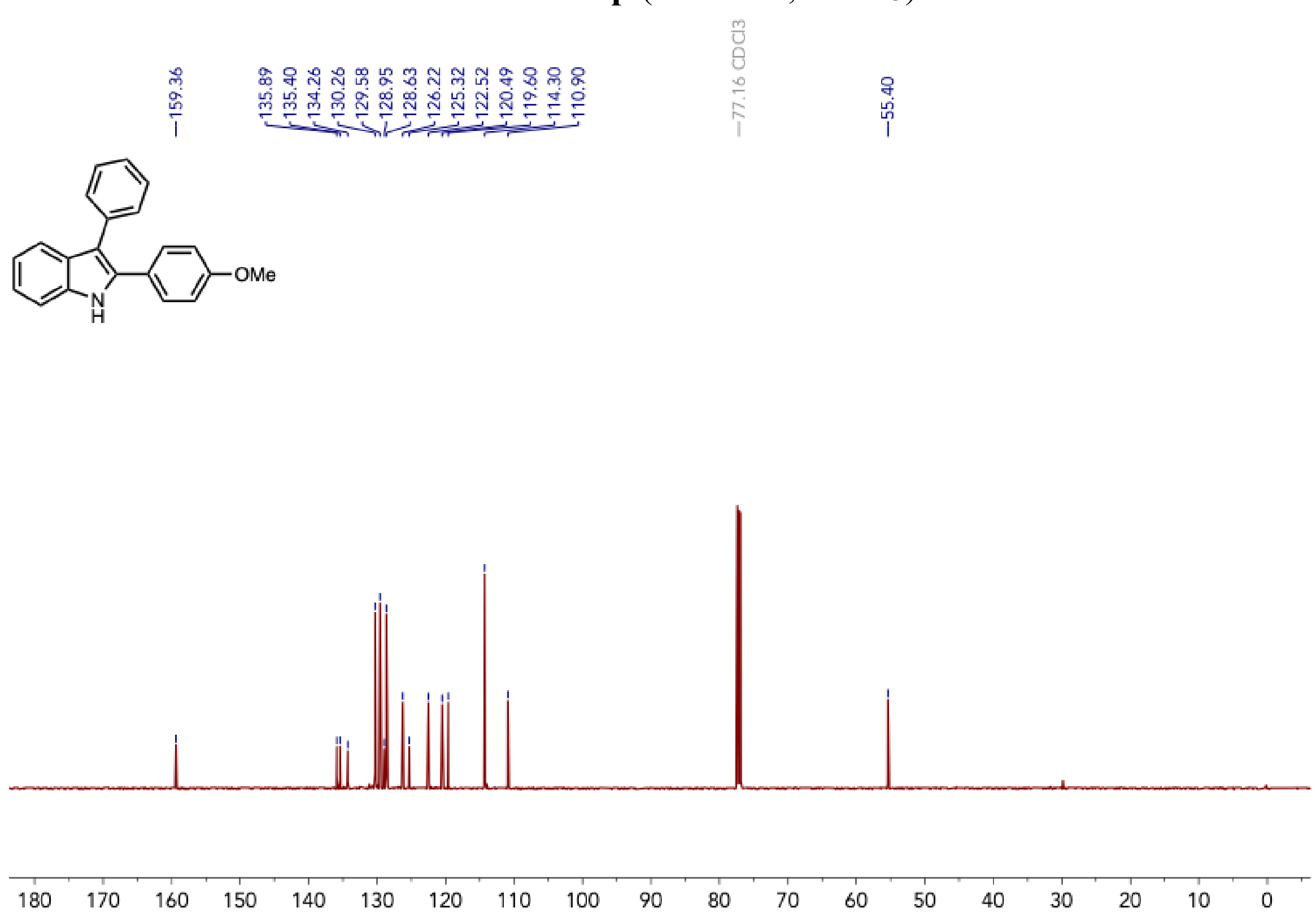

^{13}C NMR of **4p** (150 MHz, $CDCl_3$)

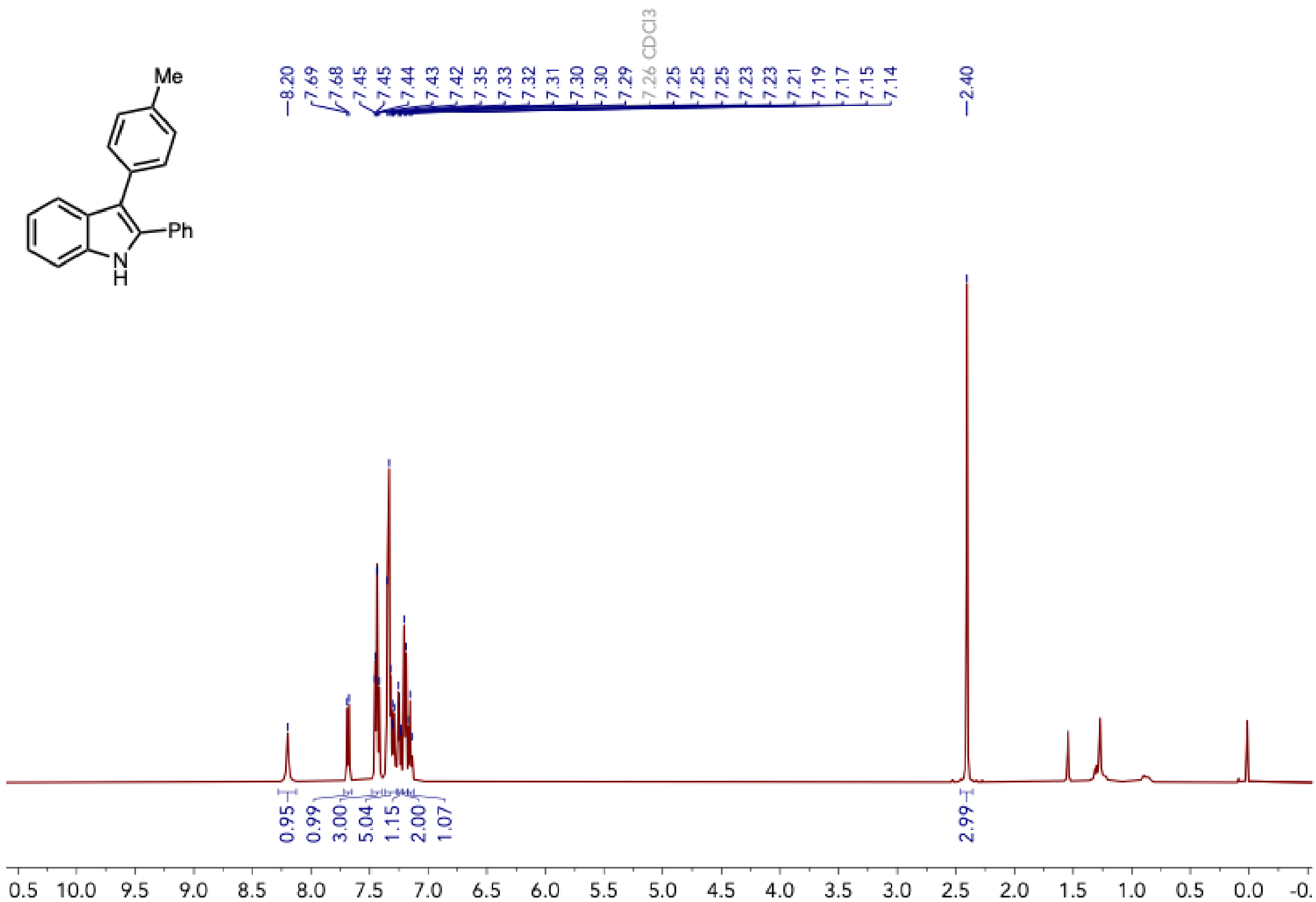

^{1}H NMR of **4q** (500 MHz, $CDCl_3$)

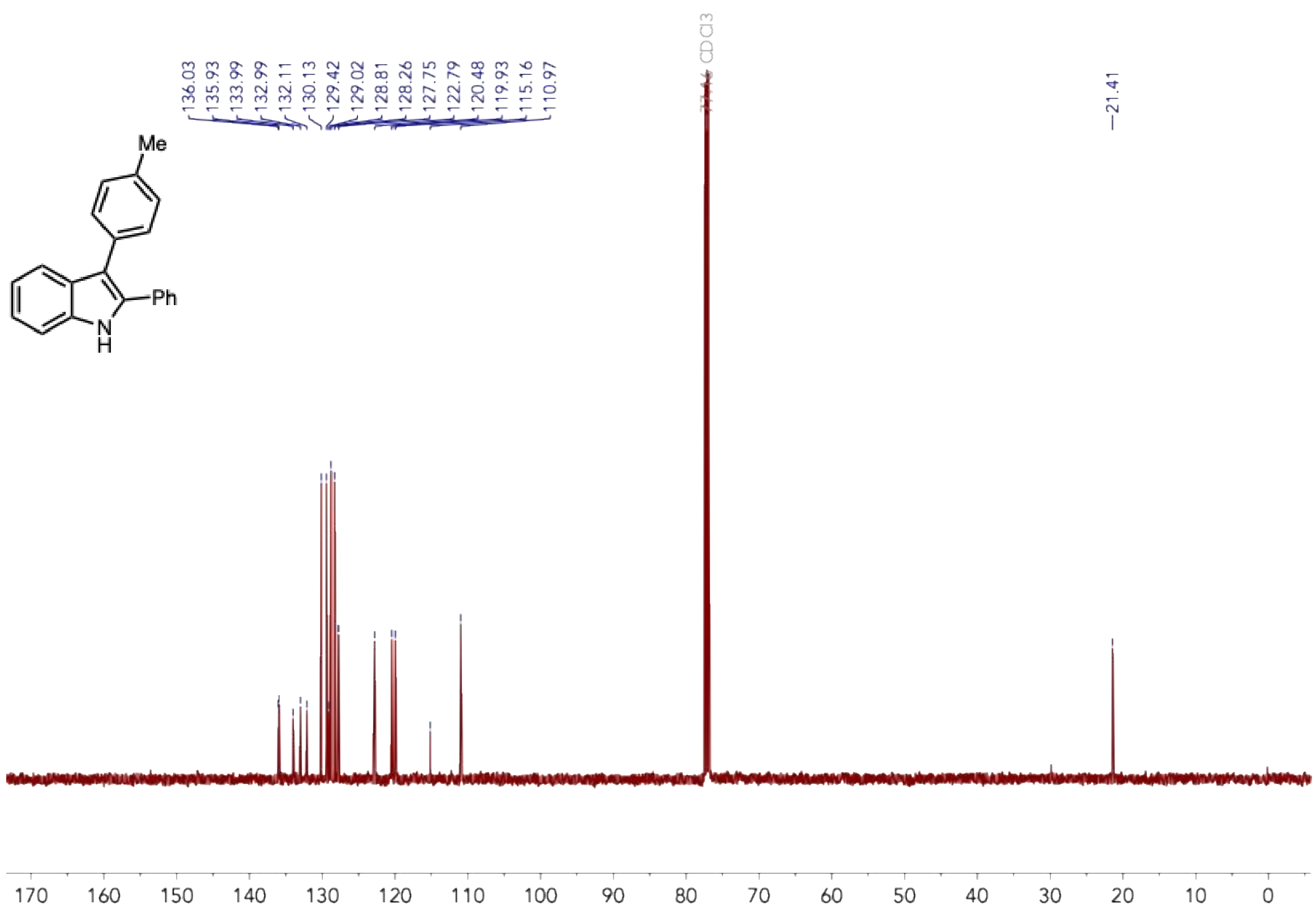

^{13}C NMR of **4q** (126 MHz, $CDCl_3$)

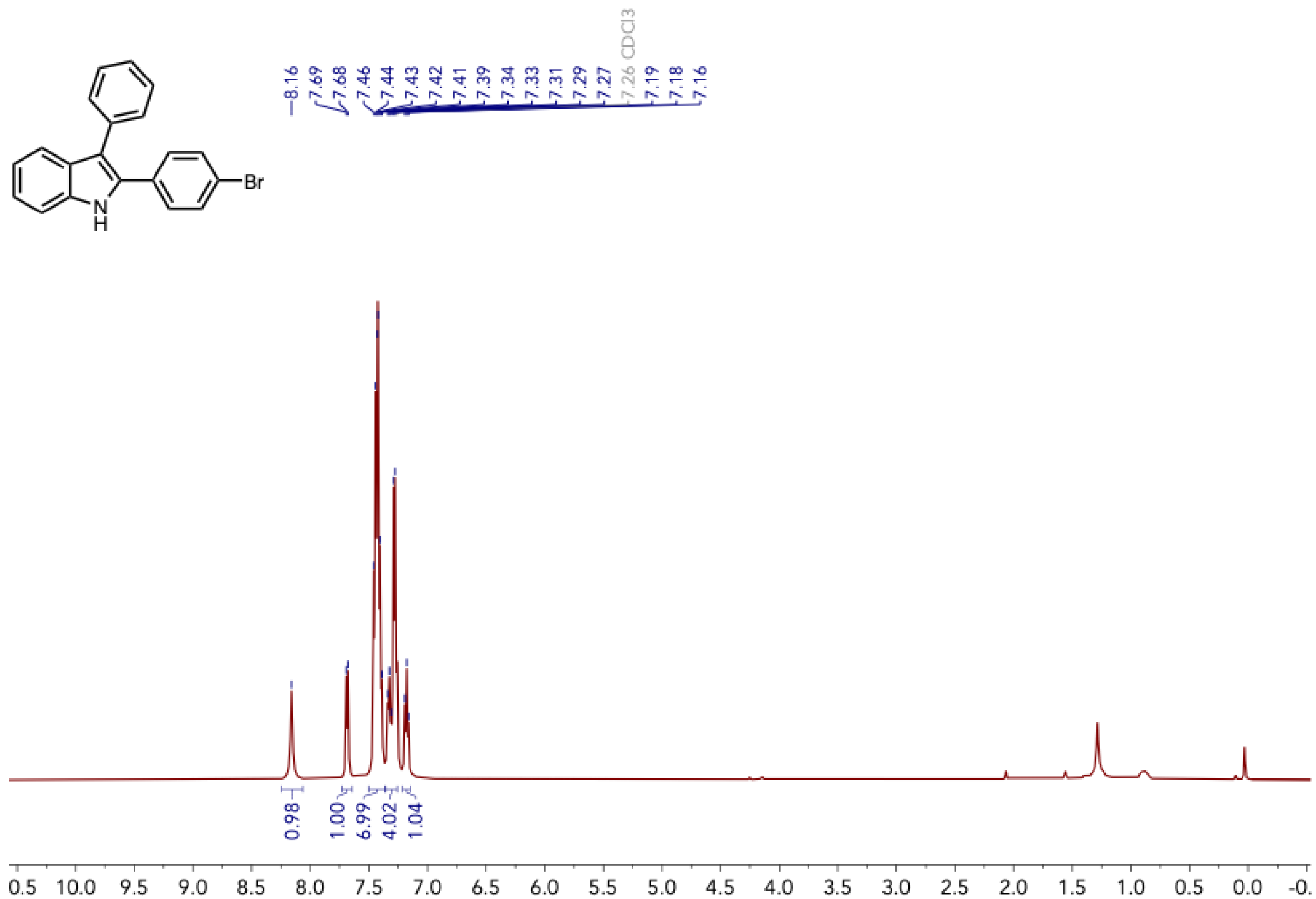

^{1}**H NMR** of **4r** (500 MHz, $CDCl_3$)

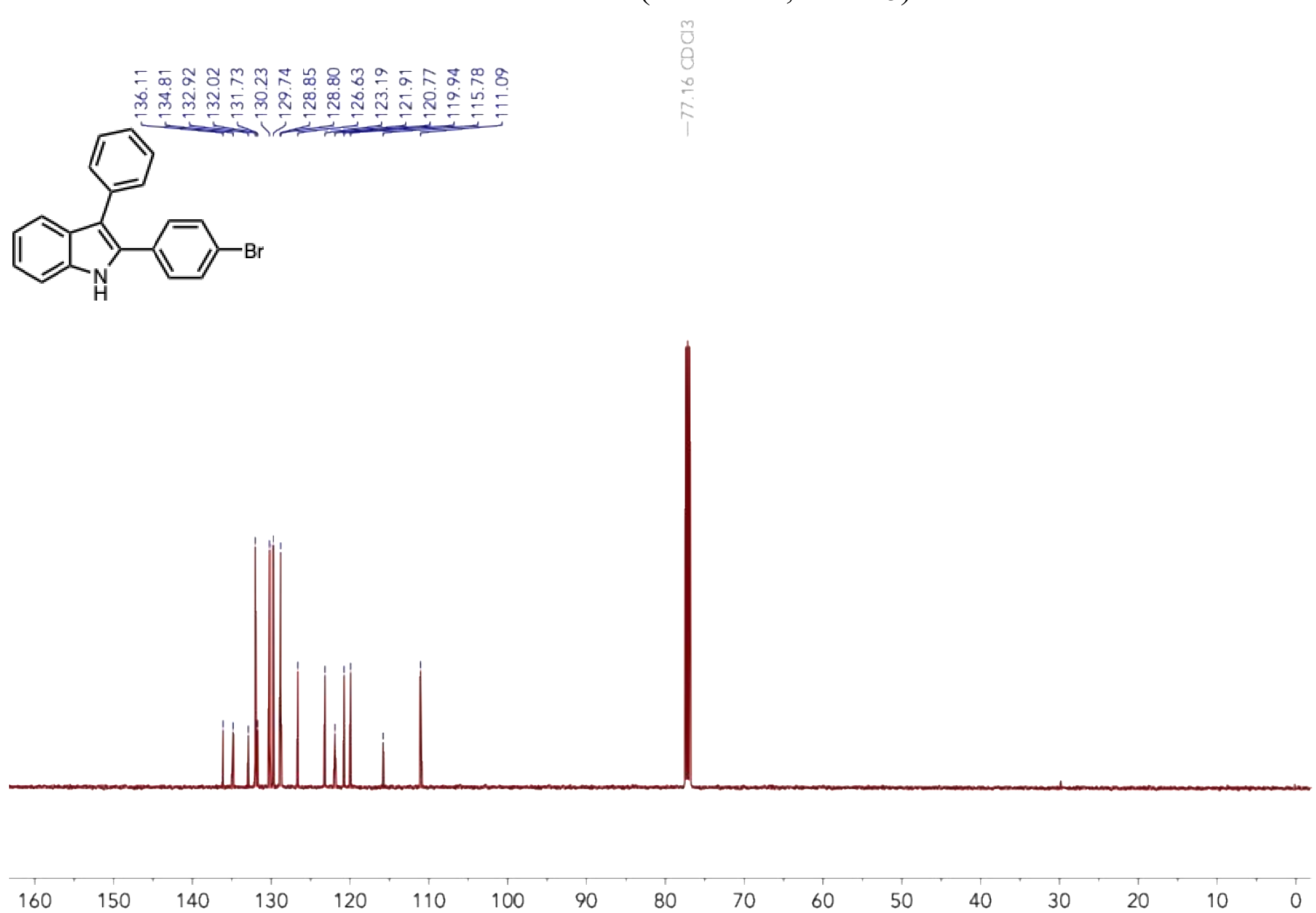

^{13}**C NMR** of **4r** (126 MHz, $CDCl_3$)

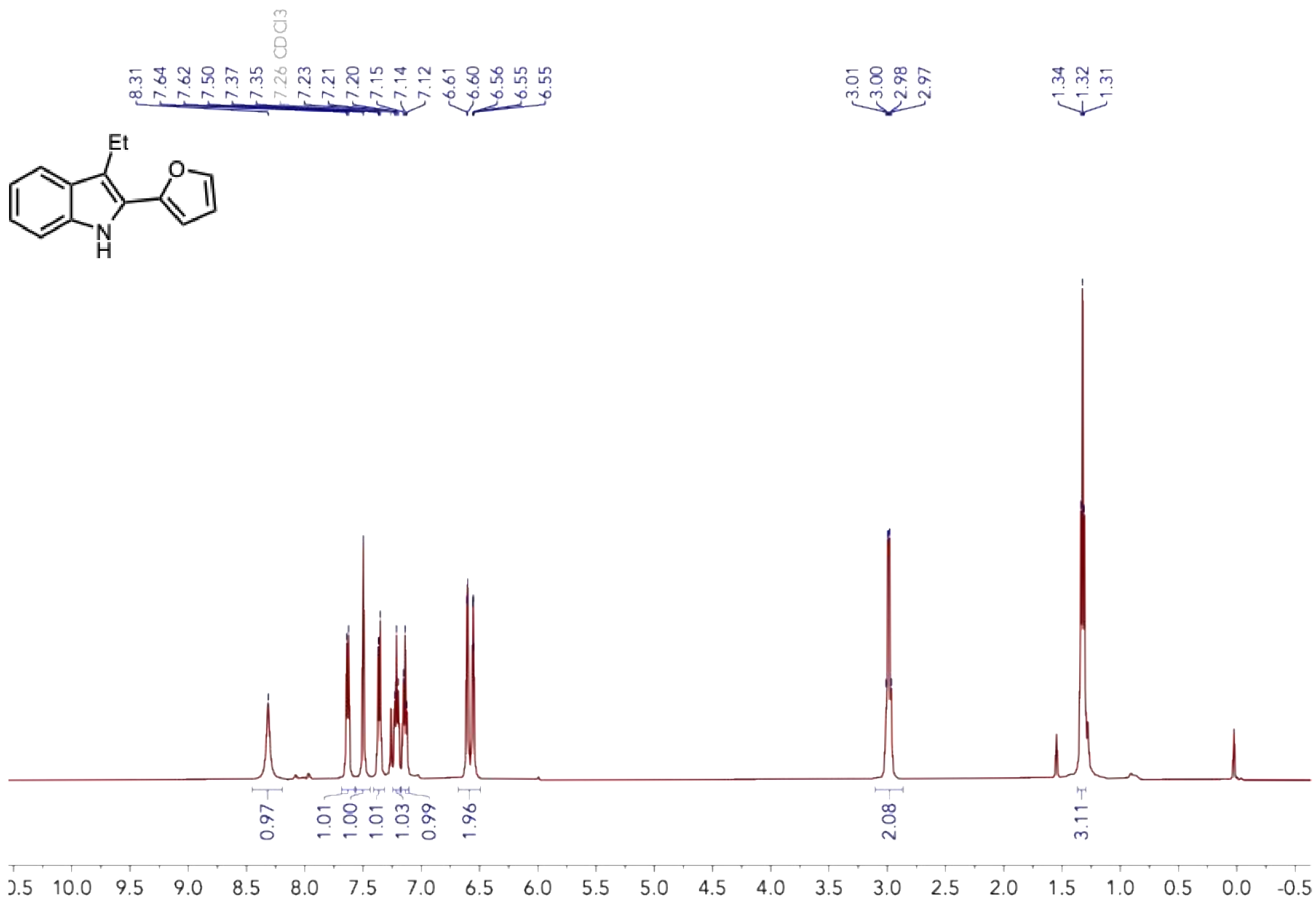

^{1}H NMR of **4s** (500 MHz, $CDCl_3$)

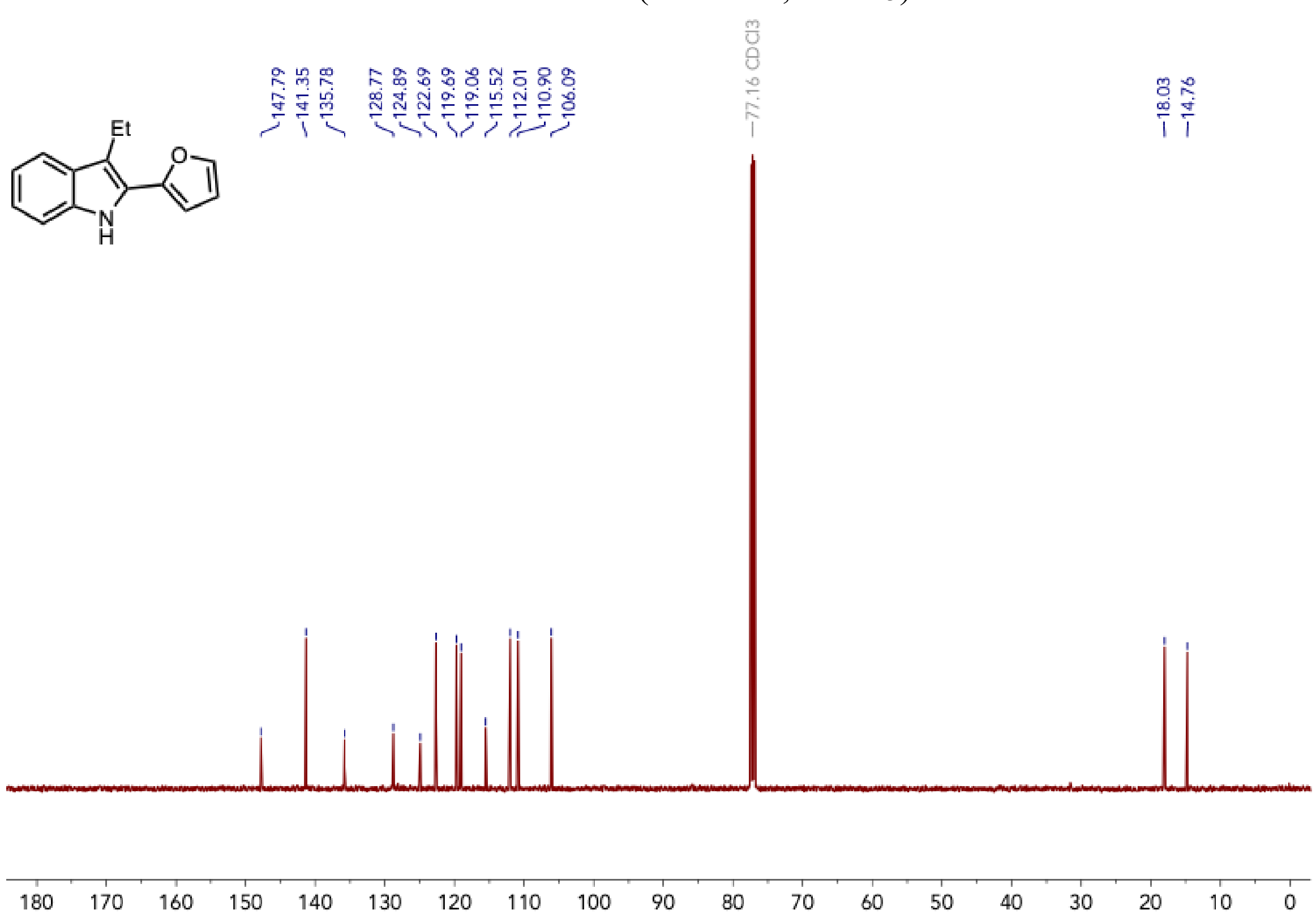

^{13}C NMR of **4s** (126 MHz, $CDCl_3$)

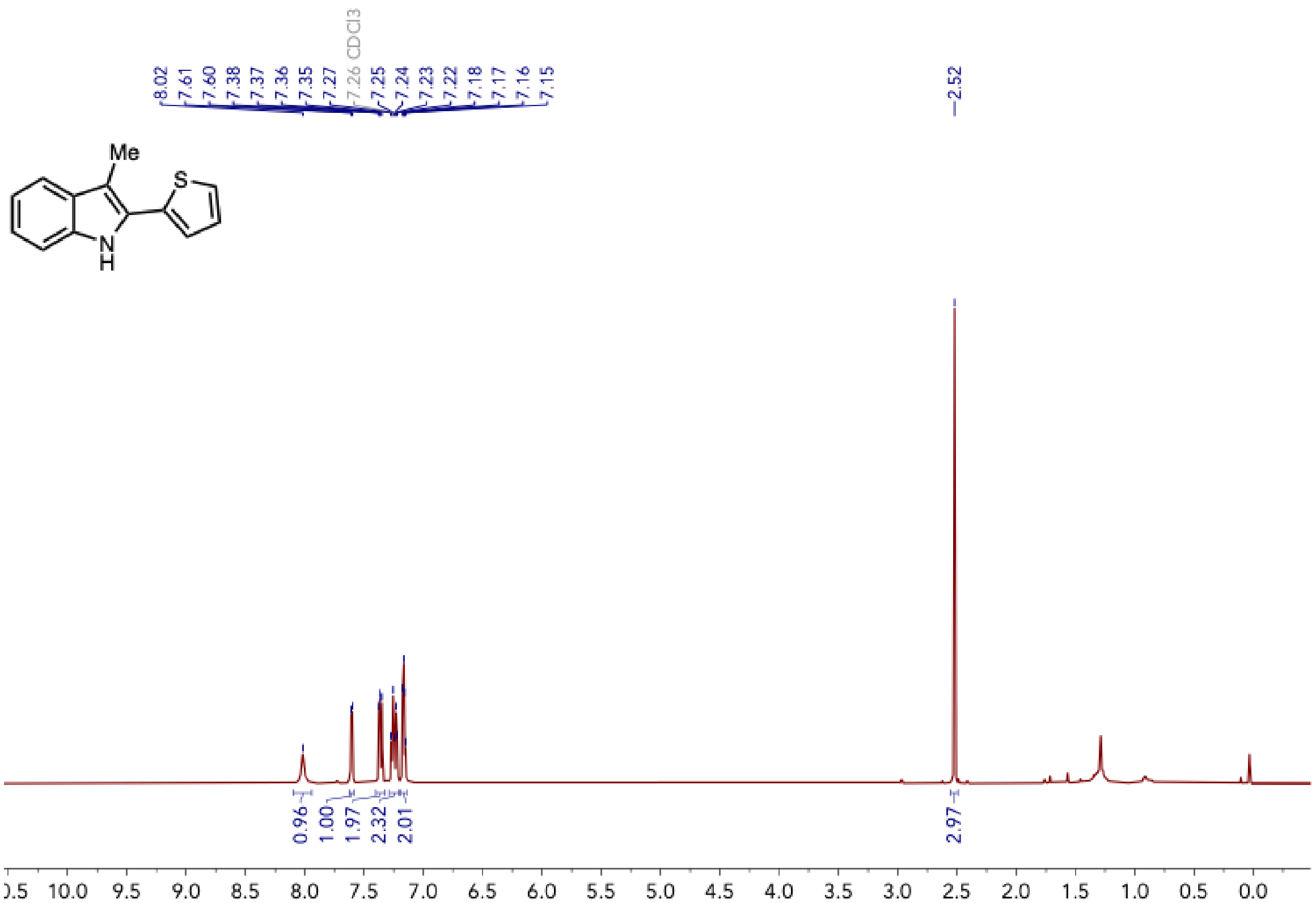

^{1}**H NMR** of **4t** (600 MHz, $CDCl_3$)

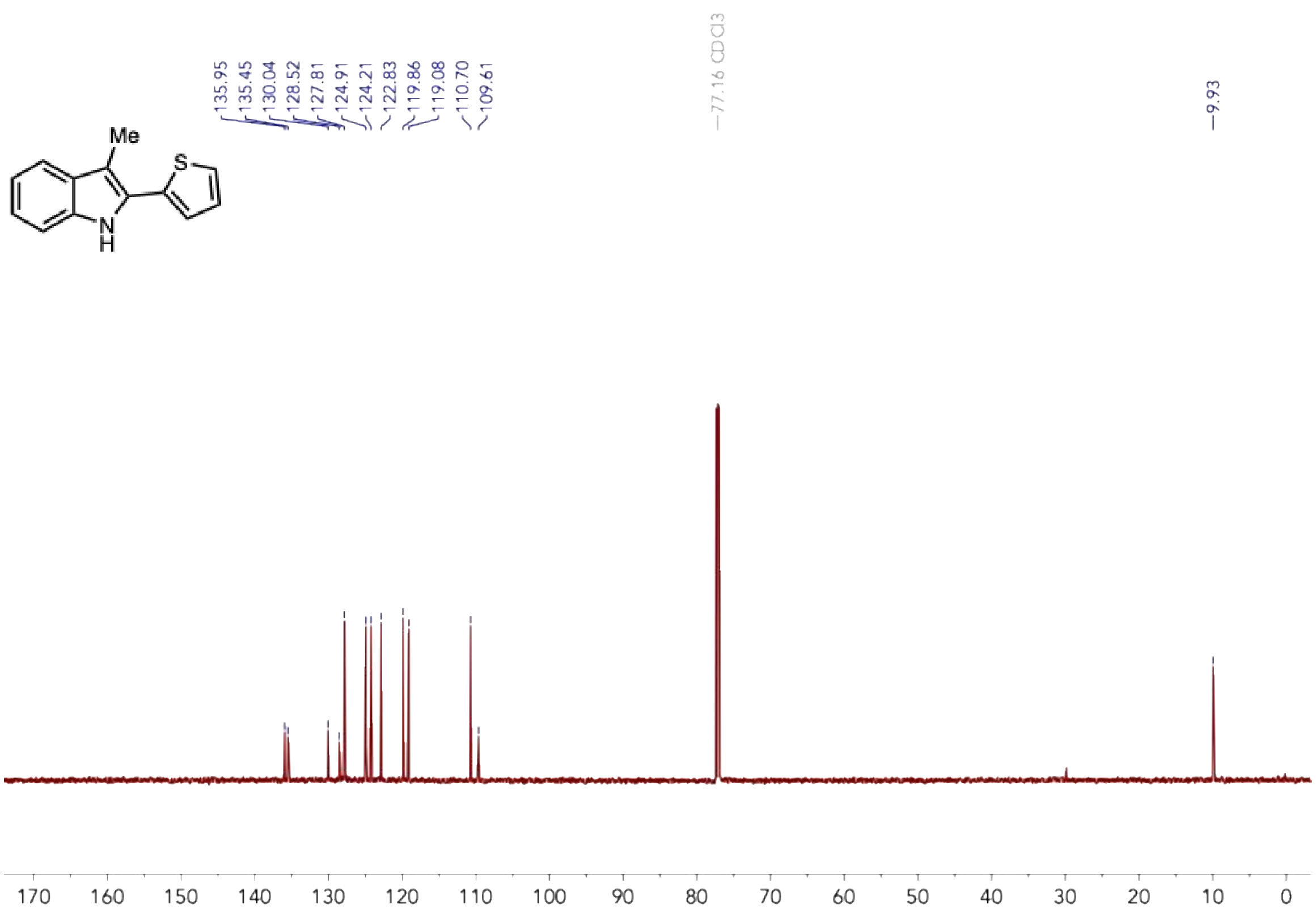

^{13}**C NMR** of **4t** (150 MHz, $CDCl_3$)

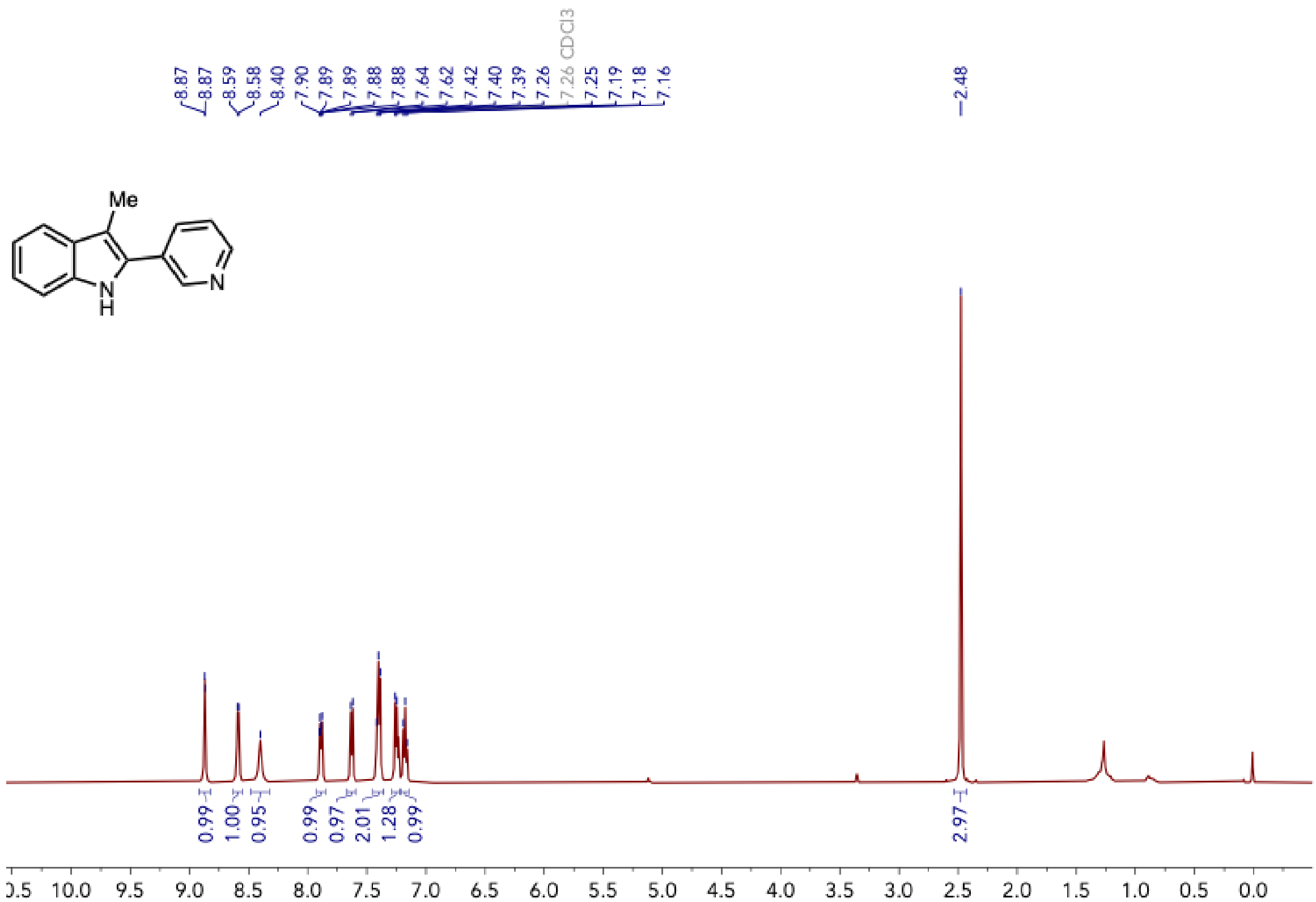

^{1}**H NMR** of **4u** (500 MHz, $CDCl_3$)

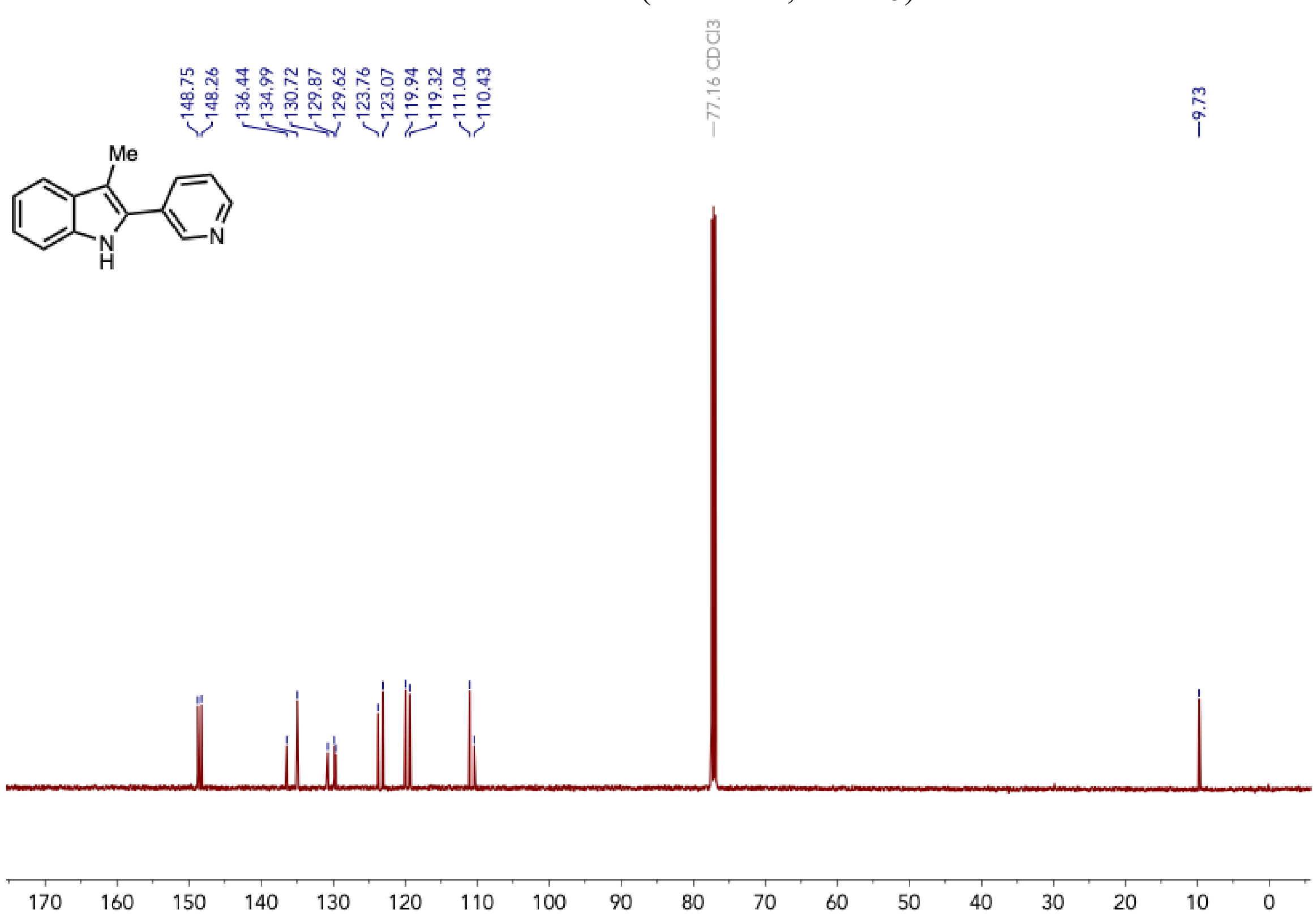

^{13}**C NMR** of **4u** (126 MHz, $CDCl_3$)

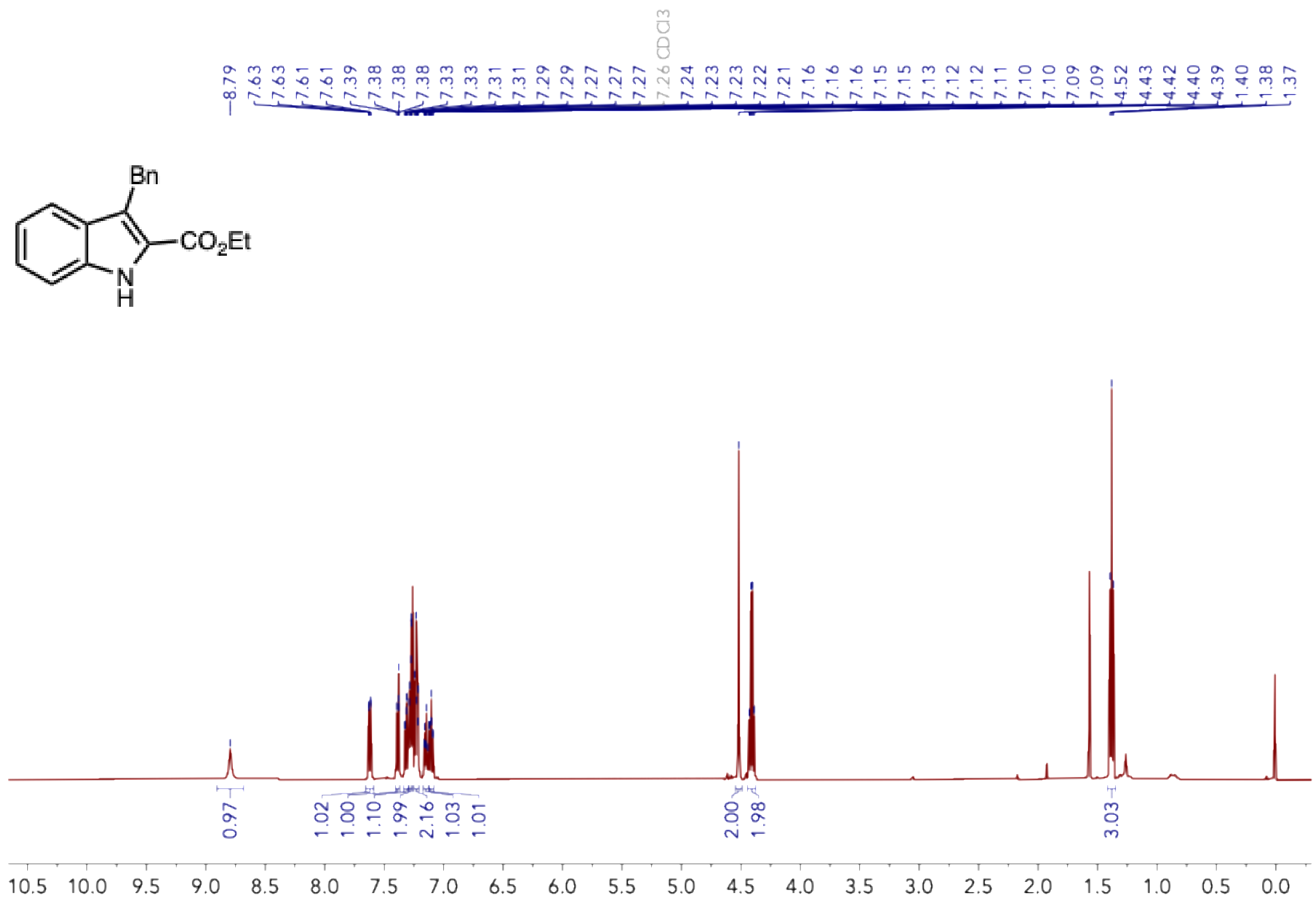

^{1}H NMR of **4v** (500 MHz, $CDCl_3$)

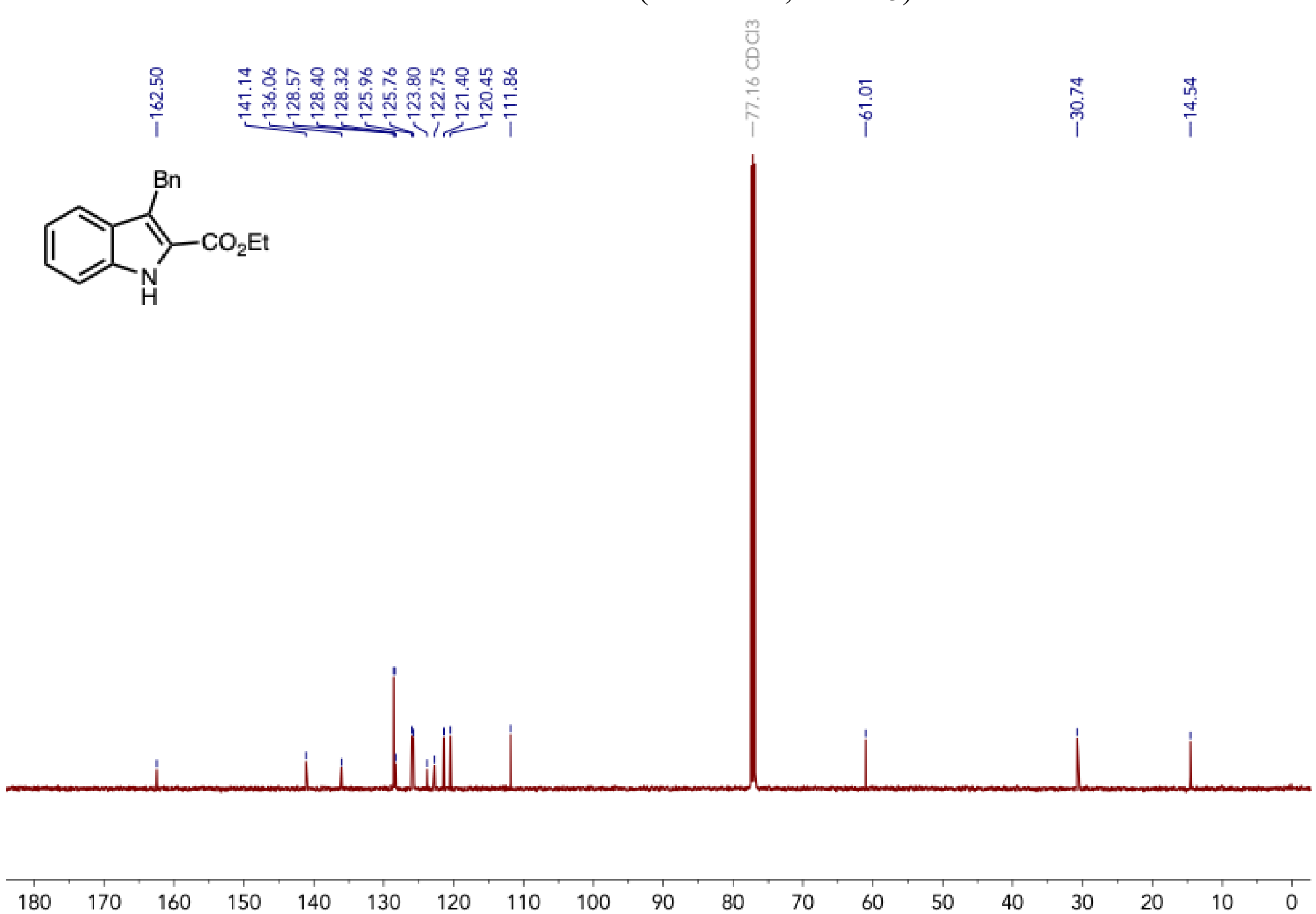

^{13}C NMR of **4v** (126 MHz, $CDCl_3$)

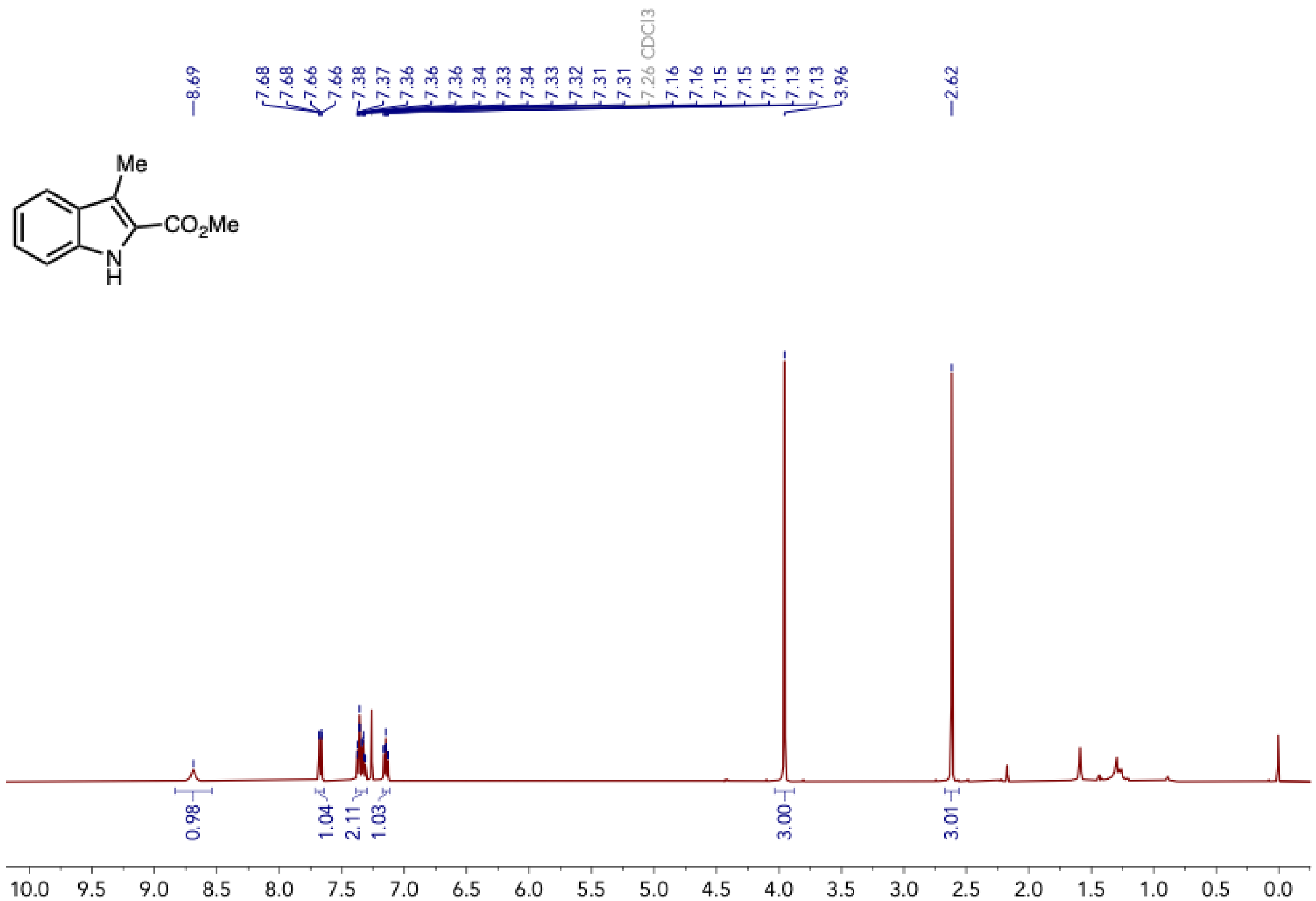

^{1}H NMR of **4w** (500 MHz, $CDCl_3$)

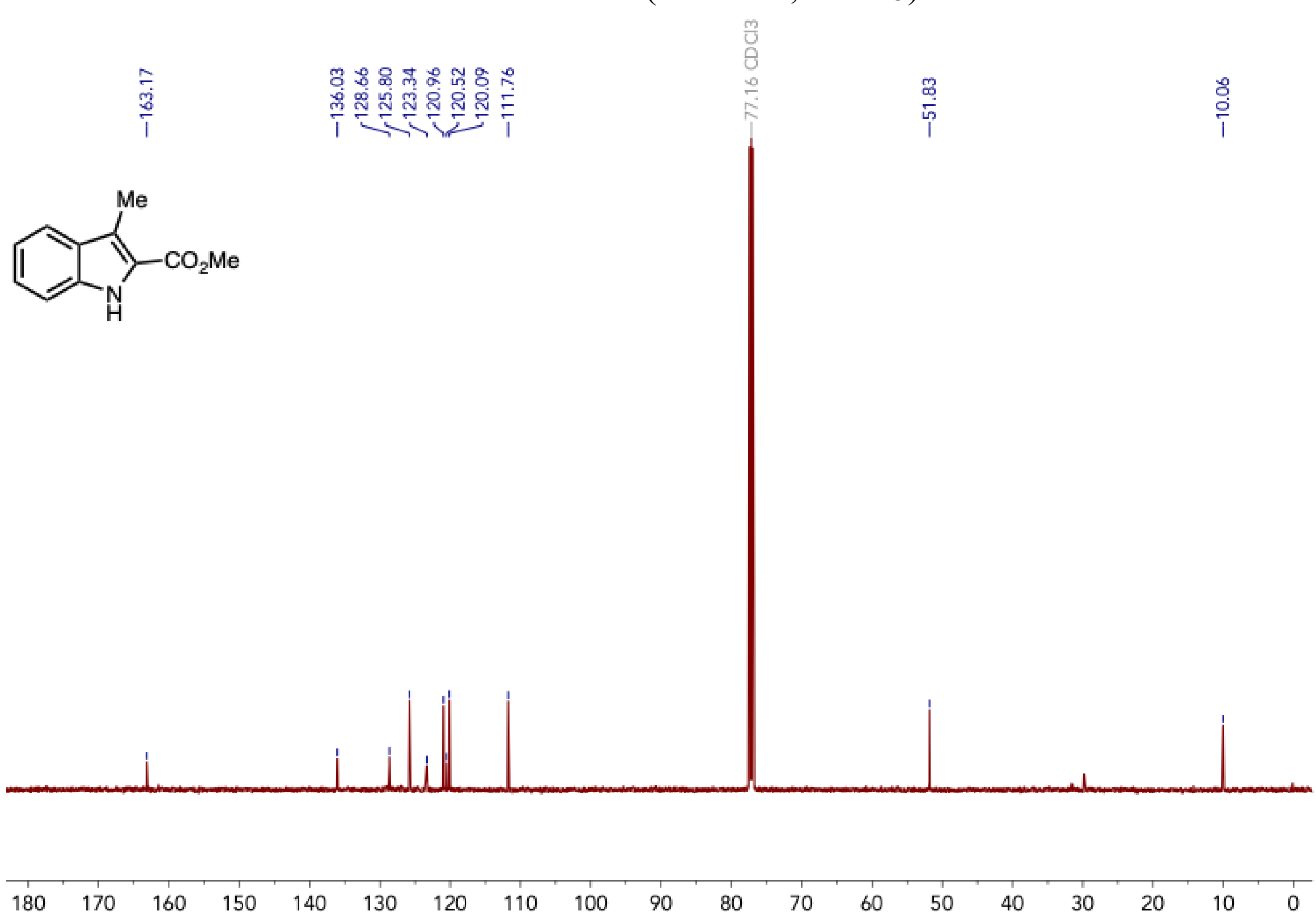

^{13}C NMR of **4w** (126 MHz, $CDCl_3$)

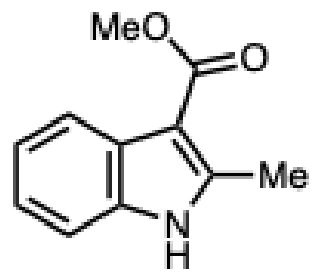

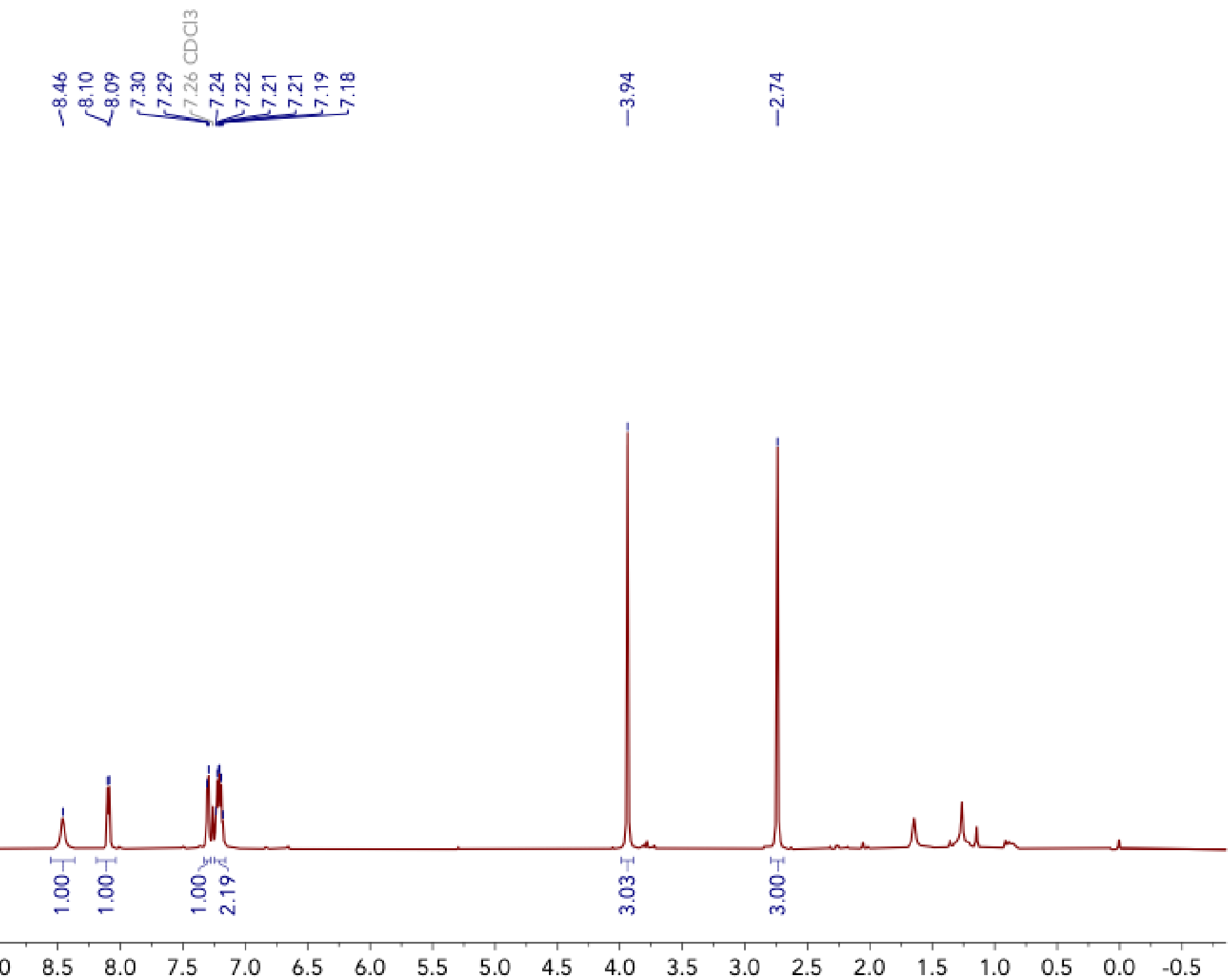

^{1}H NMR of **6a** (600 MHz, $CDCl_3$)

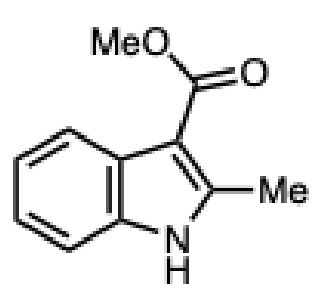

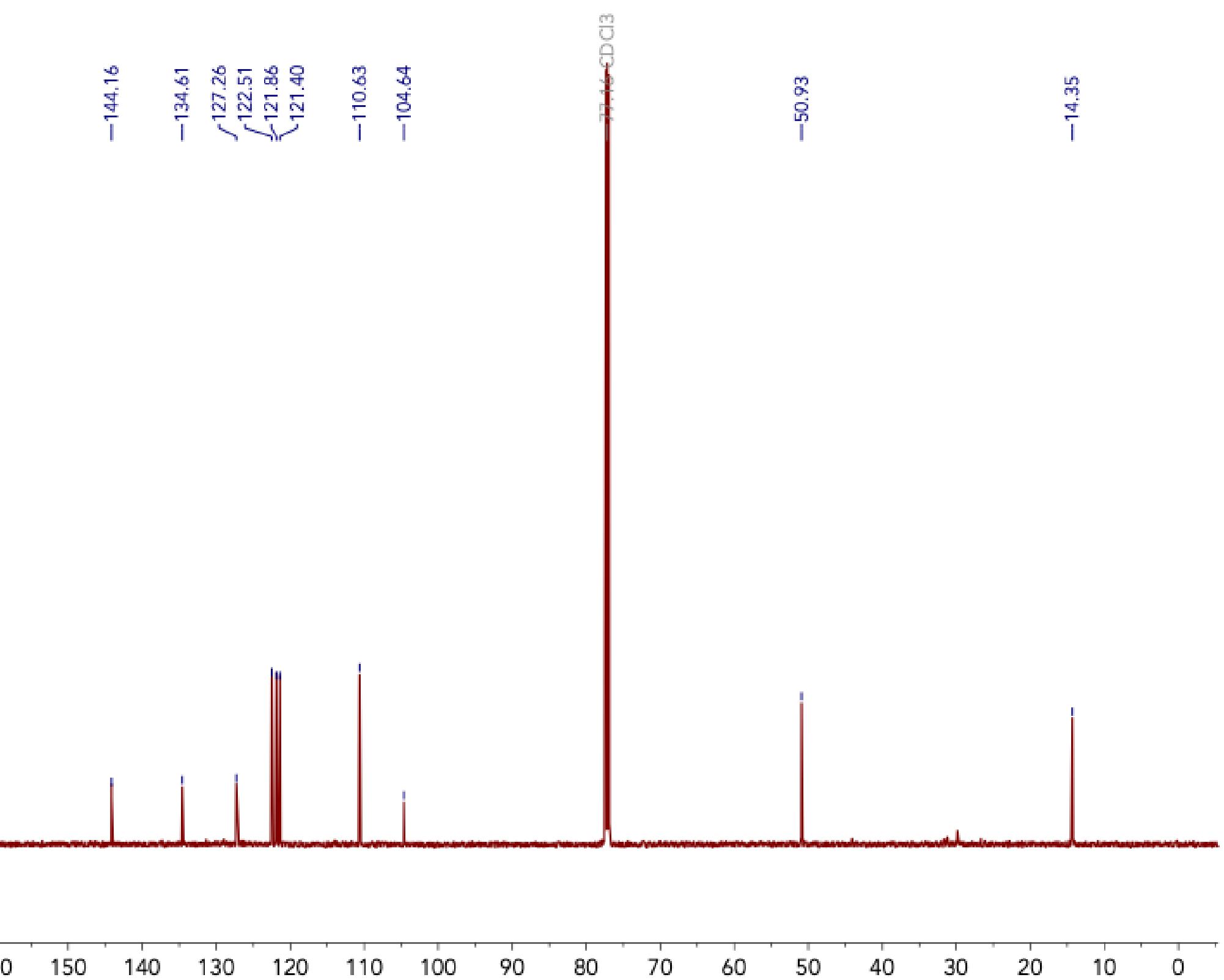

^{13}C NMR of **6a** (150 MHz, $CDCl_3$)

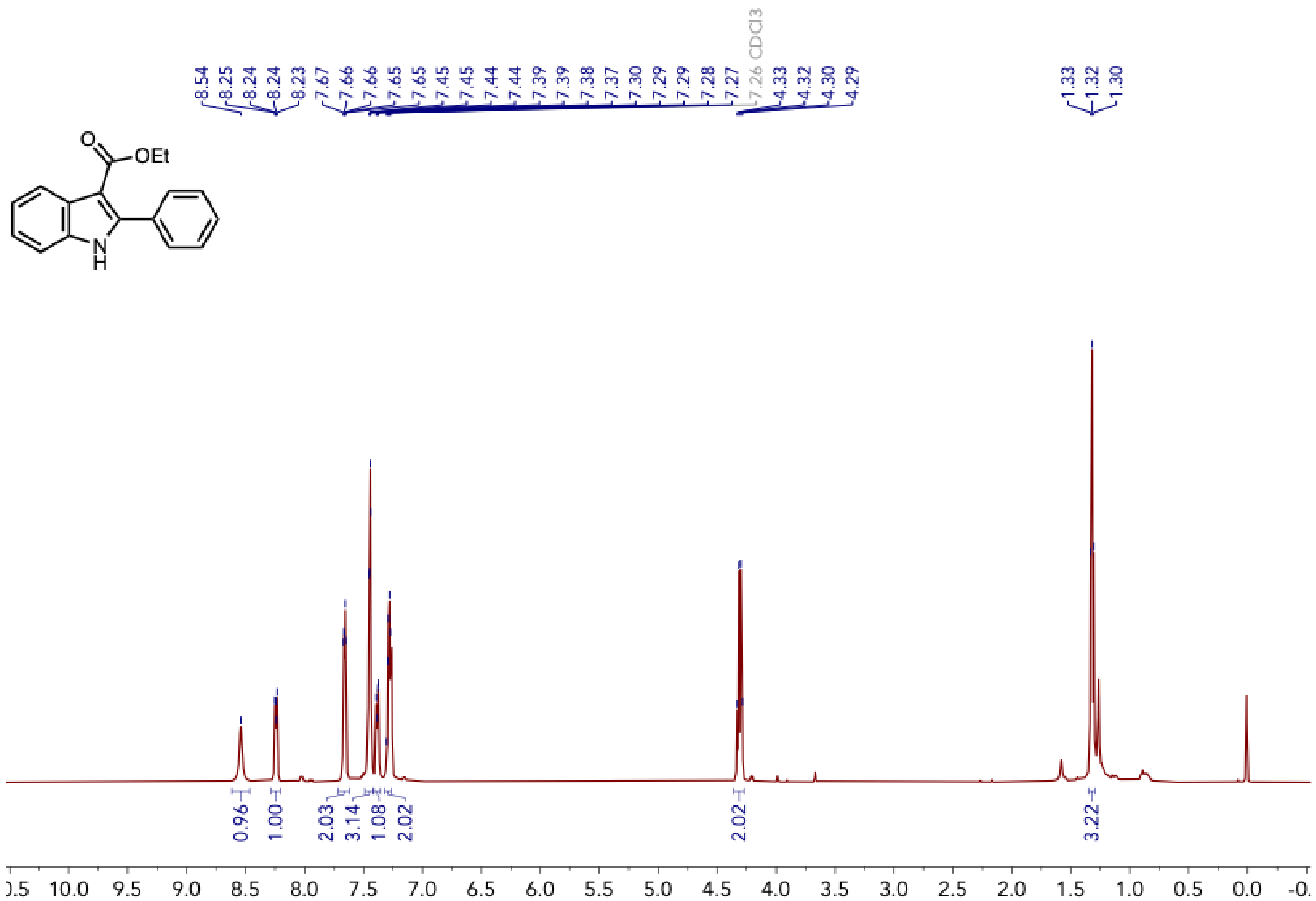

^{1}**H NMR** of **6b** (500 MHz, CDCl$_3$)

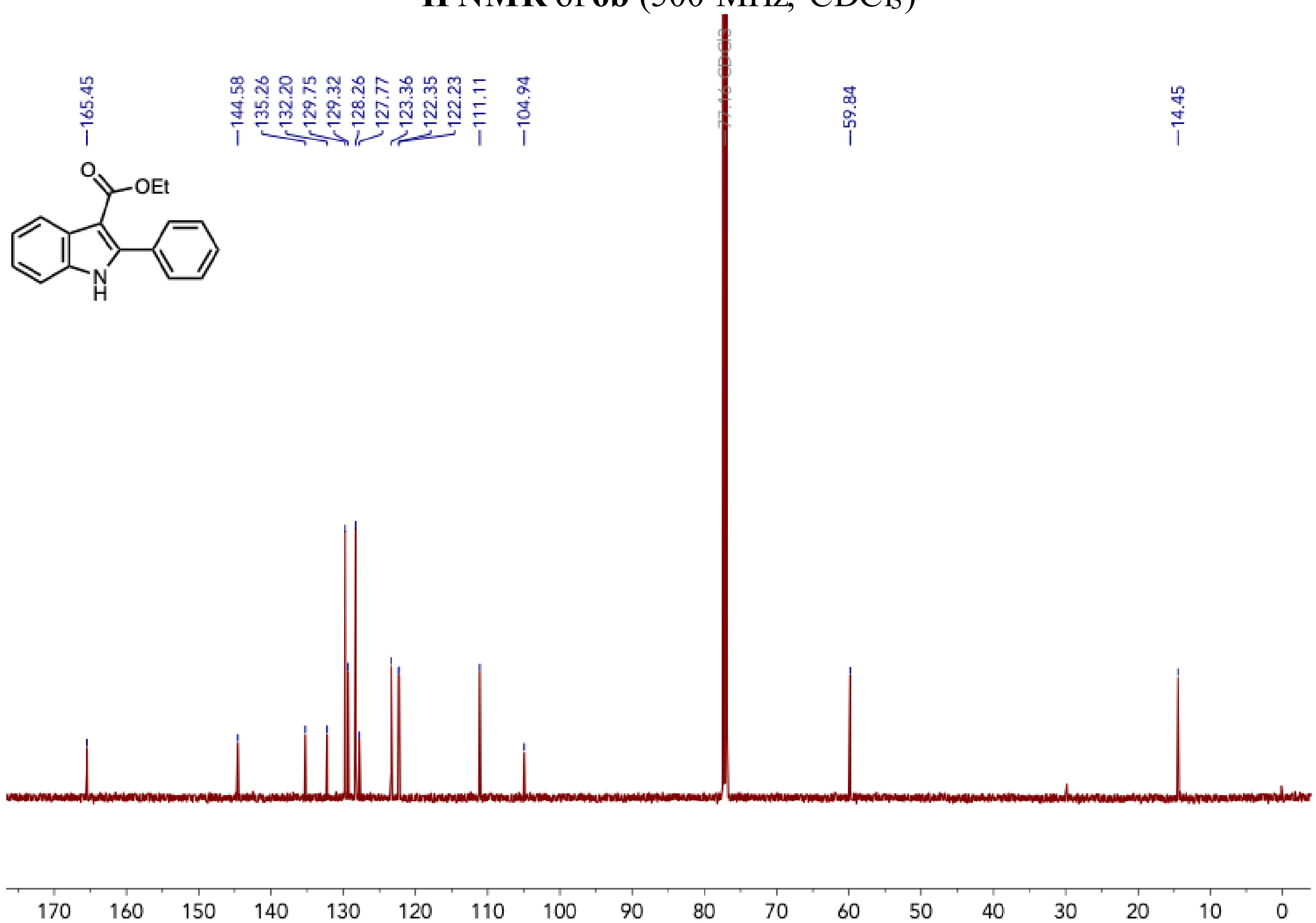

^{13}**C NMR** of **6b** (126 MHz, CDCl$_3$)

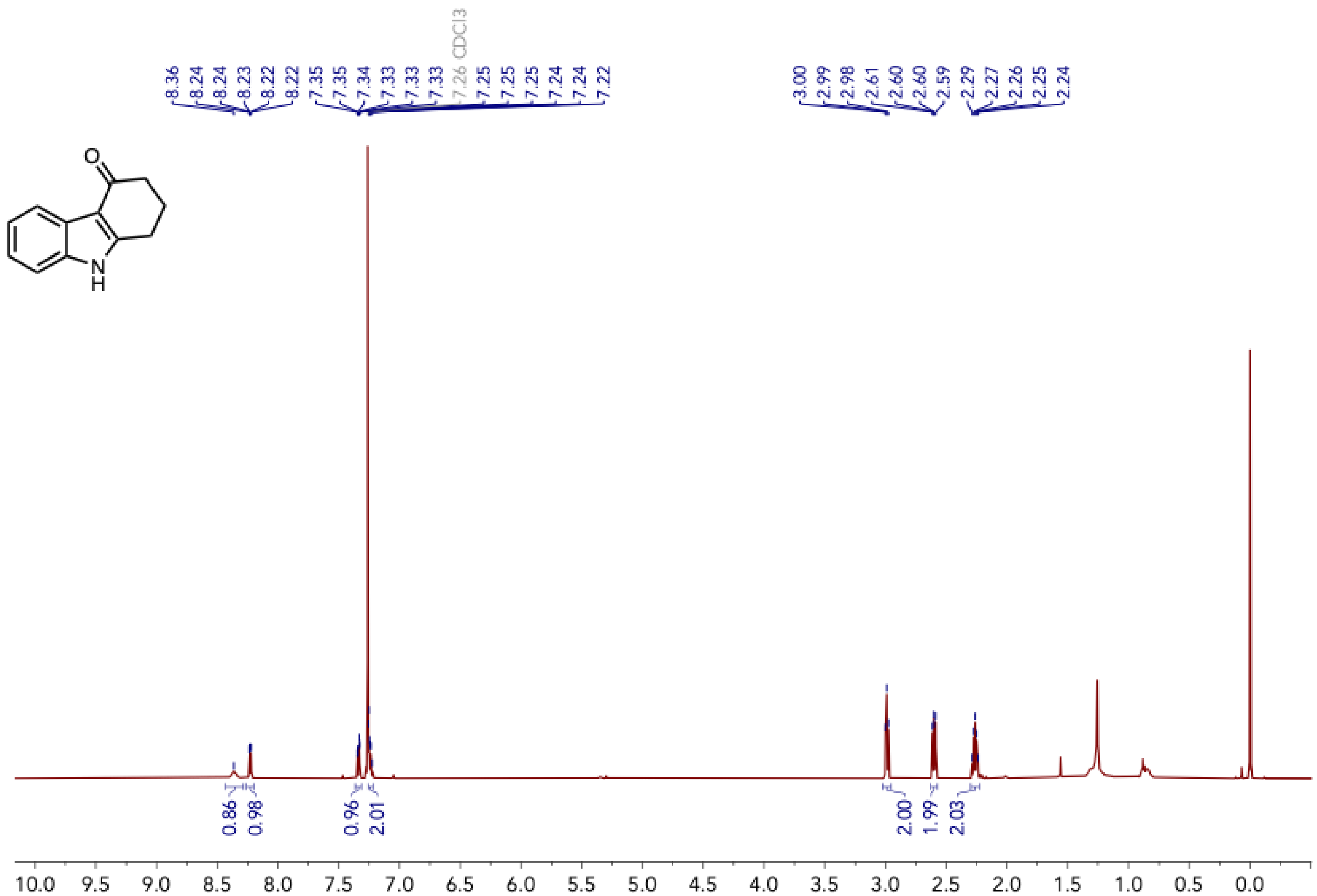

^{1}H NMR of **6c** (500 MHz, $CDCl_3$)

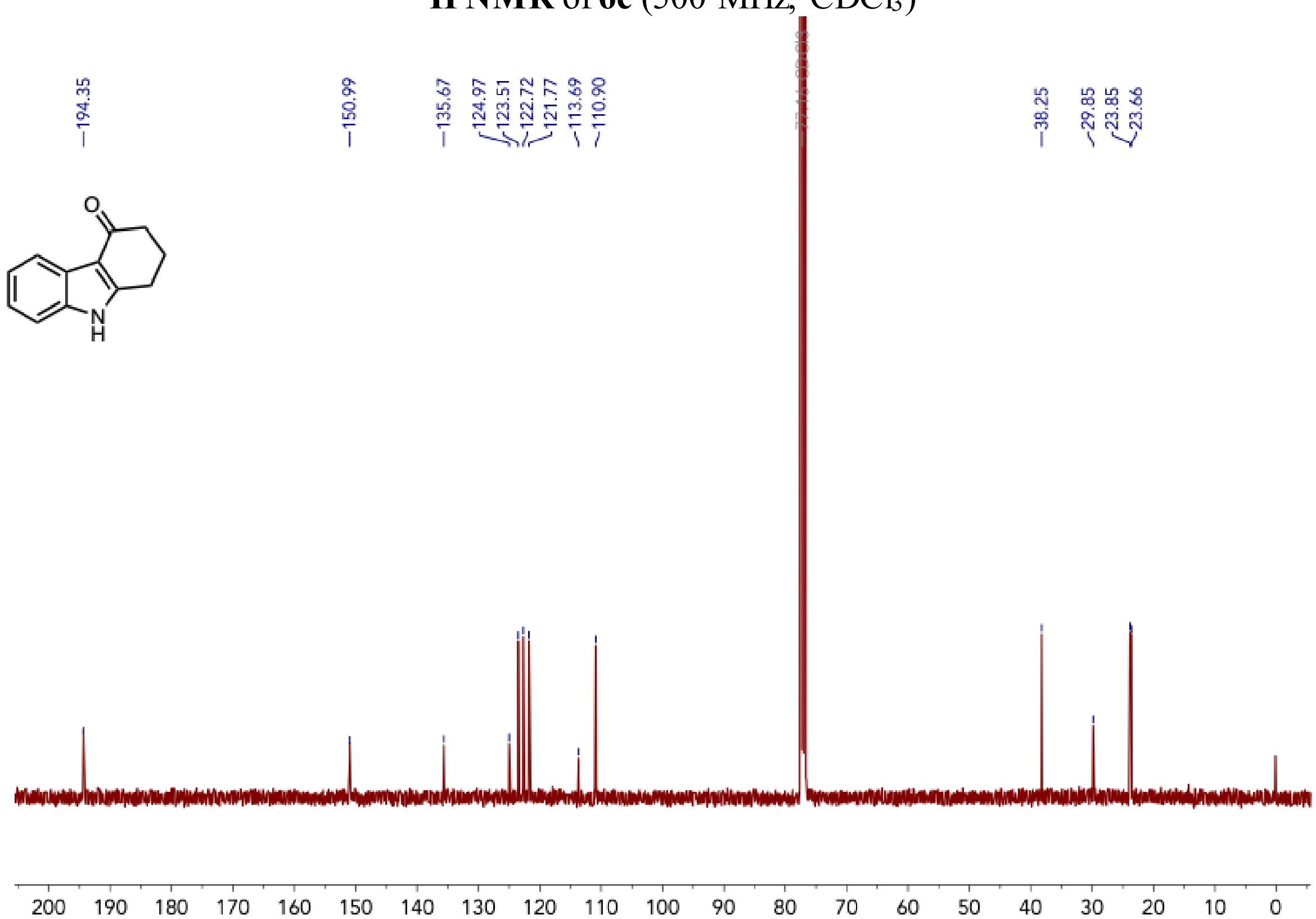

^{13}C NMR of **6c** (126 MHz, $CDCl_3$)

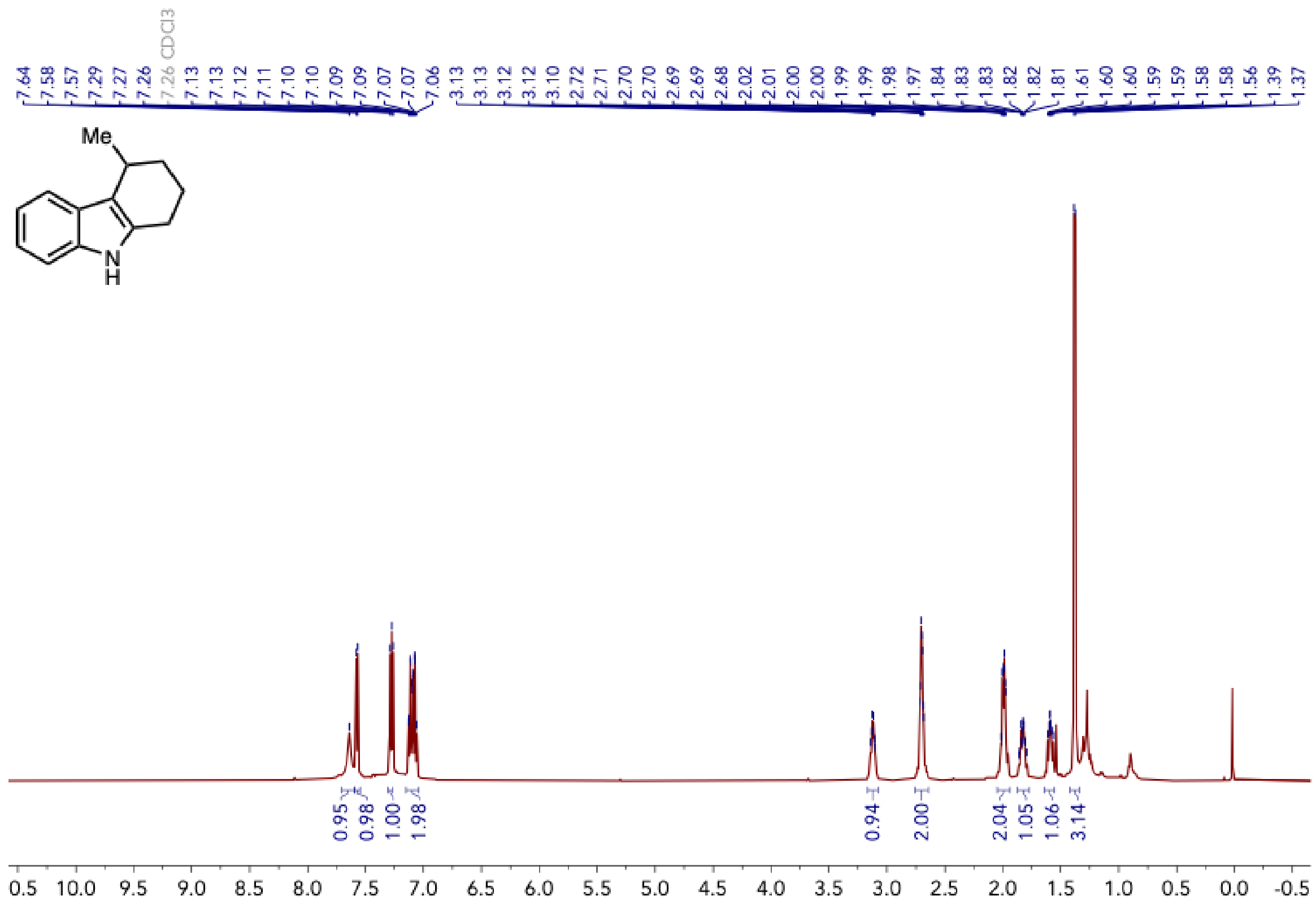

^{1}H NMR of **8a** (500 MHz, $CDCl_3$)

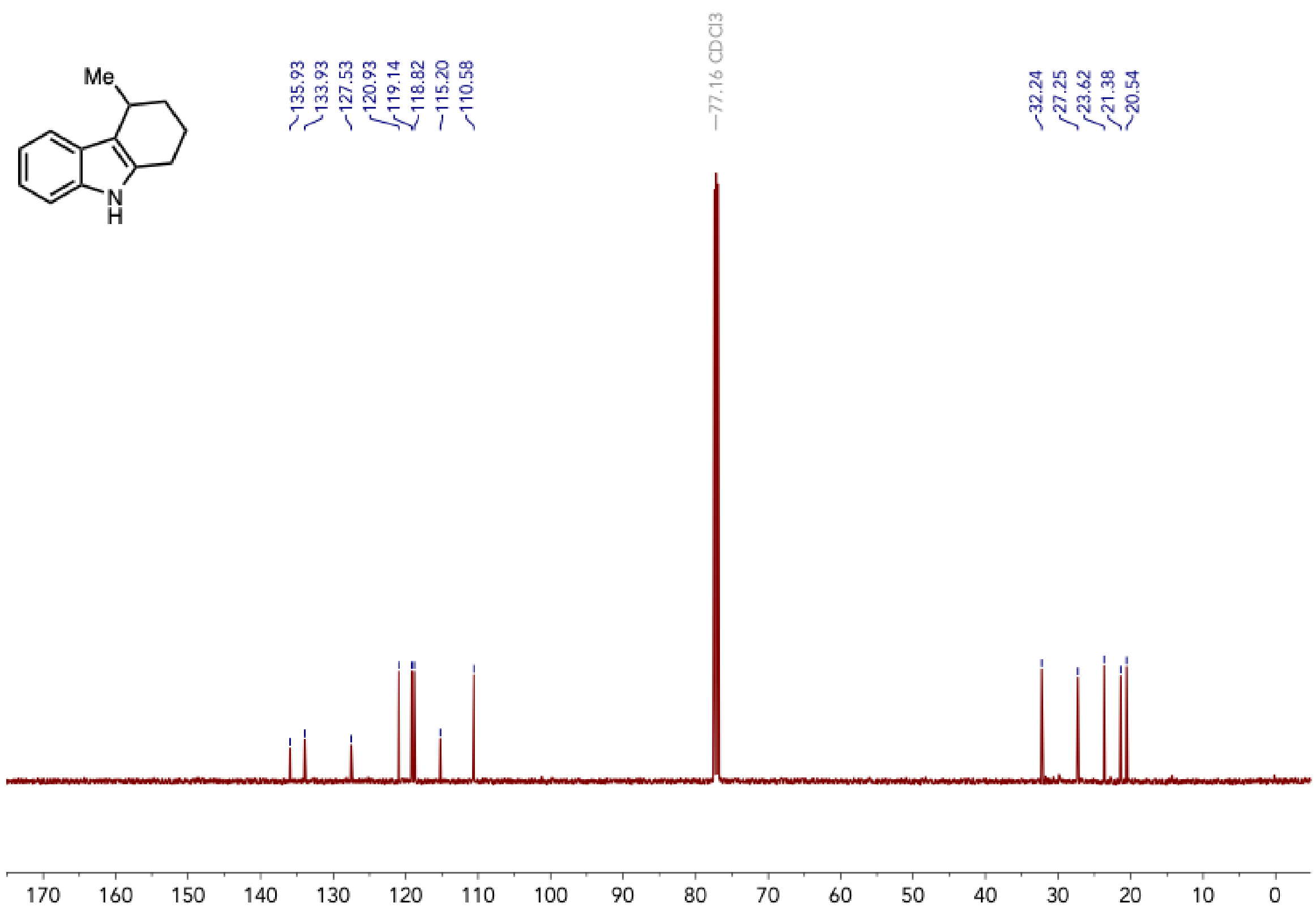

^{13}C NMR of **8a** (126 MHz, $CDCl_3$)

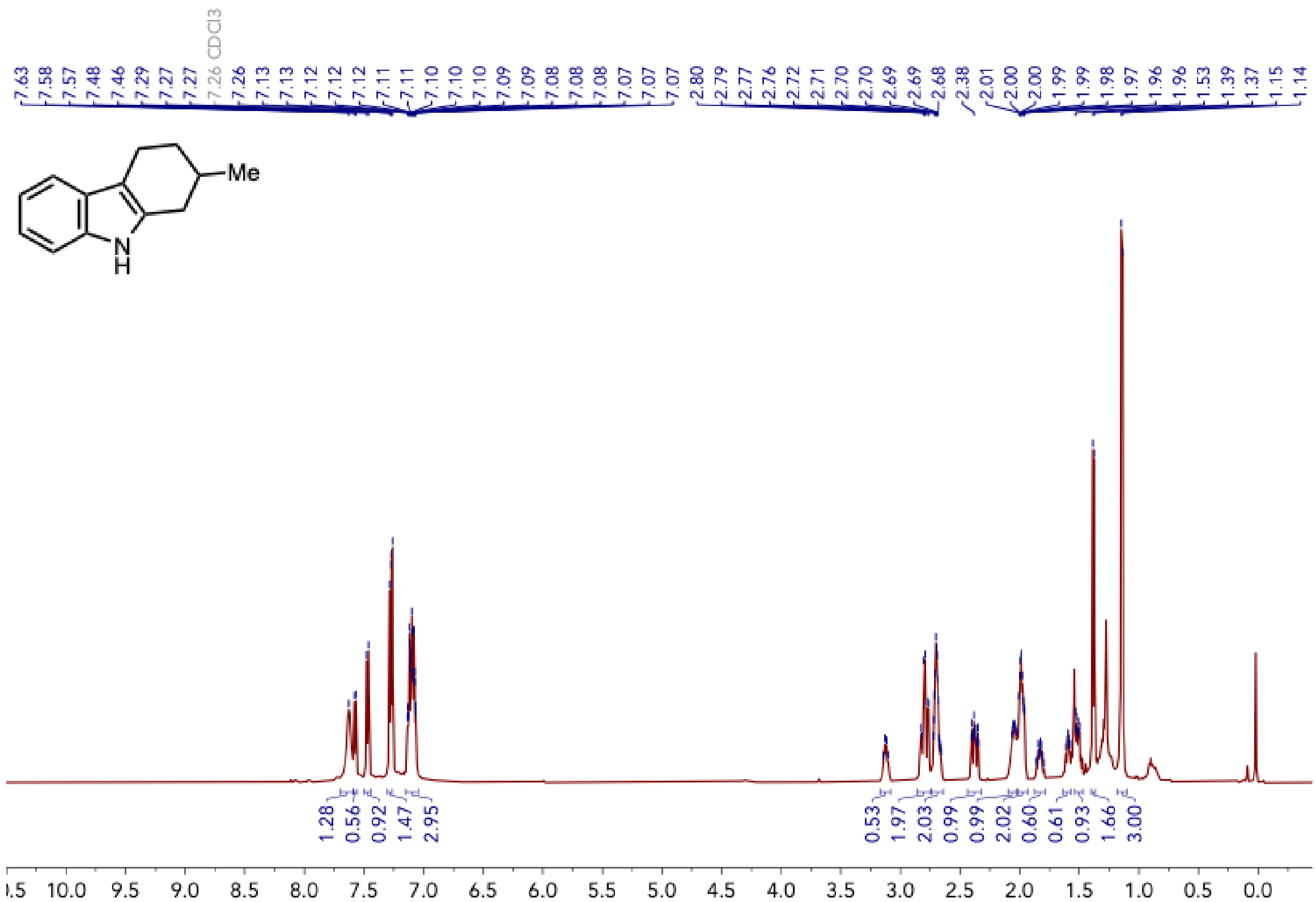

^{1}H NMR of **8a'** (500 MHz, $CDCl_3$)

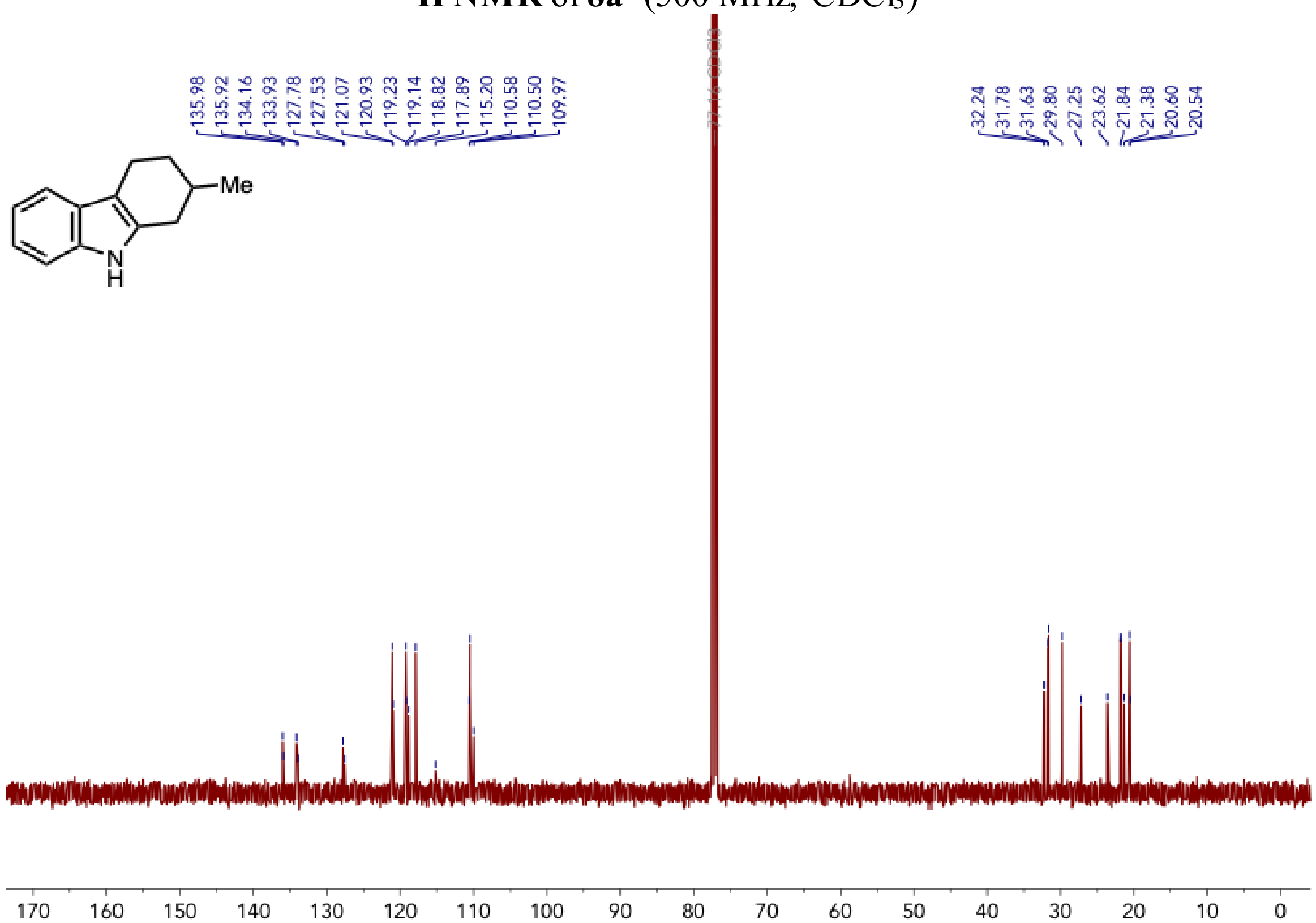

^{13}C NMR of **8a'** (126 MHz, $CDCl_3$)

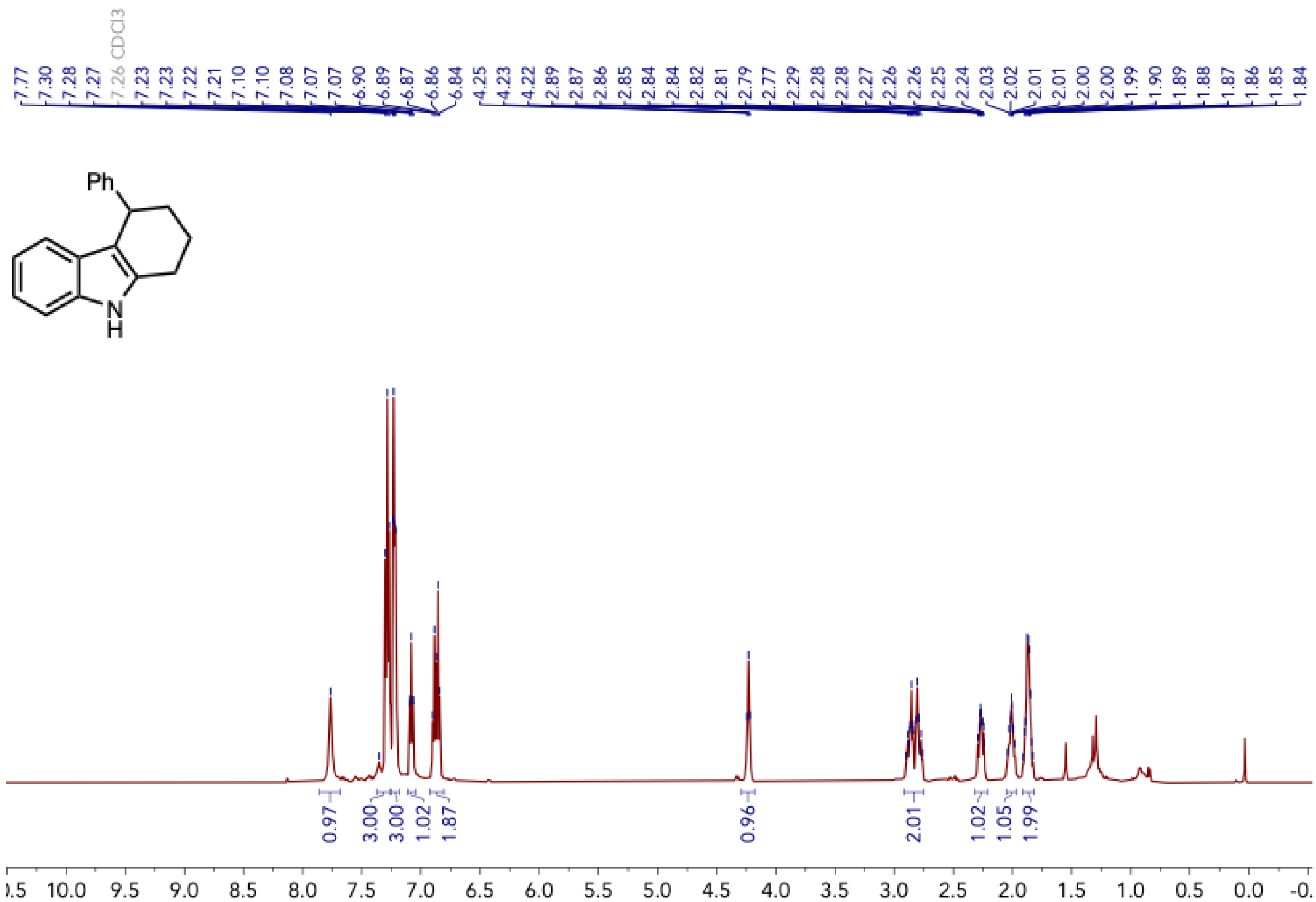

^{1}**H NMR** of **8c** (500 MHz, $CDCl_3$)

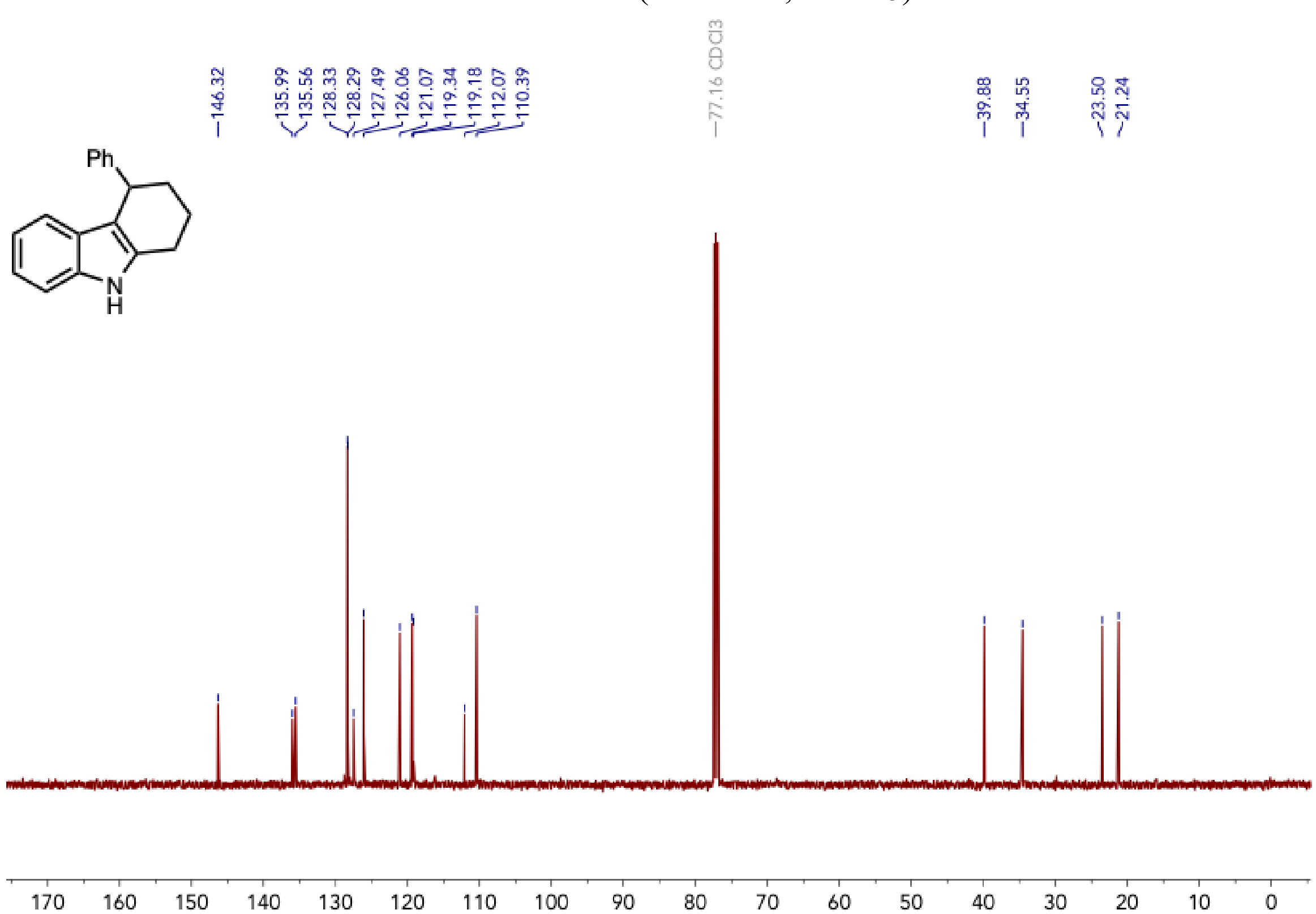

^{13}**C NMR** of **8c** (126 MHz, $CDCl_3$)

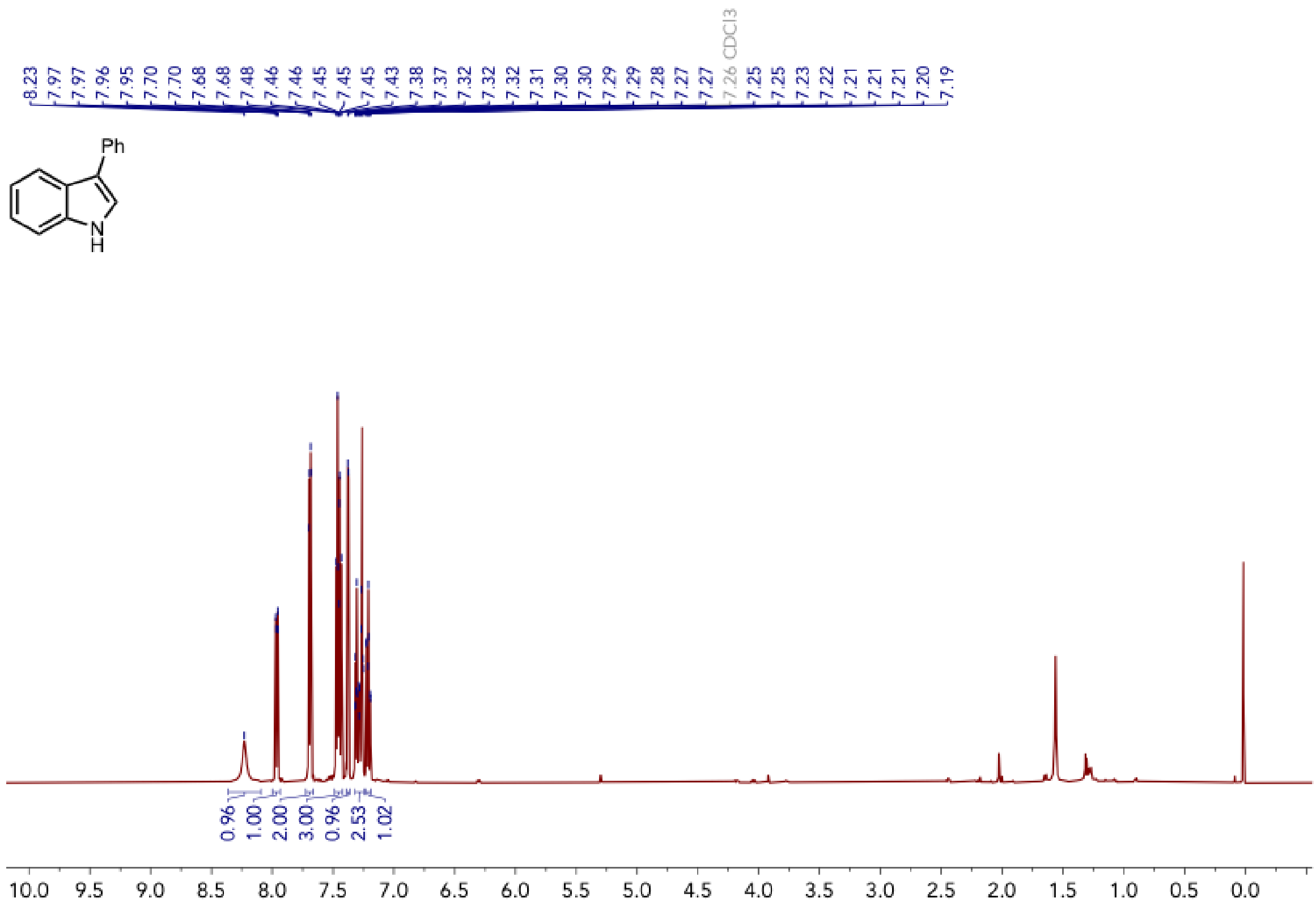

¹H NMR of **8d** (500 MHz, $CDCl_3$)

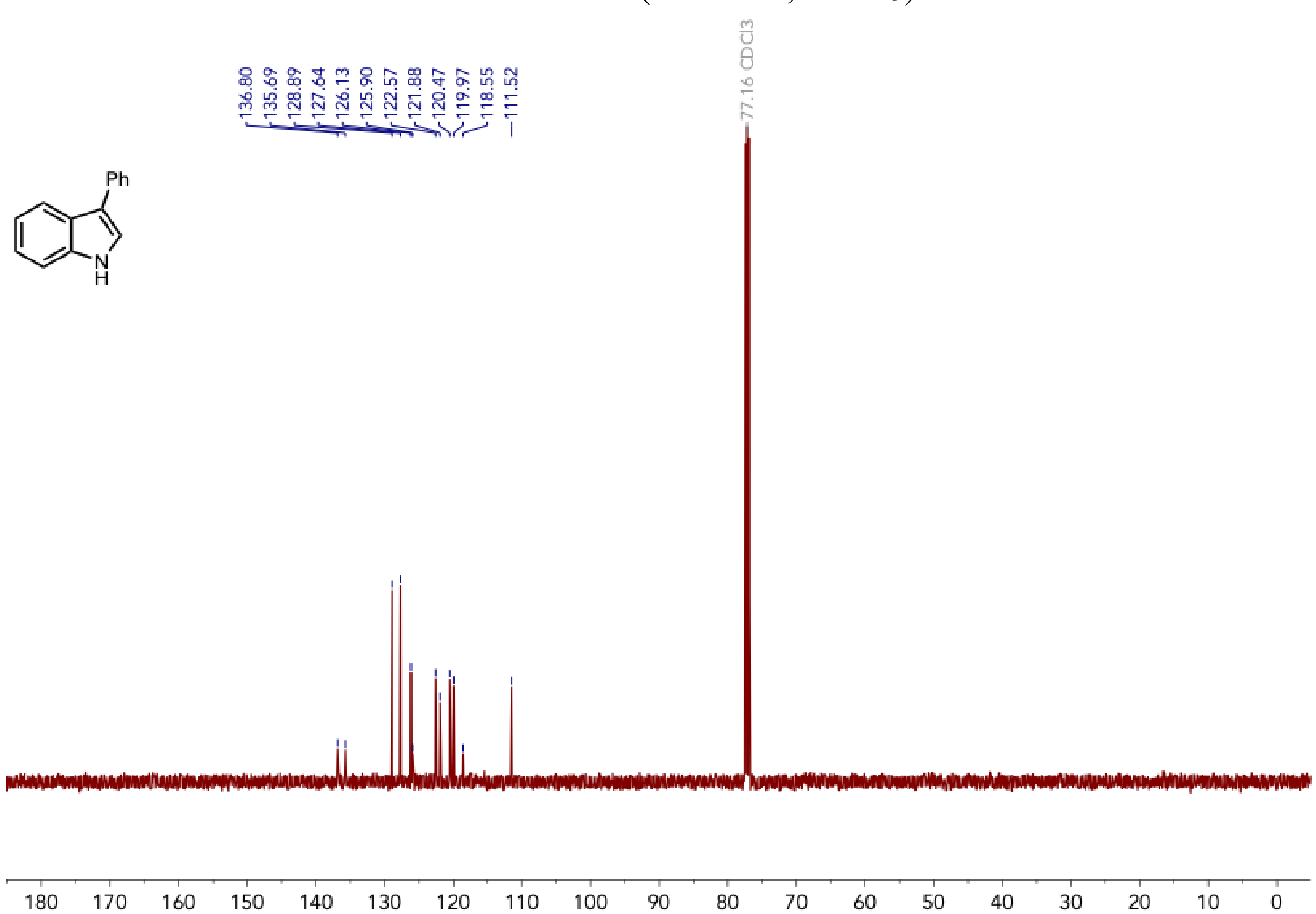

¹³C NMR of **8d** (126 MHz, $CDCl_3$)

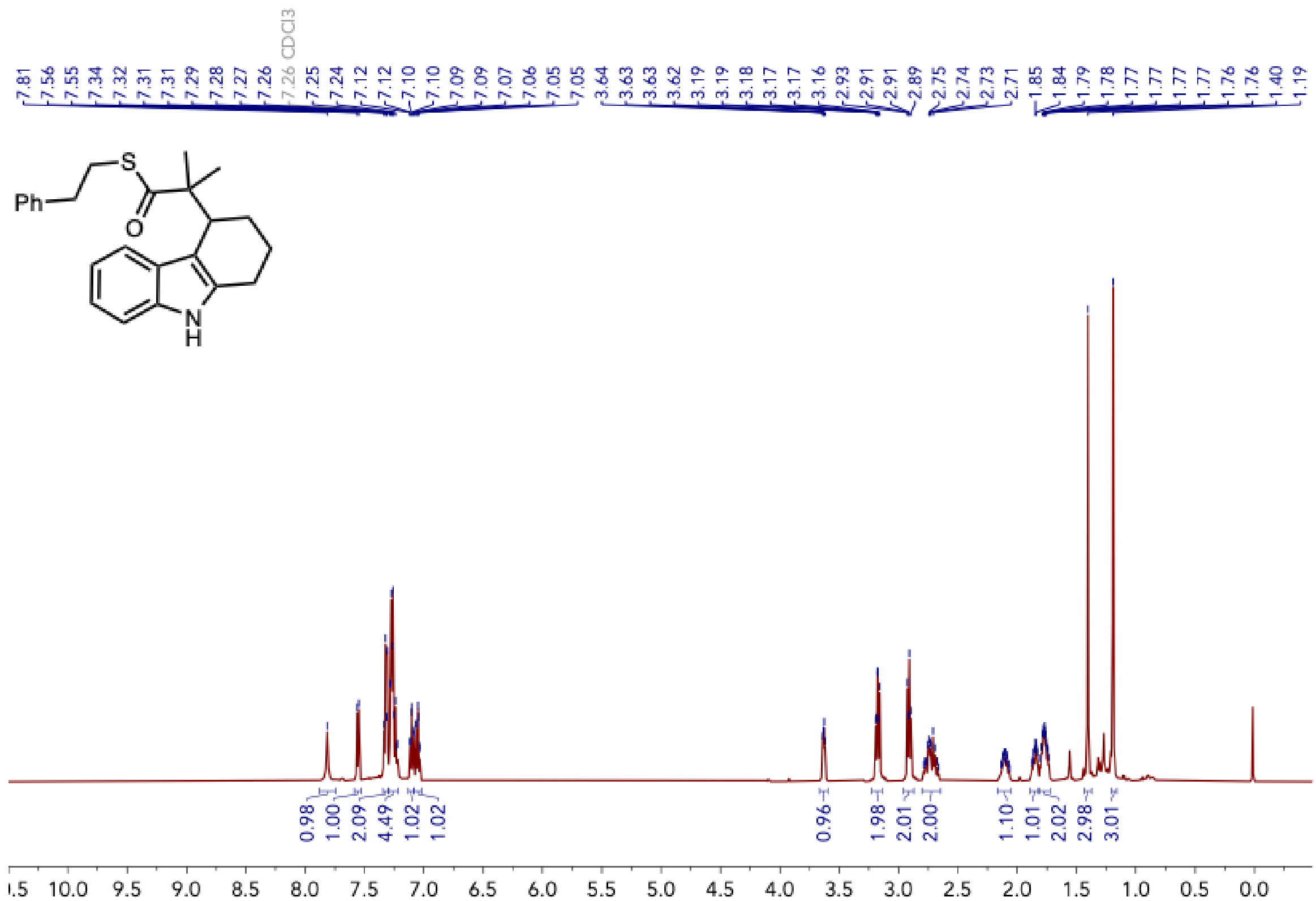

^{1}**H NMR** of **10c** (500 MHz, $CDCl_3$)

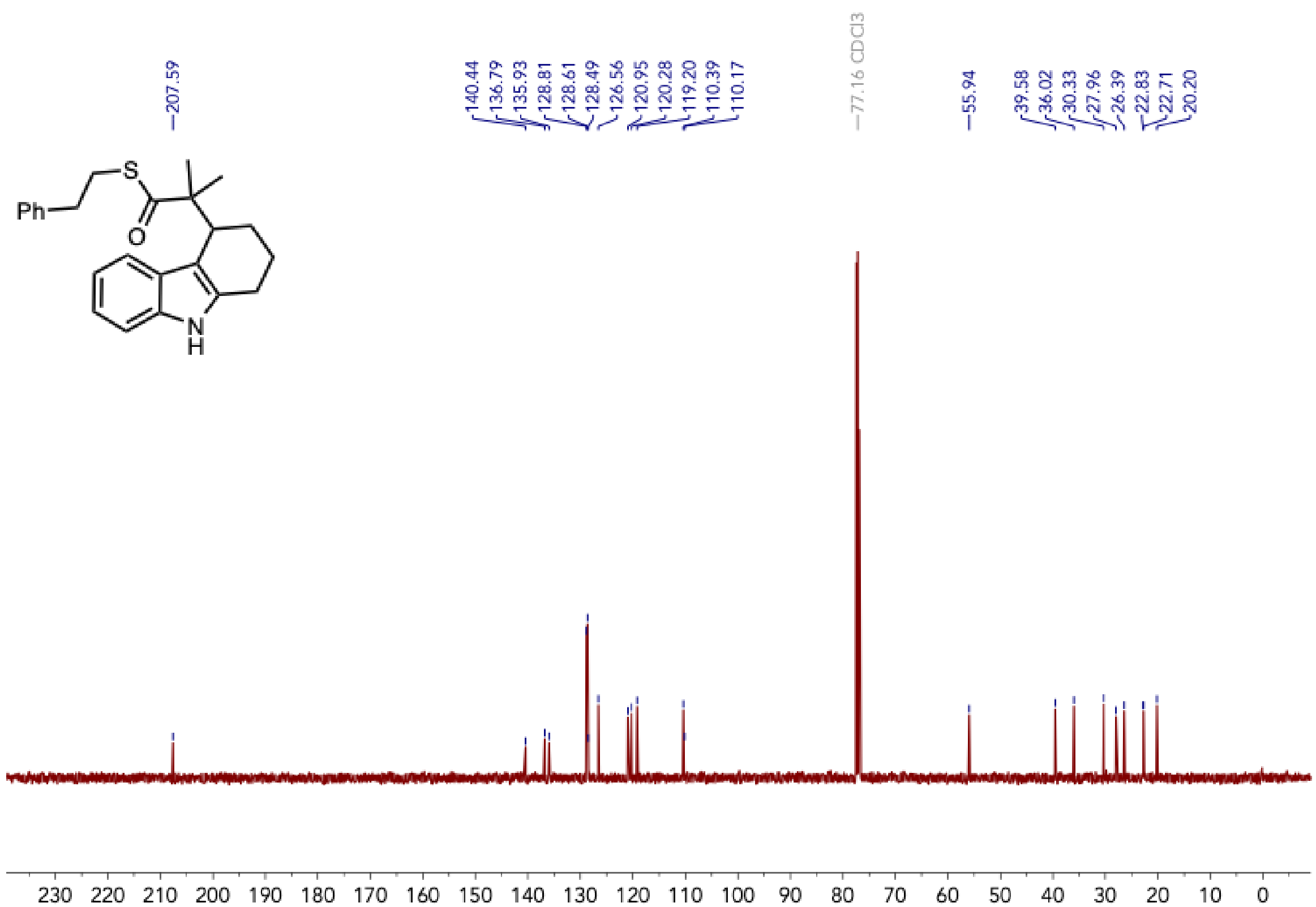

^{13}**C NMR** of **10c** (126 MHz, $CDCl_3$)

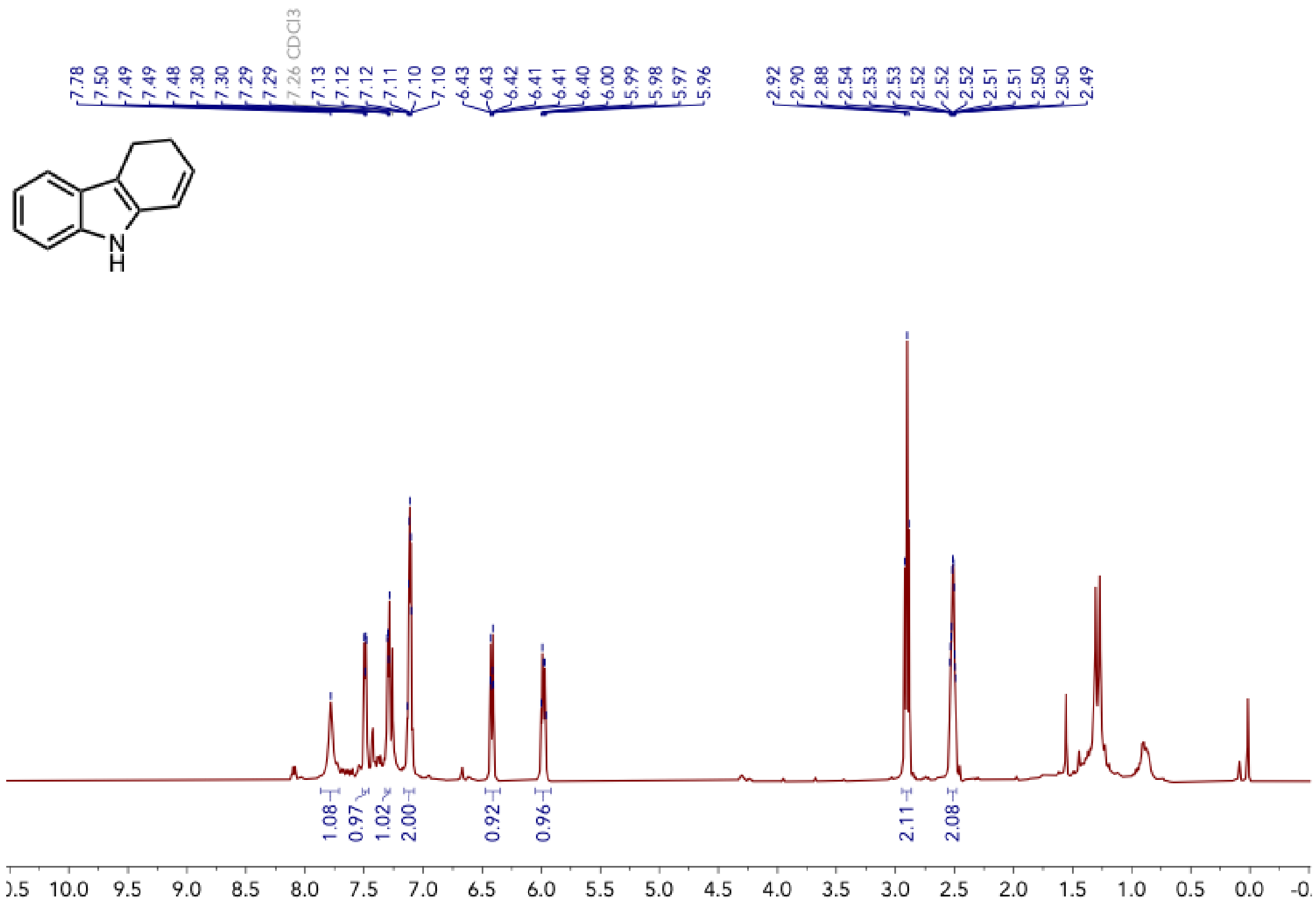

^{1}H NMR of **12a** (500 MHz, $CDCl_3$)

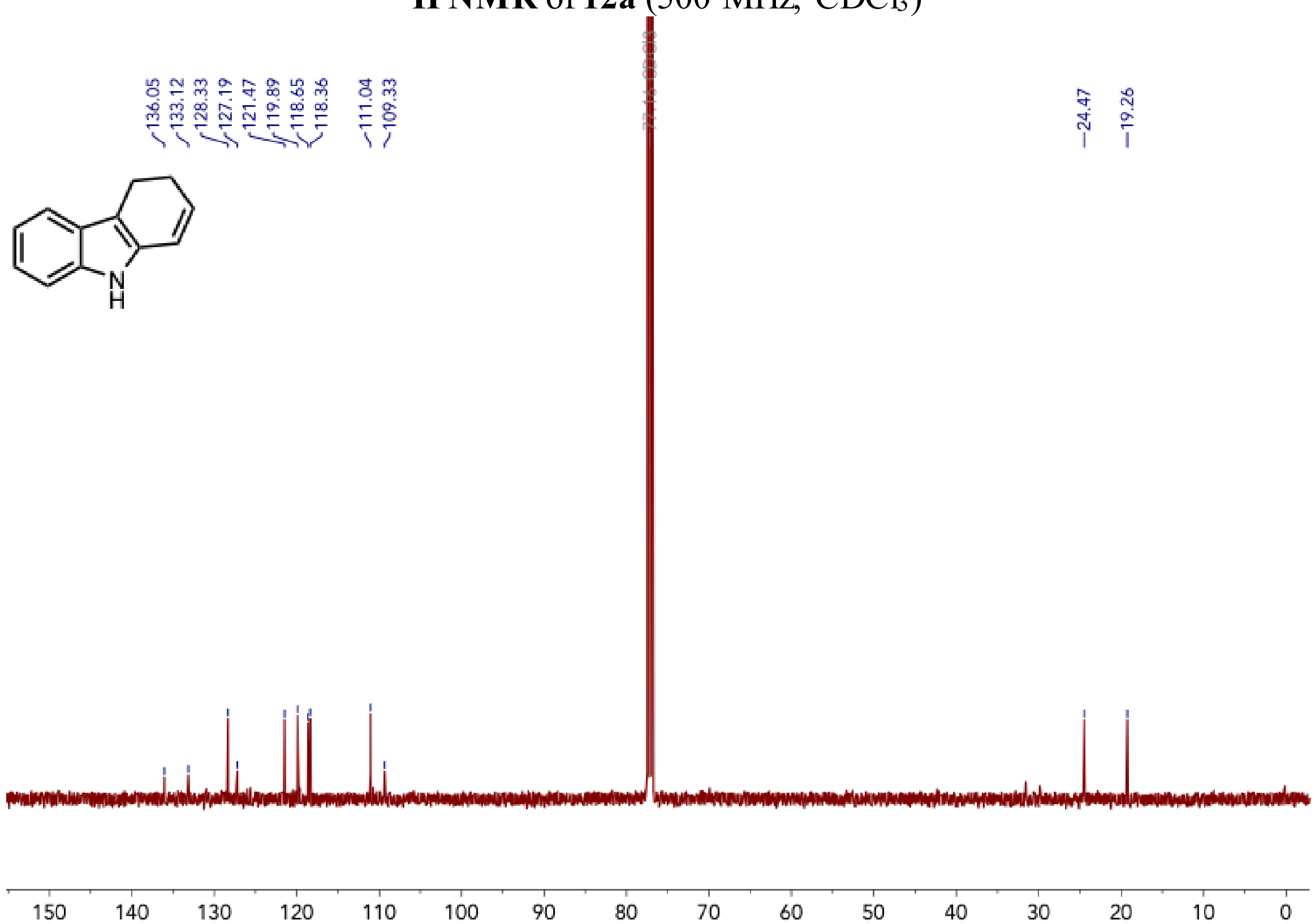

^{13}C NMR of **12a** (126 MHz, $CDCl_3$)

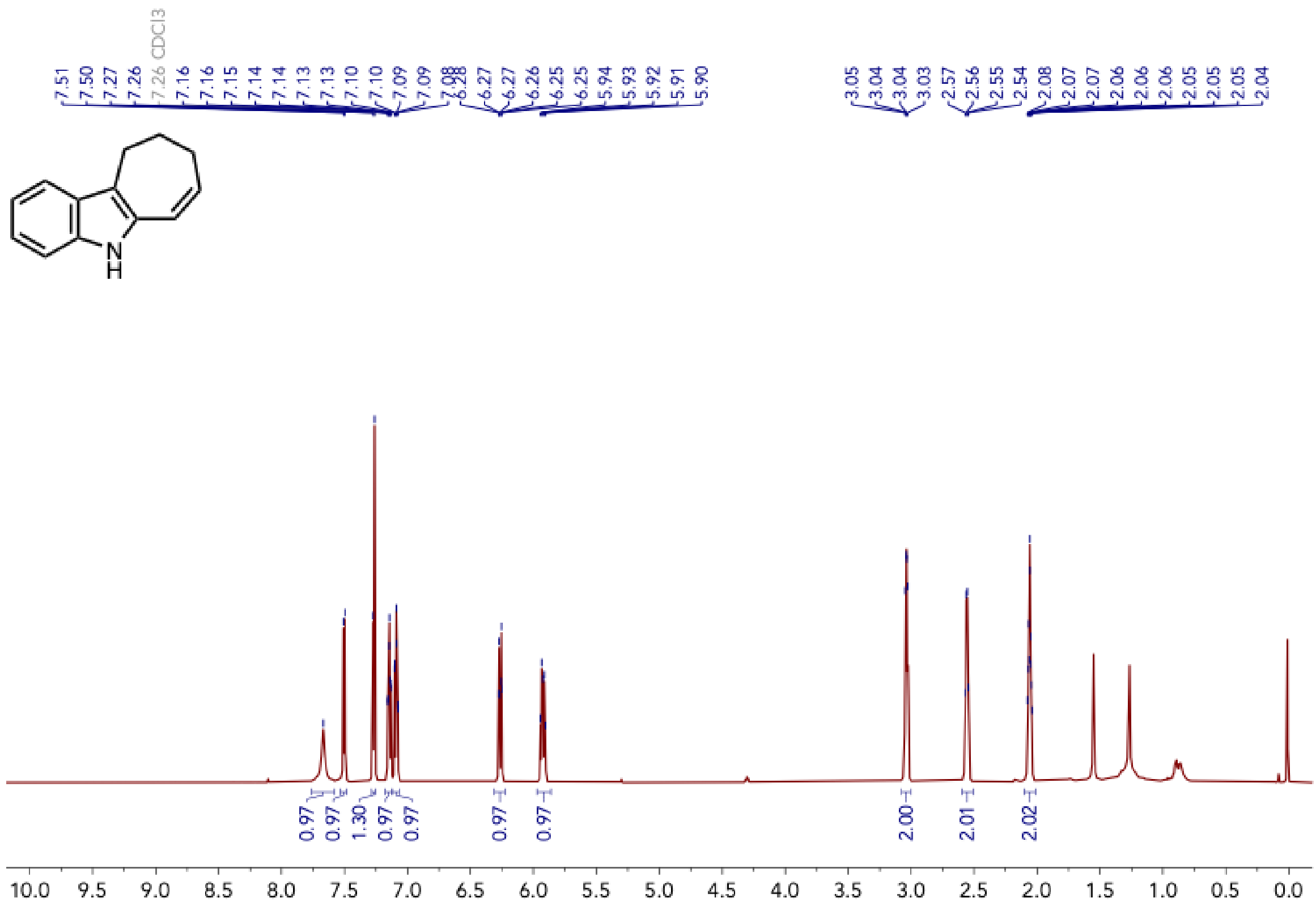

^{1}H NMR of **12b** (500 MHz, CDCl$_3$)

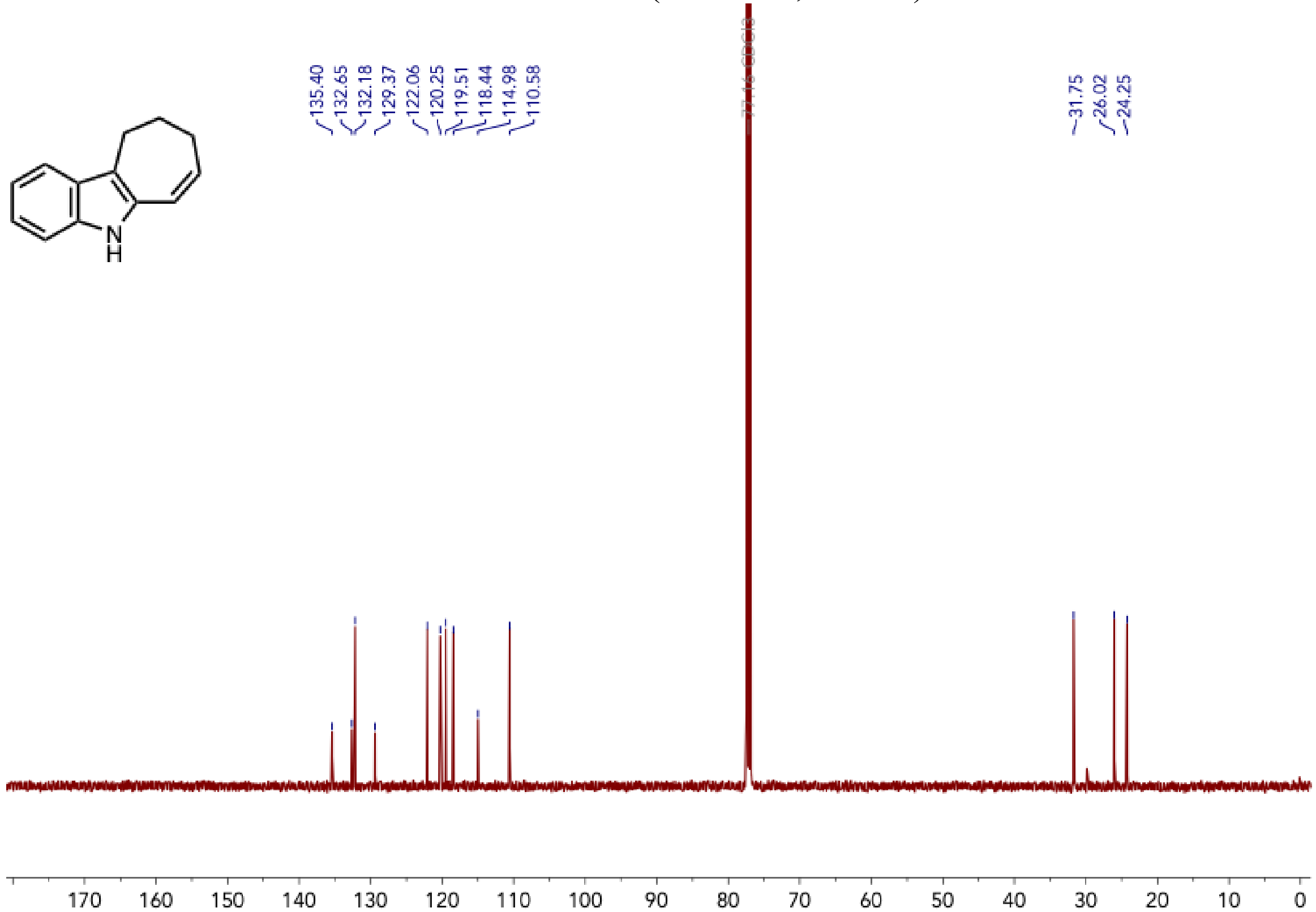

^{13}C NMR of **12b** (126 MHz, CDCl$_3$)

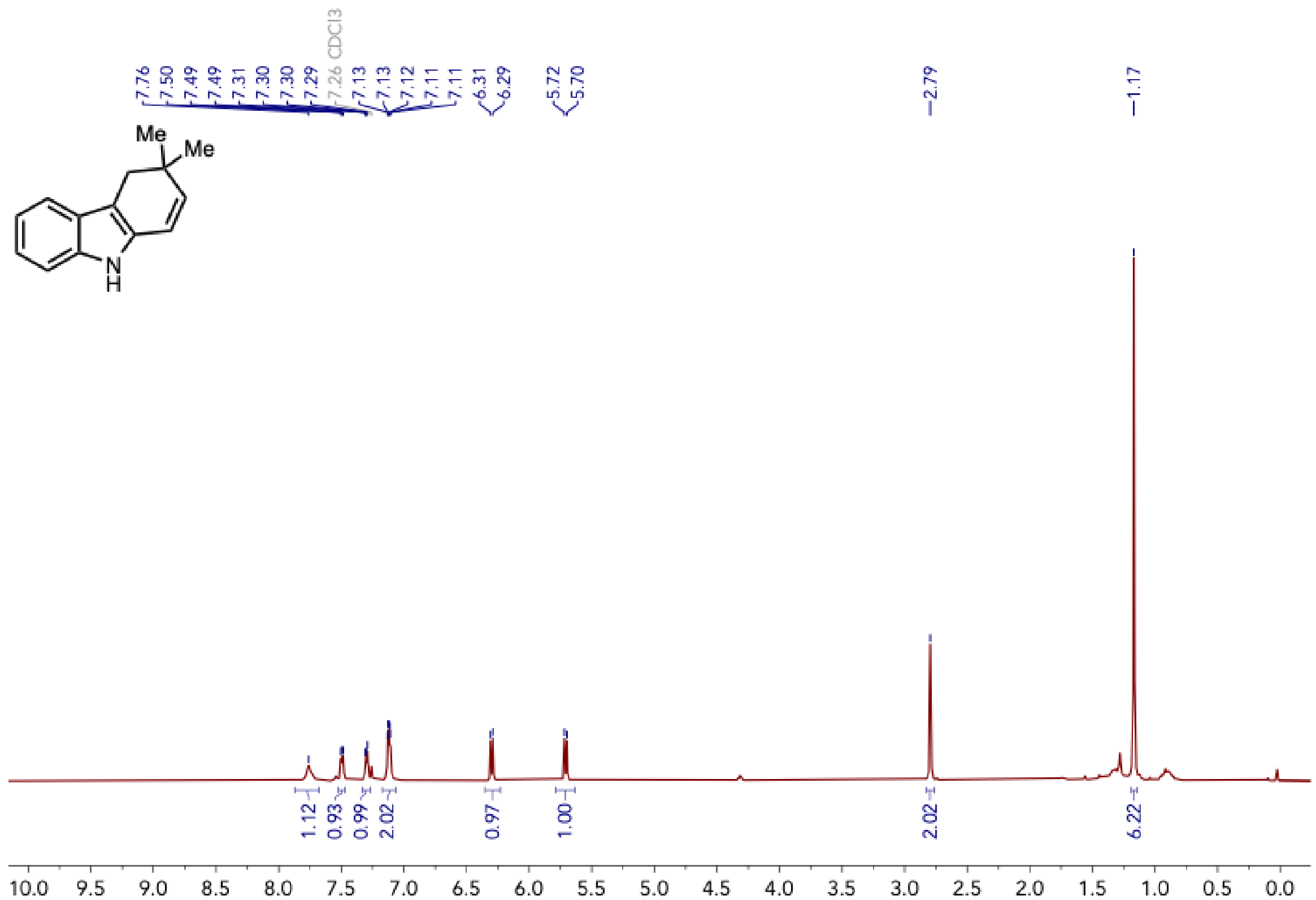

^{1}**H NMR** of **12c** (500 MHz, $CDCl_3$)

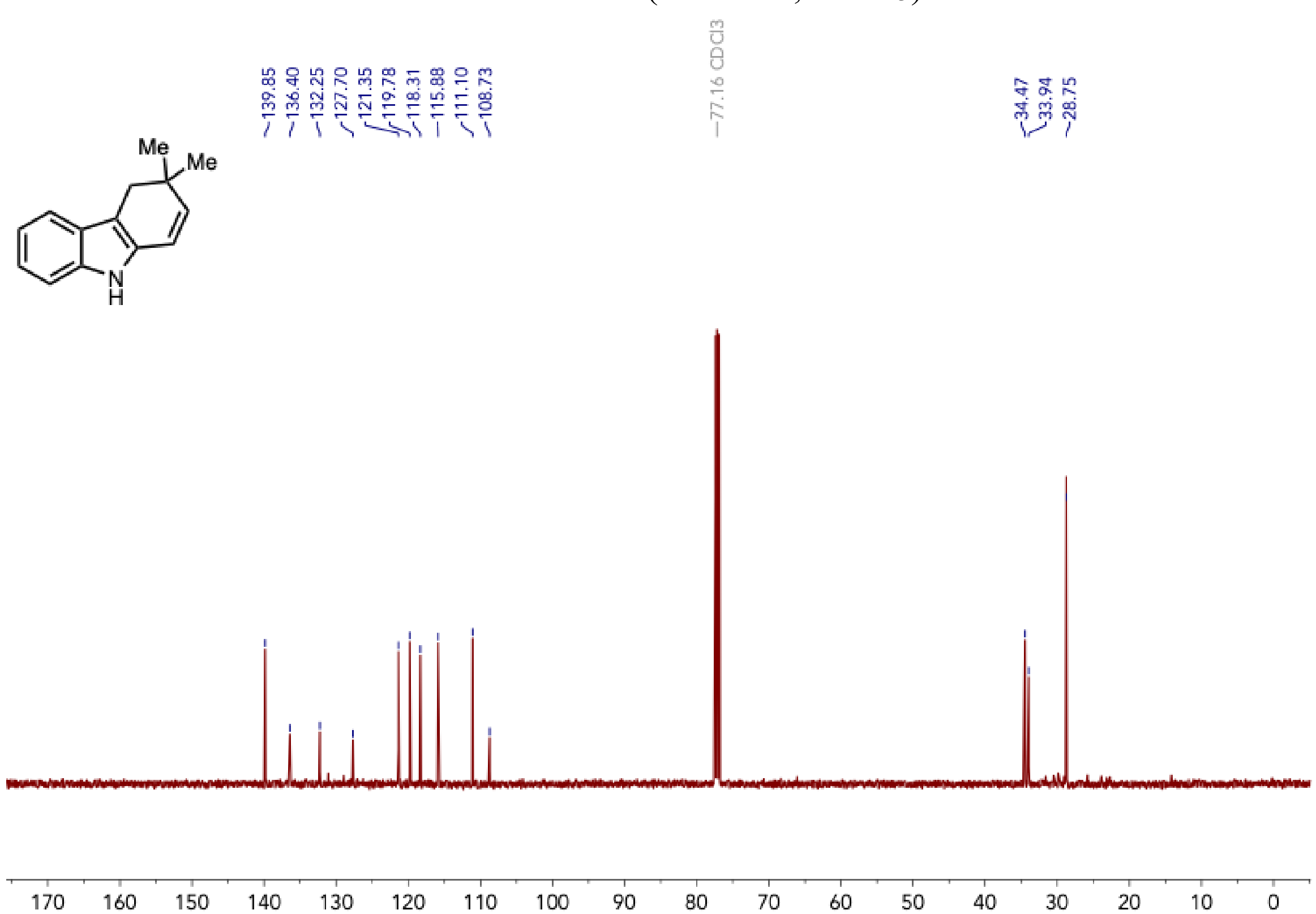

^{13}**C NMR** of **12c** (126 MHz, $CDCl_3$)

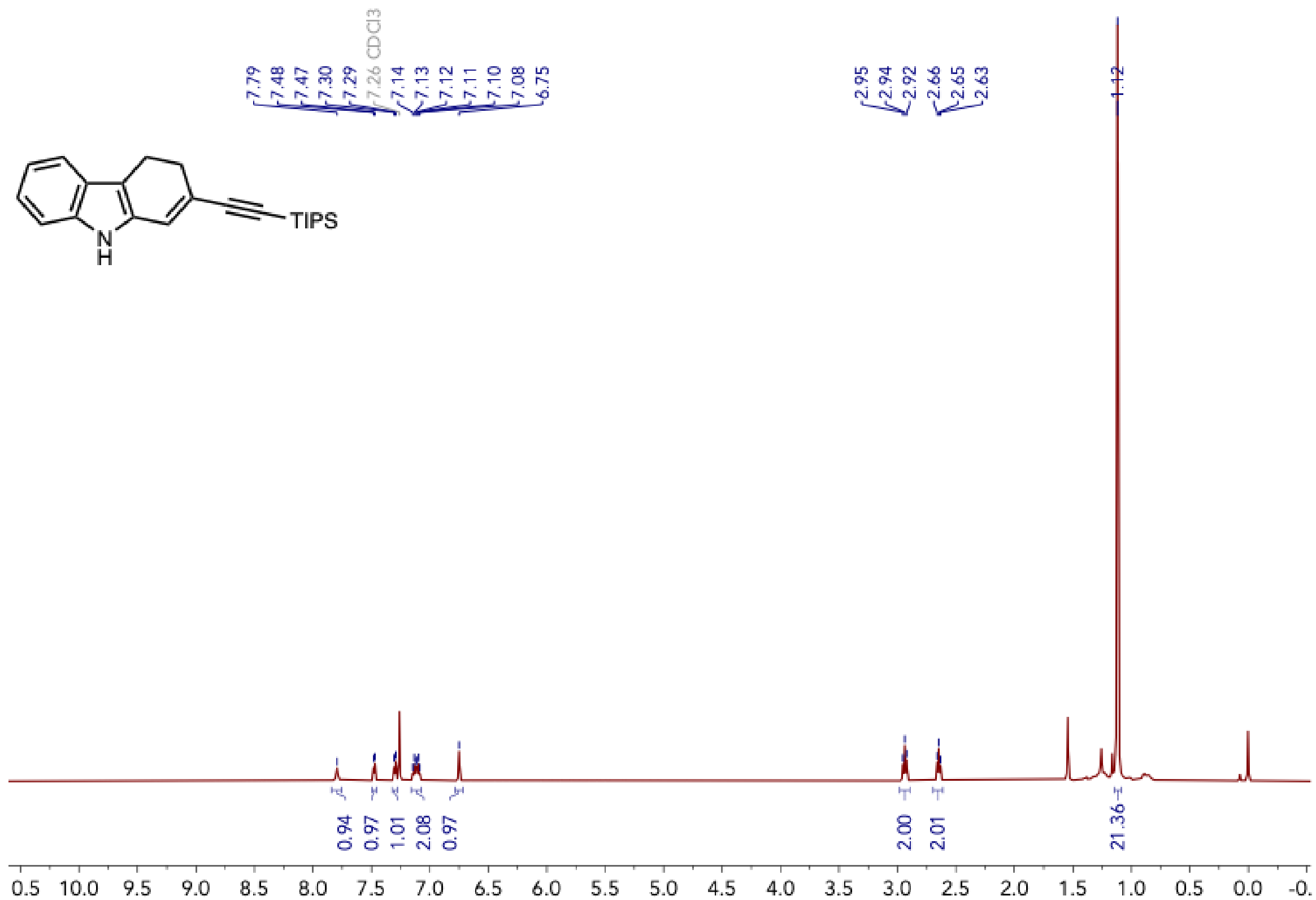

^{1}H NMR of **12d** (500 MHz, $CDCl_3$)

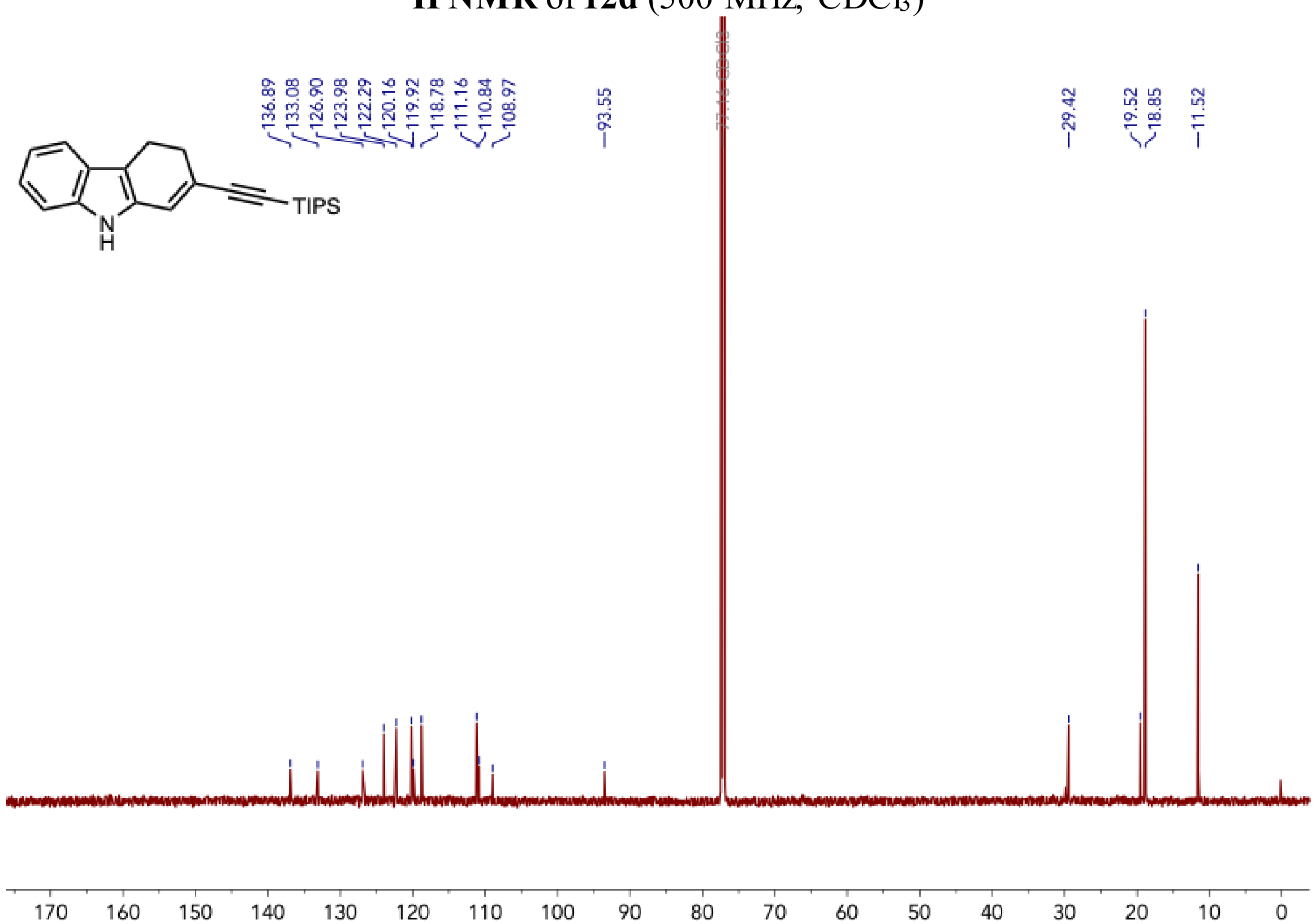

^{13}C NMR of **12d** (126 MHz, $CDCl_3$)

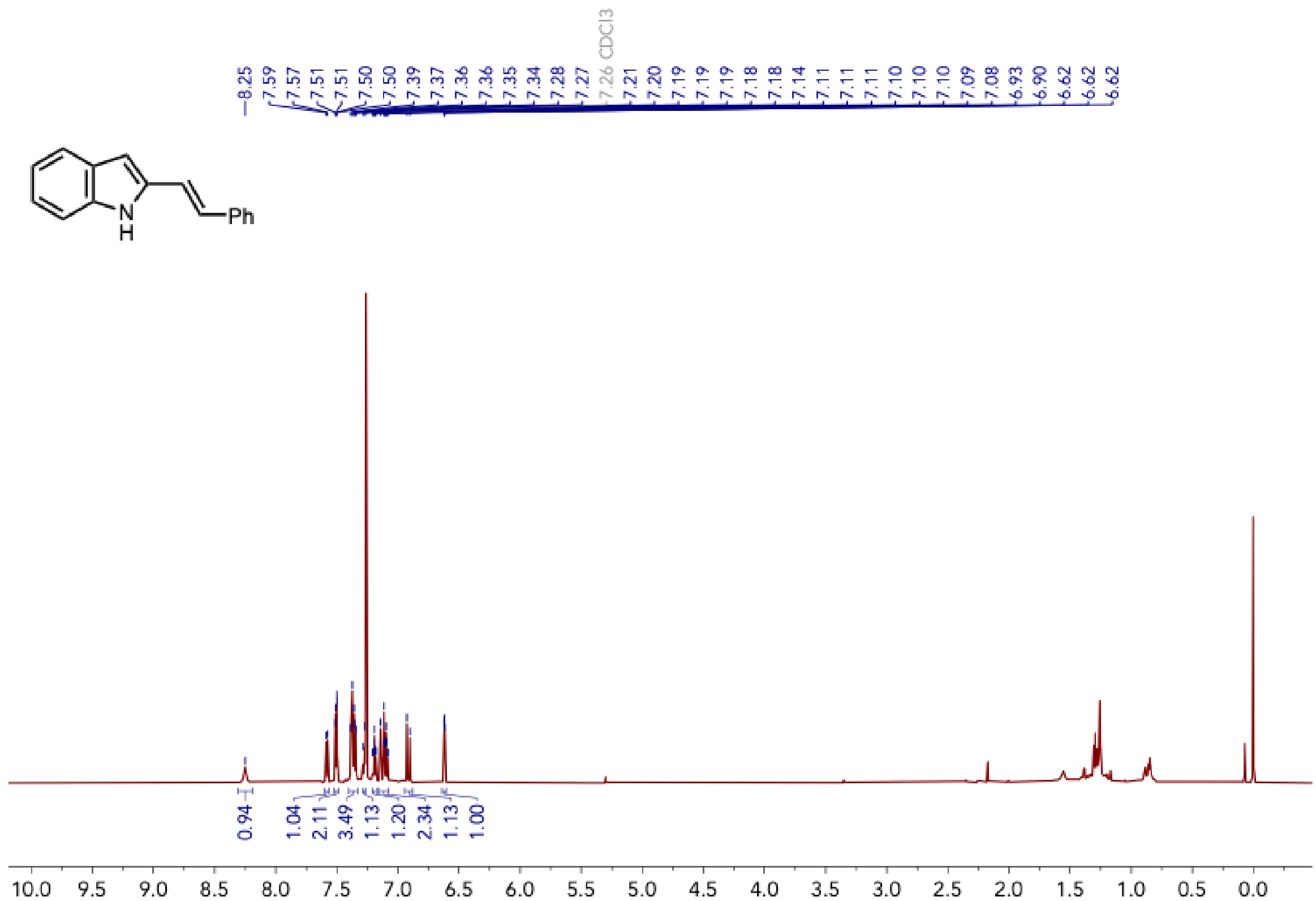

^{1}H NMR of **12e** (500 MHz, $CDCl_3$)

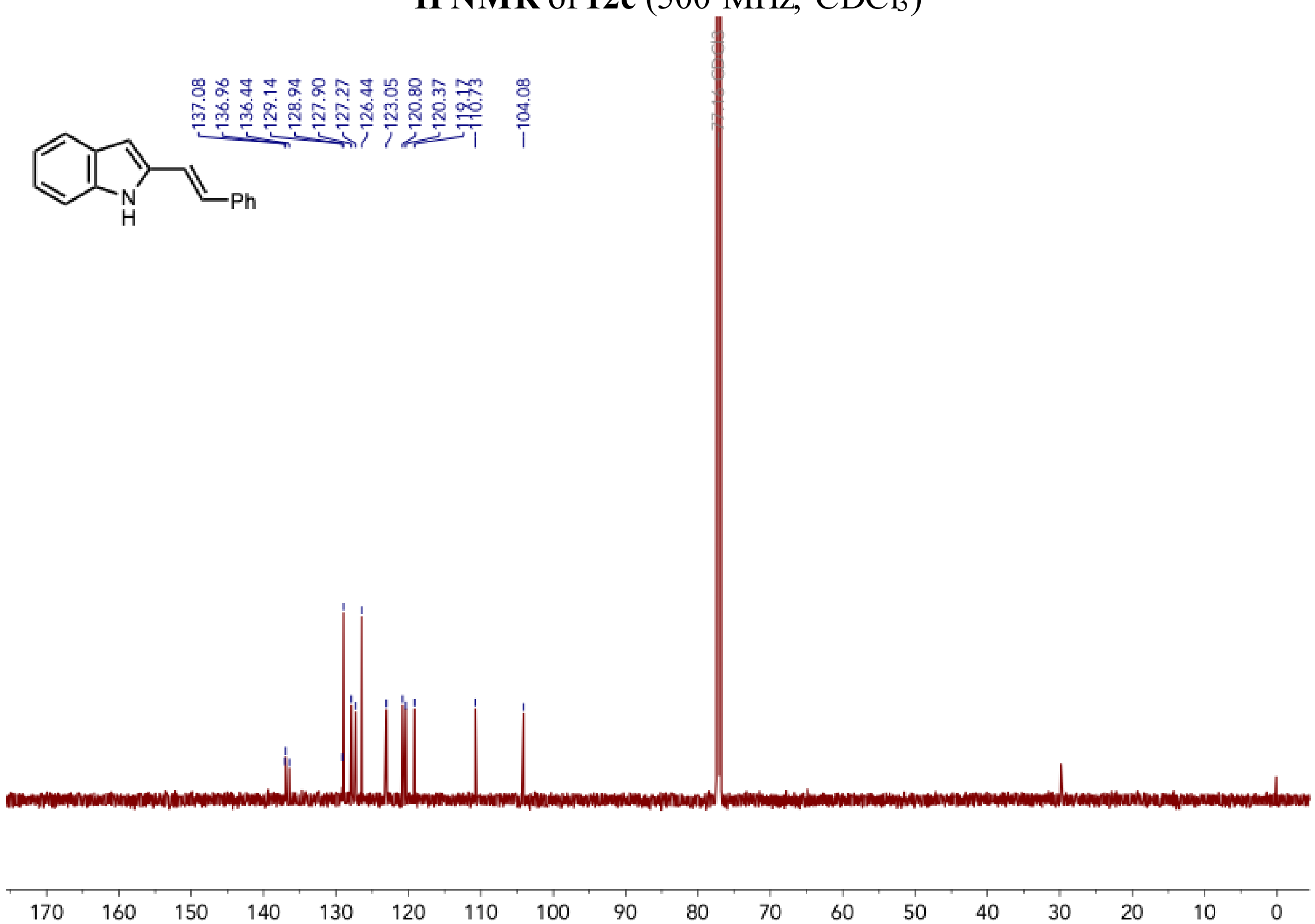

^{13}C NMR of **12e** (150 MHz, $CDCl_3$)

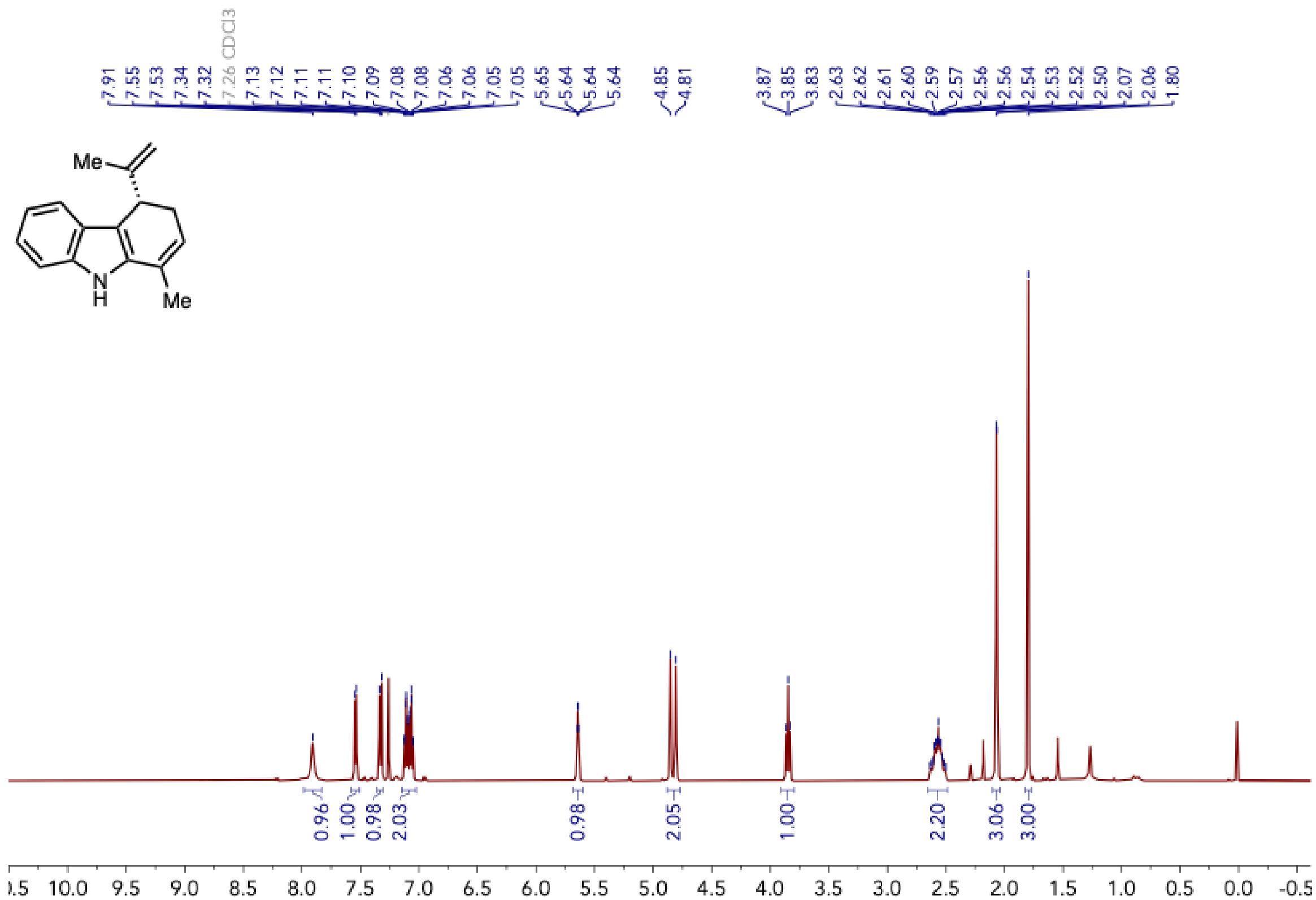

^{1}H NMR of **12f** (500 MHz, $CDCl_3$)

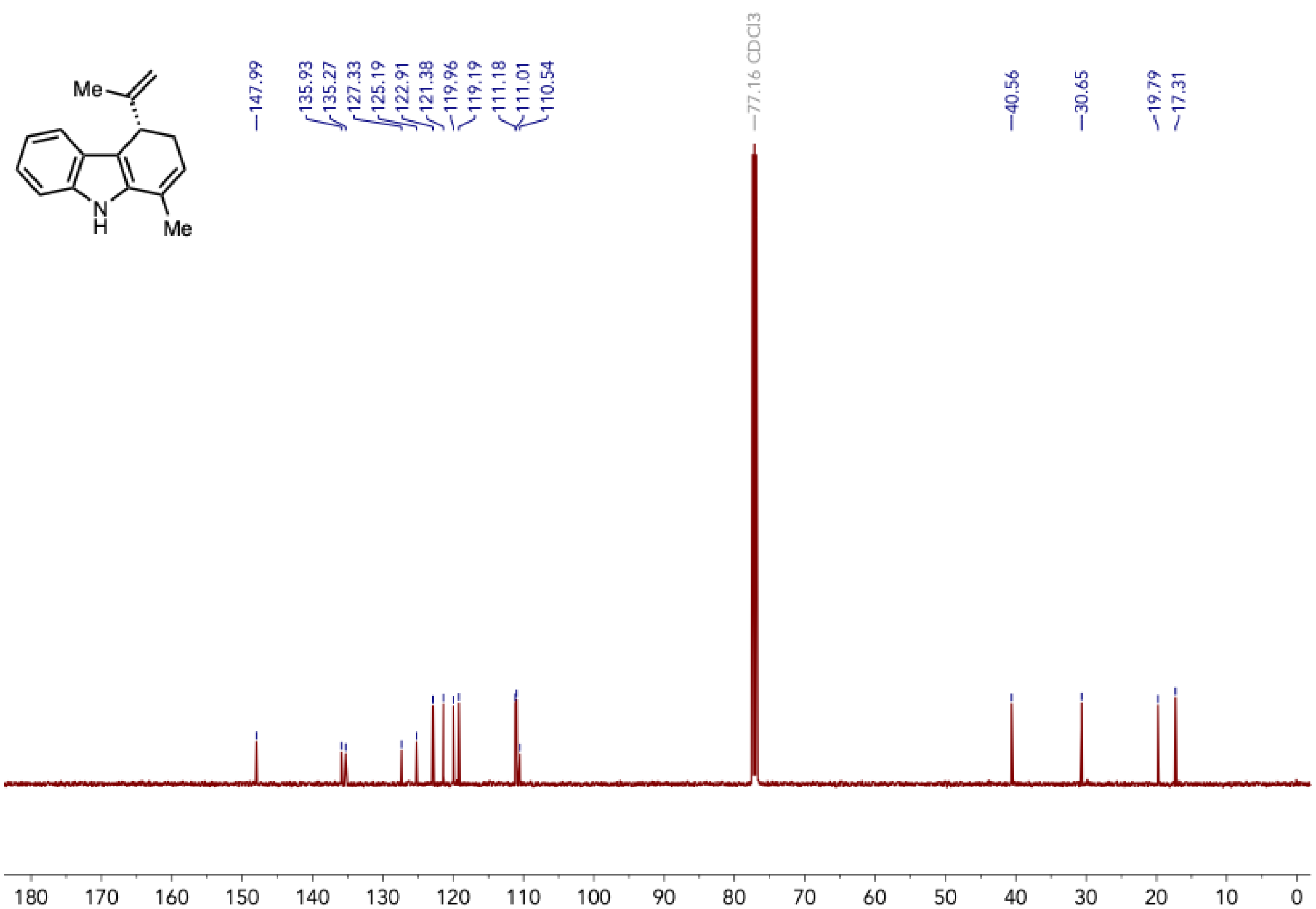

^{13}C NMR of **12f** (126 MHz, $CDCl_3$)

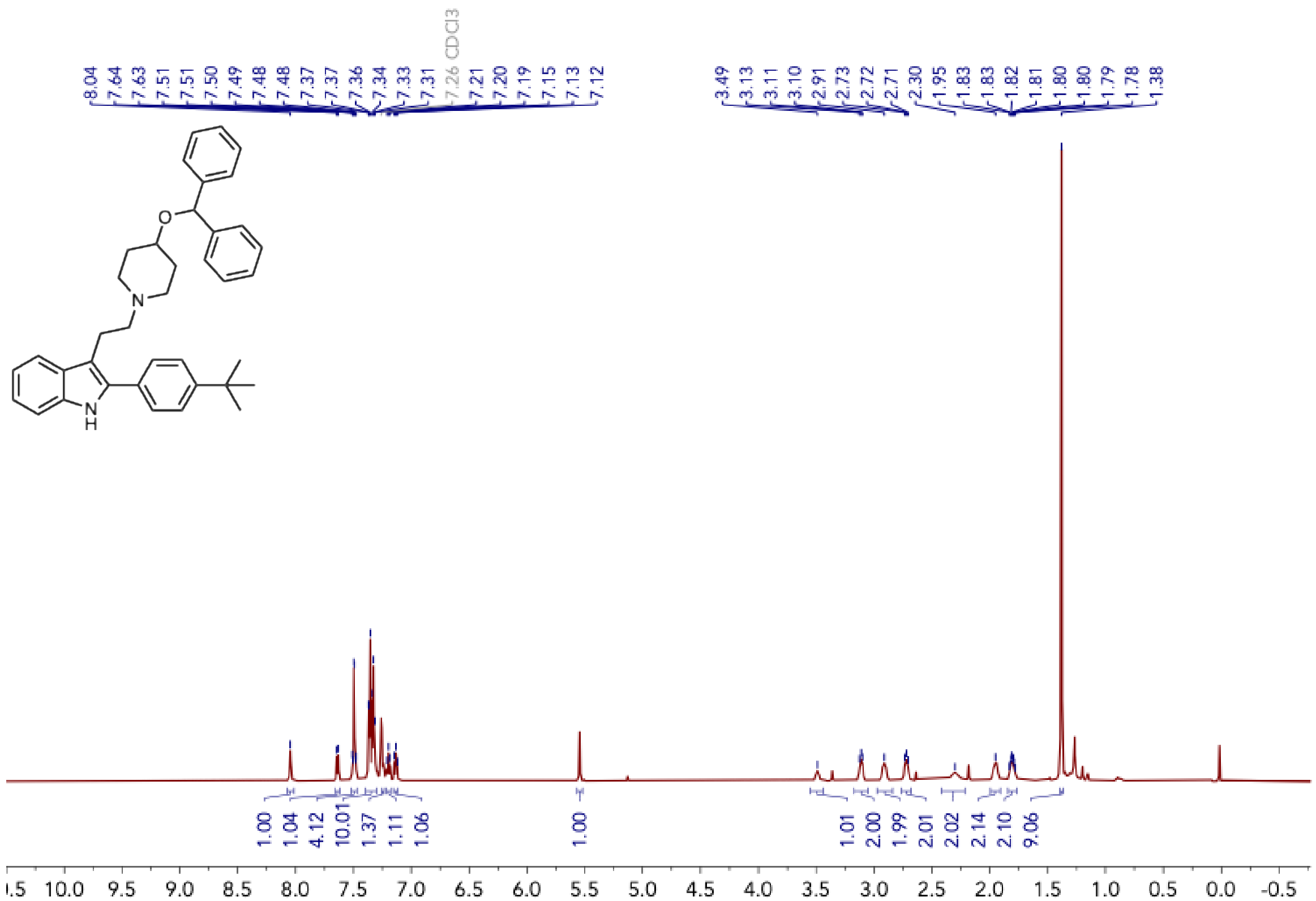

^{1}H NMR of **13** (600 MHz, $CDCl_3$)

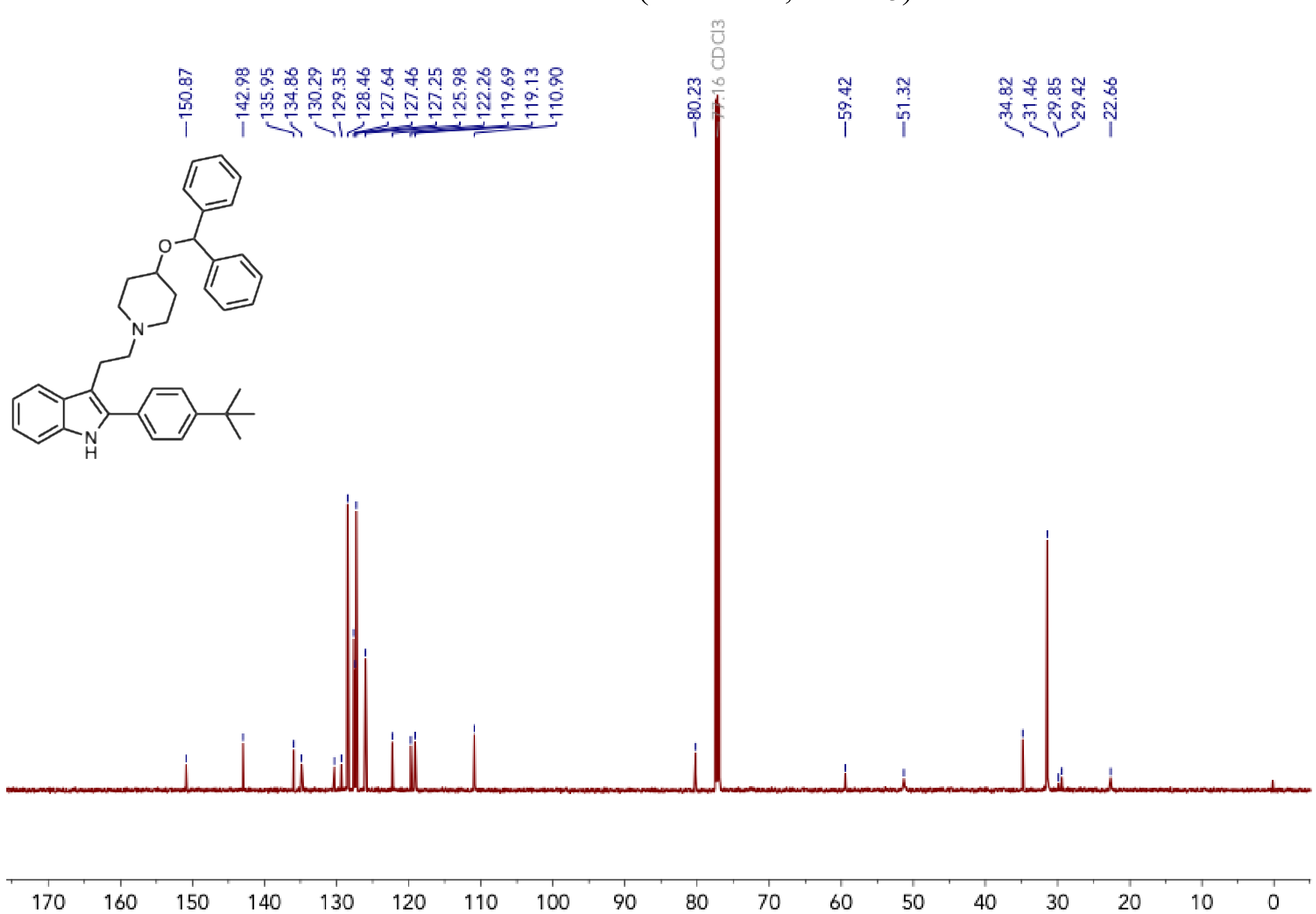

^{13}C NMR of **13** (150 MHz, $CDCl_3$)

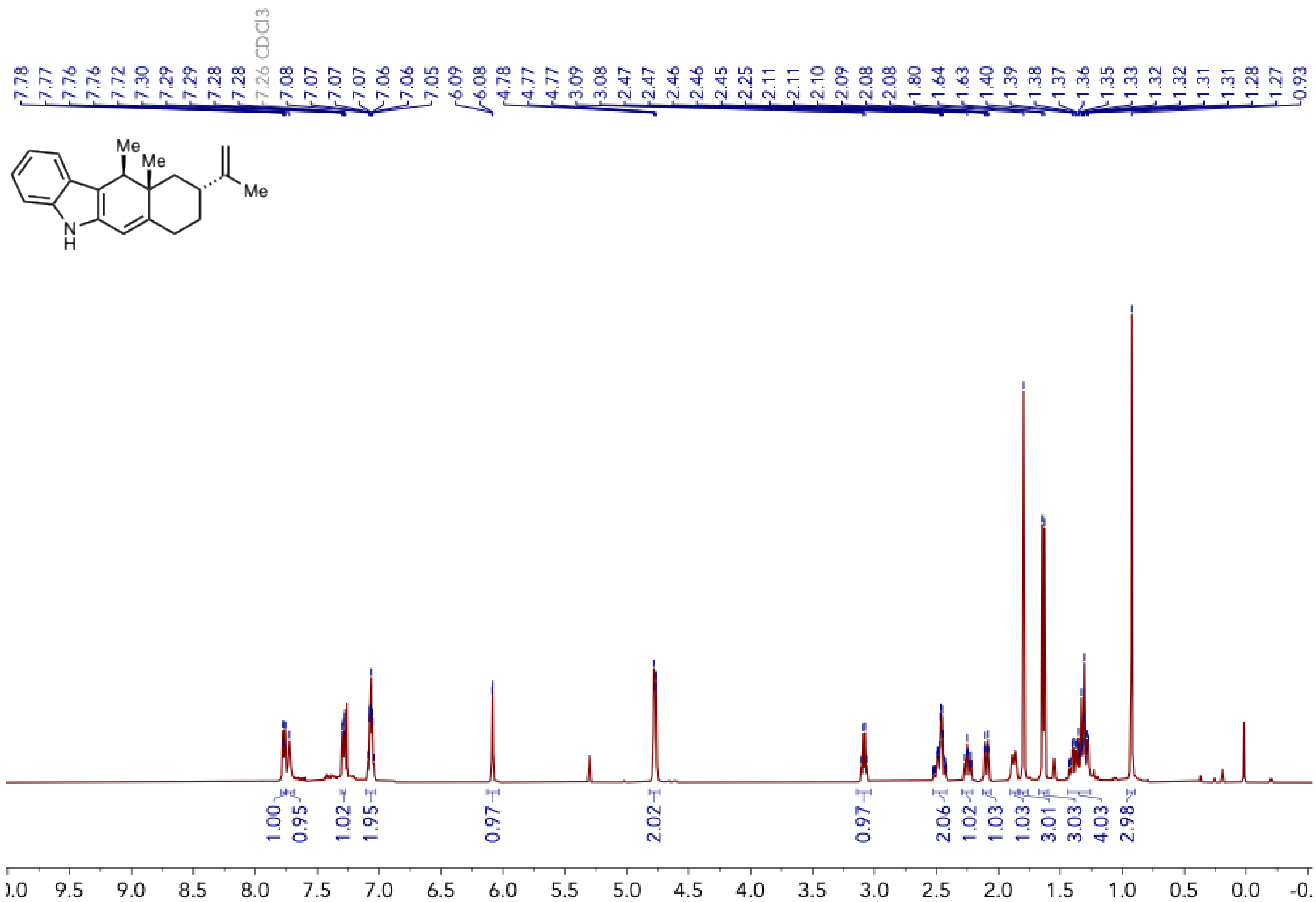

^{1}H NMR of **14** (500 MHz, $CDCl_3$)

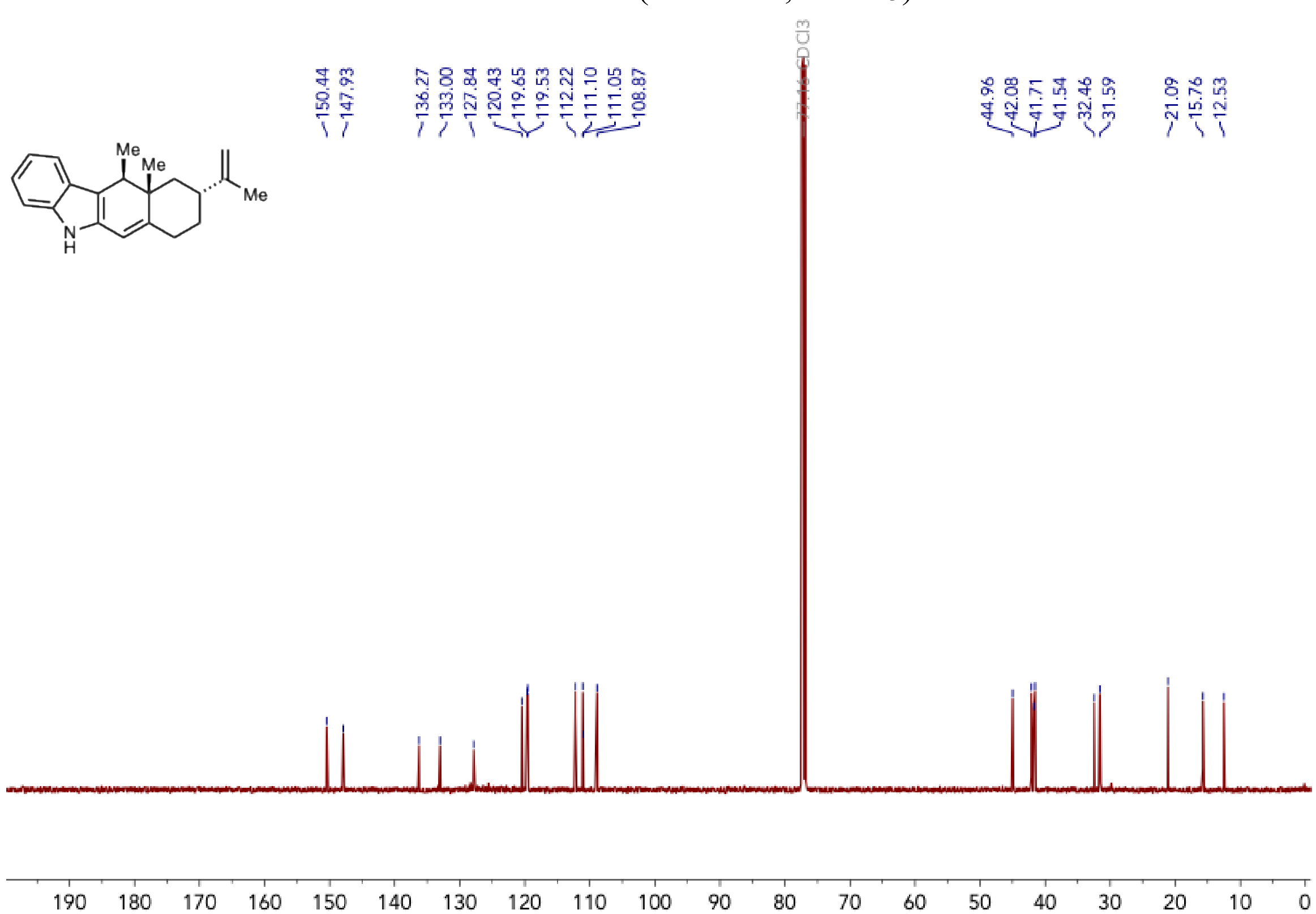

^{13}C NMR of **14** (126 MHz, $CDCl_3$)

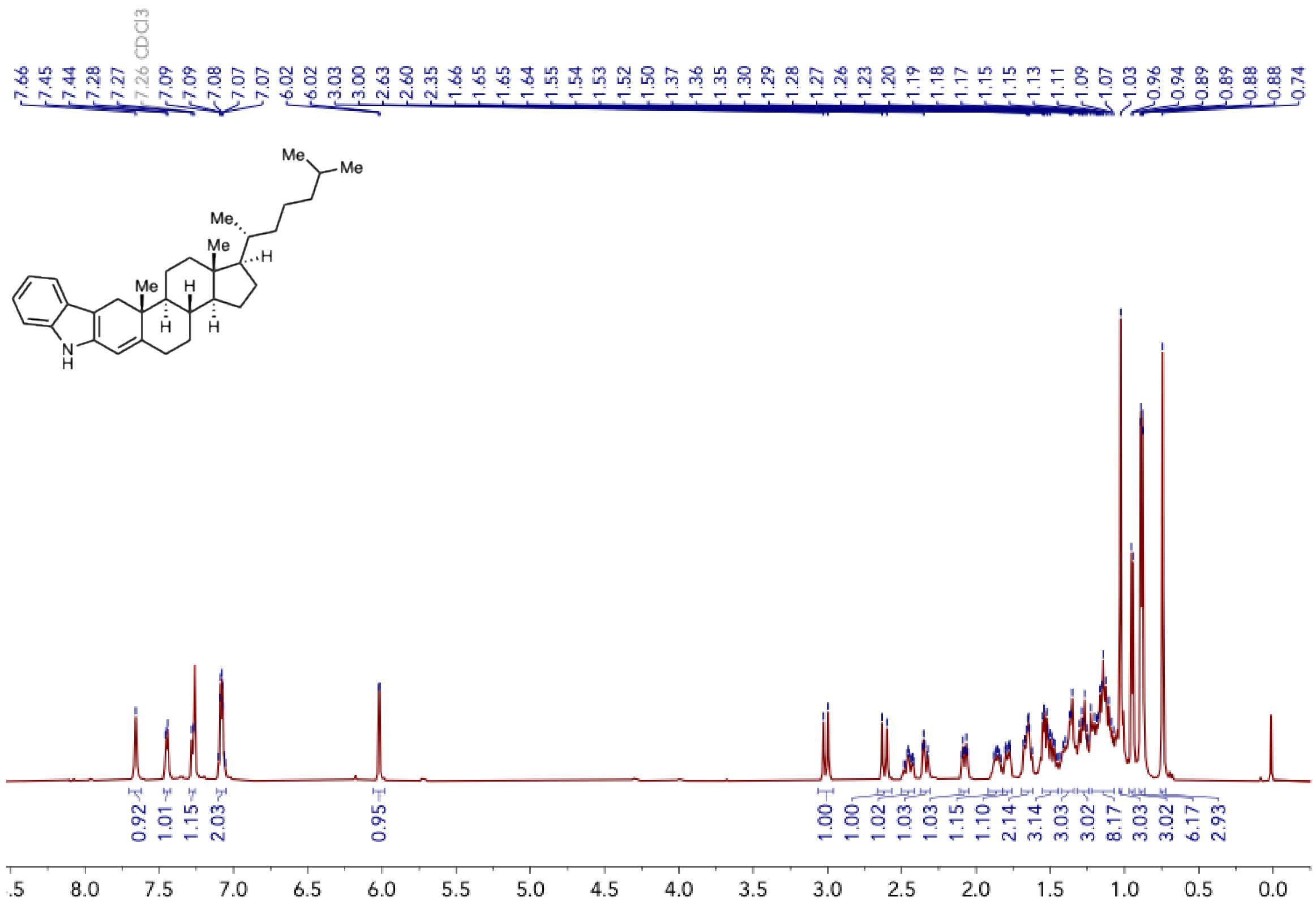

^{1}H NMR of **15** (500 MHz, $CDCl_3$)

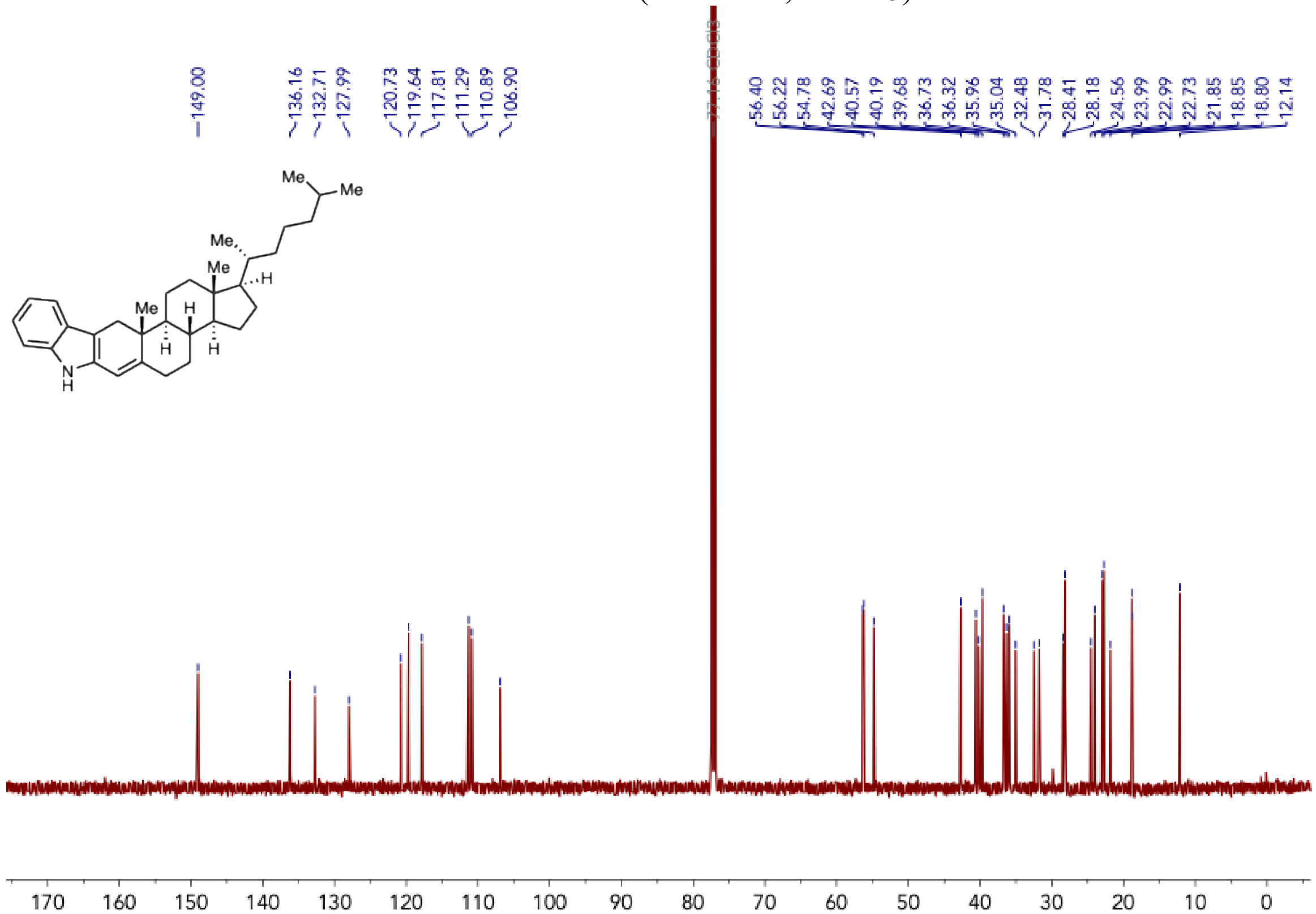

^{13}C NMR of **15** (126 MHz, $CDCl_3$)

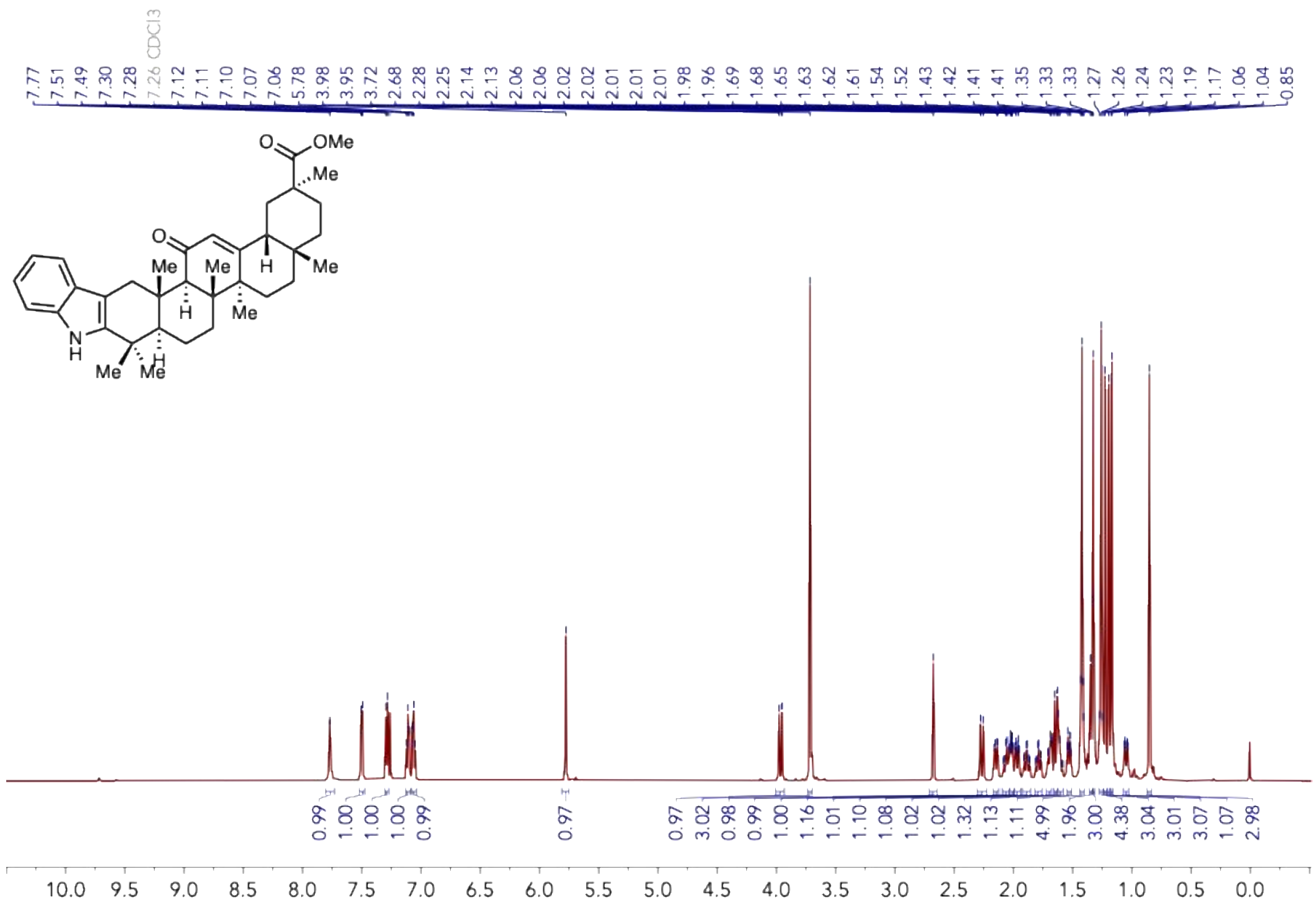

^{1}H NMR of **16** (600 MHz, $CDCl_3$)

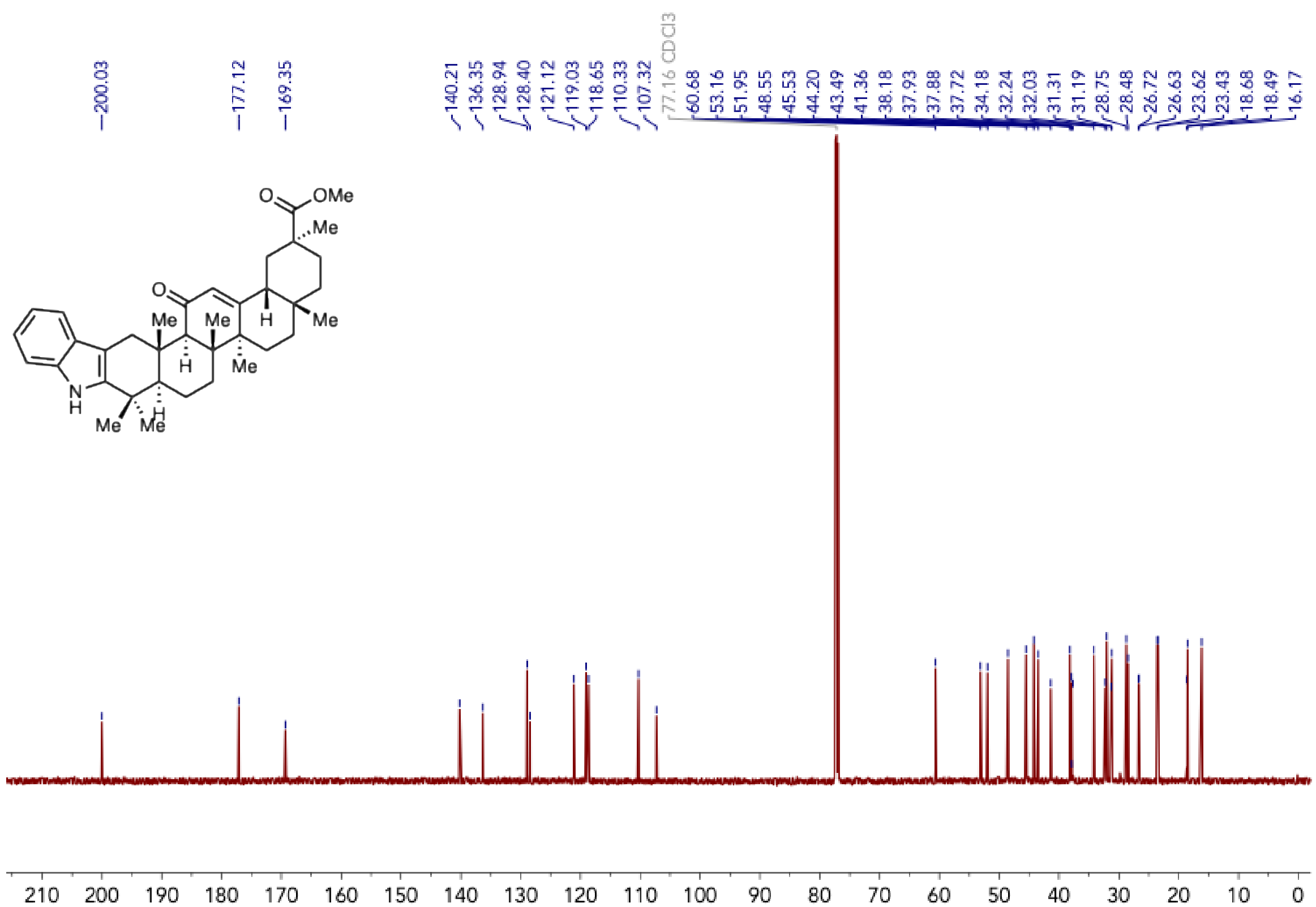

^{13}C NMR of **16** (150 MHz, $CDCl_3$)

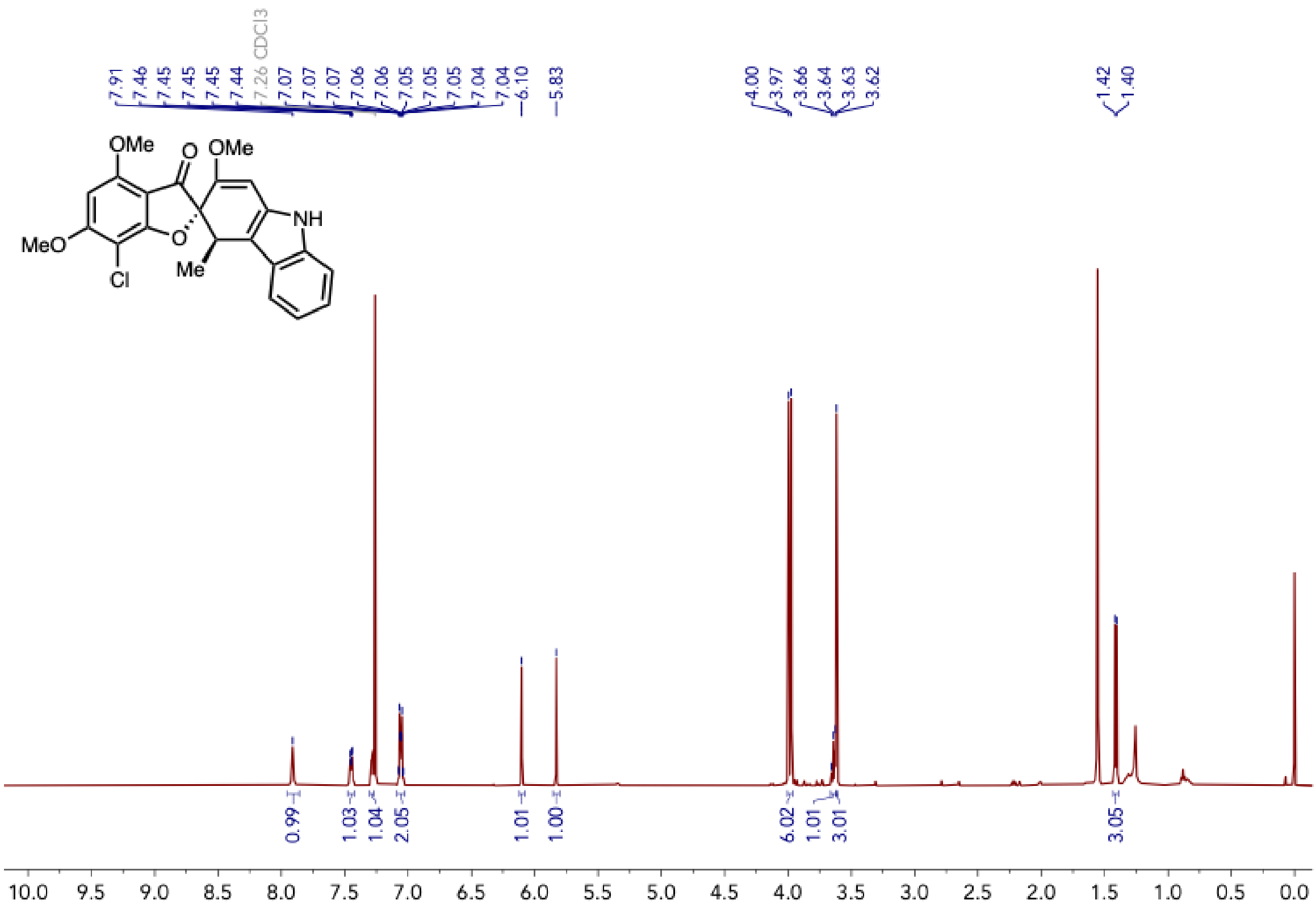

^{1}**H NMR** of **17** (500 MHz, $CDCl_3$)

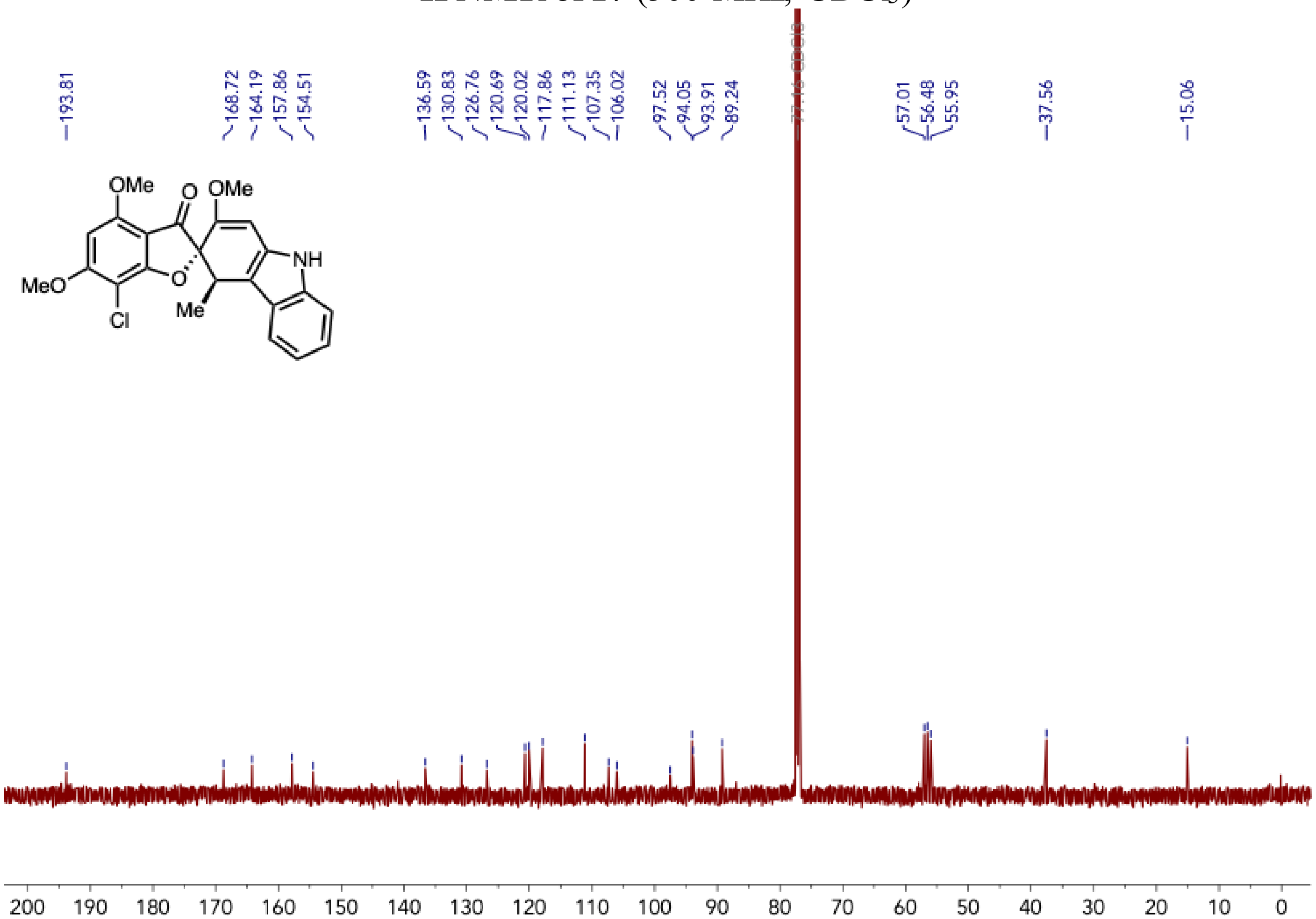

^{13}**C NMR** of **17** (126 MHz, $CDCl_3$)

6. HPLC Chromatograms

(S)-3-(tert-butyl)-2,3,4,9-tetrahydro-1H-carbazole (4c')

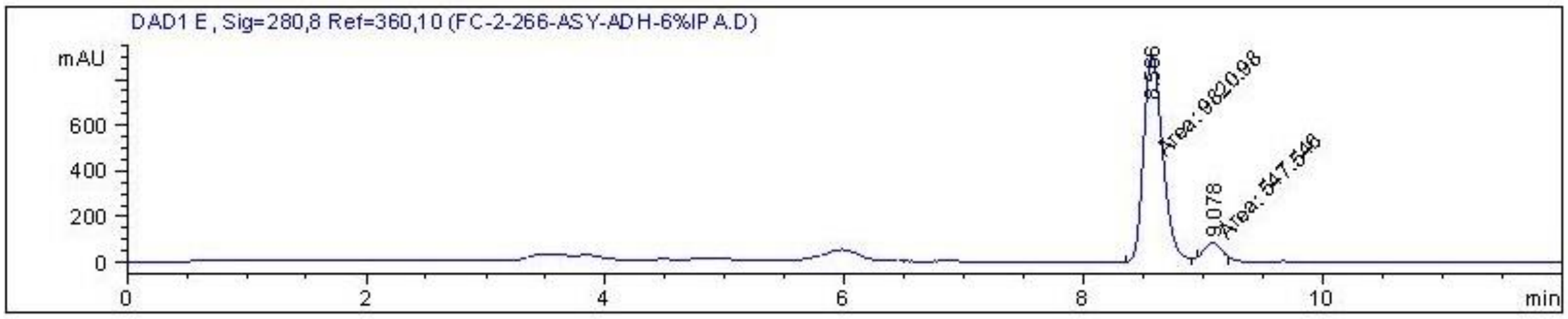

Signal 5: DAD1 E, Sig=280,8 Ref=360,10

Peak #	RetTime [min]	Type	Width [min]	Area [mAU*s]	Height [mAU]	Area %
1	8.566	MM	0.1820	9820.98438	899.48181	94.7192
2	9.078	MM	0.1469	547.54614	62.12096	5.2808

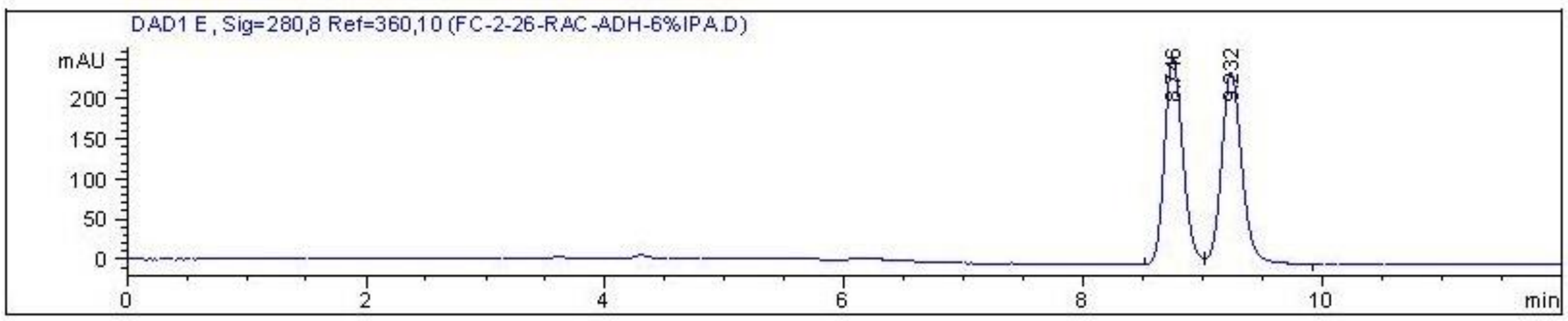

Signal 5: DAD1 E, Sig=280,8 Ref=360,10

Peak #	RetTime [min]	Type	Width [min]	Area [mAU*s]	Height [mAU]	Area %
1	8.746	BV	0.1645	2749.23438	257.54324	49.3808
2	9.232	VB	0.1814	2818.17798	239.25665	50.6192
Totals :				5567.41235	496.79990	

7. References

1. Ben Saida, A.; Chardon, A.; Osi, A.; Tumanov, N.; Wouters, J.; Adjieufack, A. I.; Champagne, B.; Berionni, G. Pushing the Lewis Acidity Boundaries of Boron Compounds with Non-Planar Triarylboranes Derived from Triptycenes. *Angew. Chem. Int. Ed.* **2019**, *58*, 16889–16893.

2. Li, L.; Carpaneto, F.; Chen, P.-P.; Houk, K. N.; Rawal, V. H. Regiospecific α-Arylation of Diverse Carbonyl Compounds Using an Organobismuth Transporter. *Nat. Chem.* **2026**. https://doi.org/10.1038/s41557-026-02231-5.

3. Misal Castro, L. C.; Sultan, I.; Nishi, K.; Tsurugi, H.; Mashima, K. Direct Synthesis of Indoles from Azoarenes and Ketones with Bis(Neopentylglycolato)Diboron Using 4,4′-Bipyridyl as an Organocatalyst. *J. Org. Chem.* **2021**, *86*, 3287–3299.

4. Sivaganesan, P.; Elanchezhian, C.; Bansal, D.; Das, M. K.; Chaudhuri, S. An Ion-Pair as a Superacidic Precatalyst for the Synthesis of Indole Alkaloids: A Novel Entry into the Fischer Indole Synthesis. *Green Chem.* **2026**, *28*, 424–432.

5. Schmidt, M. A. Effect of Terminal Alkylation of Aryl and Heteroaryl Hydrazines in the Fischer Indole Synthesis. *J. Org. Chem.* **2022**, *87*, 1941–1960.

6. Nirogi, R. V. S.; Konda, J. B.; Kambhampati, R.; Shinde, A.; Bandyala, T. R.; Gudla, P.; Kandukuri, K. K.; Jayarajan, P.; Kandikere, V.; Dubey, P. K. N,N-Dimethyl-[9-(Arylsulfonyl)-2,3,4,9-Tetrahydro-1H-Carbazol-3-Yl]Amines as Novel, Potent and Selective 5-HT6 Receptor Antagonists. *Bioorg. Med. Chem. Lett.* **2012**, *22*, 6980–6985.

7. Yi, Z.; Chen, F.; He, Y.; Fan, Q. Asymmetric Hydrogenation of Tetrahydro- *γ* -Carboline Derivatives with Phosphine-Free Ruthenium Diamine Catalysts. *Adv. Synth. Catal.* **2025**, *367*, e70029.

8. Long, H.; Xu, K.; Chen, S.; Lin, J.; Wu, D.; Wu, B.; Tian, X.; Ackermann, L. Nickel-Catalyzed Intramolecular Direct Arylation of Imines toward Diverse Indoles. *Org. Lett.* **2019**, *21*, 3053–3056.

9. Muralirajan, K.; Cheng, C. Regioselective Synthesis of Indoles *via* Rhodium-Catalyzed C–H Activation Directed by an *In-Situ* Generated Redox-Neutral Group. *Adv. Synth. Catal.* **2014**, *356*, 1571–1576.

10. Chen, J.; Chen, Z.; Zhao, H.; Zhang, T.; Wang, W.; Zou, Y.; Zhang, X.; Yan, M. Intramolecular Addition of Diarylmethanols to Imines Promoted by KOt-Bu/DMF: A New Synthetic Approach to Indole Derivatives. *Org. Biomol. Chem.* **2016**, *14*, 4071–4076.

11. Hu, W.; Yang, T.; Chen, S.; Yuan, L.; Shang, Y. Efficient Accessibility of Indole and Pyrrole Nuclei *via* Late-Stage Aryl C–H Activation of Drug Molecules Promoted by Thianthrenium Salts. *Chem. Commun.* **2024**, *60* (88), 12916–12919.

12. Yan, H.; Wang, H.; Li, X.; Xin, X.; Wang, C.; Wan, B. Rhodium-Catalyzed C–H Annulation of Nitrones with Alkynes: A Regiospecific Route to Unsymmetrical 2,3-Diaryl-Substituted Indoles. *Angew. Chem. Int. Ed.* **2015**, *54*, 10613–10617.

13. Nakamura, Y.; Ukita, T. Construction of Heterocyclic Compounds by Use of α-Diazophosphonates: New One-Pot Syntheses of Indoles and Isocoumarins. *Org. Lett.* **2002**, *4* (14), 2317–2320.

14. Yang, Q.; Marchini, M.; Xiao, W.; Ceroni, P.; Bandini, M. Visible-Light-Induced Direct Photocatalytic Carboxylation of Indoles with CBr_4/MeOH. *Chem. – Eur. J.* **2015**, *21*, 18052–18056.

15. Chebieb, A.; Kim, Y. G.; Cha, J. K. Synthesis of Indoles from o-Haloanilines. *J. Org. Chem.* **2023**, *88*, 10164–10170.

16. Iwama, T.; Birman, V. B.; Kozmin, S. A.; Rawal, V. H. Regiocontrolled Synthesis of Carbocycle-Fused Indoles via Arylation of Silyl Enol Ethers with o-Nitrophenylphenyliodonium Fluoride. *Org. Lett.* **1999**, *1*, 673–676.

17. Yang, G.; Pan, J.; Ke, Y.; Liu, Y.; Zhao, Y. Tandem Catalytic Indolization/Enantioconvergent Substitution of Alcohols by Borrowing Hydrogen to Access Tricyclic Indoles. *Angew. Chem. Int. Ed.* **2021**, *60*, 20689–20694.

18. Steuernagel, D.; Wagenknecht, H. Photocatalytic Synthesis of Acetals and Ketals from Aldehydes and Silylenolethers without the Use of Acids. *Chem. Eur. J.* **2023**, *29*, e202203767.

19. Chen, S.; Liao, Y.; Zhao, F.; Qi, H.; Liu, S.; Deng, G.-J. Palladium-Catalyzed Direct Arylation of Indoles with Cyclohexanones. *Org. Lett.* **2014**, *16*, 1618–1621.

20. Muniraj, N.; Prabhu, K. R. Cobalt(III)−Catalyzed C−H Activation: A Secondary Amide Directed Decarboxylative Functionalization of Alkynyl Carboxylic Acids Wherein Amide NH-group Remains Unreactive. *Adv. Synth. Catal.* **2018**, *360*, 1370–1375.

21. Cain, C. M.; Cousins, R. P. C.; Coumbarides, G.; Simpkins, N. S. Asymmetric Deprotonation of Prochiral Ketones Using Chiral Lithium Amide Bases. *Tetrahedron* **1990**, *46*, 523–544.
